# Enantioselective Pd-Catalyzed Electrochemical Dearomative Allylation of Tropones: Construction of All‑C Quaternary Stereocenters

**DOI:** 10.1021/acs.orglett.6c00796

**Published:** 2026-03-18

**Authors:** Giulia Monda, Sofia Kiriakidi, Olalla Nieto Faza, Andrea Mazzanti, Giulio Bertuzzi, Marco Bandini

**Affiliations:** † Dipartimento di Chimica “Giacomo Ciamician”, Alma Mater Studiorum, Università di Bologna, Via P. Gobetti 85, 40129 Bologna, Italy; ‡ Center for Chemical Catalysis, C3 Alma Mater Studiorum, Università di Bologna, Via P. Gobetti 85, 40129 Bologna, Italy; § Departamento de Química Orgánica, 16784 Universidade de Vigo, AS Lagoas (Marcosende) s/n, 36310 Vigo, Spain; ∥ Dipartimento di Chimica Industriale “Toso Montanari”, Alma Mater Studiorum, Università di Bologna, Via P. Gobetti 85, 40129 Bologna, Italy

## Abstract

A Pd-catalyzed electroreductive enantioselective dearomatization of tropones is documented. High levels of chemoselectivity toward the formation of seven-membered ring ketones featuring a stereochemically controlled all-carbon α-stereocenter are successfully accomplished by means of a Tsuji–Trost-like process (24 examples, regioselectivity >25:1, *enantiomeric ratio* of up to 96:4). Computational and electrochemical analyses allowed elucidation of the reaction manifold that involves initial reduction of the tropone motif and a subsequent chemo- and enantioselective allylation via an outer-sphere approach. In this way, a novel concept for the dearomatization of electron-poor scaffolds is disclosed by means of electrochemical reductive umpolung.

## Graphical abstract

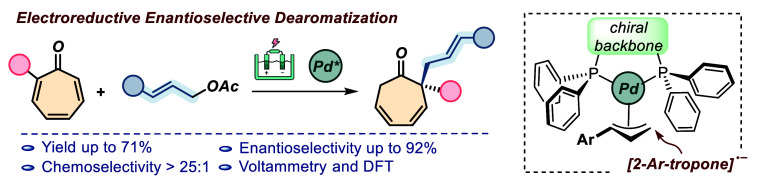

The growing interest in discovering new reaction manifolds that leverage electrochemistry can be attributed to the *in situ* reversal of the classical reactivity of a given compound through electron-transfer processes.^1^ Electrosynthesis is thus revolutionizing the horizons of synthetic organic chemistry, not only by transforming outdated and environmentally detrimental methodologies into sustainable alternatives,^2^ but also by enabling novel reactivity modes in which certain classes of compounds undergo transformations that would otherwise be inaccessible through conventional pathways. This advancement broadens the scope and overcomes the limitations of well-established and robust synthetic protocols.

Within this context, the field of enantioselective dearomatizations, which aims to construct complex, optically active three-dimensional architectures from planar two-dimensional chemical spaces, has now reached an exceptional level of maturity,^3^ and these processes serve as a key strategy underpinning the current “escape from flatland” paradigm in medicinal chemistry.^4^

However, the field of dearomatization reactions still poses a significant synthetic challenge, as it remains largely dominated by electron-rich arenes and heteroarenes (e.g., indoles, pyrroles, naphthols, phenols, etc.). These scaffolds typically offer favorable energetic profiles and enable mild and selective reaction conditions to be applied.^5^ In contrast, the involvement of electron-deficient arenes has been restricted to few isolated examples of dearomative transformations of specific pyridine derivatives,^6^ leaving substantial room for further development.

Here, we envisioned that the implementation of electrochemical methodologies could expand this chemical space by enabling electron-deficient species to participate in the target transformation, being turned into electron-rich intermediates through site-selective reductive electron-transfer processes.^7^ Remarkably, the use of electrochemistry in enantioselective dearomatizations remains unexplored to date.^8^

Our interest in discovering new electrochemical organic transformations^9^ led to the investigation of the still largely unexplored redox chemistry profiles of tropone/tropolone derivatives.^10^ In this direction, an example of *in situ* chemical umpolung (electrophile → nucleophile) was recently disclosed as a productive strategy to realize an electroreductive Ni-catalyzed alkylation of the aromatic core of tropone.^10c^

In line with these recent findings and the aforementioned working hypothesis, we herein present a new strategy toward a direct asymmetric electro-dearomatization of electron-poor arenes (i.e., tropones) through condensation with electrophilic allyl–metal species.^11^ This approach draws inspiration from the well-established α-allylation of ketones that is commonly carried out via pre-enolization under basic conditions (Figure 1, top).^12^ Alternatively, although less explored, the same process can involve the *in situ* formation of metal enolates, via conjugate hydride addition to electron-poor olefins (Figure 1, center).^13^ By recognizing the intrinsic structural analogy between α,β-unsaturated carbonyls and the tropone scaffold, we envisioned that the desired electrophile-to-nucleophile reactive inversion on the tropone core could be realized through electrochemical reduction of the seven-membered ring.

**Figure 1:**
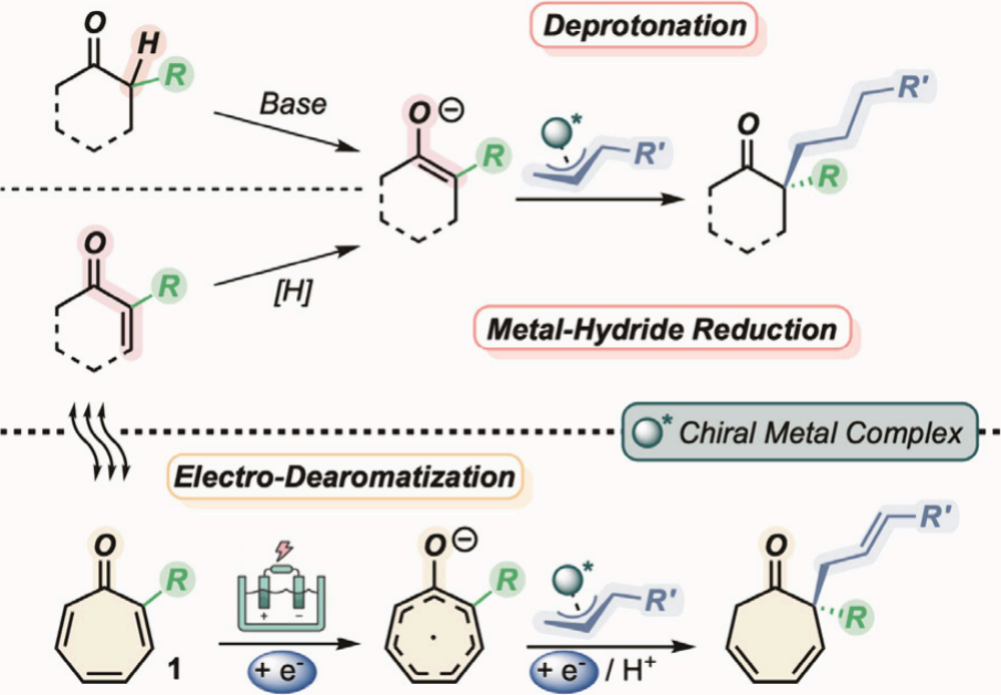
Introducing the concept of electro-dearomatization as reactivity umpolung of tropones. Previous modes of enolate generation toward enantioselective Tsuji–Trost allylations (top). Present working hypothesis addressing a metal-catalyzed electrochemical dearomative protocol (bottom).

The realized “enolate-like” radical ion might be conveniently engaged in an asymmetric allylation strategy (Tsuji–Trost-like process)^14^ to generate rapid access to densely functionalized cyclic scaffolds in enantiomerically enriched form. It is worth mentioning that our working plan would replace the use of strongly basic environments or harmful hydride sources with green electrons, enabling the reductive pathway.

Additionally, the adoption of the sacrificial anode strategy would preclude any oxidative rearomatization, leading to a fully reductive dearomatizing pathway (Figure 1, bottom). Furthermore, we foresee that the use of 2-substituted tropones **1** would potentially address the formation of synthetically challenging all-carbon quaternary stereogenic centers,^15^ in agreement with the enantioselective allylation of α-monosubstituted cyclic ketones.^16^

At the outset of our investigation, we tackled our working hypothesis by reacting 2-phenyltropone **1a** and cinnamyl acetate **2a** in the presence of [PdCl_2_(acn)_2_] and the Trost-type ligand **L1**. Importantly, the structure of **2a** was selected in order to challenge the linear and branched selectivity of our methodology. Electrochemical conditions involved the use of a sacrificial anode (Zn), a Ni cathode, and constant current electrolysis (10 mA, 1.5 h, TEABF_4_ electrolyte) in an ACN/MeOH (5:1) solvent mixture.^17^ Under these conditions, compound (*S*)-**3aa** was isolated in low yet promising yield (22%) and *enantiomeric ratio* (65:35 (Table 1, entry 1)). Remarkably, only the regioisomer featuring an all-carbon quaternary stereogenic center was detected in the reaction mixture, with the allyl moiety exclusively in the linear form. Other ligands, as well as Pd sources, proved to be far less efficient in terms of stereocontrol and isolated yield (entries 2–4 and Supporting Information). Changing the solvent from acetonitrile to THF led to an increase in the *enantiomeric ratio* to up to 78:22 (entry 5). The quantity of the protic source (i.e., MeOH) was also pivotal for the chemical outcome,^18^ and when the amount was decreased to 60:1 THF/MeOH, the yield and enantioselectivity were both improved (entry 6). Finally, with a decrease in the temperature to 0 °C, **3aa** was obtained in 85:15 *er* (entry 7).^19^

**Table 1:**
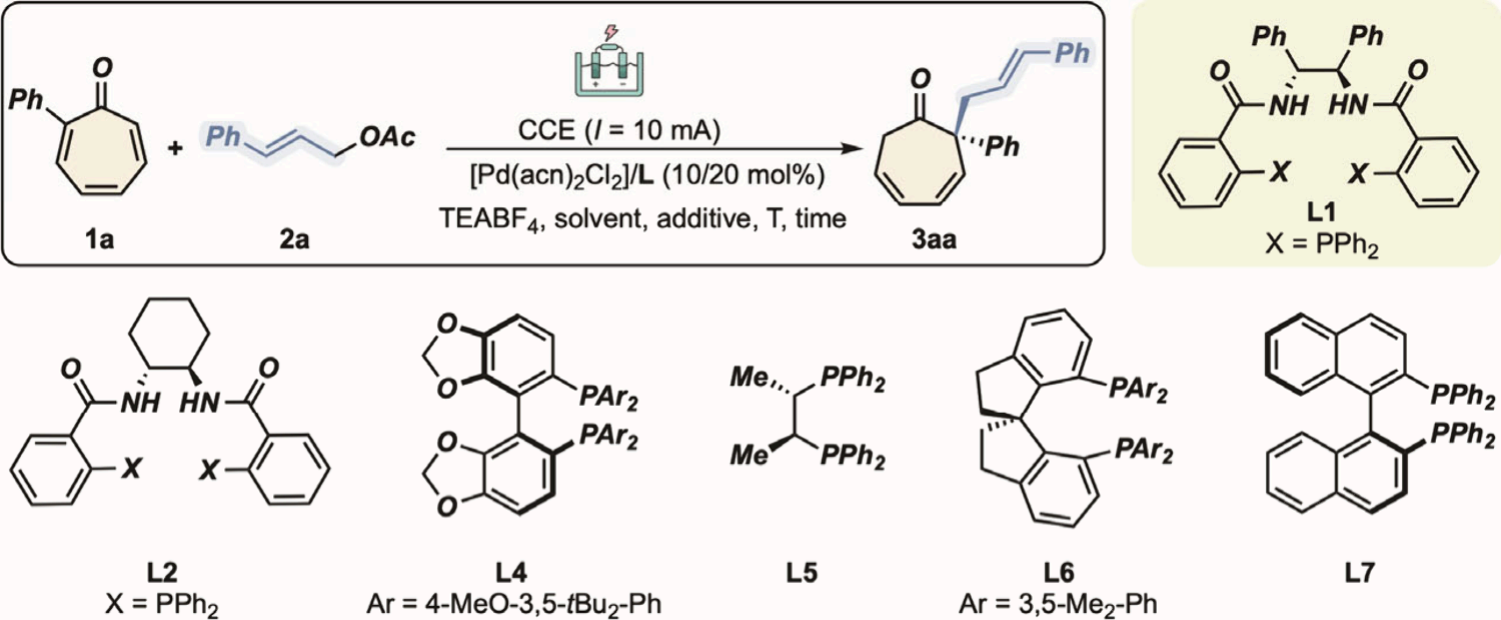
Optimization of the Reaction Conditions^a^

entry	**L**	solvent	electrode ((−)||(+))	additive	*T* (°C)	yield of **3aa** ^b^ (%)	*er* of **3aa** ^c^
1	**L1**	5:1 ACN/MeOH	Ni || Zn		25	22	65:35
2	**L2**	5:1 ACN/MeOH	Ni || Zn	–	25	36	55:45
3	**L3**	5:1 ACN/MeOH	Ni || Zn	–	25	22	55:45
4	**L4–7**	5:1 ACN/MeOH	Ni || Zn	–	25	NR	–
5^d^	**L1**	5:1 THF/MeOH	Ni || Zn	–	25	26	72:28
6^d^	**L1**	60:1 THF/MeOH	Ni || Zn	–	25	62	78:22
7^d^	**L1**	60:1 THF/MeOH	Ni || Zn	–	0	47	85:15
8^d^,^e^	**L1**	60:1 THF/MeOH	Ni || Zn	TBACl (1 equiv)	0	25	92:8
9^d^,^e^	**L1**	60:1 THF/MeOH	W || Zn	TBACl (1 equiv)	0	31	94:6
10^d^,^e^	**L1**	60:1 THF/MeOH	W || Mg	TBACl (1 equiv)	0	27	85:15
11^d^,^e^	**L1**	THF	W || Zn	H_2_O (12 equiv), TBACl (1 equiv)	0	62	94:6

a All reactions were carried in the Electrasyn 2.0 apparatus (3:1 **1a**:**2a**, 0.3 M **2a**, TEABF_4_ electrolyte).

b Isolated yields after flash chromatography.

c Determined by chiral HPLC analysis.

d The TBAPF_6_ electrolyte was used for better solubility.

e *I* = 5 mA; 3 h. NR: no reaction.

To further optimize the enantioselectivity of the process, the use of TBACl as an additive was tested, given the well-established positive role of halide ions in the enantioinduction of Tsuji–Trost allylations.^20^ Remarkably, although a decrease in efficiency (25% yield) was recorded, **3aa** was isolated in a 92:8 *enantiomeric ratio* (entry 8). These conditions prompted us to undertake an intense survey of different electrode couples, resulting in a slight improvement in the isolated yield and *enantiomeric ratio* upon replacement of Ni with W as the cathode (31%; see also the Supporting Information). Finally, upon a final screening of protic sources (see the Supporting Information and *vide infra* for mechanistic elucidations), we discovered water to be the most efficient, allowing **3aa** to be isolated in 62% yield and 94:6 *er* (entry 11).^21^

Thereafter, the scope of the reaction was initially investigated by testing a series of cinnamyl acetates (**2b**–**n**), and the results are listed in Scheme 1. The introduction of halogen atoms at the *para* and *ortho* positions of the cinnamyl aryl unit (**2b**–**e**) led to constantly high *enantiomeric ratios* (up to 93:7, **3ae**) and moderate isolated yields. Even higher levels of enantiocontrol were achieved by employing allylating agents based on EDG-substituted acetates (**2f**–**i**). Here, an *enantiomeric ratio* as high as 96:4 was recorded with the precursor carrying a *m*MeO-substituted arene, together with a synthetically useful 63% isolated yield (**3af**). The compatibility of the protocol toward different allylating units was also ascertained with the exploration of extended as well as heteroarene derivatives (**2j**–**m**). Satisfyingly, moderate to high *enantiomeric ratios* were always accompanied by reasonably high isolated yields (up to 51%, **3am**). However, when simple allyl acetate **2n** was employed, cycloheptadienone **3an** was isolated with low optical purity, although in a good yield.

**Scheme 1:**
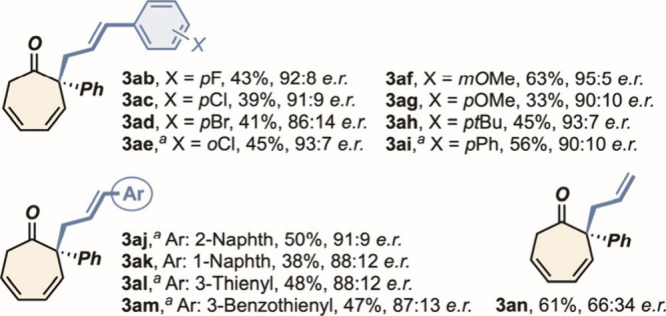
Testing Different Cinnamyl Acetates **2** (reaction conditions from entry 11 of Table 1

a At a 6:1 **1a**:**2** ratio with a reaction time of 6 h.

Then, our attention turned to assessing the generality of the protocol toward the decoration of the tropone scaffold. In this direction, a series of α-substituted tropones were synthesized via Suzuki–Miyaura cross-coupling (**1b**–**k**).

Remarkably, the regioselectivity toward the targeted quaternary stereogenic center was always recorded, with no trace of the dearomatized C(7)-allylated isomer (Scheme 1, top). Here, a wide range of functional groups could be accommodated at the tropone moiety, such as halides (**1d**), esters (**1b**), ketones (**1c**), alkyl and aryl groups (**1f** and **1g**), silyl moieties (**1e**), and extended π-systems (**1h**). In this context, isolated yields and *enantiomeric ratios* reached 70% and 96:4, respectively, in the case of compound **3ef**. Finally, isomeric 4- and 6-isopropyl-2-phenyl-tropones **1j** and **1k**, which could be divergently obtained from the same chemical source (i.e., β-thujapicin), were subjected to the present Pd-catalyzed enantioselective reductive dearomatization, leading to moderate enantiomeric ratios and good yields.

Finally, to access the versatility of products **3** toward chemical manipulation, we tackled two chemoselective reductions of **3aa** (Scheme 2, bottom). First, transformation of the ketone moiety (NaBH_4_) toward alcohol **4** was realized in excellent yield (95%), moderate diastereoselectivity (2.7:1), and high retention of the starting *enantiomeric ratio*. Alternatively, the three double bonds of **3aa** could be hydrogenated to fully saturated product **5**, retaining the carbonyl function, in excellent yield (92%), with good retention of enantioselectivity.

**Scheme 2:**
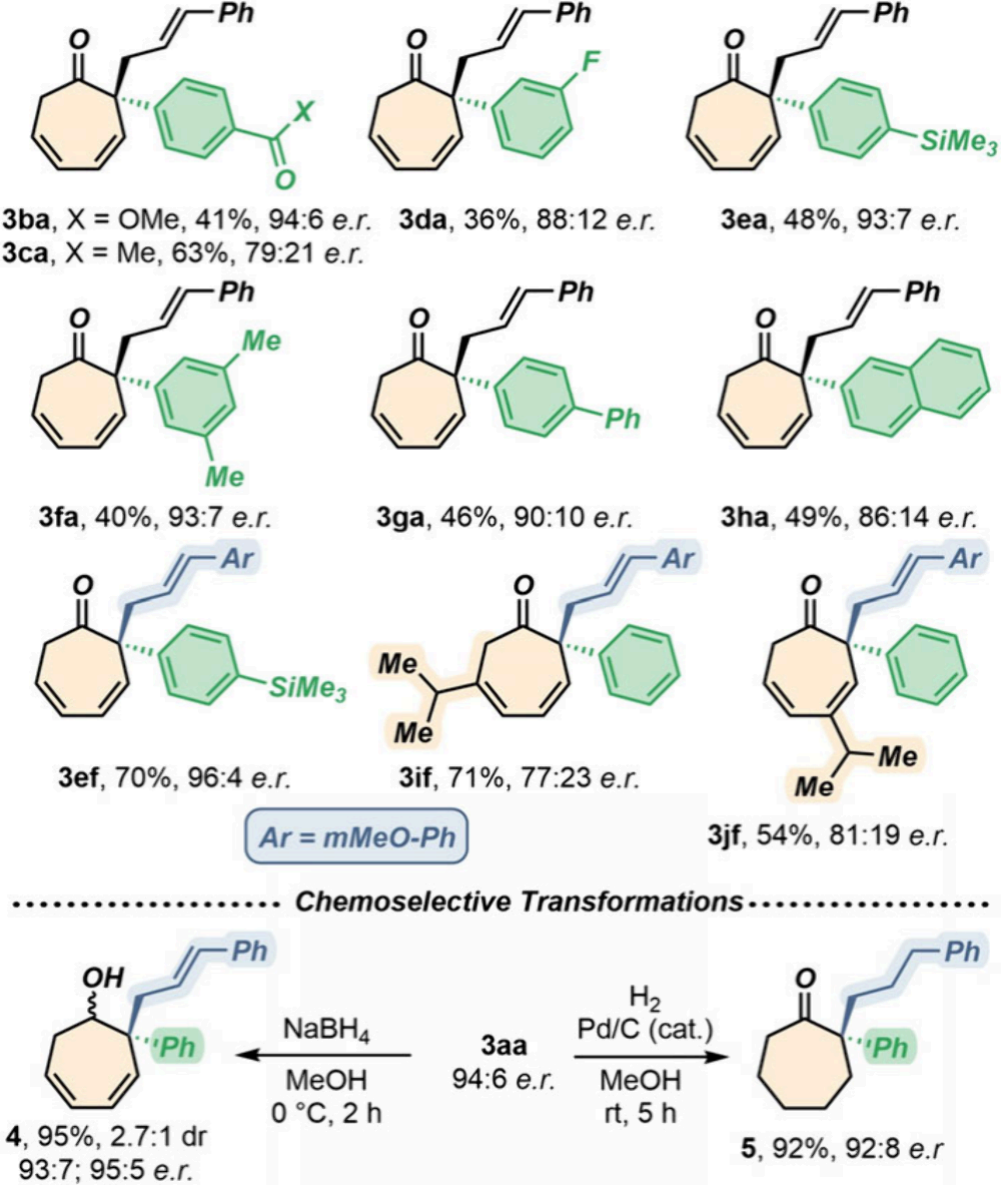
Testing Different 2-Aryltropones **1** (top, reaction conditions from entry 11 of Table 1) and Chemoselective Reduction of Dearomatized Cycloheptadienones **3aa** (bottom)

In order to gain a deeper mechanistic understanding of the studied reaction, we employed DFT calculations at the r^2^SCAN/def2-SV­(P)/THF­(CPCM) level,^22^ using THF as the solvent (see the Supporting Information for further details). The computational study is based on the results obtained from a voltametric investigation carried out on 2-phenyl-tropone **1a** and the preformed [Pd­(PPh_3_)_2_(η^3^-allyl)]­BF_4_ complex (Figure S8).^23^ These clearly indicate that reduction of **1a** occurs preferentially (−1.80 V vs Fc/Fc^+^) with respect to the Pd complex (−2.42 V vs Fc/Fc^+^), suggesting an unlikely formation of low-valent allyl–Pd intermediates and pointing toward the electrochemical generation of nucleophilic species from the reduction of **1a**. We therefore propose an initial reduction of precomplex [(**L1**)­Pd­(II)­Cl_2_] to catalytically active [(**L1**)­Pd­(0)] that can add to allyl acetate **2n** to give [Pd­(**L1**)­(η^3^-allyl)] complex **B**.^24^ At the same time, **1a** is reduced at the cathode to generate radical anion **A**. We then performed the mechanistic study by considering two alternative pathways featuring species **A** as a common initial precursor (Scheme 3).

**Scheme 3:**
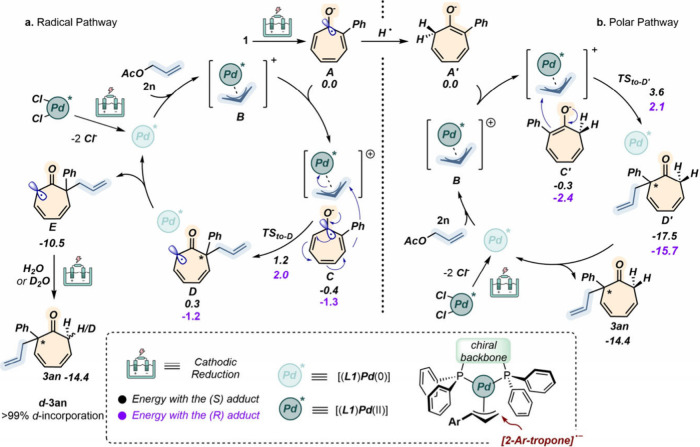
Proposed Mechanistic Pathways for the Pd-Catalyzed Electrochemical Dearomative Allylation of Tropone **1** ^a^

a The reported energies correspond to Δ*G* values in kilocalories per mole.

In particular, pathway a) relies on a direct electro-dearomatization step directly involving radical anion **A** and its condensation with *in situ*-formed [Pd­(**L1**)­(η^3^-allyl)] complex **B**. Here, weakly stabilized precomplex **C** is formed where the allyl unit approaches the carbon *ipso* to the Ph group. Both diastereomeric transition states were identified and compared from an energetic viewpoint. Interestingly, the (*S*)-TS (**C** → **D**) exhibits an activation energy (1.6 kcal/mol) lower than that of the one leading to the *R* enantiomer (3.3 kcal/mol). This finding matches the determination of the absolute configuration of the major isomer (see the Supporting Information for details) and aligns closely with the experimental observation that, with a decrease in the temperature to 0 °C, enhanced enantioselectivity was achieved (entry 6 vs entry 7, Table 1). Next, the loosely bound reduced Pd complex (**D**) dissociates leading to thermodynamically stable radical **E**. The latter was then easily quenched through reduction and protonation to yield product **3an**. The active role played by water in the final proton quenching was proved by running the model process in the presence of D_2_O as the additive. Here complete monodeuteration was detected at C-7 of the cycloheptadienyl ring.

In pathway b), we first assumed that radical anion **A** could be quenched through a HAT event, yielding **A′**, which then reacts with [Pd­(**L1**)­(η^3^-allyl)] **B** leading to precomplex **C′**. The following rate-determining reductive elimination takes place with activation energies of 3.9 and 4.4 kcal/mol for the *S* and *R* enantiomers, respectively. Resulting intermediate **D′** was quite stable for both isomers, and the subsequent release of the catalyst leading to final product **3an** was found to be uphill by 2–4 kcal/mol. Both the kinetics of the rate-determining step and the thermodynamics of catalyst release suggest that radical pathway a is most likely the mechanistic route followed by the present transformation. It is worth noting that this allylation occurs through an outer-sphere approach. In fact, all attempts to compute reaction machineries involving coordination of the Pd catalyst to the tropone core before C–C bond formation led to remarkably higher (10 kcal/mol) energy barriers for the transition states (see Figure S9). Finally, we were able to rationalize the stereoinduction experimentally recorded (*S* enantiomer as the major one) that correlated to the key interactions between the aromatic rings of the 2-(diphenylphosphino) benzoic acid amide scaffold of **L1** and the phenyl substituent of the tropone (see Figure S10).

In conclusion, a new strategy for the electroreductive enantioselective dearomatization of 2-aryltropones via a palladium-catalyzed nucleophilic allylic alkylation is presented. The protocol employs chiral *C*_2_-symmetric Trost-type palladium complexes, enabling rapid access to enantiomerically enriched seven-membered ring ketones bearing all-carbon quaternary stereogenic centers. The proposed mechanistic rationale (electrochemical enolization of tropone followed by outer-sphere-type allylation) is supported by both experimental (voltametric) and computational (DFT) studies.

## Supplementary Material



## Data Availability

The data underlying this study are available in the published article and in its Supporting Information.

## Notes

Experimental procedures, characterization details (copies of NMR spectra and HPLC chromatogram), mechanistic studies, and additional computational details (PDF)
