# Supplementary material for: Enantioselective Pd-Catalyzed Electrochemical Dearomative Allylation of Tropones: Construction of All‑C Quaternary Stereocenters
Source: Org Lett. 2026 Mar 18;28(13):4227–33. doi: 10.1021/acs.orglett.6c00796 (PMC13054876; doi:10.1021/acs.orglett.6c00796)
Supplement: Supplementary file 1 [file ol6c00796_si_001.pdf]

Enantioselective Pd-catalyzed Electrochemical Dearomative Allylation of Tropones: Construction of all-C Quaternary Stereocenters

Giulia Monda,[a,b] Sofia Kiriakidi,[a,b,c] Olalla Nieto Faza,*[c] Andrea Mazzanti,[b,d] Giulio Bertuzzi,* [a,b] and Marco Bandini* [a,b]

[a] Ms. Giulia Monda, Dr. S. Kiriakidi, Dr. G. Bertuzzi, Prof. M. Bandini, Dipartimento di Chimica "Giacomo Ciamician", Alma Mater Studiorum – Università di Bologna, Via P. Gobetti 85, 40129 Bologna, Italy.

[b] Ms. G. Monda, Prof. A. Mazzanti Dr. G. Bertuzzi, Prof. M. Bandini, Center for Chemical Catalysis – C^3 Alma Mater Studiorum – Università di Bologna, via P. Gobetti 85, 40129 Bologna, Italy.

[c] Dr. S. Kiriakidi, Prof. O. Nieto Faza, Departamento de Química Orgánica, Universidade de Vigo, AS Lagoas (Marcosende) s/n, 36310 Vigo, Spain.

[d] Prof. A. Mazzanti, Dipartimento di Chimica Industriale "Toso Montanari", Alma Mater Studiorum – Università di Bologna, Via P. Gobetti 85, 40129 Bologna, Italy.

E-mail: faza@uvigo.gal, giulio.bertuzzi2@unibo.it, marco.bandini@unibo.it.

Table of contents

1. General Methods	S3
2. Preparation of Starting Materials	S4
3. Additional Optimization Data	SS7
4. Preparation and Characterization of Compounds **3**	S12
5. Transformations of Product **3aa**	S28
6. Determination of the Absolute Configuration	S31
7. Voltametric Analysis	S38
8. Deuteration Experiment	S40
9. Additional Computational Details	S41
10. NMR spectra	S43
11. Chiral Stationary Phase HPLC Traces	S74
12. Cartesian coordinates	S101
13. References	S146

1. General Methods

^{1}H-NMR spectra were recorded on a Bruker 600 spectrometer (600 MHz). Chemical shifts are reported in ppm from TMS with the solvent resonance as the internal standard. Data are reported as follows: chemical shift, multiplicity (s = singlet, d = doublet, dd= double doublet, t = triplet, td = triple doublet, dt = double triplet, q = quartet, b = broad, m = multiplet), coupling constants (Hz). ^{13}C-NMR spectra were recorded on a Bruker 600 spectrometer (150 MHz) with complete proton decoupling. Chemical shifts are reported in ppm from TMS with the solvent as the internal standard.

HRMS spectra were obtained with a G2XS QTof mass spectrometer using ESI ionization techniques, as specified case by case.

Chromatographic purification was done with 240-400 mesh silica gel.

Anhydrous solvents, including THF for the catalytic processes, were supplied by Merck in Sureseal® bottles and used without any further purification.

Computational Methods

Density Functional Theory (DFT) as implemented in ORCA 6.0[1] was used for the quantum chemistry calculations of this study, employing the r^{2}SCAN/def2-SV(P)[2] level of theory. The rationale behind the choice of method was to combine the accuracy of a meta-GGA functional with computational efficiency for the treatment of the large catalyst molecules. Solvent effects were modelled using the CPCM[3] with THF as the solvent. The nature of all optimized structures was determined using frequency analysis to correctly identify them as minima or transition states.

2. Preparation of Starting Materials

2.1 Reported compounds

Compounds **1a-1h** are known compounds and were prepared according to our recently disclosed modification of a previously reported procedure.[4]

All acetates **2** are known compounds and were prepared according to unmodified literature procedures.[5]

2.2 Preparation and Characterization of **1i** and **1j.**

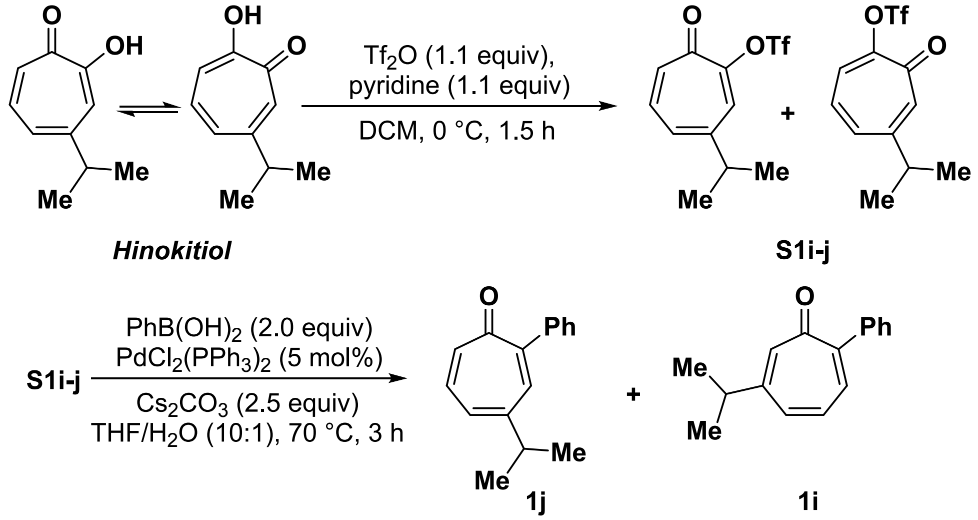

Hinokitiol triflate **S1i-j**, prepared according to our modification of the procedure reported by Jørgensen *et al.*,[6] was subjected to Suzuki coupling (1 mmol of **S1i-j**) with phenylboronic acid[4] to yield a mixture of products **1i** and **1j**. Separation of these compounds was achieved through FC on silica gel (*n*Hex/EtOAc 4:1) affording **1i** as the first eluting fraction and **1j** as the second one. The structure of **1j** was assigned through 1D-NOE NMR experiments by irradiation of the only signal in the aromatic region lacking a ^3J coupling constant (7.31 ppm). Correlation between this hydrogen and Ha (7.51 – 7.45 ppm) indicate that the signal at 7.31 ppm belongs to H-3 and that the iso-propyl group occupies position C-4 of the tropone (Figure S1). Contrarily, irradiation of the only signal in the aromatic region lacking a ^3J coupling constant of **1i** (7.10 ppm) does not exhibit the same correlation, indicating that this signal belongs to H-7 and that the isopropyl group occupies position C-6 of the tropone (Scheme S2).

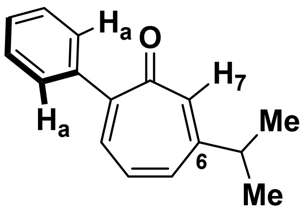

1i. Pale yellow solid. Yield = 39%, (0.39 mmol, 87.4 mg). **^{1}H NMR** (600 MHz, $CDCl_3$) δ = 7.51 – 7.46 (m, 2H), 7.41 – 7.38 (m, 2H), 7.37 – 7.34 (m, 1H), 7.26 (dd, *J* = 8.7, 0.9 Hz, 1H), 7.10 (d, *J* = 1.7 Hz, 1H), 6.97 (ddd, *J* = 11.4, 8.7, 0.7 Hz, 1H), 6.89 (ddd, *J* = 11.4, 1.9, 1.0 Hz, 1H), 2.78 (hept, *J* = 6.9 Hz, 1H), 1.26 (d, *J* = 6.9 Hz, 6H); **^{13}C NMR** (151 MHz, $CDCl_3$) δ = 186.4, 155.7, 152.1, 140.1, 138.9, 135.7, 134.9, 132.4, 129.1 (2C), 128.3, 128.1 (2C), 37.9, 22.8; **HRMS (ESI)** m/z: $[M + Na]^+$ calcd. for $C_{16}H_{16}NaO^+$: 247.1093; found 247.1095.

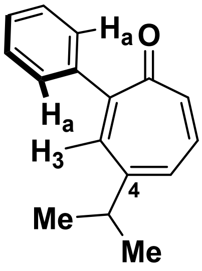

1j. Pale yellow oil. Yield = 43%, (0.43 mmol, 97.5 mg). **^{1}H NMR** (600 MHz, $CDCl_3$) δ = 7.51 – 7.45 (m, 2H), 7.43 – 7.40 (m, 2H), 7.39 – 7.36 (m, 1H), 7.31 (d, *J* = 1.7 Hz, 1H), 7.13 – 7.06 (m, 2H), 6.86 – 6.83 (m, 1H), 2.81 (hept, *J* = 6.9 Hz, 1H), 1.25 (d, *J* = 6.9 Hz, 6H); **^{13}C NMR** (151 MHz, $CDCl_3$) = 186.1, 154.7, 151.9, 140.8, 140.1, 138.5, 135.9, 129.2 (2C), 128.9, 128.3, 128.1 (2C), 38.4, 23.1; **HRMS (ESI)** m/z: $[M + Na]^+$ calcd. for $C_{16}H_{16}NaO^+$: 247.1093; found 247.1089.

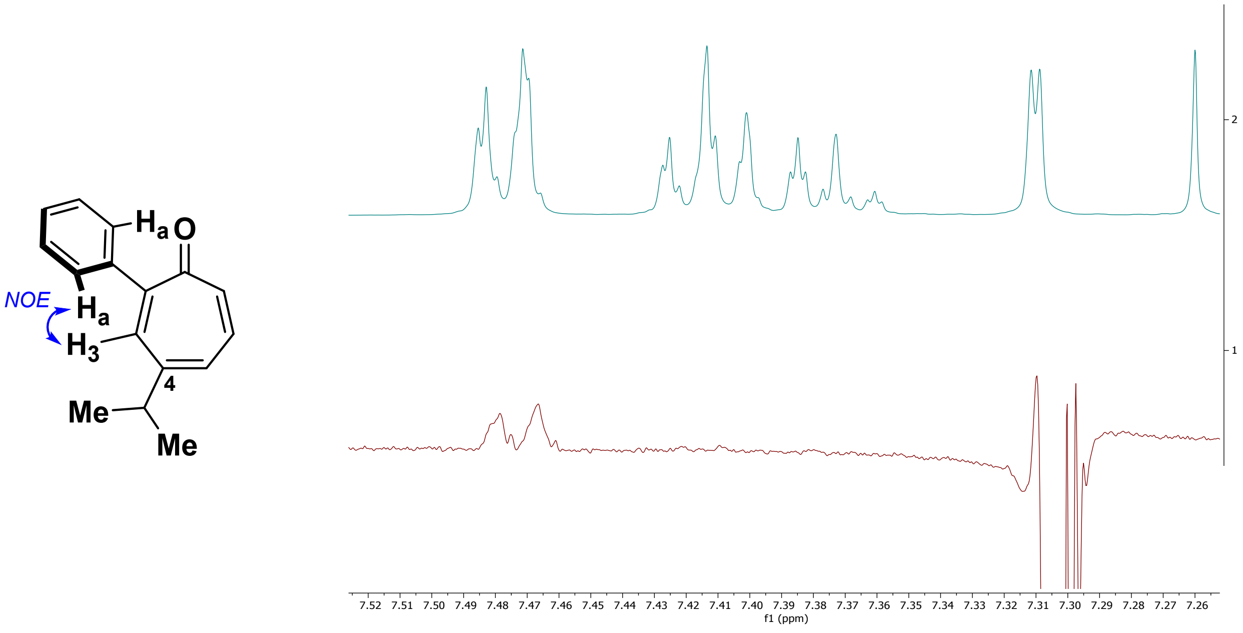

Figure S1. 1D-NOE NMR Spectrum of **1j** (relevant region)

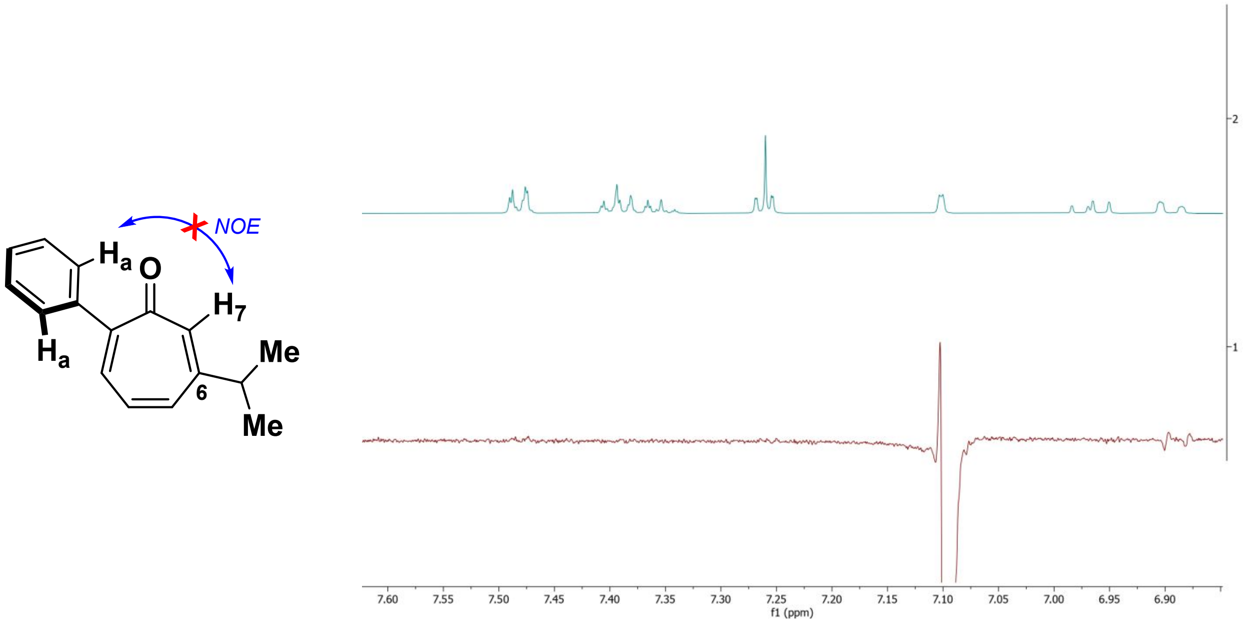

Figure S2. 1D-NOE NMR Spectrum of **1i** (relevant region)

3. Additional Optimization Results

3.1 **Table S1**: Ligands.

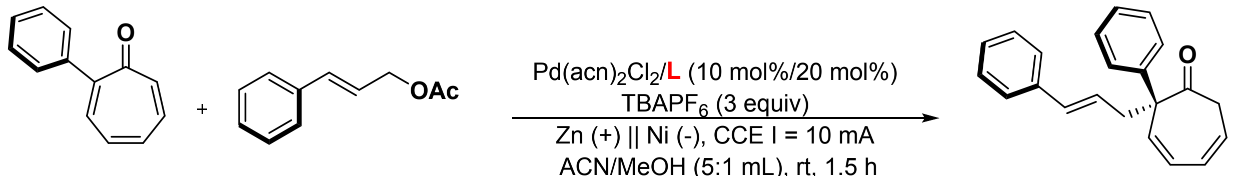

1a **2a** **3aa**

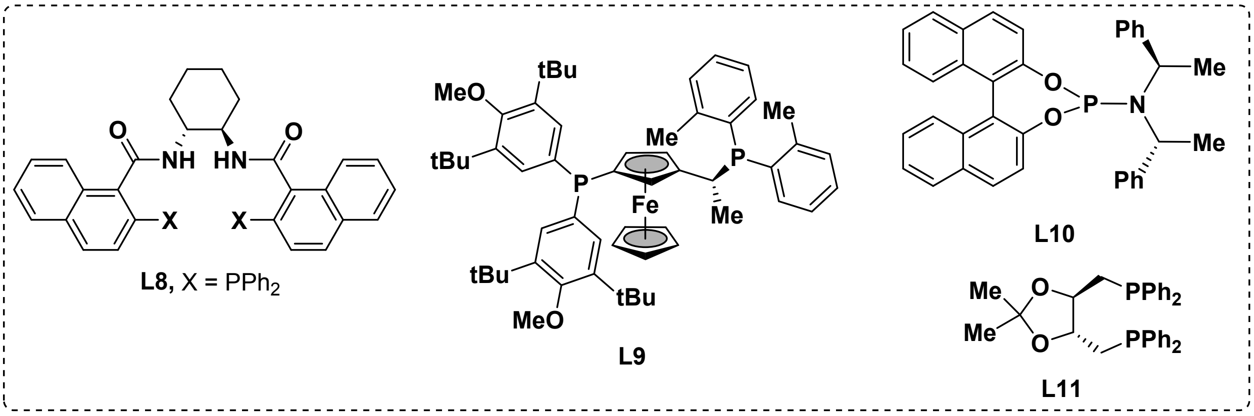

Entry[a]	Ligand	Yield [%][b]	e.r.[c]
1	**L8**	33	55:45
2	**L9**	-	
3	**L10**	18	*rac*
4	**L11**	-	
**5**	**L1**	**22**	**65:35**

[a] Reaction conditions: **1a** (0.3 mmol, 3 equiv), **2a** (0.1 mmol, 1 equiv), $TBAPF_6$ (0.3 mmol), $Pd(can)_2Cl_2$ (0.010 mmol, 10 mol%), Ligand (0.020 mmol, 20 mol%), ACN (2.5 mL), MeOH (0.5 mL), Zn(+) || Ni(-), CCE (I = 10 mA), rt, 1.5 h. [b] Isolated yield after FC on silica gel. [c] Determined by CSF HPLC.

3.2 **Table S2**: Solvents, Protic Sources and Temperature.

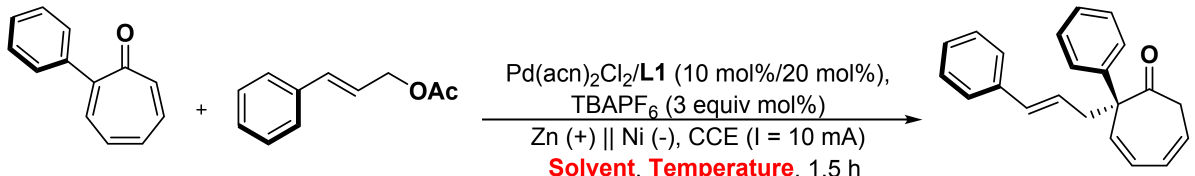

1a **2a** **3aa**

Entry[a]	Solvent	Protic Source	Ratio	Temperature	Yield [%][b]	e.r.[c]
1	THF	MeOH	5:1	rt	26	72:28
2	THF	MeOH	50:1	rt	62	78:22
3	THF	iPrOH	50:1	rt	29	77:23
4	THF	none	50:1	rt	-	-
**5[d]**	**THF**	**MeOH**	**50:1**	**0 °C**	**47**	**85:15**
6[d]	THF	MeOH	50:1	-20 °C	25	80:20
7[d]	1,4-Dioxane	MeOH	50:1	0 °C	-	-
8[d]	2-MeTHF	MeOH	50:1	0 °C	-	-
9[d]	$PhCF_3$	MeOH	50:1	0 °C	25	*rac*
10[d]	PhH/THF	MeOH	50:1	0 °C	38	72:28
11[d]	THF	tBuOH	50:1	0 °C	10	64:36
12[d]	THF	TFE	50:1	0 °C	-	-
13[d]	THF	HFIP	50:1	0 °C	-	-
14[d]	THF	PivOH	50:1	0 °C	-	-

[a] Reaction conditions: **1a** (0.3 mmol, 3 equiv), **2a** (0.1 mmol, 1 equiv), $TBAPF_6$ (0.3 mmol), $Pd(ACN)_2Cl_2$ (0.010 mmol, 10 mol%), **L1** (0.020 mmol, 20 mol%), Solvent/Protic Source (total 3 mL, ratio indicated case by case), Zn(+) || Ni(-), CCE (I = 10 mA), rt, 1.5 h. [b] Isolated yield after FC on silica gel. [c] Determined by CSF HPLC. [d] CCE (I = 5 mA), 3 h.

3.3 **Table S3**: Leaving Groups.

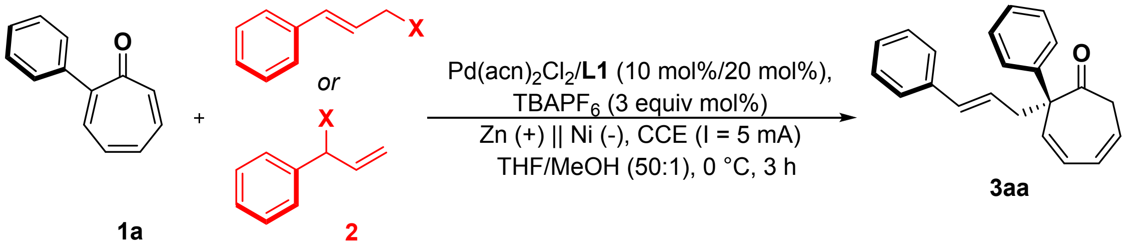

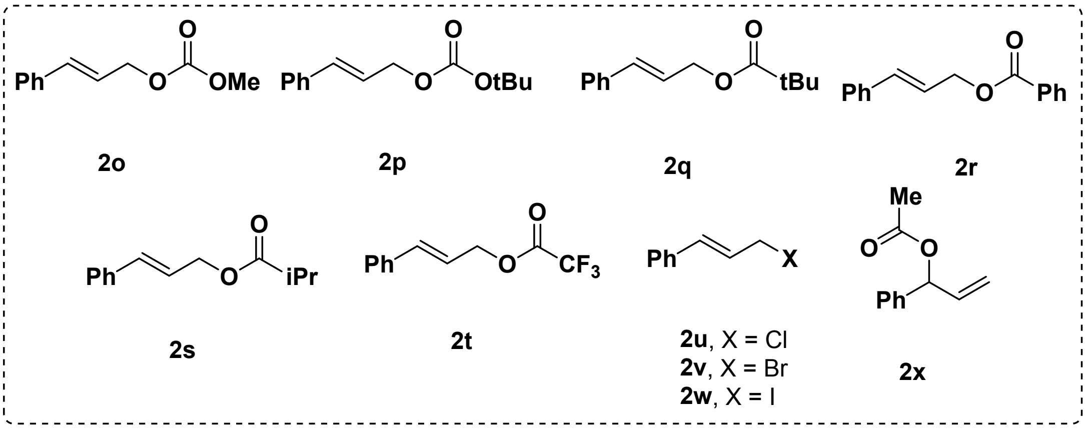

Entry[a]	**2**	Yield [%][b]	e.r.[c]
1	**2o**	-	
2	**2p**	-	
3	**2q**	20	85:15
4	**2r**	10	65:35
5	**2s**	-	
6	**2t**	-	
7	**2u**	-	
8	**2v**	-	
9	**2w**	-	
10	**2x**	15	87:13
**11**	**2a**	**47**	**85:15**

[a] Reaction conditions: **1a** (0.3 mmol, 3 equiv), **2** (0.1 mmol, 1 equiv), $TBAPF_6$ (0.3 mmol), $Pd(can)_2Cl_2$ (0.010 mmol, 10 mol%), **L1** (0.020 mmol, 20 mol%), THF/MeOH (50:1, total 3 mL), Zn(+) || Ni(-), CCE (I = 5 mA), 0 °C, 3 h. [b] Isolated yield after FC on silica gel. [c] Determined by CSF HPLC.

3.4 **Table S4**: Pd-source.

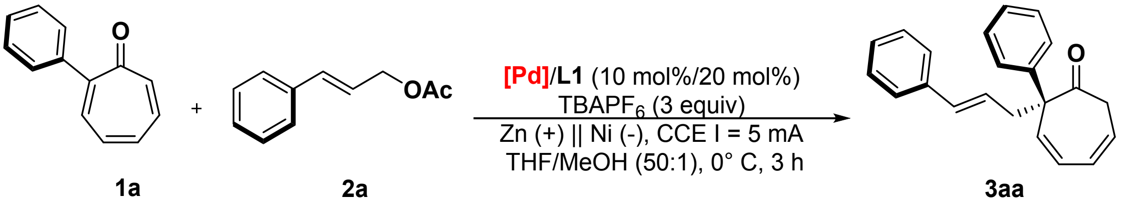

Entry[a]	[Pd]	Yield [%][b]	e.r.[c]
1	$PdCl_2$	60	58:42
2	$[Pd(allyl)Cl]_2$	-	-
3	$Pd(dba)_2$	-	-
4	$Pd(cod)Cl_2$	40	86:14
**5**	**$Pd(acn)Cl_2$**	**47**	**85:15**

[a] Reaction conditions: **1a** (0.3 mmol, 3 equiv), **2** (0.1 mmol, 1 equiv), $TBAPF_6$ (0.3 mmol), [Pd] (0.010 mmol or 0.05 mmol for entry 2, 10 mol% in total Pd), **L1** (0.020 mmol, 20 mol%), THF/MeOH (50:1, total 3 mL), Zn(+) || Ni(-), CCE (**I** = 5 mA), 0 °C, 3 h. [b] Isolated yield after FC on silica gel. [c] Determined by CSF HPLC.

3.5 **Table S5**: Halogenide Additives.

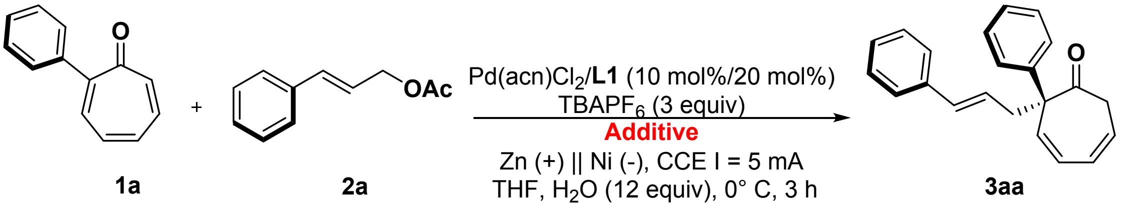

Entry[a]	Additive	Yield [%][b]	e.r.[c]
1	TBABr	15	92:8
2	TBAI	18	91:9
**3**	**TBACl**	**62**	**94:6**

[a] Reaction conditions: **1a** (0.3 mmol, 3 equiv), **2** (0.1 mmol, 1 equiv), $TBAPF_6$ (0.3 mmol), $Pd(acn)Cl_2$ (0.010 mmol, 10 mol%), **L1** (0.020 mmol, 20 mol%), H_2O (12 equiv), Additive (1 equiv), THF (3 mL), Zn(+) || Ni(-), CCE (**I** = 5 mA), 0 °C, 3 h. [b] Isolated yield after FC on silica gel. [c] Determined by CSF HPLC.

3.6 Unsuccessful Substrates

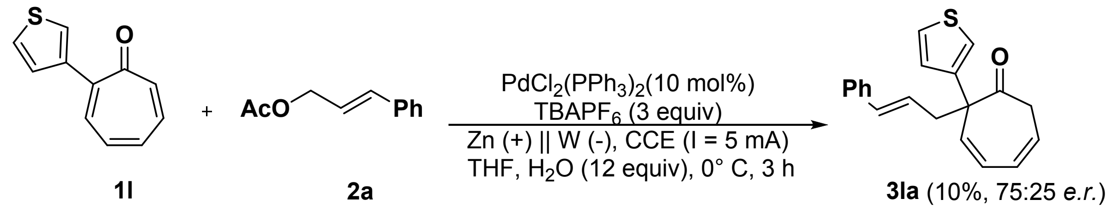

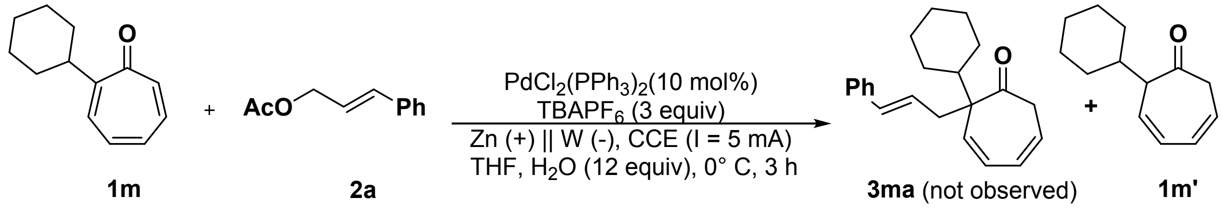

Hetero-aromatic substituted tropone **1l** delivered the corresponding product **3la** in 10% yield and 75:25 *e.r.* 2-Cyclohexyltropone **1m** did not undergo the desired process, while delivering reduced product **1m'** as the only reaction outcome.

4. Preparation and characterization of compounds 3.

4.1 General Procedures

General procedure **A** for the preparation of racemic products ***rac*-3**:

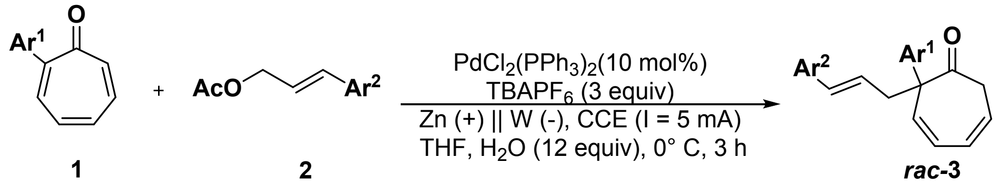

The ElectraSyn vial (5 mL), equipped with a stir bar, was charged with the appropriate tropone derivative **1** (0.30 mmol, 3.0 equiv.), $PdCl_2(PPh_3)_2$ (0.01 mmol, 10 mol%, 7 mg), $TBAPF_6$ (0.30 mmol, 116 mg) and the appropriate cinnamyl acetate **2** (0.1 mmol, 1 equiv). The ElectraSyn vial cap, equipped with anode (Zn) and cathode (W), was inserted into the mixture and closed with a rubber septum. The vessel was evacuated and backfilled with Ar three times, then dry THF (3.0 mL) was added, and the mixture stirred until complete dissolution of the solids occurred, while bubbling with Ar (balloon, 30 s). Then H_2O (12 equiv, 22 μL) was added via a Hamilton syringe. The reaction mixture was electrolysed (under Ar, balloon) at a constant current of 5.0 mA, for 3 hours at room temperature (0.56 mF, 5.6 F/mol**2**). Then, the ElectraSyn vial cap was removed, and the electrodes and vial were rinsed with EtOAc (10 mL) and $NH_4Cl_{(aq)}$ (saturated, 5 mL) and water (5 mL), which were combined with the crude mixture in a separatory funnel. Then, the organic layer was separated, and the aqueous layer was extracted with EtOAc (2 x 10 mL). The combined organic layers were washed with $NH_4Cl_{(aq)}$ (0.1 M, 3 x 10 mL), dried over Na_2SO_4 and concentrated in vacuo. The crude product was finally purified by FC on silica gel to afford pure products ***rac*-3**.

General procedure **B** for the preparation of enantioenriched products **3**:

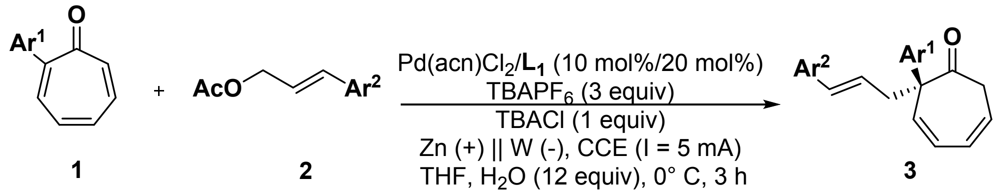

In a Schlenk tube, under an Ar atmosphere and magnetic stirring, $Pd(acn)_2Cl_2$ (0.01 mmol, 10 mol%, 2.6 mg), and ligand **L1** (0.02 mmol, 20 mol%, 16.5 mg) were stirred in THF (1 mL) at room temperature for 1 h. Then, the ElectraSyn vial (5 mL), equipped with a stir bar, was charged with the appropriate tropone derivative **1** (0.30 mmol, 3.0 equiv.), $TBAPF_6$ (0.30 mmol, 77.3 mg), TBACl (0.1 mmol, 27.8 mg) and the appropriate cinnamyl acetate **2** (0.1 mmol, 1 equiv). The ElectraSyn vial cap, equipped with anode (Zn) and cathode (W), was

inserted into the mixture and closed with a rubber septum. The vessel was evacuated and backfilled with Ar three times, then dry THF (2.0 mL) and the previously formed Pd-complex (in 1 ml of THF) was added, and the mixture stirred until complete dissolution of the solids occurred, while bubbling with Ar (balloon, 30 s). Then H_2O (12 equiv, 22 μL) was added via a Hamilton syringe. The reaction mixture was electrolysed (under Ar, balloon) at a constant current of 5.0 mA, for 3 hours at 0°C (0.56 mF, 5.6 F/mol**2**). Then, the ElectraSyn vial cap was removed, and the electrodes and vial were rinsed with EtOAc (10 mL) and $NH_4Cl_{(aq)}$ (saturated, 5 mL) and water (5 mL), which were combined with the crude mixture in a separatory funnel. Then, the organic layer was separated, and the aqueous layer was extracted with EtOAc (2 x 10 mL). The combined organic layers were washed with $NH_4Cl_{(aq)}$ (0.1 M, 3 x 10 mL), dried over Na_2SO_4 and concentrated in vacuo. The crude product was finally purified by FC on silica gel to afford pure products **3**.

*<u>Note A</u>: The reactions leading to compounds **3ae**, **3ai**, **3aj** and **3al** were performed with 6 equiv of 2-phenyltropone **1a** (0.60 mmol, 109.2 mg), for 6 h (1.12 mF, 11.2 F/mol**2**).*

<u>Note B</u>: The Electrasyn 2.0 electrochemical workstation and all the electrodes for the preparative electrolyses were purchased from IKA. All the electrodes have the shape of a rectangular parallelepiped with 5.3 cm x 0.8 cm x 0.2 cm dimensions resulting in a wet surface area of 1.2 cm^2 in the case of the 0.1 mmol scale reaction and 2.8 cm^2 for the 1.0 mmol scale reaction. The distance between the electrodes in this set-up is fixed and equal to 0.5 cm.

<u>Note C</u>: The reaction outcome was not found to be particularly sensitive to stirring. A fixed stirring rate of 1000 rpm was adopted in all cases.

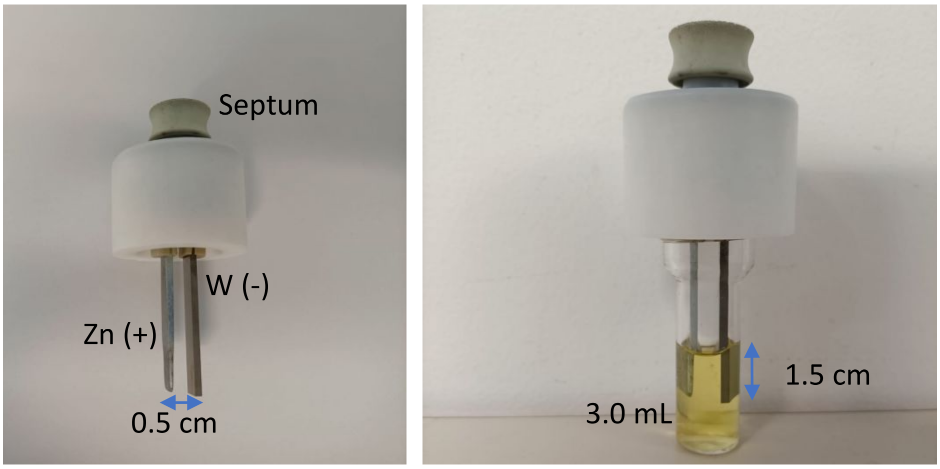

Figure S3. Electrasyn vial cap equipped with Zn anode and W cathode (left). Electrasyn vial (equipped with electrodes and cap) filled with the reaction mixture (right).

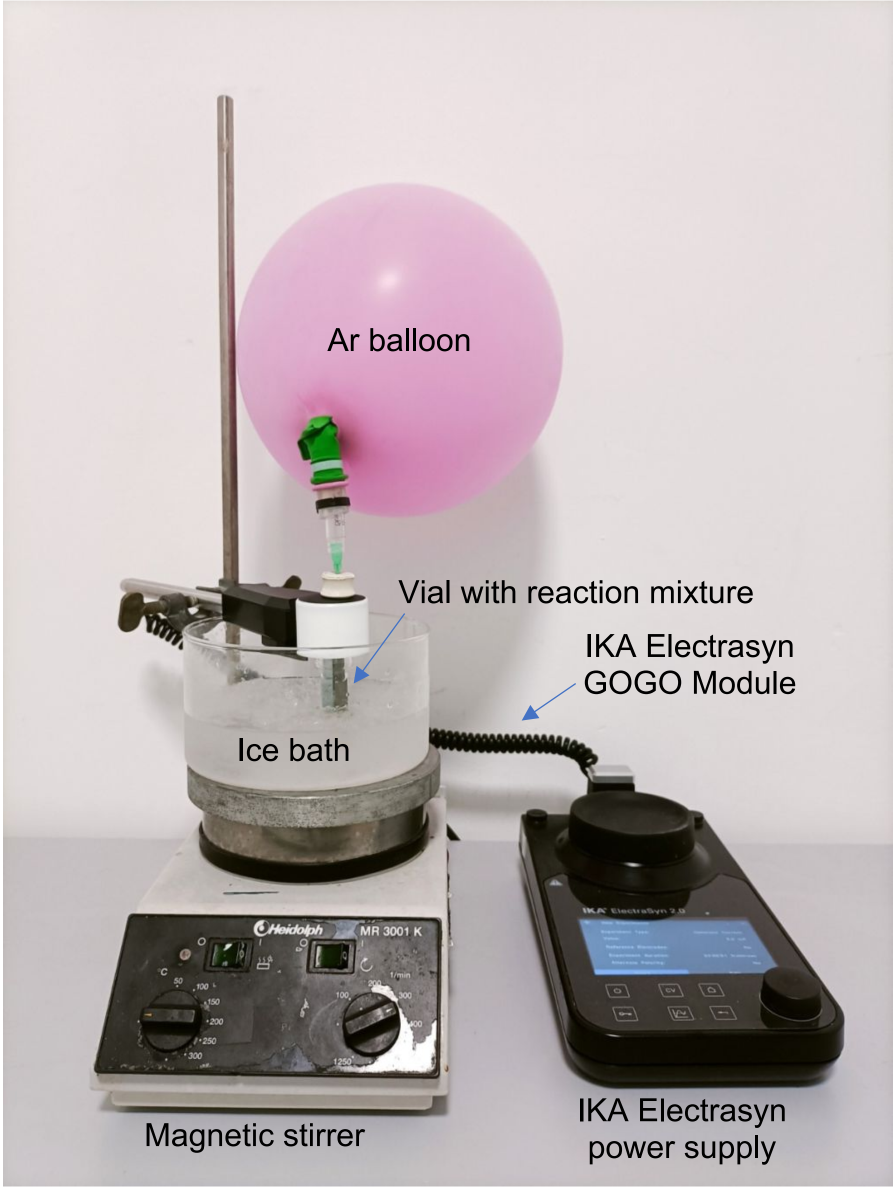

Figure S4. Complete reaction set-up. The IKA Electrasyn GOGO Module is an extension that allows reactions in ElectraSyn 2.0 vials away from the device.

4.2 Characterization data of compounds **3**

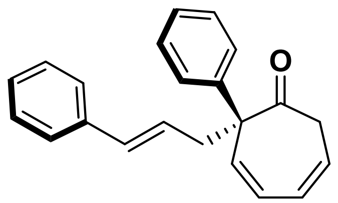

3aa. Pale yellow oil. FC eluent: *n*-hexane/EtOAc: 50:1. Yield = 62%, (0.062 mmol, 20.6 mg). ***e.r.*** = 94:6. **[α]D** = -.42.3 (c = 0.26, $CHCl_3$). **HPLC analysis**: ODH column (280 nm), 30 °C, method: n-Hex:IPA = 90:10, flow 0.5 mL/min, $t_{(R)}$ = 11.4 min, $t_{(S)}$ = 12.1 min. **^{1}H NMR** (600 MHz, $CDCl_3$) δ = 7.38 – 7.35 (m, 2H), 7.35 – 7.31 (m, 2H), 7.29 – 7.22 (m, 5H), 7.19 – 7.16 (m, 1H), 6.39 – 6.34 (m, 2H), 6.10 (dddd, *J* = 10.3, 5.5, 2.0, 0.6 Hz, 1H), 5.93 – 5.85 (m, 2H), 5.62 – 5.56 (m, 1H), 3.30 (ddd, *J* = 14.2, 5.7, 2.0 Hz, 1H), 3.12 (ddd, *J* = 13.9, 7.7, 1.3 Hz, 1H), 3.07 (dd, *J* = 14.2, 6.8 Hz, 1H), 2.83 (ddd, *J* = 13.9, 7.1, 1.5 Hz, 1H); **^{13}C NMR** (151 MHz, $CDCl_3$) δ = 201.7, 139.0, 136.5, 132.1, 132.1, 127.5 (2C), 127.4 (2C), 127.1, 127.0, 126.7 (2C), 126.3, 126.0, 125.1 (2C), 125.0, 122.1, 63.0, 42.1, 40.8; **HRMS (ESI)** m/z: $[M + Na]^+$ calcd. for $C_{22}H_{20}NaO^+$: 323.1406; found 323.1401.

Preparation of **3aa** on 1.0 mmol scale. In a Schlenk tube, under an Ar atmosphere and magnetic stirring, $Pd(acn)_2Cl_2$ (0.10 mmol, 10 mol%, 26.0 mg), and ligand **L1** (0.2 mmol, 20 mol%, 165.0 mg) were stirred in THF (3.0 mL) at room temperature for 1 h. Then, the ElectraSyn vial (10 mL), equipped with a stir bar, was charged with derivative **1a** (3.0 mmol, 546 mg, 3.0 equiv), $TBAPF_6$ (3.0 mmol, 773 mg), TBACl (1.0 mmol, 278 mg) and acetate **2a** (1.0 mmol, 176 mg, 1 equiv). The ElectraSyn vial cap, equipped with anode (Zn) and cathode (W), was inserted into the mixture and closed with a rubber septum. The vessel was evacuated and backfilled with Ar three times, then dry THF (6.0 mL) and the previously formed Pd-complex (in 3.0 mL of THF) was added, and the mixture stirred until complete dissolution of the solids occurred, while bubbling with Ar (balloon, 3 min). Then H_2O (12 equiv, 220 μL) was added via a Hamilton syringe. The reaction mixture was electrolysed (under Ar, balloon) at a constant current of 5.0 mA, for 30 hours at 0°C (5.6 mF, 5.6 F/mol_{2a}). Then, the ElectraSyn vial cap was removed, and the electrodes and vial were rinsed with EtOAc (30 mL) and $NH_4Cl_{(aq)}$ (saturated, 15 mL) and water (15 mL), which were combined with the crude mixture in a separatory funnel. Then, the organic layer was separated, and the aqueous layer was extracted with EtOAc (2 x 20 mL). The combined organic layers were washed with $NH_4Cl_{(aq)}$ (0.1 M, 3 x 20 mL), dried over Na_2SO_4 and concentrated in vacuo. The crude product was finally purified by FC on silica gel (*n*-hexane/EtOAc: 50:1 to afford products **3aa** as a pale yellow oil (131.6 mg, 0.44 mmol, 44% yield).

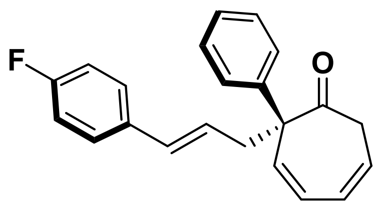

3ab. Pale yellow oil. FC eluent: *n*-hexane/EtOAc: 50:1. Yield = 43%, (0.043 mmol, 13.5 mg). ***e.r.*** = 92:8. **[α]$_D$** = -28.9 (c = 0.14, $CHCl_3$). **HPLC analysis**: ODH column (280 nm), 30 °C, method: n-Hex:IPA = 90:10, flow 0.5 mL/min, $t_{(R)}$ = 11.2 min, $t_{(S)}$ = 12.0 min. **^{1}H NMR** (600 MHz, $CDCl_3$) δ = 7.38 – 7.31 (m, 4H), 7.28 – 7.25 (m, 1H), 7.21 – 7.16 (m, 2H), 6.96 – 6.91 (m, 2H), 6.36 (dd, *J* = 11.4, 5.5 Hz, 1H), 6.32 (dd, *J* = 15.8, 1.5 Hz, 1H), 6.10 (ddd, *J* = 10.3, 5.5, 1.9 Hz, 1H), 5.89 (d, *J* = 11.4 Hz, 1H), 5.80 (dt, *J* = 15.8, 7.3 Hz, 1H), 5.59 (ddd, *J* = 10.3, 6.8, 5.6 Hz, 1H), 3.29 (ddd, *J* = 14.2, 5.7, 2.0 Hz, 1H), 3.12 (ddd, *J* = 13.9, 7.6, 1.3 Hz, 1H), 3.07 (dd, *J* = 14.2, 6.9 Hz, 1H), 2.80 (ddd, *J* = 13.9, 7.1, 1.5 Hz, 1H); **^{13}C NMR** (150 MHz, $CDCl_3$) δ = 201.6, 161.0 (d, *J* = 246.2 Hz), 139.0, 132.6 (d, *J* = 3.3 Hz), 132.1, 130.9, 127.5 (2C), 127.0 (d, *J* = 4.3 Hz, 2C), 126.6 (2C), 126.5, 126.4, 126.3, 124.8 (d, *J* = 2.2 Hz), 122.2, 114.2 (d, *J* = 21.3 Hz, 2C).63.0, 42.1, 40.7;**^{19}F NMR** (565 MHz, $CDCl_3$) δ = -115.34 – -115.44 (m, 1F); **HRMS (ESI)** m/z: $[M + Na]^+$ calcd. for $C_{22}H_{19}FNaO^+$: 341.1312; found 341.1308.

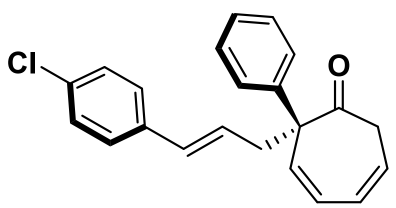

3ac. Pale yellow oil. FC eluent: *n*-hexane/EtOAc: 50:1. Yield = 39%, (0.039 mmol, 13.0 mg). ***e.r.*** = 91:9. **[α]$_D$** = -34.1 (c = 0.17, $CHCl_3$). **HPLC analysis**: ODH column (280 nm), 30 °C, method: n-Hex:IPA = 90:10, flow 0.5 mL/min, $t_{(R)}$ = 11.7 min, $t_{(S)}$ = 12.7 min. **^{1}H NMR** (600 MHz, $CDCl_3$) δ = 7.37 – 7.31 (m, 4H), 7.29 – 7.26 (m, 1H), 7.23 – 7.19 (m, 2H), 7.17 – 7.13 (m, 2H), 6.36 (dd, *J* = 11.40, 5.49 Hz, 1H), 6.31 (d, *J* = 15.83 Hz, 1H), 6.09 (ddd, *J* = 10.28, 5.50, 1.92 Hz, 1H), 5.91 – 5.83 (m, 2H), 5.62 – 5.56 (m, 1H), 3.29 (ddd, *J* = 14.19, 5.72, 2.03 Hz, 1H), 3.13 (ddd, *J* = 13.92, 7.61, 1.33 Hz, 1H), 3.07 (dd, *J* = 14.21, 6.89 Hz, 1H), 2.79 (ddd, *J* = 13.91, 7.11, 1.50 Hz, 1H); **^{13}C NMR** (151 MHz, $CDCl_3$) δ = 202.5, 140.0, 136.0, 133.0, 132.6, 131.9, 128.6 (4C), 128.1, 128.1, 127.7 (2C), 127.4, 127.3 (2C), 126.9, 123.3, 64.0, 43.1, 41.7; **HRMS (ESI)** m/z: $[M + Na]^+$ calcd. for $C_{22}H_{19}ClNaO^+$: 357.1017; found 357.1012.

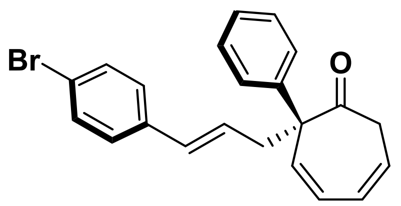

3ad. Pale yellow oil. FC eluent: *n*-hexane/EtOAc: 50:1. Yield = 41%, (0.041 mmol, 15.5 mg). ***e.r.*** = 86:14. **[α]D** = -51 (c = 0.19, $CHCl_3$). **HPLC analysis**: ODH column (280 nm), 30 °C, method: n-Hex:IPA = 90:10, flow 0.5 mL/min, $t_{(R)}$ = 12.1 min, $t_{(S)}$ = 13.1 min. **^{1}H NMR** (600 MHz, $CDCl_3$) δ= 7.38 – 7.31 (m, 6H), 7.29 – 7.26 (m, 1H), 7.11 – 7.07 (m, 2H), 6.36 (dd, *J* = 11.4, 5.5 Hz, 1H), 6.29 (d, *J* = 15.8 Hz, 1H), 6.12 – 6.07 (m, 1H), 5.91 – 5.84 (m, 2H), 5.59 (ddd, *J* = 10.3, 6.9, 5.6 Hz, 1H), 3.29 (ddd, *J* = 14.2, 5.7, 2.0 Hz, 1H), 3.13 (ddd, *J* = 13.9, 7.6, 1.3 Hz, 1H), 3.06 (dd, *J* = 14.2, 6.9 Hz, 1H), 2.78 (ddd, *J* = 13.9, 7.1, 1.5 Hz, 1H); **^{13}C NMR** (151 MHz, $CDCl_3$) δ = 202.5, 140.0, 136.4, 133.0, 131.9, 131.5 (2C), 128.6 (2C), 128.1, 128.1, 127.7 (2C), 127.6 (2C), 127.4, 127.0, 123.3, 120.7, 64.0, 43.1, 41.7; **HRMS (ESI)** m/z: $[M + Na]^+$ calcd. for $C_{22}H_{19}BrNaO^+$: 401.0511; found 401.0518.

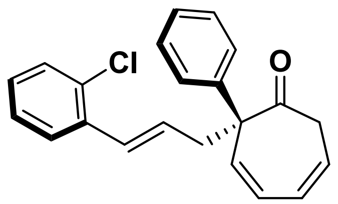

3ae. Tropone derivative **1a** (0.60 mmol, 6.0 equiv.) and reaction time 6 hours (1.12 mF, 11.2 F/mol_{2e}). Pale yellow oil. FC eluent: *n*-hexane/EtOAc: 50:1. Yield = 45%, (0.045 mmol, 15,0 mg). ***e.r.*** = 93.7. **[α]D** = -33.7 (c = 0.12, $CHCl_3$). **HPLC analysis**: ADH column (280 nm), 30 °C, method: n-Hex:IPA = 98:2, flow 1 mL/min, $t_{(R)}$ = 7.2 min, $t_{(S)}$ = 7.9 min. **^{1}H NMR** (600 MHz, $CDCl_3$) δ = 7.39 – 7.36 (m, 2H), 7.35 – 7.32 (m, 3H), 7.32 – 7.26 (m, 2H), 7.15 – 7.10 (m, 2H), 6.73 (d, *J* = 15.81 Hz, 1H), 6.38 (dd, *J* = 11.40, 5.47 Hz, 1H), 6.11 (ddd, *J* = 10.25, 5.48, 1.91 Hz, 1H), 5.91 (d, *J* = 11.42 Hz, 1H), 5.86 (dt, *J* = 15.77, 7.37 Hz, 1H), 5.60 (ddd, *J* = 10.27, 6.92, 5.65 Hz, 1H), 3.30 (ddd, *J* = 14.11, 5.70, 2.04 Hz, 1H), 3.19 (ddd, *J* = 13.87, 7.44, 1.38 Hz, 1H), 3.07 (dd, *J* = 14.14, 6.93 Hz, 1H), 2.84 (ddd, *J* = 13.86, 7.30, 1.47 Hz, 1H);**^{13}C NMR** (151 MHz, $CDCl_3$) δ = 201.5, 138.9, 134.6, 132.0, 131.6, 128.5, 128.4, 128.1, 127.5 (2C), 127.1, 127.0, 127.0, 126.6 (2C), 126.3, 125.7, 125.7, 122.2, 63.0, 42.0, 40.9; **HRMS (ESI)** m/z: $[M + Na]^+$ calcd. for $C_{22}H_{19}ClNaO^+$: 357.1017; found 357.1008.

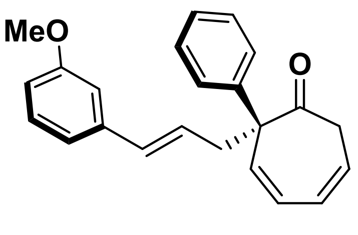

3af. Pale yellow oil. FC eluent: *n*-hexane/EtOAc: 50:1. Yield = 63%, (0.063 mmol, 20.8 mg). ***e.r.*** = 95:5. **[α]D** = -45.9 (c = 0.14, $CHCl_3$). **HPLC analysis**: ID column (280 nm), 30 °C, method: n-Hex:IPA = 80:20, flow 0.5 mL/min, $t_{(R)}$ = 13.1 min, $t_{(S)}$ = 12.3 min. **^{1}H NMR** (600 MHz, $CDCl_3$) δ = 7.39 – 7.35 (m, 2H), 7.33 (dd, *J* = 8.61, 6.73 Hz, 2H), 7.29 – 7.26 (m, 1H), 7.17 (t, *J* = 7.90 Hz, 1H), 6.84 (dt, *J* = 7.62, 1.21 Hz, 1H), 6.77 (t, *J* = 2.04 Hz, 1H), 6.76 – 6.72 (m, 1H), 6.39 – 6.31 (m, 2H), 6.10 (ddd, *J* = 10.34, 5.47, 1.91 Hz, 1H), 5.94 – 5.84 (m, 2H), 5.59 (ddd, *J* = 10.27, 6.85, 5.71 Hz, 1H), 3.78 (s, 3H), 3.30 (ddd, *J* = 14.19, 5.74, 1.96 Hz, 1H), 3.12 (ddd, *J* = 13.86, 7.70, 1.31 Hz, 1H), 3.07 (dd, *J* = 14.19, 6.85 Hz, 1H), 2.81 (ddd, *J* = 13.85, 7.07, 1.47 Hz, 1H);**^{13}C NMR** (151 MHz, $CDCl_3$) δ = 202.7, 159.7, 140.0, 139.0, 133.1, 133.0, 129.4, 128.5 (2C), 128.1, 128.0, 127.7 (2C), 127.3, 126.5, 123.2, 118.8, 112.5, 111.6, 64.0, 55.2, 43.2, 41.8; **HRMS (ESI)** m/z: $[M + Na]^+$ calcd. for $C_{23}H_{22}NaO_2^+$: 353.1512; found 353.1517.

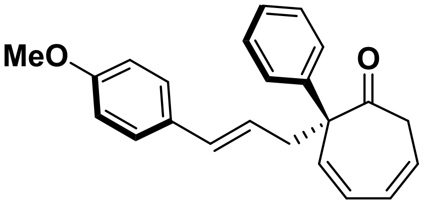

3ag. Pale yellow oil. FC eluent: *n*-hexane/EtOAc: 45:1. Yield = 33%, (0.033 mmol, 10.9 mg). ***e.r.*** = 90:10. **[α]D** = -31.25 (c = 0.17, $CHCl_3$). **HPLC analysis**: ODH column (280 nm), 30 °C, method: n-Hex:IPA = 90:10, flow 0.5 mL/min, $t_{(R)}$ = 13.3 min, $t_{(S)}$ = 14.2 min. **^{1}H NMR** (600 MHz, $CDCl_3$) δ = 7.38 – 7.34 (m, 2H), 7.32 (ddd, *J* = 7.91, 7.02, 1.40 Hz, 2H), 7.29 – 7.24 (m, 1H), 7.19 – 7.13 (m, 2H), 6.81 – 6.76 (m, 2H), 6.35 (dd, *J* = 11.41, 5.45 Hz, 1H), 6.31 (d, *J* = 15.77 Hz, 1H), 6.12 – 6.07 (m, 1H), 5.91 (d, *J* = 11.42 Hz, 1H), 5.74 (ddd, *J* = 15.73, 7.71, 7.02 Hz, 1H), 5.58 (dt, *J* = 10.27, 6.28 Hz, 1H), 3.78 (s, 3H), 3.29 (ddd, *J* = 14.32, 5.93, 2.01 Hz, 1H), 3.11 – 3.02 (m, 2H), 2.80 (ddd, *J* = 13.87, 6.99, 1.48 Hz, 1H); **^{13}C NMR** (151 MHz, $CDCl_3$) δ = 201.9, 157.8, 139.1, 132.3, 131.4, 129.4, 127.5 (2C), 127.1, 126.9, 126.7 (2C), 126.2, 126.2 (2C), 122.8 (2C), 122.1, 112.8, 63.0, 54.3, 42.2, 40.9; **HRMS (ESI)** m/z: $[M + Na]^+$ calcd. for $C_{23}H_{22}NaO_2^+$: 353.1512; found 353.1515.

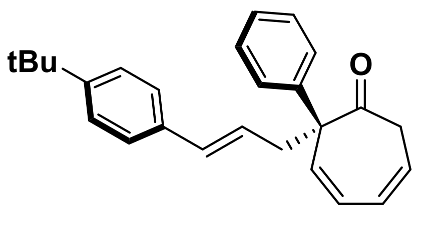

3ah. Pale yellow oil. FC eluent: *n*-hexane/EtOAc: 50:1. Yield = 45%, (0.045 mmol, 16.0 mg). ***e.r.*** = 93:7. **[α]$_D$** = -52.3 (c = 0.39, $CHCl_3$). **HPLC analysis**: ODH column (280 nm), 30 °C, method: n-Hex:IPA = 90:10, flow 0.5 mL/min, $t_{(R)}$ = 9.4 min, $t_{(S)}$ = 10.1 min. **^{1}H NMR** (600 MHz, $CDCl_3$) δ = 7.37 – 7.34 (m, 2H), 7.34 – 7.30 (m, 2H), 7.29 – 7.27 (m, 3H), 7.20 – 7.17 (m, 2H), 6.37 – 6.32 (m, 1H), 6.10 (dddd, *J* = 10.26, 5.45, 1.91, 0.64 Hz, 2H), 5.90 (d, *J* = 11.42 Hz, 1H), 5.85 (ddd, *J* = 15.71, 7.70, 7.01 Hz, 1H), 5.58 (ddd, *J* = 10.28, 6.69, 5.73 Hz, 1H), 3.29 (ddd, *J* = 14.28, 5.80, 1.96 Hz, 1H), 3.11 – 3.04 (m, 2H), 2.83 (ddd, *J* = 13.86, 7.05, 1.50 Hz, 1H), 1.29 (s, 9H);**^{13}C NMR** (151 MHz, $CDCl_3$) δ = 202.9, 150.1, 140.1, 134.8, 133.3, 132.8, 128.5 (2C), 128.1, 127.9, 127.7 (2C), 127.3, 125.8 (2C), 125.3 (2C), 125.2, 123.2, 64.0, 43.2, 42.0, 34.5, 31.3 (3C); **HRMS (ESI)** m/z: $[M + Na]^+$ calcd. for $C_{26}H_{28}NaO^+$: 379.2032; found 379.2034.

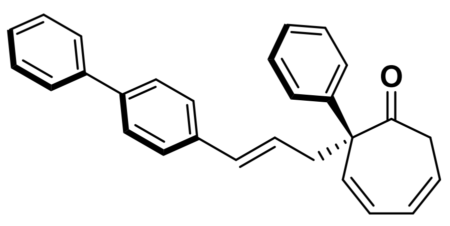

3ai. Tropone derivative **1a** (0.60 mmol, 6.0 equiv.) and reaction time 6 hours (1.12 mF, 11.2 F/mol$_{\mathbf{2i}}$). Pale yellow oil. FC eluent: *n*-hexane/EtOAc: 50:1. The title compound was further purified by preparative TLC (*n*-hexane/EtOAc: 20:1). Yield = 56%, (0.056 mmol, 14.9 mg). ***e.r.*** = 90:10. **[α]$_D$** = -48.9 (c = 0.27, $CHCl_3$). **HPLC analysis**: ODH column (280 nm), 30 °C, method: n-Hex:IPA = 90:10, flow 0.5 mL/min, $t_{(R)}$ = 16.5 min, $t_{(S)}$ = 18.6 min. **^{1}H NMR** (600 MHz, $CDCl_3$) δ = ^{1}H NMR 7.58 – 7.55 (m, 2H), 7.51 – 7.48 (m, 2H), 7.42 (dd, *J* = 8.44, 7.03 Hz, 2H), 7.39 – 7.36 (m, 2H), 7.36 – 7.30 (m, 5H), 7.29 – 7.23 (m, 1H), 6.43 – 6.35 (m, 2H), 6.12 (ddd, *J* = 10.29, 5.46, 1.85 Hz, 1H), 5.98 – 5.91 (m, 2H), 5.60 (ddd, *J* = 10.32, 6.83, 5.72 Hz, 1H), 3.31 (ddd, *J* = 13.92, 6.14, 2.12 Hz, 1H), 3.15 (ddd, *J* = 13.83, 7.64, 1.33 Hz, 1H), 3.08 (dd, *J* = 14.23, 6.83 Hz, 1H), 2.85 (ddd, *J* = 13.86, 7.06, 1.49 Hz, 1H); **^{13}C NMR** (151 MHz, $CDCl_3$) δ = 202.7, 140.8, 140.0, 139.8, 136.5, 133.2, 132.6, 128.8 (2C), 128.6 (2C), 128.1, 128.0, 127.7 (2C), 127.3, 127.2, 127.2 (2C), 126.9 (2C), 126.5 (2C), 126.2, 123.2, 64.0, 43.2, 41.9; **HRMS (ESI)** m/z: $[M + Na]^+$ calcd. for $C_{28}H_{24}NaO^+$ 399.1719; found 399.1724.

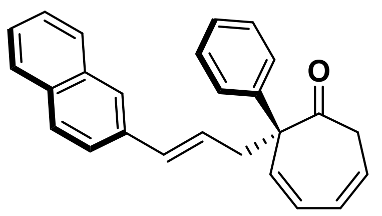

3aj. Tropone derivative **1a** (0.60 mmol, 6.0 equiv.) and reaction time 6 hours (1.12 mF, 11.2 F/mol**2j**). Pale yellow oil. FC eluent: *n*-hexane/EtOAc: 50:1. Yield = 50%, (0.050 mmol, 17.5 mg). ***e.r.*** = 91:9. **[α]D** = -56.8 (c = 0.13, $CHCl_3$). **HPLC analysis**: IC column (280 nm), 30 °C, method: n-Hex:IPA = 95:5, flow 0.6 mL/min, $t_{(R)}$ = 13.6 min, $t_{(S)}$ = 14.8 min. **^{1}H NMR** (600 MHz, $CDCl_3$) δ = 7.77 – 7.74 (m, 2H), 7.71 (d, *J* = 8.5 Hz, 1H), 7.60 (s, 1H), 7.45 – 7.38 (m, 5H), 7.37 – 7.33 (m, 2H), 7.30 – 7.27 (m, 1H), 6.53 (d, *J* = 15.8 Hz, 1H), 6.38 (dd, *J* = 11.4, 5.4 Hz, 1H), 6.11 (dddd, *J* = 10.3, 5.5, 2.0, 0.6 Hz, 1H), 6.02 (dt, *J* = 15.7, 7.4 Hz, 1H), 5.94 (d, *J* = 11.4 Hz, 1H), 5.63 – 5.57 (m, 1H), 3.31 (ddd, *J* = 14.2, 5.7, 1.9 Hz, 1H), 3.19 (ddd, *J* = 13.8, 7.6, 1.3 Hz, 1H), 3.09 (dd, *J* = 14.2, 6.9 Hz, 1H), 2.87 (ddd, *J* = 13.9, 7.1, 1.5 Hz, 1H); **^{13}C NMR** (151 MHz, $CDCl_3$) δ = 202.7, 140.1, 134.9, 133.6, 133.2, 133.2, 132.7, 128.6 (2C), 128.1, 128.1, 128.0, 127.9, 127.7 (2C), 127.6, 127.4, 126.5, 126.2, 125.7, 125.6, 123.5, 123.2, 64.1, 43.2, 42.0; **HRMS (ESI)** m/z: $[M + Na]^+$ calcd. for $C_{26}H_{22}NaO^+$: 373.1563; found 373.1567.

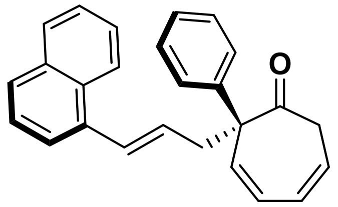

3ak. Pale yellow oil. FC eluent: *n*-hexane/EtOAc: 50:1. The title compound was further purified by preparative TLC (*n*-hexane/EtOAc: 20:1). Yield = 38%, (0.038 mmol, 13.3 mg). ***e.r.*** = 88:12. **[α]D** = -54.8 (c = 0.25, $CHCl_3$). **HPLC analysis**: ADH column (280 nm), 30 °C, method: n-Hex:IPA = 98:2, flow 1.0 mL/min, $t_{(R)}$ = 12.7 min, $t_{(S)}$ = 13.6 min. **^{1}H NMR** (600 MHz, $CDCl_3$) δ = 7.97 – 7.93 (m, 1H), 7.83 – 7.78 (m, 1H), 7.72 (dd, *J* = 7.1, 2.0 Hz, 1H), 7.49 – 7.44 (m, 2H), 7.43 – 7.32 (m, 6H), 7.30 – 7.26 (m, 1H), 7.06 (d, *J* = 15.5 Hz, 1H), 6.40 (dd, *J* = 11.4, 5.5 Hz, 1H), 6.11 (ddd, *J* = 10.4, 5.4, 1.9 Hz, 1H), 5.99 (d, *J* = 11.4 Hz, 1H), 5.92 (dt, *J* = 15.6, 7.4 Hz, 1H), 5.61 (ddd, *J* = 10.3, 6.9, 5.6 Hz, 1H), 3.32 (ddd, *J* = 14.2, 5.8, 2.1 Hz, 1H), 3.25 (ddd, *J* = 13.7, 7.5, 1.3 Hz, 1H), 3.09 (dd, *J* = 14.2, 6.9 Hz, 1H), 2.95 (ddd, *J* = 13.8, 7.2, 1.5 Hz, 1H).; **^{13}C NMR** (151 MHz, $CDCl_3$) δ= 202.5, 140.1, 135.5, 133.5, 133.1, 131.1, 130.7, 129.3, 128.6 (2C), 128.4, 128.1, 128.1, 127.8 (2C), 127.4, 127.3, 125.8, 125.6, 125.6, 124.0, 123.7, 123.3, 64.2, 43.2, 42.2; **HRMS (ESI)** m/z: $[M + Na]^+$ calcd. for $C_{26}H_{22}NaO^+$: 373.1563; found 373.1561.

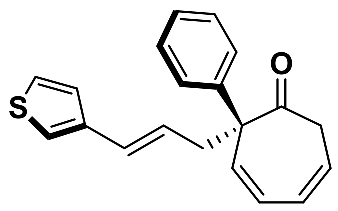

3al. Tropone derivative **1a** (0.60 mmol, 6.0 equiv.) and reaction time 6 hours (1.12 mF, 11.2 F/mol$_{\mathbf{2l}}$). Pale yellow oil. FC eluent: *n*-hexane/EtOAc: 50:1. Yield = 48%, (0.048 mmol, 14,7 mg). ***e.r.*** = 88:12. **[α]$_D$** = -57.1 (c = 0.32, $CHCl_3$). **HPLC analysis**: ODH column (280 nm), 30 °C, method: n-Hex:IPA = 90:10, flow 0.5 mL/min, $t_{(R)}$ = 12.5 min, $t_{(S)}$ = 14.2 min. **^{1}H NMR** (600 MHz, $CDCl_3$) δ = 7.30 (dd, *J* = 5.06, 2.97 Hz, 1H), 7.28 – 7.23 (m, 4H), 7.20 – 7.16 (m, 1H), 7.15 (dd, *J* = 2.99, 1.43 Hz, 1H), 7.07 (dd, *J* = 5.06, 1.39 Hz, 1H), 6.37 (dt, *J* = 15.73, 1.41 Hz, 1H), 6.30 (ddd, *J* = 11.44, 5.44, 0.75 Hz, 1H), 6.15 (ddd, *J* = 10.14, 5.41, 1.99 Hz, 1H), 5.95 (d, *J* = 11.42 Hz, 1H), 5.89 (dt, *J* = 15.70, 7.38 Hz, 1H), 5.68 (ddd, *J* = 10.14, 7.48, 5.59 Hz, 1H), 3.27 (ddd, *J* = 12.51, 5.67, 2.05 Hz, 1H), 3.14 – 3.07 (m, 2H), 2.79 (ddd, *J* = 13.72, 7.25, 1.47 Hz, 1H);**^{13}C NMR** (151 MHz, $CDCl_3$) δ = 201.9, 141.5, 137.5, 133.9, 133.1, 128.5 (2C), 128.1, 127.3, 127.1, 126.8, 126.1 (2C), 125.9, 125.7, 122.8, 122.8, 61.8, 43.0, 41.9; **HRMS (ESI)** m/z: $[M + Na]^+$ calcd. for $C_{20}H_{18}NaOS^+$: 329.0971; found 329.0965.

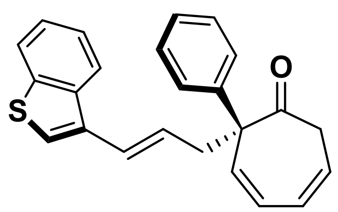

3am. Pale yellow oil. FC eluent: *n*-hexane/EtOAc: 50:1. The title compound was further purified by preparative TLC (*n*-hexane/EtOAc: 20:1). Yield = 47%, (0.047 mmol, 17.4 mg). ***e.r.*** = 87:13. **[α]$_D$** = - 49.2 (c = 0.15, $CHCl_3$). **HPLC analysis**: IC column (280 nm), 30 °C, method: n-Hex:IPA = 98:2, flow 0.7 mL/min, $t_{(R)}$ = 14.5 min, $t_{(S)}$ = 13.8 min. **^{1}H NMR** (600 MHz, $CDCl_3$) δ = 7.84 – 7.80 (m, 1H), 7.77 – 7.74 (m, 1H), 7.42 – 7.38 (m, 2H), 7.38 – 7.30 (m, 4H), 7.30 – 7.25 (m, 2H), 6.61 (dq, *J* = 15.73, 1.24 Hz, 1H), 6.39 (dd, *J* = 11.44, 5.48 Hz, 1H), 6.11 (ddd, *J* = 10.33, 5.51, 1.93 Hz, 1H), 6.00 – 5.90 (m, 2H), 5.63 – 5.57 (m, 1H), 3.31 (ddd, *J* = 14.24, 5.72, 2.01 Hz, 1H), 3.20 (ddd, *J* = 13.88, 7.59, 1.34 Hz, 1H), 3.09 (dd, *J* = 14.22, 6.89 Hz, 1H), 2.87 (ddd, *J* = 13.85, 7.13, 1.50 Hz, 1H);**^{13}C NMR** (151 MHz, $CDCl_3$) δ = ^{13}C NMR (151 MHz, $CDCl_3$) δ 202.7, 140.5, 140.2, 137.9, 134.4, 133.2, 128.8 (2C), 128.3, 128.3, 128.2, 127.9 (2C), 127.6, 125.7, 124.5, 124.3, 123.5, 123.0, 122.2, 121.4, 64.3, 43.3, 42.3; **HRMS (ESI)** m/z: $[M + Na]^+$ calcd. for $C_{24}H_{20}NaOS^+$:379.1127; found 379.1133.

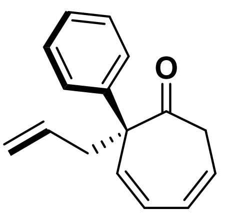

3an. Pale yellow oil. FC eluent: *n*-hexane/EtOAc: 50:1. Yield = 61%, (0.061 mmol, 14.52 mg). ***e.r.*** = 66:34. **[α]$_D$** = -31.5 (c = 0.36, $CHCl_3$). **HPLC analysis**: ODH column (280 nm), 30 °C, method: n-Hex:IPA = 90:10, flow 0.5 mL/min, $t_{(R)}$ = 8.5 min, $t_{(S)}$ = 8.8 min. **^{1}H NMR** (600 MHz, $CDCl_3$) δ = 7.34 – 7.29 (m, 4H), 7.26 – 7.23 (m, 1H), 6.36 (dd, *J* = 11.4, 5.5 Hz, 1H), 6.11 (dddd, *J* = 10.3, 5.5, 2.0, 0.7 Hz, 1H), 5.86 (d, *J* = 11.4 Hz, 1H), 5.58 (dddd, *J* = 10.3, 6.5, 5.7, 0.5 Hz, 1H), 5.49 (dddd, *J* = 17.0, 10.2, 7.5, 6.8 Hz, 1H), 5.03 (ddt, *J* = 17.1, 2.2, 1.4 Hz, 1H), 4.98 (ddt, *J* = 10.2, 2.2, 1.1 Hz, 1H), 3.27 (ddd, *J* = 14.3, 5.7, 1.8 Hz, 1H), 3.04 (dd, *J* = 14.3, 6.8 Hz, 1H), 2.92 (ddt, *J* = 14.0, 7.5, 1.1 Hz, 1H), 2.72 (ddt, *J* = 14.0, 6.8, 1.4 Hz, 1H);**^{13}C NMR** (151 MHz, $CDCl_3$) δ = 202.7, 140.0, 134.2, 133.1, 128.4 (2C), 128.1, 127.8, 127.7 (2C), 127.2, 123.2, 118.0, 63.6, 43.1, 42.7; **HRMS (ESI)** m/z: $[M + Na]^+$ calcd. for $C_{16}H_{16}NaO^+$: 247.1093; found 247.1101.

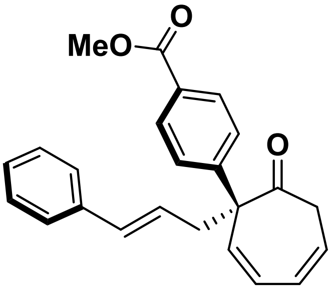

3ba. Pale yellow oil. FC eluent: *n*-hexane/EtOAc: 50:1. Yield = 41%, (0.041 mmol, 14.7 mg). ***e.r.*** = 94:6. **[α]$_D$** = -52.2 (c = 0.18, $CHCl_3$). **HPLC analysis**: ODH column (280 nm), 30 °C, method: n-Hex:IPA = 90:10, flow 0.5 mL/min, $t_{(R)}$ = 21.8 min, $t_{(S)}$ = 19.5 min. **^{1}H NMR** (600 MHz, $CDCl_3$) δ = 8.00 – 7.96 (m, 2H), 7.41 – 7.38 (m, 2H), 7.26 – 7.21 (m, 4H), 7.20 – 7.16 (m, 1H), 6.41 – 6.35 (m, 2H), 6.08 (ddt, *J* = 10.4, 5.4, 1.3 Hz, 1H), 5.91 (d, *J* = 11.3 Hz, 1H), 5.86 (ddd, *J* = 15.7, 7.8, 6.9 Hz, 1H), 5.53 (dt, *J* = 10.4, 6.0 Hz, 1H), 3.91 (s, 3H), 3.28 (ddd, *J* = 15.4, 6.1, 1.6 Hz, 1H), 3.16 – 3.04 (m, 2H), 2.83 (ddd, *J* = 13.9, 7.0, 1.5 Hz, 1H); **^{13}C NMR** (151 MHz, $CDCl_3$) δ = 203.0, 166.8, 145.1, 137.3, 133.6, 132.2, 129.7 (2C), 129.1, 128.9, 128.5 (2C), 128.4, 127.6 (2C), 127.2, 126.1 (2C), 125.3, 123.6, 64.1, 52.2, 43.5, 42.2; **HRMS (ESI)** m/z: $[M + H]^+$ calcd. for $C_{24}H_{22}NaO_3^+$: 381.1461; found 381.1467.

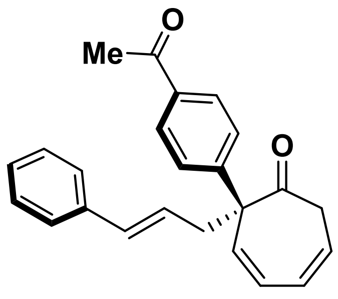

3ca. Pale yellow oil. FC eluent: *n*-hexane/EtOAc: 50:1. Yield = 63%, (0.063 mmol, 20.8 mg). ***e.r.*** = 79:21. **[α]$_D$** = -28.5 (c = 0.16, $CHCl_3$). **HPLC analysis**: ODH column (280 nm), 30 °C, method: n-Hex:IPA = 90:10, flow 0.5 mL/min, $t_{(R)}$ = 26.0 min, $t_{(S)}$ = 28.3 min. **^{1}H NMR** (600 MHz, $CDCl_3$) δ = 7.94 – 7.87 (m, 2H), 7.46 – 7.38 (m, 2H), 7.27 – 7.22 (m, 4H) partially overlapped with the residual solvent peak, 7.18 (ddt, *J* = 8.58, 6.34, 1.64 Hz, 1H), 6.43 – 6.35 (m, 2H), 6.09 (ddt, *J* = 10.37, 5.36, 1.35 Hz, 1H), 5.92 (d, *J* = 11.28 Hz, 1H), 5.87 (ddd, *J* = 15.74, 7.77, 6.92 Hz, 1H), 5.54 (dt, *J* = 10.38, 6.08 Hz, 1H), 3.29 (ddd, *J* = 15.36, 6.16, 1.63 Hz, 1H), 3.14 – 3.10 (m, 1H), 3.08 (ddd, *J* = 13.95, 7.83, 1.27 Hz, 1H), 2.84 (ddd, *J* = 13.96, 6.97, 1.49 Hz, 1H), 2.59 (s, 3H);**^{13}C NMR** (151 MHz, $CDCl_3$) δ = 203.0, 197.7, 145.3, 137.3, 136.0, 133.7, 132.3, 129.0, 128.5 (4C), 128.5, 127.8 (2C), 127.3, 126.1 (2C), 125.2, 123.6, 64.1, 43.5, 42.3, 26.6; **HRMS (ESI)** m/z: $[M + Na]^+$ calcd. for $C_{24}H_{22}NaO_2^+$: 365.1512; found 365.1516.

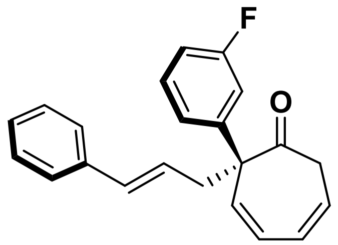

3da. Pale yellow oil. FC eluent: *n*-hexane/EtOAc: 50:1. The title compound was further purified by preparative TLC (*n*-hexane/EtOAc: 20:1). Yield = 36%, (0.036 mmol, 11.5 mg). ***e.r.*** = 88:12. **[α]$_D$** = -31.8 (c = 0.17, $CHCl_3$). **HPLC analysis**: IC column (280 nm), 30 °C, method: n-Hex:IPA = 95:5, flow 0.5 mL/min, $t_{(R)}$ = 12.5 min, $t_{(S)}$ = 11.4 min. **^{1}H NMR** (600 MHz, $CDCl_3$) δ= 7.32 – 7.27 (m, 1H), 7.27 – 7.23 (m, 4H), 7.21 – 7.17 (m, 1H), 7.12 (ddd, *J* = 7.9, 1.9, 1.0 Hz, 1H), 7.07 (ddd, *J* = 10.6, 2.5, 1.8 Hz, 1H), 6.96 (tdd, *J* = 8.3, 2.6, 0.9 Hz, 1H), 6.42 – 6.35 (m, 2H), 6.11 (dddd, *J* = 10.3, 5.4, 1.8, 0.8 Hz, 1H), 5.92 – 5.84 (m, 2H), 5.59 (dt, *J* = 10.3, 6.2 Hz, 1H), 3.29 (ddd, *J* = 14.6, 5.9, 1.8 Hz, 1H), 3.10 (dd, *J* = 14.9, 6.6 Hz, 1H) partially overlapped with 3.07 (ddd, *J* = 13.9, 7.7, 1.3 Hz, 1H), 2.80 (ddd, *J* = 13.9, 7.0, 1.5 Hz, 1H); **^{13}C NMR** (150 MHz, $CDCl_3$) δ = 202.7, 162.8 (d, *J* = 246.3 Hz), 142.6 (d, *J* = 6.7 Hz), 137.3, 133.5, 132.5, 129.9 (d, *J* = 8.2 Hz), 128.6, 128.5 (2C), 128.3, 127.2, 126.1 (2C), 125.5, 123.4, 123.3 (d, *J* = 2.7 Hz), 114.8 (d, *J* = 22.5 Hz), 114.3 (d, *J* = 21.2 Hz), 63.7 (d, *J* = 1.4 Hz), 43.3, 42.1; **^{19}F NMR** (565 MHz, $CDCl_3$) δ = -112.09 – -112.43 (m, 1F); **HRMS (ESI)** m/z: $[M + Na]^+$ calcd. for $C_{22}H_{19}FNaO^+$: 341.1312; found 341.1314.

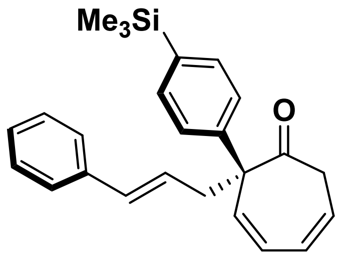

3ea. Pale yellow oil. FC eluent: *n*-hexane/EtOAc: 50:1. Yield = 48%, (0.048 mmol, 17.9 mg). ***e.r.*** = 93:7. **[α]$_D$** = -45.2 (c = 0.18, $CHCl_3$). **HPLC analysis**: ODH column (280 nm), 30 °C, method: n-Hex:IPA = 93:7, flow 0.5 mL/min, $t_{(R)}$ = 8.2 min, $t_{(S)}$ = 9.0 min. **^{1}H NMR** (600 MHz, $CDCl_3$) δ = 7.49 – 7.46 (m, 2H), 7.38 – 7.34 (m, 2H), 7.26 – 7.22 (m, 4H), 7.17 (ddd, *J* = 8.55, 5.91, 2.22 Hz, 1H), 6.40 – 6.33 (m, 2H), 6.12 (ddd, *J* = 10.27, 5.48, 1.97 Hz, 1H), 5.94 – 5.86 (m, 2H), 5.62 (ddd, *J* = 10.24, 7.16, 5.61 Hz, 1H), 3.29 (ddd, *J* = 13.46, 5.65, 2.06 Hz, 1H), 3.17 (ddd, *J* = 13.84, 7.53, 1.31 Hz, 1H), 3.06 (dd, *J* = 13.50, 7.20 Hz, 1H), 2.80 (ddd, *J* = 13.84, 7.16, 1.47 Hz, 1H), 0.26 (s, 9H); **^{13}C NMR** (151 MHz, $CDCl_3$) δ = 203.5, 141.8, 140.6, 138.7, 134.7 (2C), 134.7, 134.2, 129.5 (2C), 129.2, 128.9, 128.2, 128.1 (2C), 127.2 (3C), 124.0, 65.2, 44.3, 42.7, 0.0 (3C); **HRMS (ESI)** m/z: $[M + Na]^+$ calcd. for $C_{25}H_{28}NaOSi^+$: 395.1802; found 395.1811.

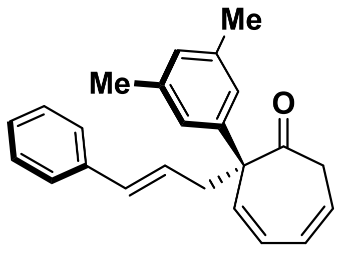

3fa. Pale yellow oil. FC eluent: *n*-hexane/EtOAc: 50:1. Yield = 40%, (0.040 mmol, 13.1 mg). ***e.r.*** = 93:7. **[α]$_D$** = -51.9 (c = 0.25, $CHCl_3$). **HPLC analysis**: ODH column (280 nm), 30 °C, method: n-Hex:IPA = 90:10, flow 0.5 mL/min, $t_{(R)}$ = 8.6 min, $t_{(S)}$ = 9.7 min. **^{1}H NMR** (600 MHz, $CDCl_3$) δ = 7.25 (d, *J* = 5.99 Hz, 4H), 7.17 (ddd, *J* = 8.57, 5.64, 2.54 Hz, 1H), 6.99 (s, 2H), 6.91 (s, 1H), 6.36 (dd, *J* = 15.79, 1.41 Hz, 1H), 6.33 (ddd, *J* = 11.54, 5.55, 0.72 Hz, 1H), 6.11 (ddd, *J* = 10.20, 5.54, 2.14 Hz, 1H), 5.92 – 5.84 (m, 2H), 5.64 (ddd, *J* = 10.20, 7.44, 5.43 Hz, 1H), 3.29 (ddd, *J* = 13.26, 5.53, 2.23 Hz, 1H), 3.13 (ddd, *J* = 13.85, 7.56, 1.32 Hz, 1H), 3.04 (dd, *J* = 13.23, 7.47 Hz, 1H), 2.78 (ddd, *J* = 13.84, 7.10, 1.50 Hz, 1H), 2.31 (s, 6H); **^{13}C NMR** (151 MHz, $CDCl_3$) δ = 202.3, 140.1, 138.0, 137.6, 133.7, 132.8, 129.1, 128.4 (2C), 128.0, 127.4, 127.0, 126.4, 126.1 (2C), 125.6 (2C), 122.9, 63.9, 42.9, 41.5, 21.5 (2C); **HRMS (ESI)** m/z: $[M + H]^+$ calcd. for $C_{24}H_{24}NaO^+$ 351.1719; found 351.1713.

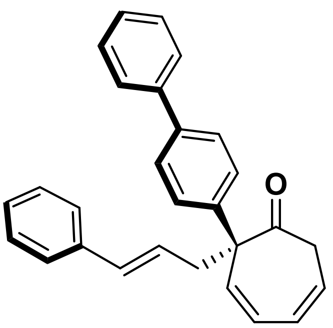

3ga. Pale yellow oil. FC eluent: *n*-hexane/EtOAc: 50:1. The title compound was further purified by preparative TLC (*n*-hexane/EtOAc: 20:1). Yield = 46%, (0.046 mmol, 17.2 mg). ***e.r.*** = 90:10. **[α]D** = -45.4 (c = 0.16, $CHCl_3$). **HPLC analysis**: ODH column (280 nm), 30 °C, method: n-Hex:IPA = 90:10, flow 0.5 mL/min, $t_{(R)}$ = 19.7 min, $t_{(S)}$ = 15.9 min. **^{1}H NMR** (600 MHz, $CDCl_3$) δ= 7.63 – 7.59 (m, 2H), 7.59 – 7.56 (m, 2H), 7.47 – 7.43 (m, 4H), 7.38 – 7.34 (m, 1H), 7.28 – 7.26 (m, 4H), 7.19 (ddd, *J* = 8.8, 5.3, 3.7 Hz, 1H), 6.44 – 6.37 (m, 2H), 6.14 (ddd, *J* = 10.3, 5.5, 1.9 Hz, 1H), 5.99 – 5.90 (m, 2H), 5.64 (ddd, *J* = 10.3, 6.9, 5.7 Hz, 1H), 3.34 (ddd, *J* = 13.9, 5.8, 2.0 Hz, 1H), 3.17 (ddd, *J* = 13.9, 7.7, 1.3 Hz, 1H), 3.12 (dd, *J* = 14.0, 6.9 Hz, 1H), 2.88 (ddd, *J* = 13.9, 7.0, 1.5 Hz, 1H); **^{13}C NMR** (150 MHz, $CDCl_3$) δ = 201.6, 139.4, 139.1, 138.1, 136.5, 132.3, 132.1, 127.8 (2C), 127.4 (2C), 127.1, 127.1 (2C), 127.0, 126.4, 126.1 (2C), 126.0, 126.0 (2C), 125.1 (2C), 125.0, 122.1, 62.8, 42.2, 40.8; **HRMS (ESI)** m/z: $[M + Na]^+$ calcd. for $C_{28}H_{24}NaO^+$ 399.1719; found 399.1716.

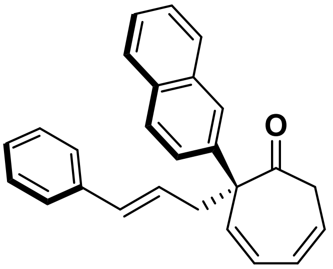

3ha. Pale yellow oil. FC eluent: *n*-hexane/EtOAc: 50:1. Yield = 49%, (0.049 mmol, 17.2 mg). ***e.r.*** = 86:14. **[α]D** = -46.6 (c = 0.12, $CHCl_3$). **HPLC analysis**: ODH column (280 nm), 30 °C, method: n-Hex:IPA = 90:10, flow 0.5 mL/min, $t_{(R)}$ = 14.2 min, $t_{(S)}$ = 15.5 min. **^{1}H NMR** (600 MHz, $CDCl_3$) δ = 7.80 – 7.69 (m, 4H), 7.47 – 7.39 (m, 3H), 7.17 – 7.14 (m, 3H), 7.09 (ddt, *J* = 7.16, 5.27, 2.24 Hz, 1H), 6.41 – 6.29 (m, 2H), 6.08 – 6.01 (m, 1H), 5.95 (d, *J* = 11.41 Hz, 1H), 5.84 (ddd, *J* = 15.80, 7.84, 6.88 Hz, 1H), 5.51 (dt, *J* = 10.34, 6.23 Hz, 1H), 5.23 (s, 1H), 3.28 (ddd, *J* = 14.41, 5.82, 1.91 Hz, 1H), 3.10 (ddd, *J* = 13.98, 7.83, 1.24 Hz, 1H), 3.01 (dd, *J* = 14.43, 6.72 Hz, 1H), 2.89 (ddd, *J* = 13.94, 6.87, 1.50 Hz, 1H); **^{13}C NMR** (151 MHz, $CDCl_3$) δ = 202.9, 137.5, 137.4, 133.2, 133.2, 133.1, 132.5, 128.4 (2C), 128.3, 128.2, 128.1 (2C), 127.5, 127.2, 127.1, 126.3, 126.2, 126.1 (2C), 126.0, 125.2, 123.4, 64.1, 43.2, 42.0; **HRMS (ESI)** m/z: $[M + Na]^+$ calcd. for $C_{26}H_{22}NaO^+$: 373.1563; found 373.1561.

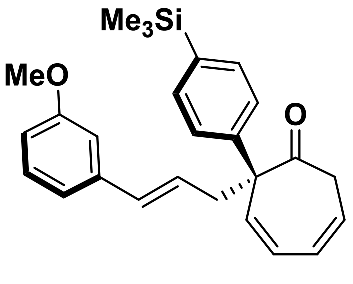

3ef. Pale yellow oil. FC eluent: *n*-hexane/EtOAc: 50:1. Yield = 70%, (0.07 mmol, 28.14 mg). ***e.r.*** = 96:4. **[α]D** = -36.4 (c = 0.22, $CHCl_3$). **HPLC analysis**: ODH column (280 nm), 30 °C, method: n-Hex:IPA = 95:5, flow 0.6 mL/min, $t_{(R)}$ = 9.5 min, $t_{(S)}$ = 10.4 min. **^{1}H NMR** (600 MHz, $CDCl_3$) δ= 7.49 – 7.46 (m, 2H), 7.38 – 7.34 (m, 2H), 7.18 (t, *J* = 7.92 Hz, 1H), 6.85 (dt, *J* = 7.63, 1.23 Hz, 1H), 6.78 (t, *J* = 2.05 Hz, 1H), 6.74 (ddd, *J* = 8.16, 2.63, 0.92 Hz, 1H), 6.40 – 6.30 (m, 2H), 6.12 (ddd, *J* = 10.26, 5.46, 1.99 Hz, 1H), 5.92 – 5.84 (m, 2H), 5.62 (ddd, *J* = 10.23, 7.21, 5.61 Hz, 1H), 3.78 (s, 3H), 3.29 (ddd, *J* = 13.60, 5.68, 2.17 Hz, 1H), 3.17 (ddd, *J* = 13.84, 7.53, 1.29 Hz, 1H), 3.06 (dd, *J* = 13.47, 7.23 Hz, 1H), 2.79 (ddd, *J* = 13.87, 7.17, 1.44 Hz, 1H), 0.26 (s, 9H); **^{13}C NMR** (151 MHz, $CDCl_3$) δ = 202.5, 159.9, 140.8, 139.7, 139.2, 133.8 (2C), 133.7, 133.1, 129.6, 128.3,128.0, 127.2 (2C), 126.7, 123.1, 119.0, 112.7, 111.7, 64.2, 55.4, 43.3, 41.7, -0.9 (3C). **HRMS (ESI)** m/z: $[M + Na]^+$ calcd. for $C_{26}H_{30}NaO_2Si^+$: 425.1907; found 425.1914.

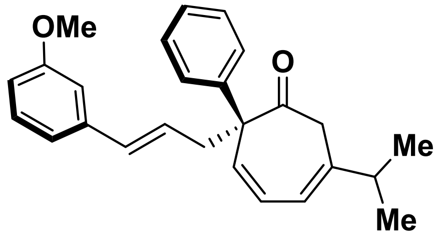

3if. Pale yellow oil. FC eluent: *n*-hexane/EtOAc: 50:1. Yield = 71%, (0.071 mmol, 26.6 mg). ***e.r.*** = 77:23. **[α]D** = -63.2 (c = 0.29, $CHCl_3$). **HPLC analysis**: ODH column (280 nm), 30 °C, method: n-Hex:IPA = 95:5, flow 0.7 mL/min, $t_{(R)}$ = 11.5 min, $t_{(S)}$ = 12.4 min. **^{1}H NMR** (600 MHz, $CDCl_3$) δ= 7.43 – 7.40 (m, 2H), 7.35 – 7.31 (m, 2H), 7.25 – 7.22 (m, 1H), 7.17 (t, *J* = 7.9 Hz, 1H), 6.84 (dt, *J* = 7.7, 1.3 Hz, 1H), 6.77 (t, *J* = 2.0 Hz, 1H), 6.73 (ddd, *J* = 8.2, 2.6, 0.9 Hz, 1H), 6.37 – 6.28 (m, 2H), 5.91 – 5.84 (m, 2H), 5.83 (d, *J* = 11.5 Hz, 1H), 3.78 (s, 3H), 3.27 (dt, *J* = 12.4, 1.0 Hz, 1H), 3.12 (ddd, *J* = 13.8, 7.6, 1.3 Hz, 1H), 2.99 (d, *J* = 12.4 Hz, 1H), 2.81 (ddd, *J* = 13.8, 7.1, 1.5 Hz, 1H), 2.34 (h, *J* = 6.8 Hz, 1H), 0.93 (d, *J* = 6.8 Hz, 3H), 0.91 (d, *J* = 6.8 Hz, 3H); **^{13}C NMR** (150 MHz, $CDCl_3$) δ = 202.8, 159.7, 141.8, 140.3, 139.1, 132.7, 131.8, 129.4, 128.5 (2C), 128.2, 127.8 (2C), 127.3, 126.7, 120.2, 118.8, 112.6, 111.4, 63.4, 55.2, 45.0, 41.4, 36.3, 20.8, 20.7; **HRMS (ESI)** m/z: $[M + Na]^+$ calcd. for $C_{26}H_{28}NaO_2^+$:395.1982; found 395.1988.

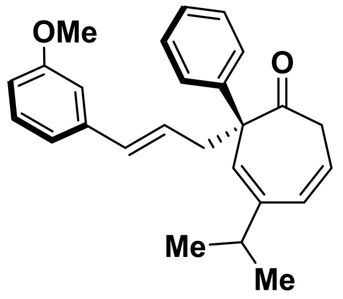

3jf. Pale yellow oil. FC eluent: *n*-hexane/EtOAc: 50:1. Yield = 54%, (0.054 mmol, 18.6 mg). ***e.r.*** = 81:19. **[α]D** = -71.1 (c = 0.31, $CHCl_3$). **HPLC analysis**: ODH column (280 nm), 30 °C, method: n-Hex:IPA = 95:5, flow 0.5 mL/min, $t_{(R)}$ = 10.9 min, $t_{(S)}$ = 12.6 min. **^{1}H NMR** (600 MHz, $CDCl_3$) δ= 7.31 – 7.27 (m, 4H), 7.25 – 7.21 (m, 1H), 7.17 (t, *J* = 7.9 Hz, 1H), 6.83 (dd, *J* = 7.7, 1.3 Hz, 1H), 6.77 (t, *J* = 2.1 Hz, 1H), 6.73 (ddd, *J* = 8.1, 2.6, 0.9 Hz, 1H), 6.30 (d, *J* = 15.8 Hz, 1H), 6.08 – 6.00 (m, 1H), 5.89 (dt, *J* = 15.8, 7.4 Hz, 1H), 5.68 (s, 1H), 5.51 (dt, *J* = 10.5, 6.1 Hz, 1H), 3.78 (s, 3H), 3.27 (ddd, *J* = 15.3, 6.4, 1.4 Hz, 1H), 3.13 – 3.02 (m, 2H), 2.72 (ddd, *J* = 13.7, 7.2, 1.4 Hz, 1H), 2.51 (hept, *J* = 6.2, 5.6 Hz, 1H), 1.14 – 1.06 (m, 6H);**^{13}C NMR** (150 MHz, $CDCl_3$) δ = 204.3, 159.7, 146.7, 140.6, 139.1, 132.9, 129.9, 129.4, 128.3 (2C), 127.4 (2C), 127.0, 126.8, 126.2, 123.1, 118.8, 112.4, 111.5, 62.4, 55.2, 43.1, 42.7, 35.9, 22.2, 22.2; **HRMS (ESI)** m/z: $[M + Na]^+$ calcd. for $C_{26}H_{28}NaO_2^+$:395.1982; found 395.1977.

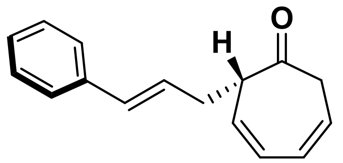

3ka. Pale yellow oil. FC eluent: *n*-hexane/EtOAc: 50:1. Yield = 48%, (0.048 mmol, 10.8 mg). ***e.r.*** = 50:50. **HPLC analysis**: IC column (280 nm), 30 °C, method: n-Hex:IPA = 98:2, flow 0.7 mL/min, $t_{(R)}$ = 11.7 min, $t_{(S)}$ = 11.9 min. Although only partial separation of the peaks was observed, the analysis revealed incontrovertibly that a near racemate was obtained. **^{1}H NMR** (600 MHz, $CDCl_3$) δ = 7.36 – 7.32 (m, 2H), 7.30 – 7.27 (m, 2H), 7.22 – 7.18 (m, 1H), 6.46 (d, *J* = 15.8 Hz, 1H), 6.34 – 6.29 (m, 1H), 6.28 – 6.24 (m, 1H), 6.20 (dt, *J* = 15.8, 7.2 Hz, 1H), 5.85 (ddd, *J* = 10.9, 7.5, 3.8 Hz, 1H), 5.63 (dd, *J* = 10.3, 4.8 Hz, 1H), 3.25 (dd, *J* = 18.4, 7.5 Hz, 1H), 3.16 – 3.11 (m, 1H), 3.03 – 2.96 (m, 1H), 2.80 (dddd, *J* = 14.3, 7.0, 5.7, 1.6 Hz, 1H), 2.59 (dtd, *J* = 14.5, 7.7, 1.4 Hz, 1H); **^{13}C NMR** (150 MHz, $CDCl_3$) δ = 209.2, 137.4, 132.0, 129.6, 129.3, 128.6, 128.5 (2C), 127.6, 127.1, 126.1 (2C), 125.6, 53.6, 44.9, 32.3; **HRMS (ESI)** m/z: $[M + Na]^+$ calcd. for $C_{16}H_{16}NaO^+$: 247.1093: found 247.1099.

5. Transformations of Product 3aa

5.1 Preparation and Characterization of Alcohol **4**

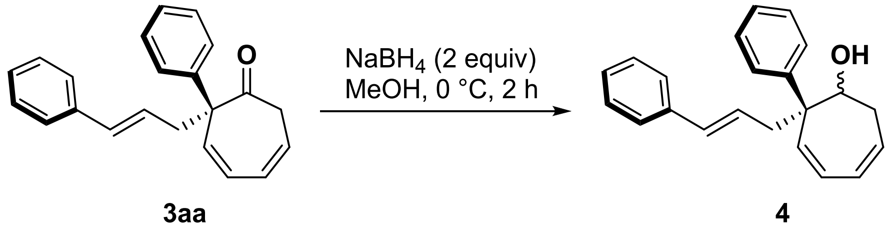

In a small vial, $NaBH_4$ (7.4 mg, 0.2 mmol) was added to a solution of compound **3aa** (30.0 mg, 0.1 mmol) in MeOH (0.5 mL) at 0 °C. The reaction mixture was stirred at the same temperature for 2 h and then quenched with with EtOAc (3 mL), $NH_4Cl_{(aq)}$ (saturated, 3 mL) and water (1 mL), and finally transferred to a separatory funnel. Then, the organic layer was separated, and the aqueous layer was extracted with EtOAc (2 x 3 mL). The combined organic layers were washed with $NH_4Cl_{(aq)}$ (0.1 M, 2 x 3 mL), dried over Na_2SO_4 and concentrated in vacuo. The crude product was finally purified by FC on silica gel to afford pure products **4**.

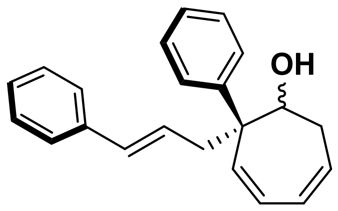

Pale yellow oil. FC eluent: *n*-hexane/EtOAc: 10:1. Yield = 95%, (0.095 mmol, 29.4 mg). ***d.r.*** = 2.7.1. ***e.r.*** *(major diastereoisomer)* = 93:7. ***e.r.*** *(minor diastereoisomer)* = 97:3. **HPLC analysis**: ADH column (280 nm), 30 °C, method: n-Hex:IPA = 95:5, flow 1 mL/min, major diastereoisomer: $t_{(R)}$ = 8.7 min, $t_{(S)}$ = 7.9 min, minor diastereoisomer: $t_{(R)}$ = 12.1 min, $t_{(S)}$ = 9.9 min.. **^{1}H NMR** (600 MHz, $CDCl_3$) δ= 7.45 – 7.41 (m, 2H minor), 7.36 (dd, *J* = 8.5, 7.0 Hz, 2H minor), 7.34 – 7.30 (m, 2H major), 7.30 – 7.18 (m, 7H major + 6H minor), 7.17 – 7.13 (m, 1H major), 6.52 (d, *J* = 15.8 Hz, 1H minor), 6.44 (d, *J* = 16.3 Hz, 1H major),6.25 (dd, *J* = 12.1, 7.3 Hz, 1H major), 6.13 (ddd, *J* = 15.9, 8.7, 5.9 Hz, 1H minor), 6.09 (dd, *J* = 12.0, 7.0 Hz, 1H minor), 5.97 (ddd, *J* = 11.6, 7.3, 3.0 Hz, 1H major), 5.91 – 5.86 (m, 1H minor) partially overlapped with 5.86 (ddd, *J* = 15.8, 9.4, 5.3 Hz, 1H major), 5.81 (d, *J* = 12.1 Hz, 1H major) overlapped with 5.81 (d, *J* = 12.1 Hz, 1H minor), 5.67 (ddd, *J* = 11.1, 6.5, 4.0 Hz, 1H minor), 5.55 (dddd, *J* = 11.7, 6.6, 3.0, 1.0 Hz, 1H major), 4.18 – 4.12 (m, 1H major), 4.03 – 3.99 (m, 1H minor), 3.01 (ddd, *J* = 14.3, 9.4, 1.0 Hz, 1H major), 2.95 (ddd, *J* = 14.2, 5.4, 1.7 Hz, 1H major) overlapped with 2.97 – 2.93 (m, 1H minor), 2.88 (ddd, *J* = 14.0, 5.9, 1.8 Hz, 1H minor), 2.48 (ddd, *J* = 19.3, 6.7, 4.8 Hz, 1H major) partially overlapped with 2.46 – 2.42 (m, 1H minor), 2.28 – 2.23 (m, 1H minor), 2.07 (dq, *J* = 19.6, 3.4 Hz, 1H major). **^{13}C NMR** (150 MHz, $CDCl_3$) δ = 143.4 (major), 141.6 (minor), 136.4

(minor), 136.4 (major), 133.9 (minor), 132.5 (major), 132.2 (major), 132.1 (minor), 129.2 (major), 127.9 (minor), 127.8 (major), 127.7 (minor), 127.4 (minor), 127.4 (major), 127.1 (minor), 126.1 (minor), 126.0 (major), 126.0 (major), 125.9 (major), 125.7 (minor), 125.5 (major), 125.4 (minor), 125.2(major), 125.1 (minor), 125.0 (major), 124.6 (minor), 124.2 (major), 123.8 (minor), 73.4 (major), 72.0 (minor), 54.4 (major), 53.7 (minor), 41.7 (major), 41.1 (minor), 35.4 (minor), 34.4 (major); **HRMS (ESI)** m/z: $[M + Na]^+$ calcd. for $C_{22}H_{22}NaO^+$: 325.1563; found 325.1554.

5.1 Preparation and Characterization of Saturated Ketone **5**

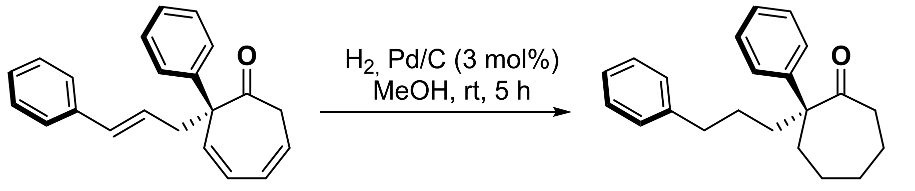

3aa **5**

In a 2-necked round bottom flask, a mixture of compound **3aa** (30.0 mg, 0.1 mmol) and Pd/C (10% wt, 3.2 mg, 0.003 mmol, 3.0 mol%) in MeOH (1 mL) was stirred under H_2 atmosphere (balloon) at room temperature for 5 h. The reaction mixture was then diluted with EtOAc (2 mL) and filtered through a short pad of Celite, washing with EtOAc (3 x 2 mL). The crude mixture was evaporated in vacuo to obtain product **5** without the need of further purification.

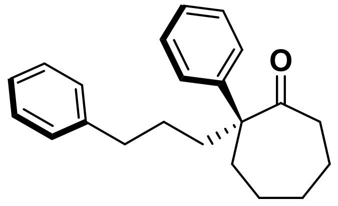

5. Pale yellow oil. Yield = 92%, (0.092 mmol, 28.6 mg). ***e.r.*** = 92:8. **[α]$_D$** = -12 (c = 0.24, $CHCl_3$). **HPLC analysis**: ODH column (280 nm), 30 °C, method: n-Hex:IPA = 95:5, flow 0.7 mL/min, $t_{(S)}$ = 14.8 min, $t_{(R)}$ = 15.3 min. **^{1}H NMR** (600 MHz, $CDCl_3$) δ = 7.32 – 7.28 (m, 2H), 7.24 – 7.19 (m, 3H), 7.15 – 7.11 (m, 3H), 7.06 – 7.03 (m, 2H), 2.52 – 2.41 (m, 3H), 2.32 – 2.21 (m, 2H), 2.12 – 2.01 (m, 2H), 1.89 – 1.75 (m, 2H), 1.71 – 1.47 (m, 4H), 1.45 – 1.34 (m, 3H); **^{13}C NMR** (151 MHz, $CDCl_3$) δ = 213.5, 141.4, 141.2, 127.5 (2C), 127.3 (2C), 127.1 (2C), 125.8 (2C), 125.6, 124.6, 57.9, 39.9, 35.6, 35.3, 31.3, 29.5, 25.9, 25.1, 23.1; **HRMS (ESI)** m/z: [M + Na]$^+$ calcd. for $C_{22}H_{26}NaO$: 329.1876; found 329.1881.

6. Determination of the Absolute Configuration

Compound **3aa** was chosen as the prototype for the assignment of the absolute configuration.

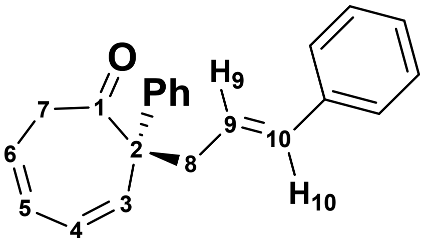

6.1 Assignment of the relative configuration

The conformational space of compound **3aa** was sampled using CREST (version 2.12).[7] Using a meta-dynamic calculation at the tight-binding GFN2-xTB[8] level considering acetonitrile as an implicit solvation thanks to the GBSA theory.[9] All the unique conformers from the output (69 geometries) were further optimized, sorted and pruned using Gaussian 16 (version 6.0.0) using the DFT B3LYP/6-31G(d) level of theory. After optimization, the 69 starting structures were clustered into 5 geometries (named as **c5**, **c7**, **c8**, **c11** and **c21**), that were further optimized at the B3LYP/6-311G(d,p) level, including the solvent acetonitrile with IEFPCM model. Being **c7** and **c11** interconnected by rotation of the exocyclic CH_2-CH bond (C_8-C_9), three additional conformations (i.e. **c5b**, **c8b** and **c21b**) were built by C_8-C_9 rotation and optimized. Frequency analysis confirmed that all the optimized structures corresponded to energy minima (no imaginary frequency). The relative energies among conformation were then evaluated as ZPE-corrected enthalpy and ZPE-corrected Free Gibbs Energy, ad from Table S6.

Table S6. Summary of DFT calculated energies. IEFPCM (acetonitrile) B3LYP/6-311G(d,p) level.

Conf.	H° (a.u.)	ΔH° (kcal/mol)	Pop. %	G° (a.u.)	ΔG° (kcal/mol)	Pop. %
**c5**	-925.417351	0.02	29	-925.489119	0.24	20
**c7**	-925.415839	0.96	6	-925.487181	1.45	3
**c8**	-925.416793	0.37	16	-925.489336	0.10	25
**c11**	-925.416477	0.56	12	-925.488705	0.50	13

**c21**	-925.415850	0.96	6	-925.488409	0.68	9
**c5b**	-925.414590	1.75	1	-925.485949	2.23	<1
**c8b**	-925.417376	**0.00**	30	-925.489495	**0.00**	29
**c21b**	-925.412556	3.02	0	-925.485083	2.77	<1

Table S7. Summary of the geometrical parameters of the best 8 conformations of **3aa**

Conf.	Cycle shape	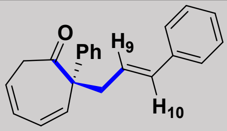	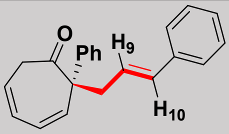	*pro-R* dihedral H_9-H_8
**c5**	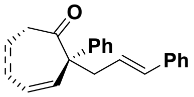	-66	-122	Gauche
**c7**	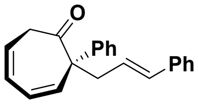	-171	-122	Gauche
**c8**	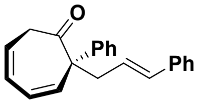	69	-117	Gauche
**c11**	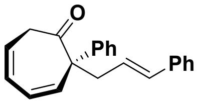	-173	122	Anti
**c21**	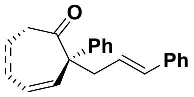	68	121	Anti
**c5b**	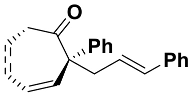	-73	116	Anti
**c8b**	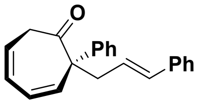	66	122	Anti
**c21b**	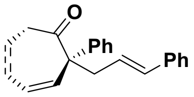	61	-145	Gauche

The structural differences among the 8 conformations are due to the different shape of the seven-membered ring, to the Cq-CH_2 bond rotation (C_7-C_8 bond, CO-Cq-CH_2-CH dihedral angle in Table S7), and to the CH_2-CH=CH rotation (C_8-C_9 bond, Cq-CH_2-CH=CH dihedral in Table S7). Two conformations are quite high in energy (**c5b** and **c21b**) with respect to the global minimum, and will not be further taken into consideration for the following discussion.

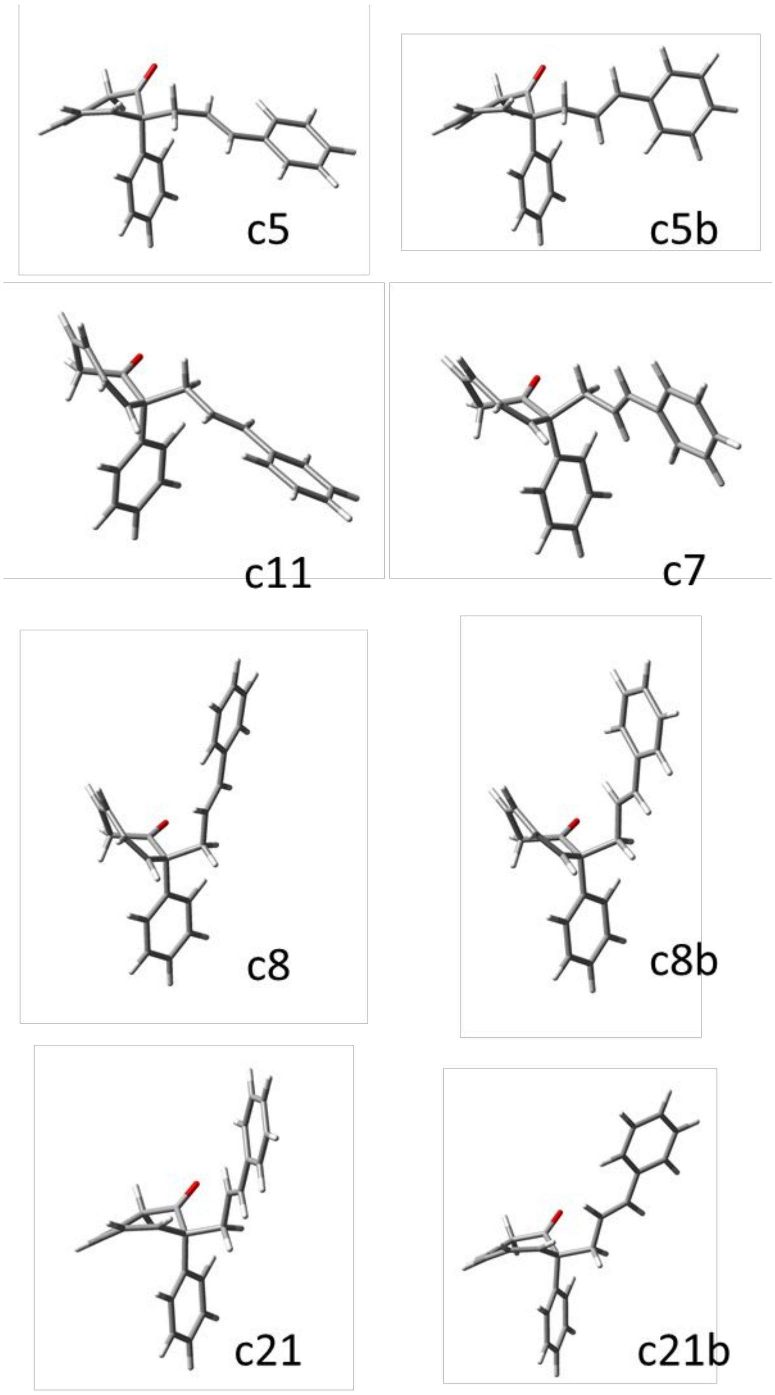

Figure S5. 3D structures of the most stable conformers of compound **3aa**. Optimization at the IEFPCM(acetonitrile) B3LYP/6-311G(d,p) level.

NMR spectroscopy was therefore used to check the conformational preferences suggested by DFT. Full assignment of the ^{1}H and ^{13}C NMR spectra was achieved by standard 2D-NMR analysis and J-coupling analysis of a CD_3CN sample of **3aa**. The signal of H_9 was assigned at 5.94 ppm by COSY, partially overlapped with H_3, and the two diastereotopic H_8' and H_8" were assigned at 3.06 and 2.80 ppm. The ^{3}J coupling constants of H_9 with H_8' and H_8" C_8 were measured as 7.4 Hz for both hydrogens (16.0 Hz the H_9-H_{10} ^{3}J). Since in all the four

conformations **c7**, **c8**, **c8b**, **c11** one of the diastereotopic hydrogens is anti to H_9 (≈180° dihedral angle) and the second diastereotopic hydrogen is gauche (≈ 60°), the two coupling constants should be different. The experimental value suggests that a mixture of conformations exists, with averaging of the two H_9-H_8' and H_9-H_8" coupling constant values. NOE-NMR spectra were then acquired in order to further investigate the conformational preferences. On saturating the signal at 5.94 ppm, nearly identical NOE enhancements were observed on both H_8 hydrogens (Figure S6), thus confirming the conformational averaging of **c11**/**c7** and **c8**/**c8b**, in such a way to yield averaged similar distances between H_{10} and H_8'/H_8''.

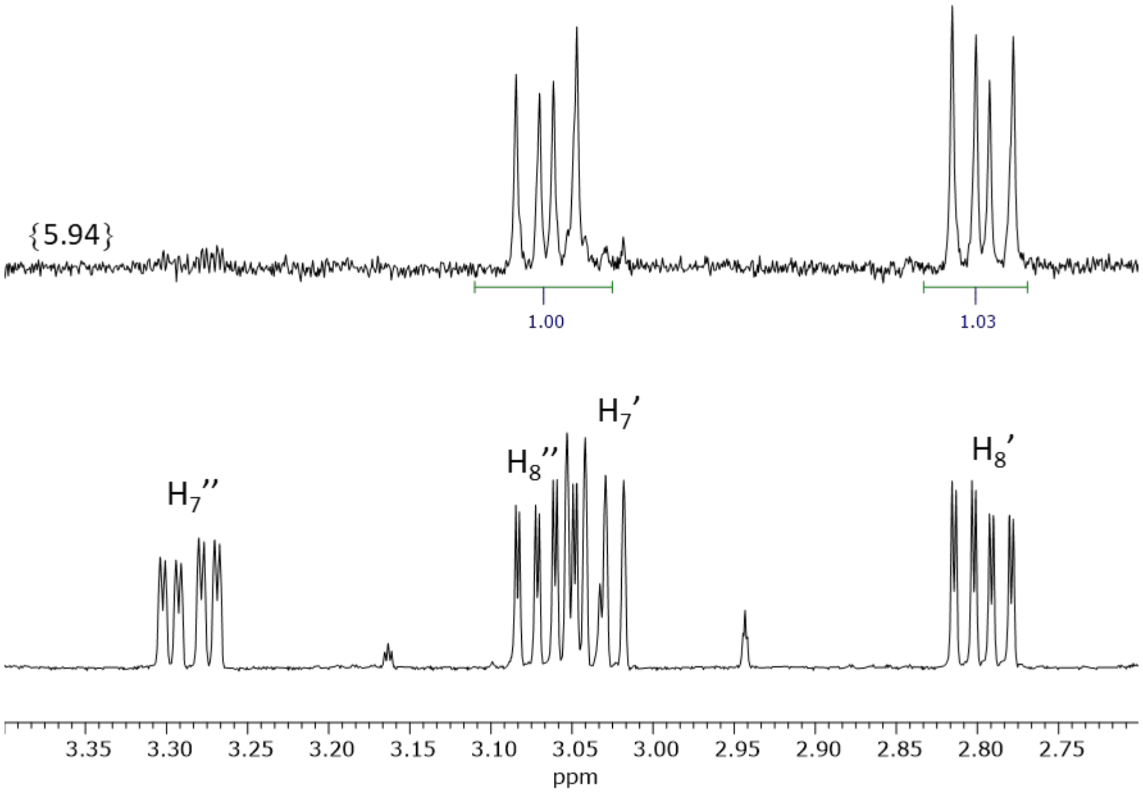

Figure S6. Bottom: aliphatic portion of ^{1}H NMR spectrum of **3aa** (600 MHz in CD_3CN), showing the four diastereotopic hydrogens on C_7 and C_8. Top: DPFGSE-NOE on saturation of the H_{10} signal at 5.94 ppm (50 Hz R-SNOB selective pulse was used).

The experimental data are well supported when considering the optimized energies form Table S6. If considering the *pro-R* hydrogen on C8, the H9 vinyl hydrogen is *anti* in conformations **c5b**, **c8b**, **c11** and **c21**, whereas it is *gauche* in conformations **c5**, **c7**, **c8** and **c21b**. Taking into considerations the sum of the respective populations, the anti/gauche ratio is 49:51 when using ΔH°, and 43:57 when using ΔG°.

6.2 Assignment of the Absolute configuration

The theoretical simulation of the electronic circular dichroism spectra (ECD) by TD-DFT was selected for the absolute configuration assignment of **3aa** because of the presence of strong UV chromophores.[10] The ECD spectrum was acquired on a JASCO J-810 spectropolarimeter in LC-MS grade acetonitrile solution (about $1\cdot10^{-4}$ M) with a cell path of 0.1 cm in the 190-400 nm region by the sum of 16 scans at 50 nm/min scan rate (Figure S7). The ECD spectrum showed four Cotton effects at 300, 261, 232 and 205 nm, being the 261 nm positive cotton effect corresponding to the first strong UV absorption.

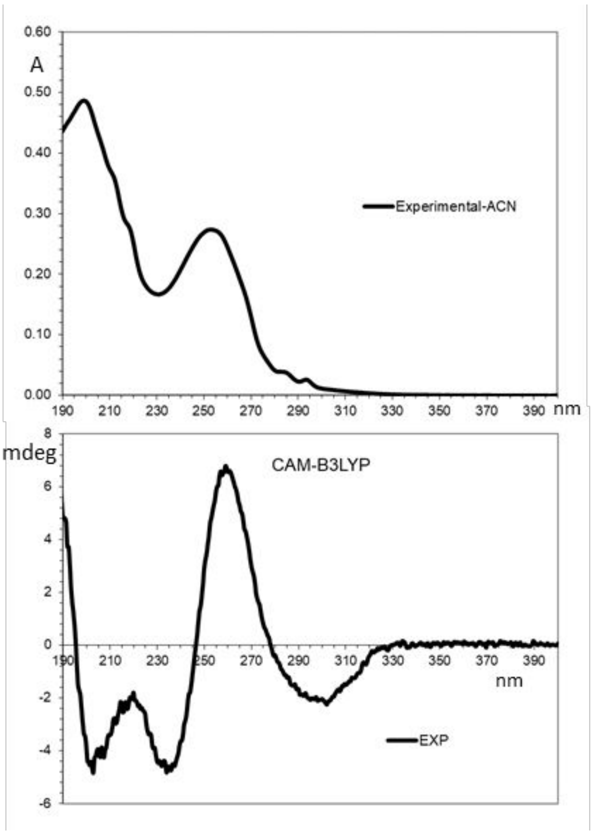

Figure S7. UV (top) and ECD (bottom) spectra of compound **3aa** in acetonitrile, with 0.1 cm pathlength.

The TD-DFT simulations of the UV and ECD spectrum were performed using the geometries of the six conformations from Table S6. For data redundancy, calculations were performed with the hybrid functional M06-2X,[11] with ωB97XD that includes empirical dispersion,[12] and with CAM-B3LYP[13] that includes long range correction. The calculations employed the 6-311++G(2d,p) basis set, that is known to yield good performances at a reasonable computational cost.[14] For each conformation, 40 transitions were calculated in order to cover the 400-150 nm range.

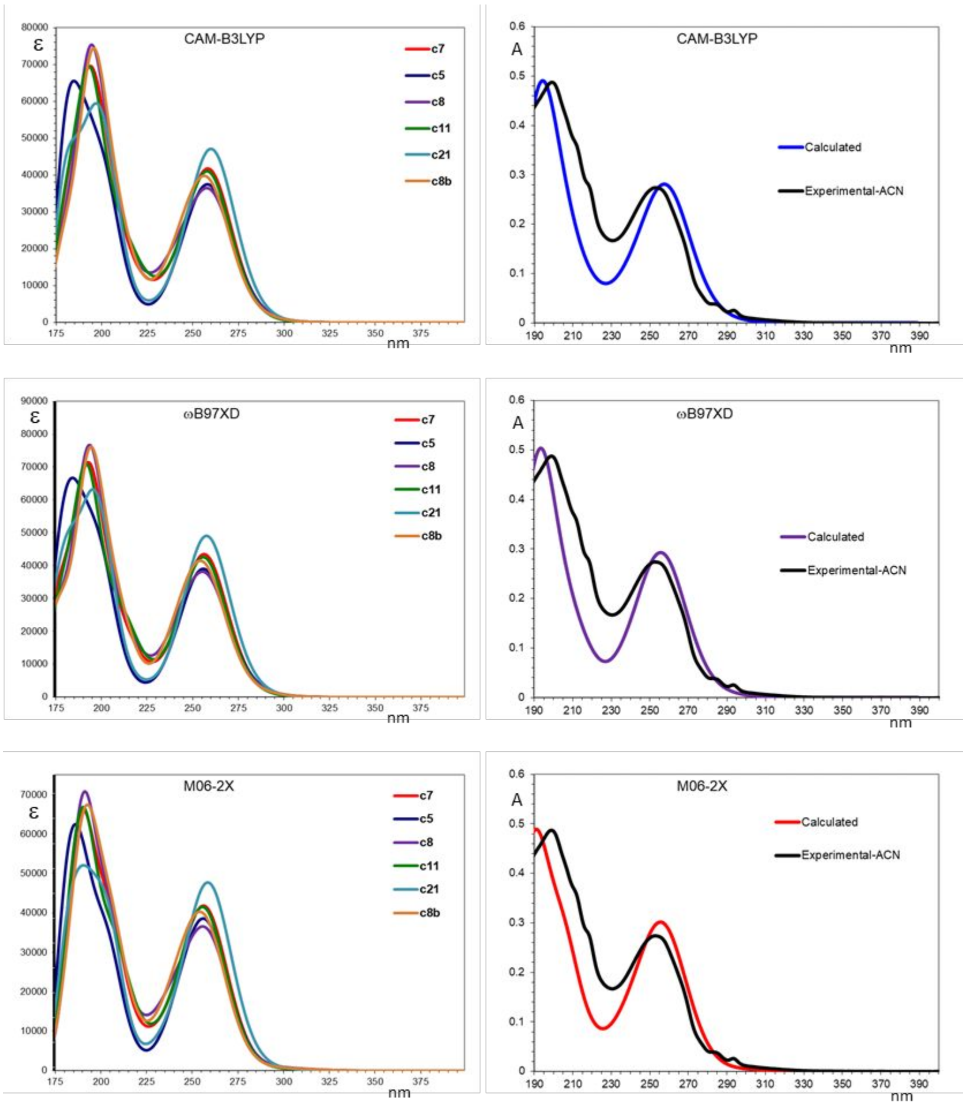

Figure S8. Left: TD-DFT simulations for the six populated conformations of **3aa**, with three different functionals and the same 6-311++G(2d,p) basis set. Right: comparison of the Bolzmann-averaged simulation from Table S6 (ΔH°) and the experimental UV spectrum. The vertical scale of the simulated spectra were scaled in order to match the experimental intensity. No red-shift was applied to the simulations to match the experimental ECD spectrum.

The rotational strengths were calculated in both length and velocity representation, obtaining similar results (RMS difference < 5%) that ruled out large basis set incompleteness errors (BSSE).[15] The *S* absolute configuration of **3aa** was arbitrarily chosen for the calculations. The Boltzmann averaged spectra were then obtained using the ΔH° relative energies from Table S6. The agreement with the experimental UV and ECD spectra is very good (Figures S8 and S9), and the S absolute configuration can be reliably assigned to compound **3aa**.

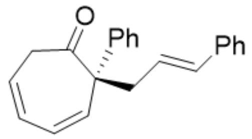

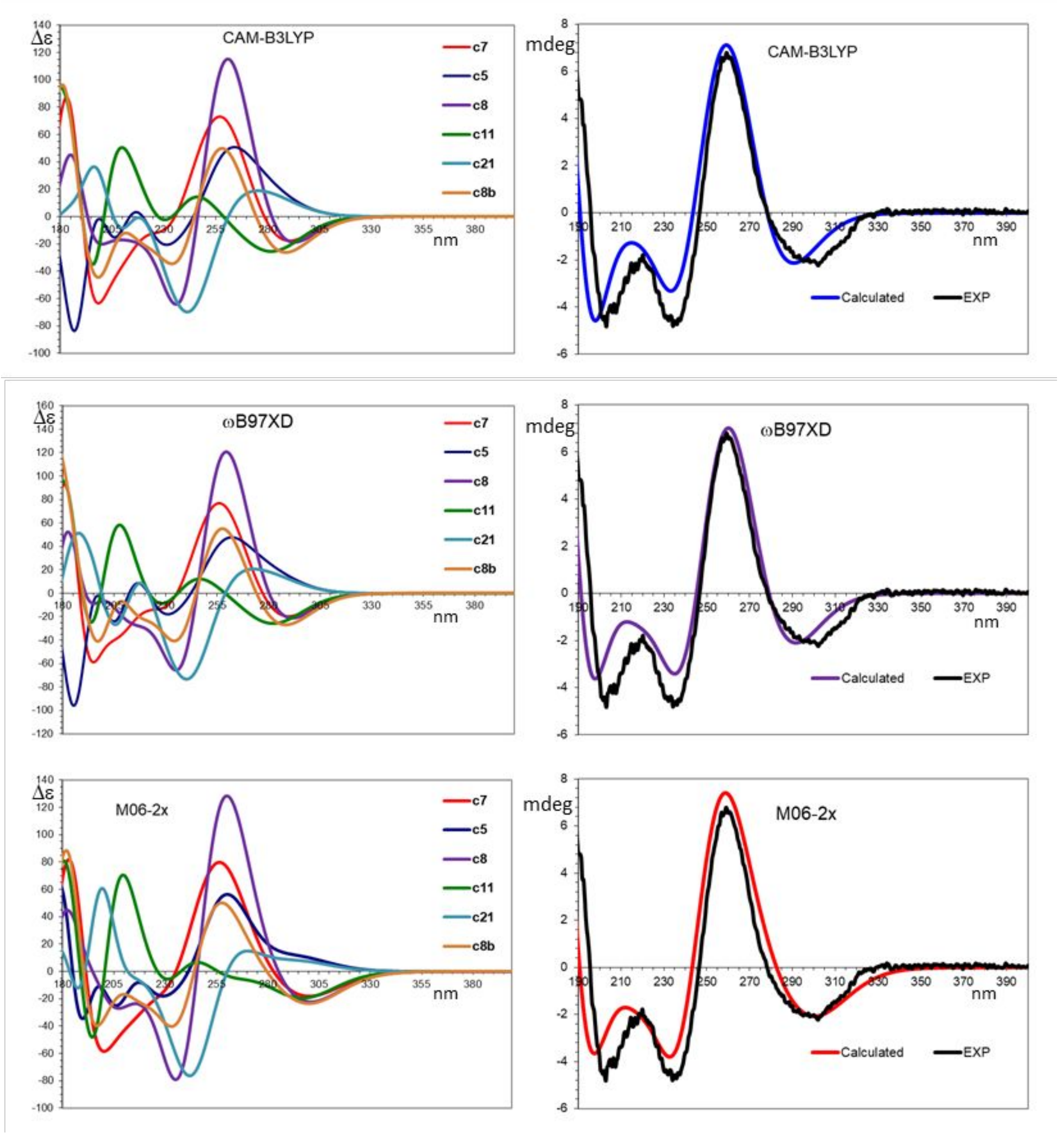

Figure S9. Left: TD-DFT simulations for the six populated conformations of **3aa**, with three different functionals and the same 6-311++G(2d,p) basis set. Right: comparison of the Bolzmann-averaged simulation from Table S6 (ΔH°) and the experimental ECD spectrum. The vertical scale of the simulates spectra were scaled in order to match the experimental intensity. No red-shift was applied to the simulations to match the experimental ECD spectrum.

7. Voltametric Analysis

Cyclic voltammetry (CV) experiments were performed in a 15 mL vial using an Autolab PGSTAT302N potentiostat (Metrohm), with data acquisition controlled by the Nova 2.1.8 software. A total of 15 mg of **1a** or pre-formed [Pd(η^3-allyl)(PPh_3)$_2$](BF_4)[16] complex were dissolved in 7 mL of acetonitrile (CH_3CN, 0.02 mol L^{-1}). Tetraethylammonium tetrafluoroborate ($TEABF_4$) at a concentration of 0.1 mol L^{-1} was employed as the supporting electrolyte. The measurements were conducted at room temperature at a scan rate of 50 mV s^{-1} using a glassy carbon disk working electrode (3 mm diameter) and a platinum counter electrode (GC||Pt). A silver wire was used as the reference electrode, and all potentials were internally referenced to the ferrocene/ferrocenium (Fc/Fc^+) redox couple. The use of [Pd(η^3-allyl)(PPh_3)$_2$](BF_4) aims to simulate the active Pd-species that is formed after reduction of the Pd(II) pre-complex and oxidative addition to allyl acetate. The choice of PPh_3 ligand (instead of **L1**), and an allyl group instead of a cinnamyl unit, was made for the sake of simplicity; these conditions however simulate well the real catalytic process, as the reaction proceeds with the same chemo- and regioselectivity both in the presence of PPh_3 (preparation of racemic products **3**) and allyl acetate (product **3an**). The use of ACN as solvent was due to solubility issues of [Pd(η^3-allyl)(PPh_3)$_2$](BF_4) in THF, leading to poor resolution spectra in this solvent. However, the reaction proceeds with the same selectivity and in high yields in ACN as well (main text, Table 1 entries 1-4).

Product **1a** shows an irreverisble reduction peak at E_p = -1.80 V *vs* Fc/Fc^+ while [Pd(allyl)(PPh_3)](BF_4) shows an irreversible reduction peak at -2.42 V *vs* Fc/Fc^+.

<u>Note A</u>: The working electrode was polished by brushing with diamond paste on a polyester cloth.

<u>Note B</u>: The solution was de-gassed by bubbling N_2 for 1 min prior to analysis. The N_2 flow was continued during the analysis to prevent oxygen contamination.

*<u>Note C</u>: Initial potential: -0.40 V vs Fc/Fc^+; direction of initial scan: reduction; switching potential: -2.2 V vs Fc/Fc^+ for **1a** and -2.5 V for [Pd(η^3-allyl)(PPh_3)$_2$](BF_4).*

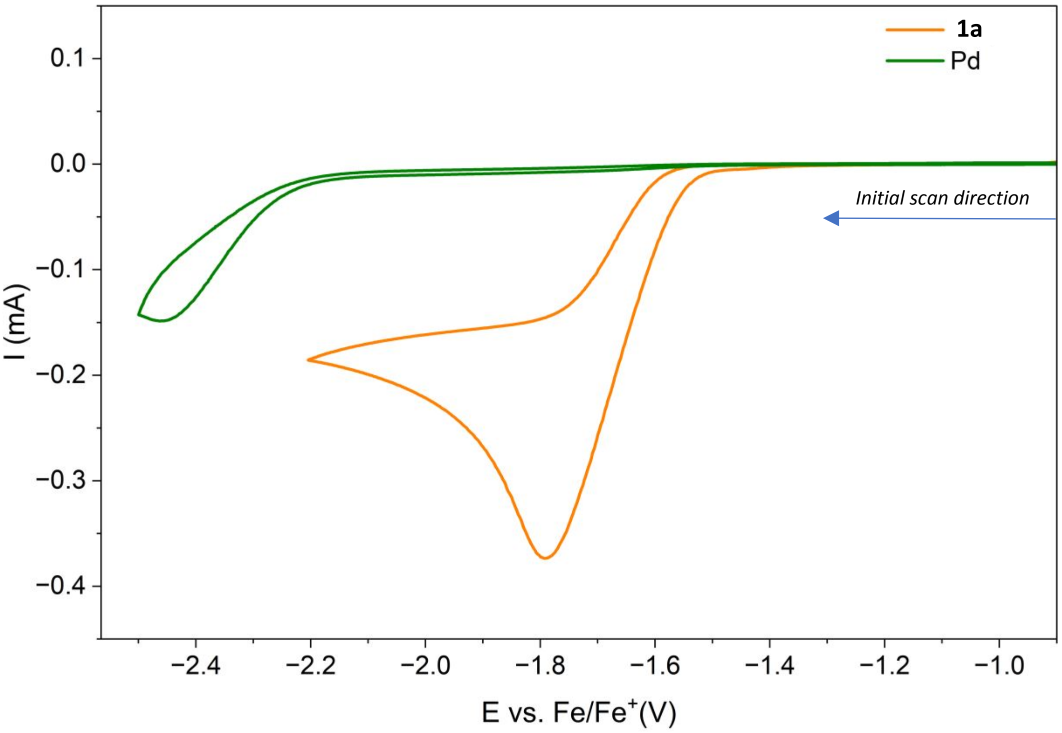

Figure S10. Voltametric responses plotted following the IUPAC convention.

8. Deuteration Experiment

By running General Procedure **B** in the presence of D_2O instead of H_2O, product ***d*-3aa** was isolated in 54% yield. Complete mono-deuteration α to the carbonyl moiety was observed. In particular, the signal at 3.30 ppm shows 65% deuteration and the one at 3.12 ppm (partially overlapped with one of the allylic CH signals) shows 35% deuteration, indicating that the incorporation of deuterium at this position occurs in a 65:35 diastereomeric ratio. These results suggests a final quenching by D_2O of the enolate intermediate leading to the desired product.

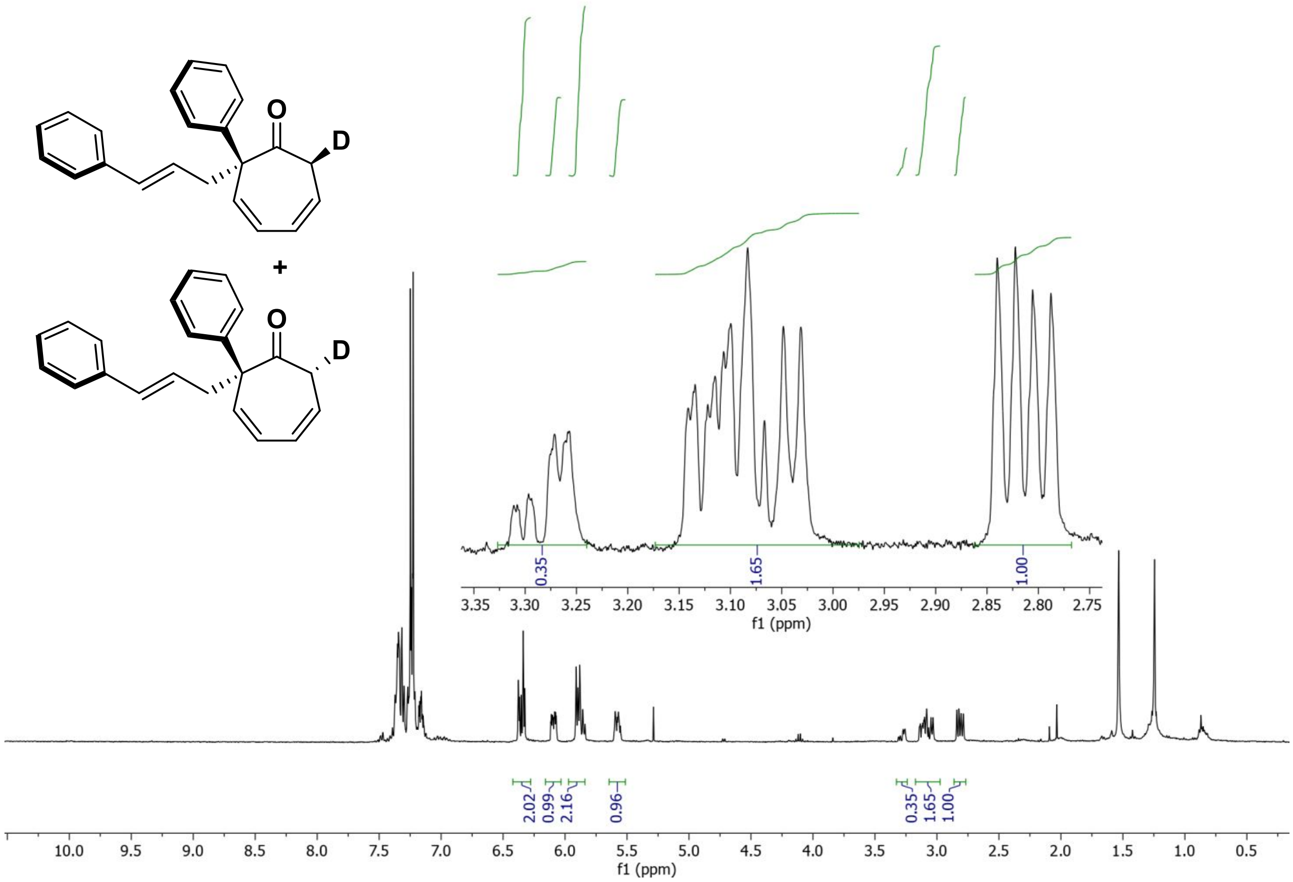

Figure S11. ^1H NMR (400 MHz, $CDCl_3$) of ***d*-3aa** and zoom of the relevant region.

9. Additional Computational Details

9.1 Inner vs Outer Sphere Mechanism

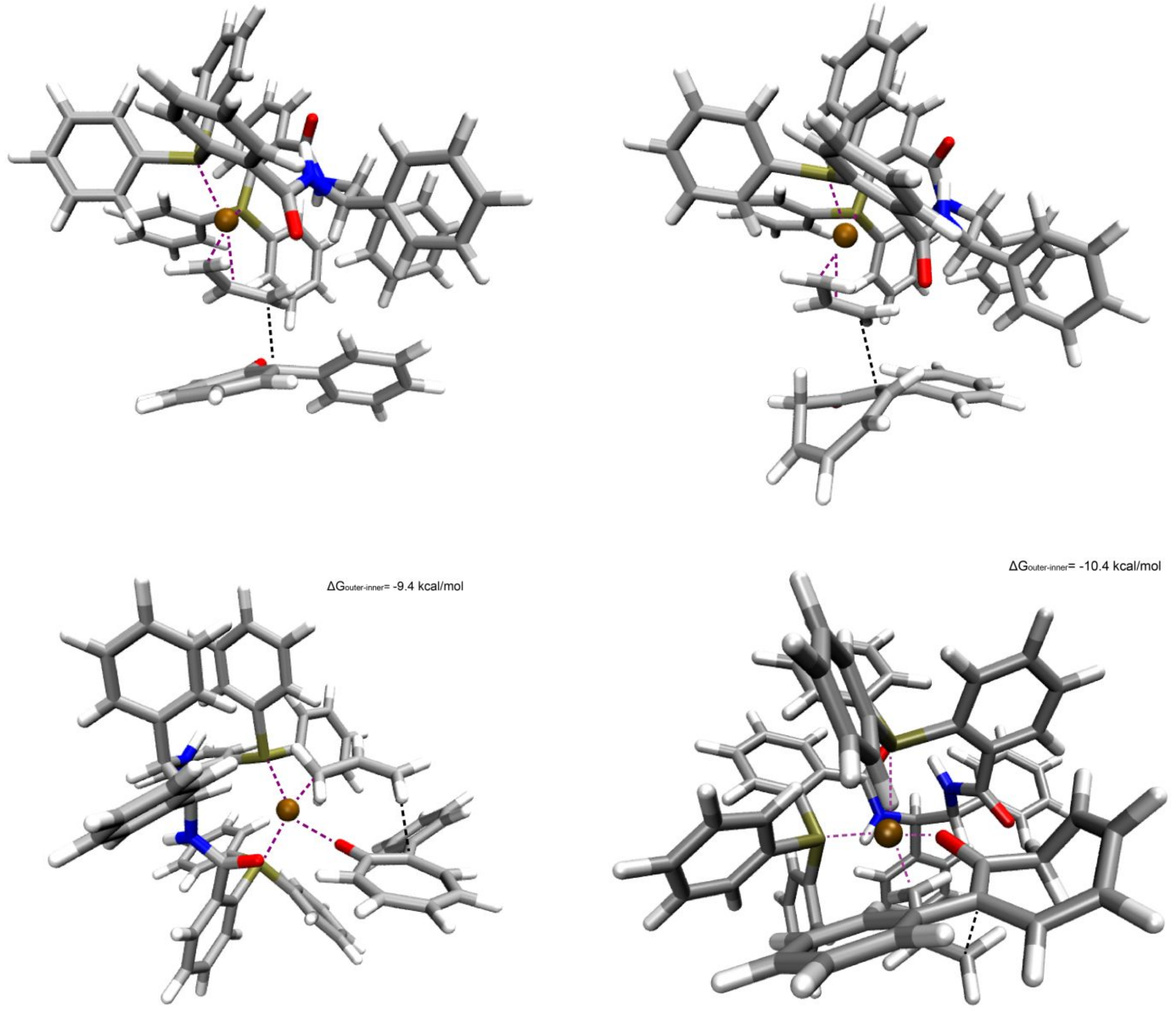

Figure S12. Comparison of the TS structures for outer (top) and inner (bottom) sphere mechanisms for both the radical (left) and polar (right) pathways. The metal coordination is depicted with magenta dashed lines whereas the bond formation with black.

In the case of the outer sphere pathway, the allyl is connected through an η^2-bond with Pd, which, in the case of inner sphere gives its place to a simple η^1-bond, in order to accommodate the oxygen of the tropone. The outer sphere TS is lower in energy for 9.4 kcal/mol for the radical and 10.4 kcal/mol for the polar mechanism.

9.2 Rationalization of the Enantioselectivity

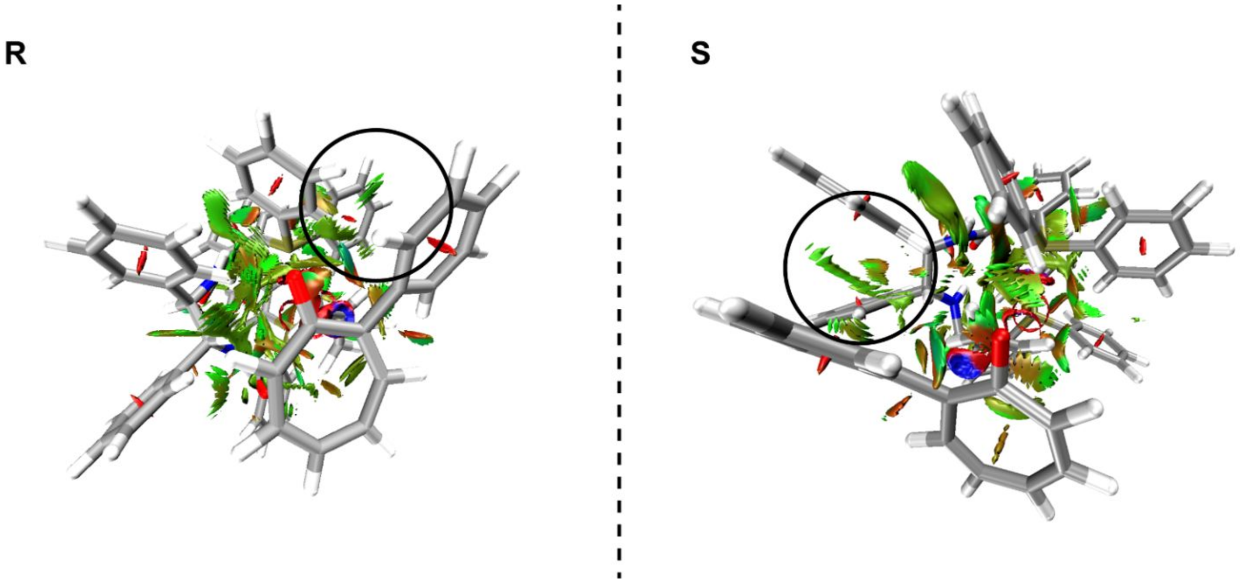

Figure S13. NCI plots of the TS to-D for the *R* and the *S* enantiomer.

The NCI analysis, also known as RDG analysis, is a method developed by Yang and coworkers,[17] that uses the electron density and its derivatives to detect non-covalent interactions in real space. In this work, the NCI were calculated and visualized using the r^{2}SCAN/def2-SVP(P)/CPCM wavefunction and the corresponding function of MultiWfn.[18] For better visualization, VMD[19] was used with the RDGfill.vmd script provided with the MultiWfn software. The NCI plot indicates that in the case of the *S*-enantiomer there are favourable non-covalent interactions between the aromatic rings of the DPPBA (2-(diphenylphosphino) benzoic acid) scaffold and the phenyl substituent of the tropone (right panel, in black circle) that in the case of the *R* enantiomer are almost absent (left panel, in black circle).

10. NMR Spectra

1i ^{1}H NMR (600 MHz, $CDCl_3$)

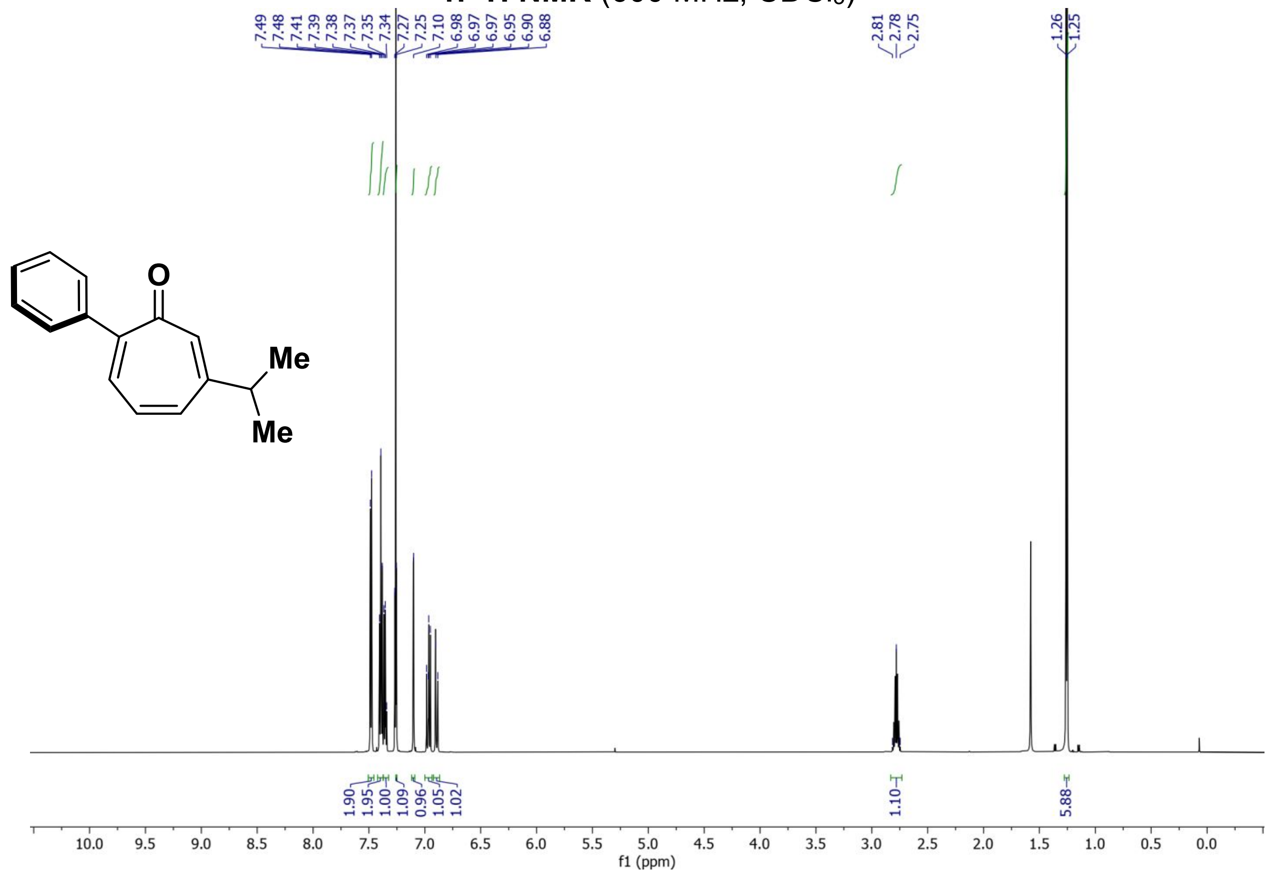

1i ^{13}C NMR (151 MHz, $CDCl_3$)

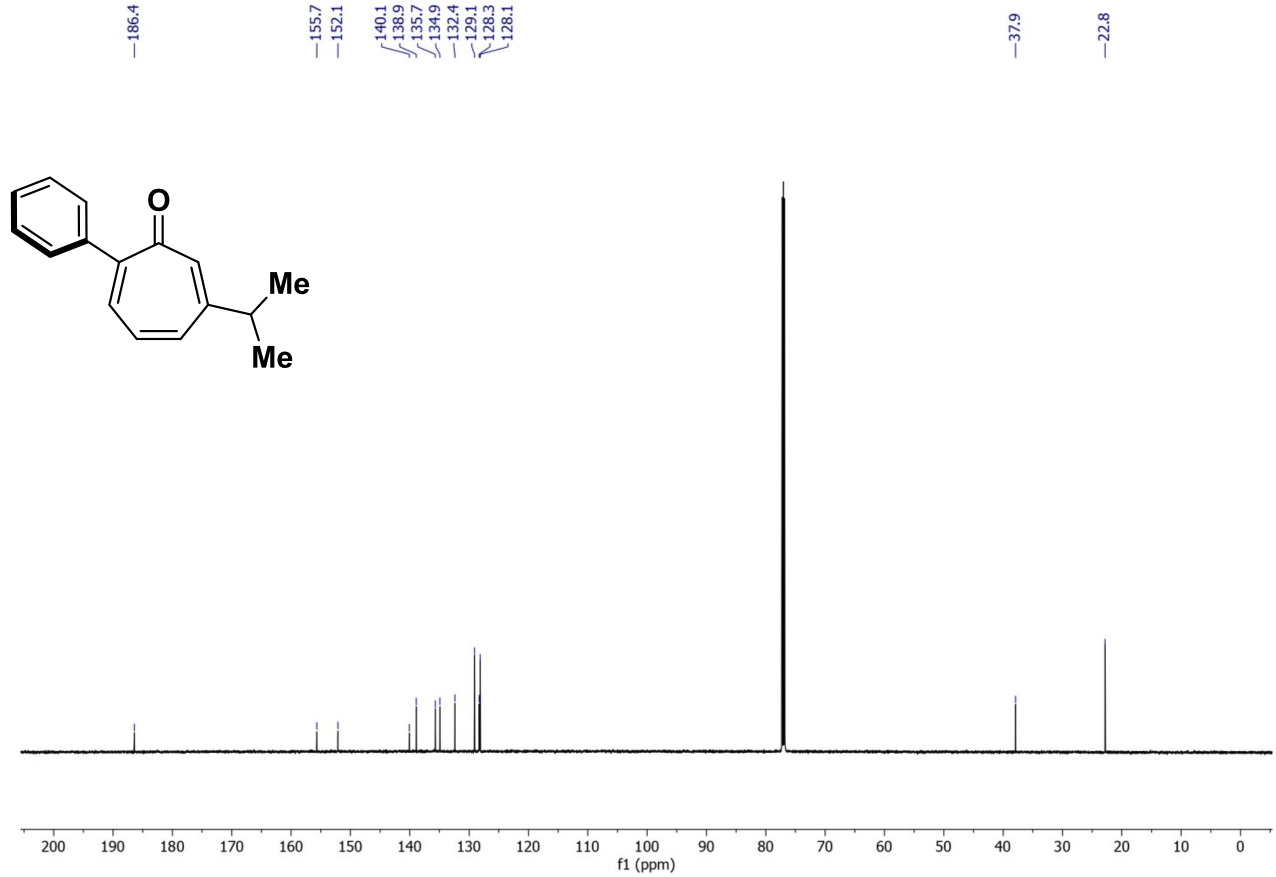

1j ^{1}H NMR (600 MHz, $CDCl_3$)

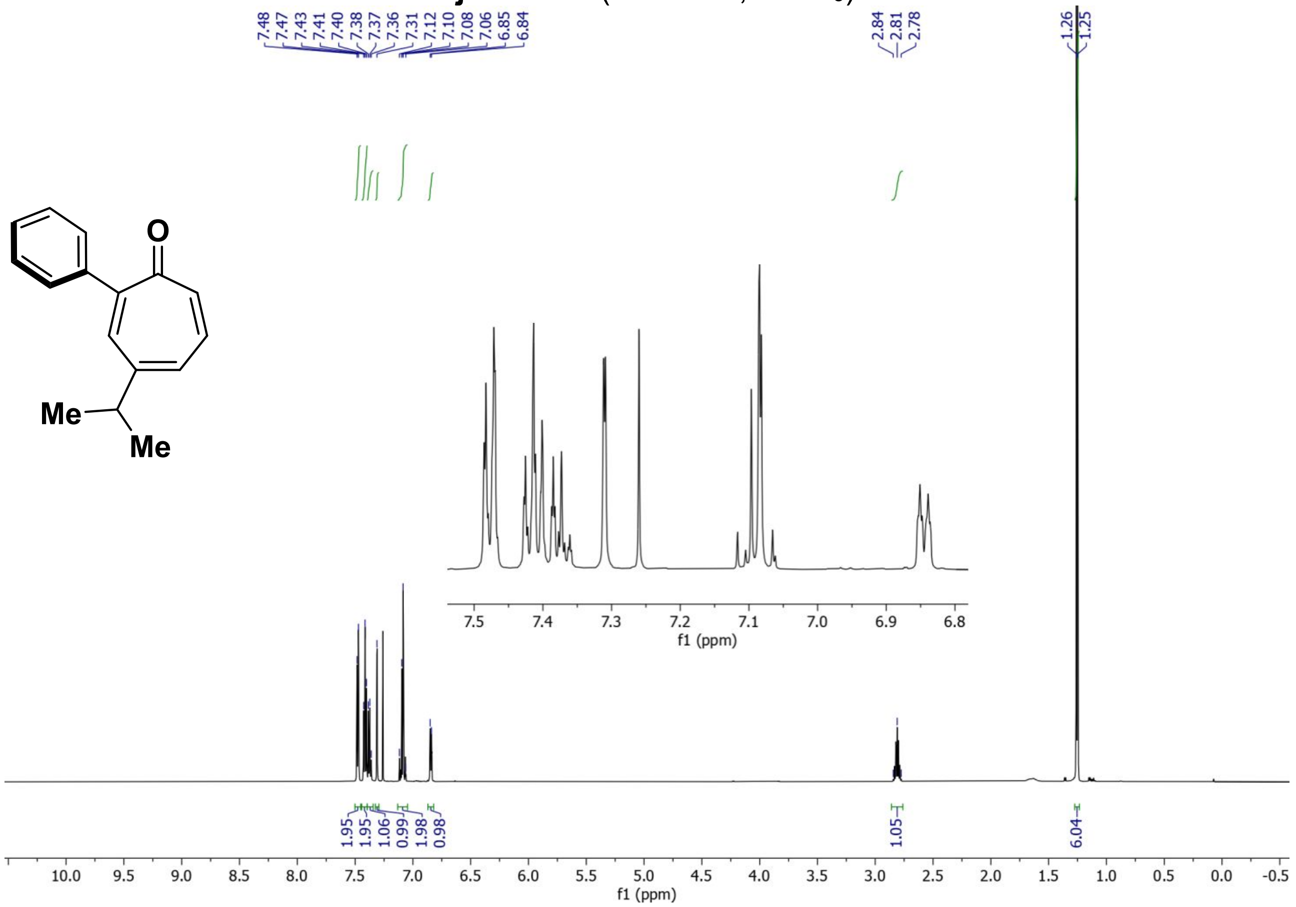

1j ^{13}C NMR (151 MHz, $CDCl_3$)

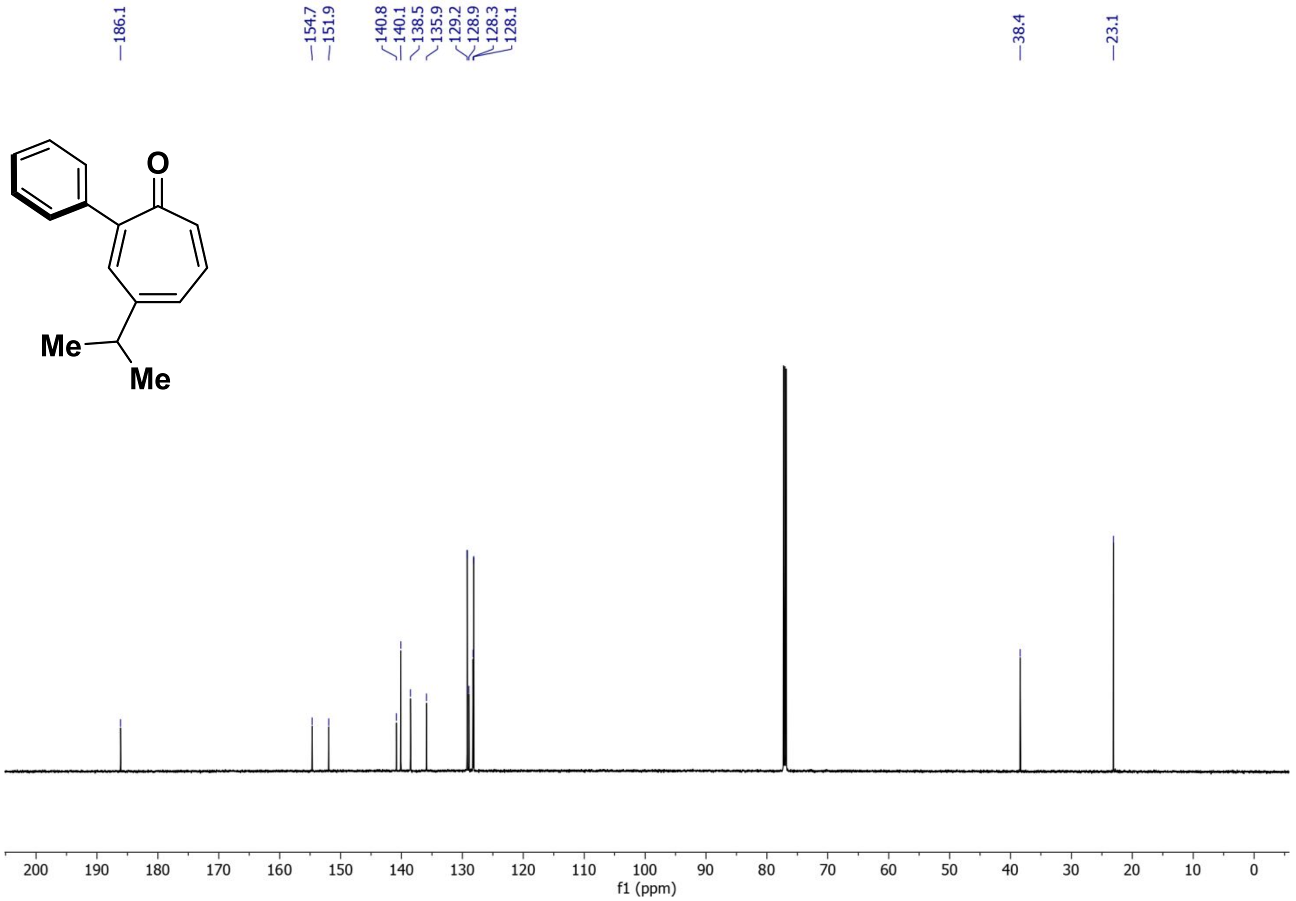

3aa ^{1}H NMR (600 MHz, $CDCl_3$)

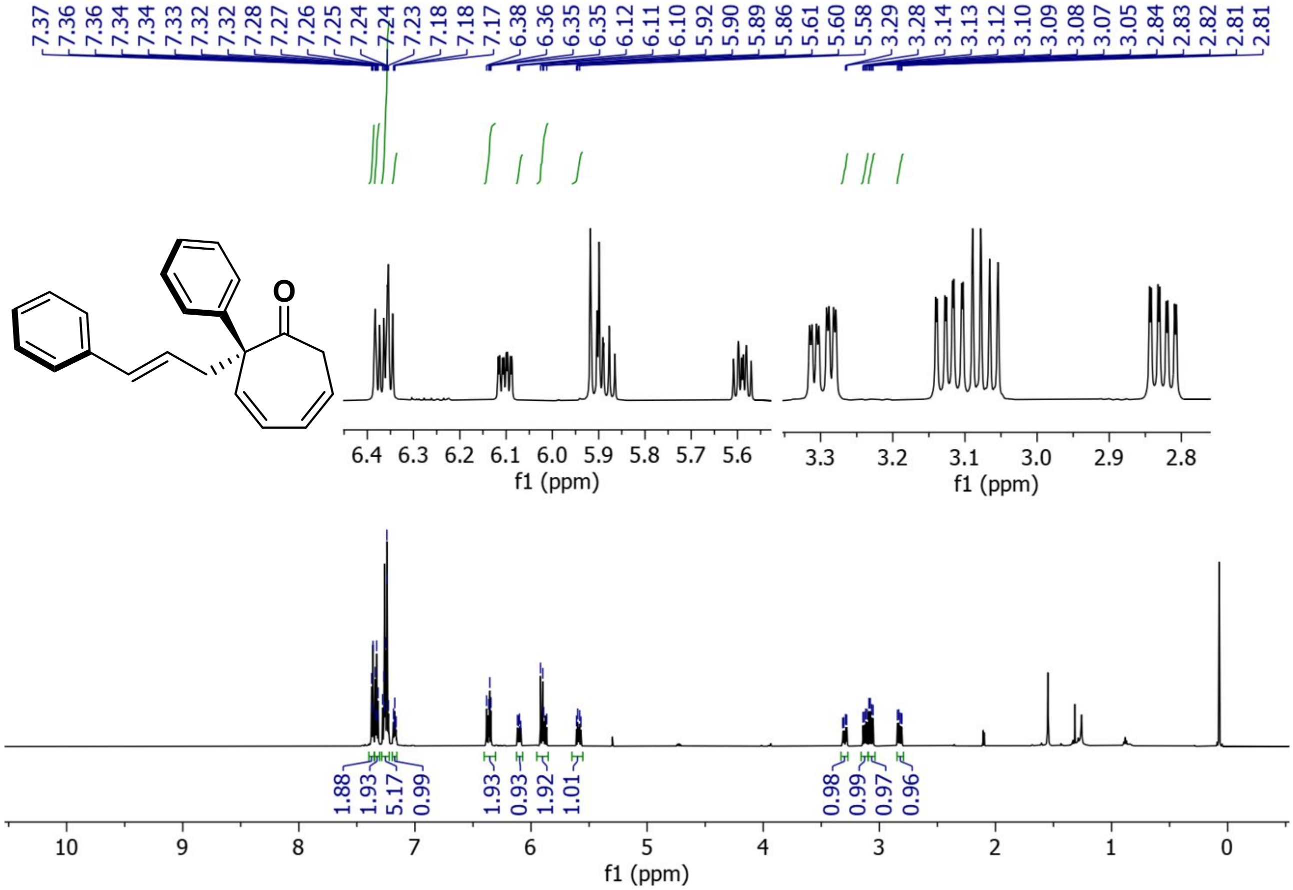

3aa ^{13}C NMR (151 MHz, $CDCl_3$)

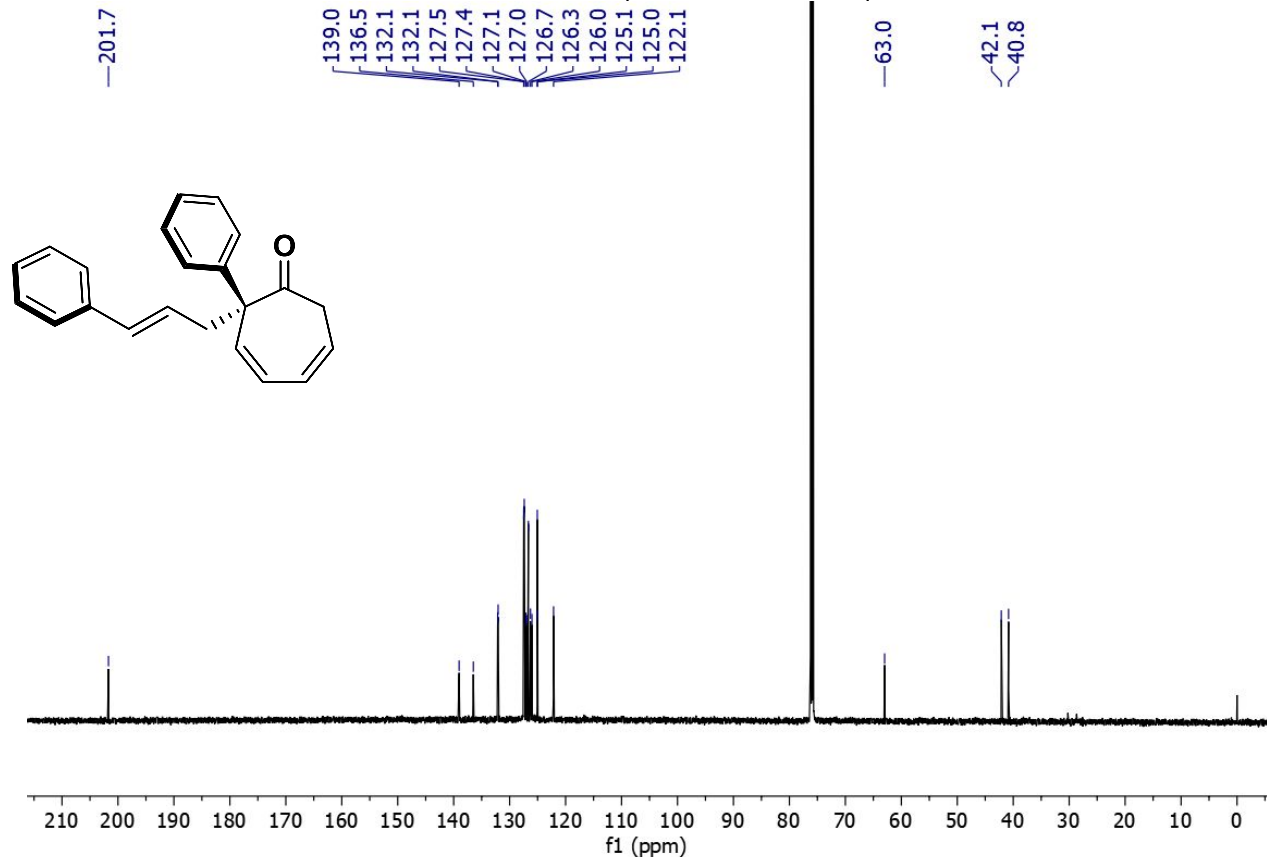

3ab ^{1}H NMR (600 MHz, $CDCl_3$)

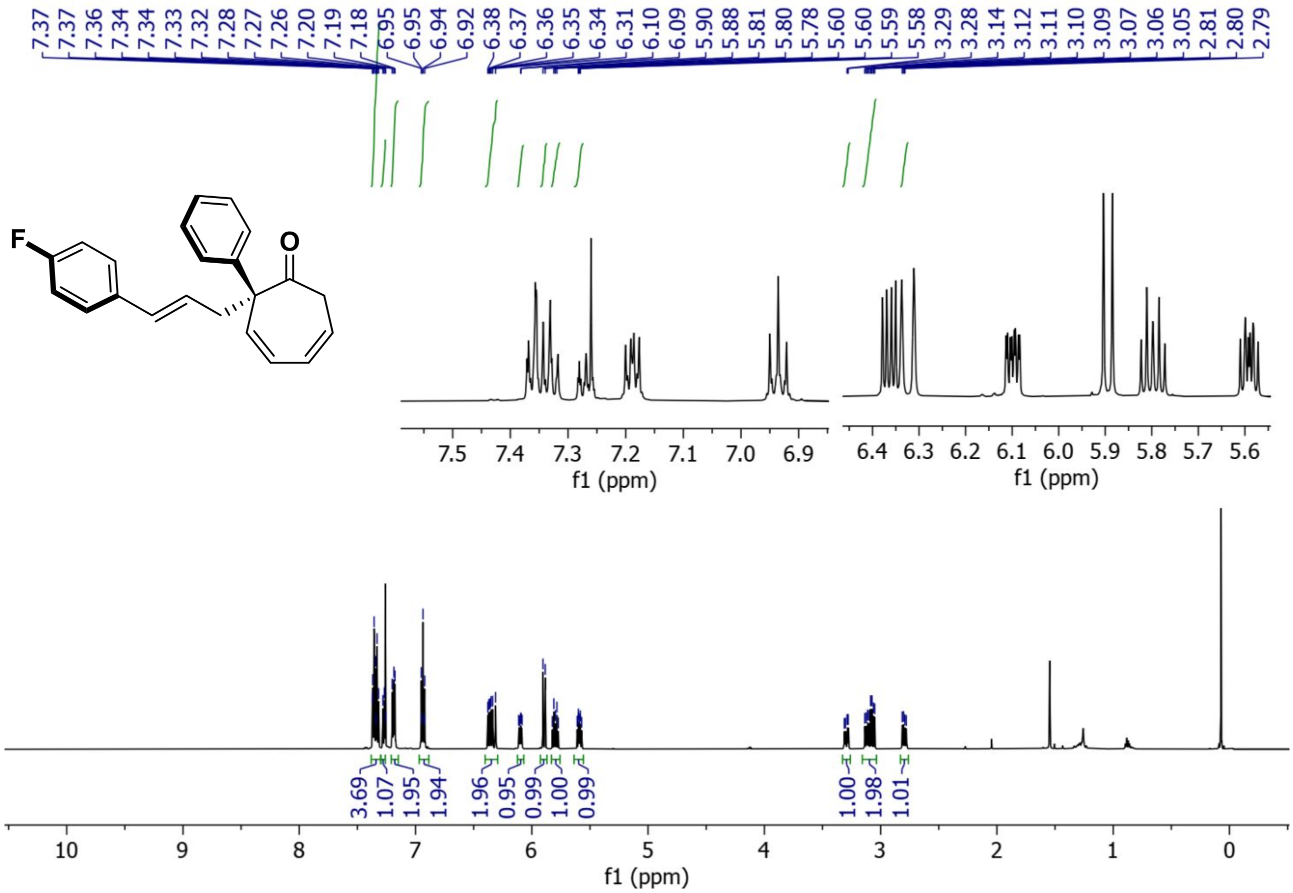

3ab ^{13}C NMR (151 MHz, $CDCl_3$)

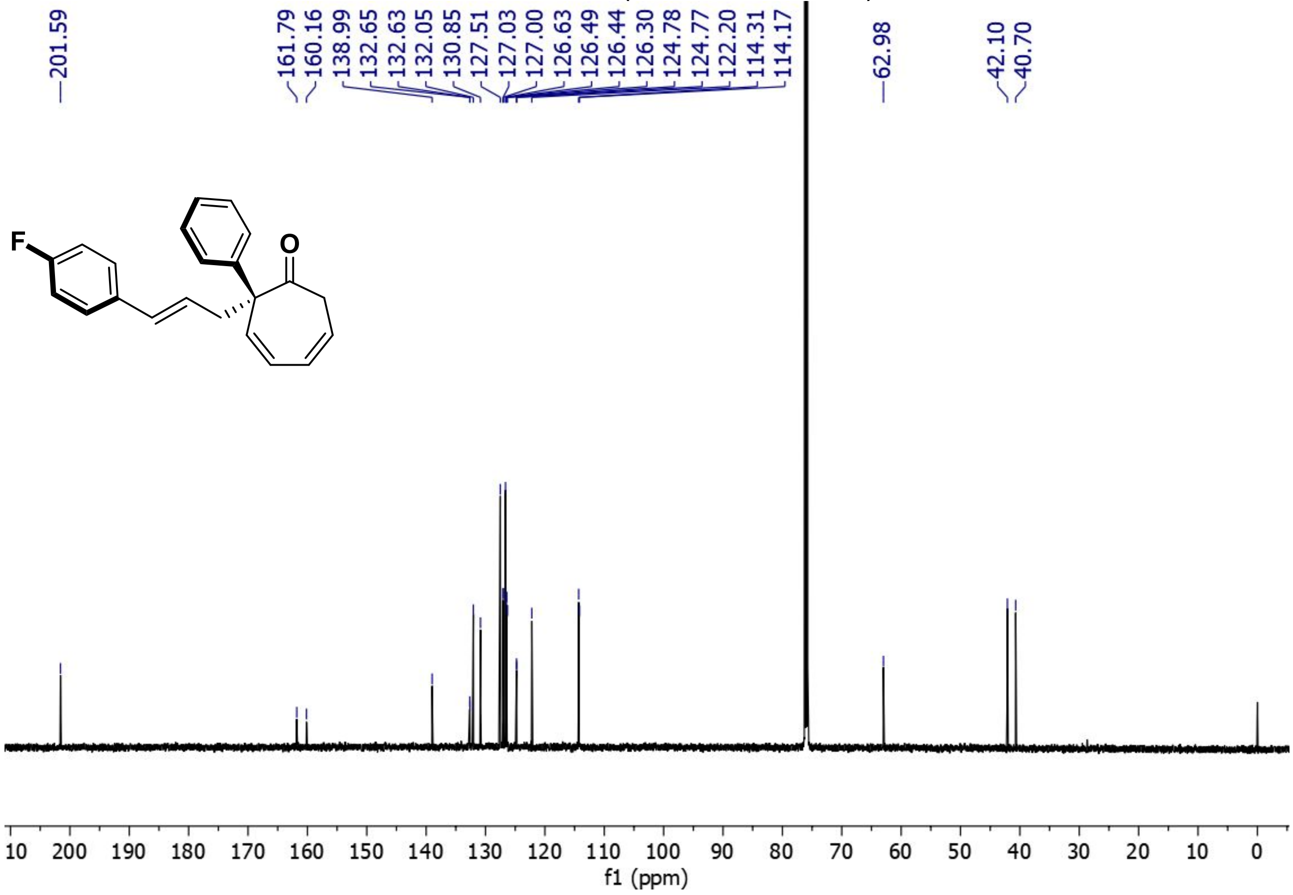

3ab ^{19}F NMR (576 MHz, CDCl$_3$)

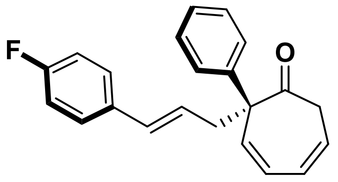

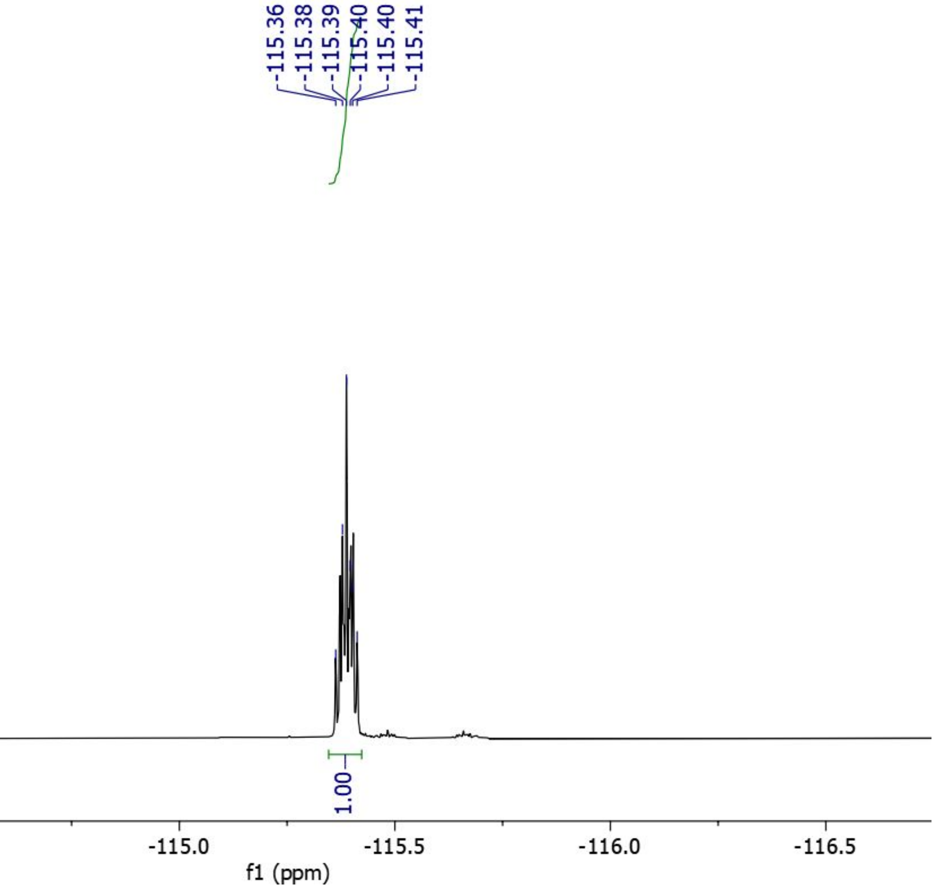

3ac ^{1}H NMR (600 MHz, CDCl$_3$)

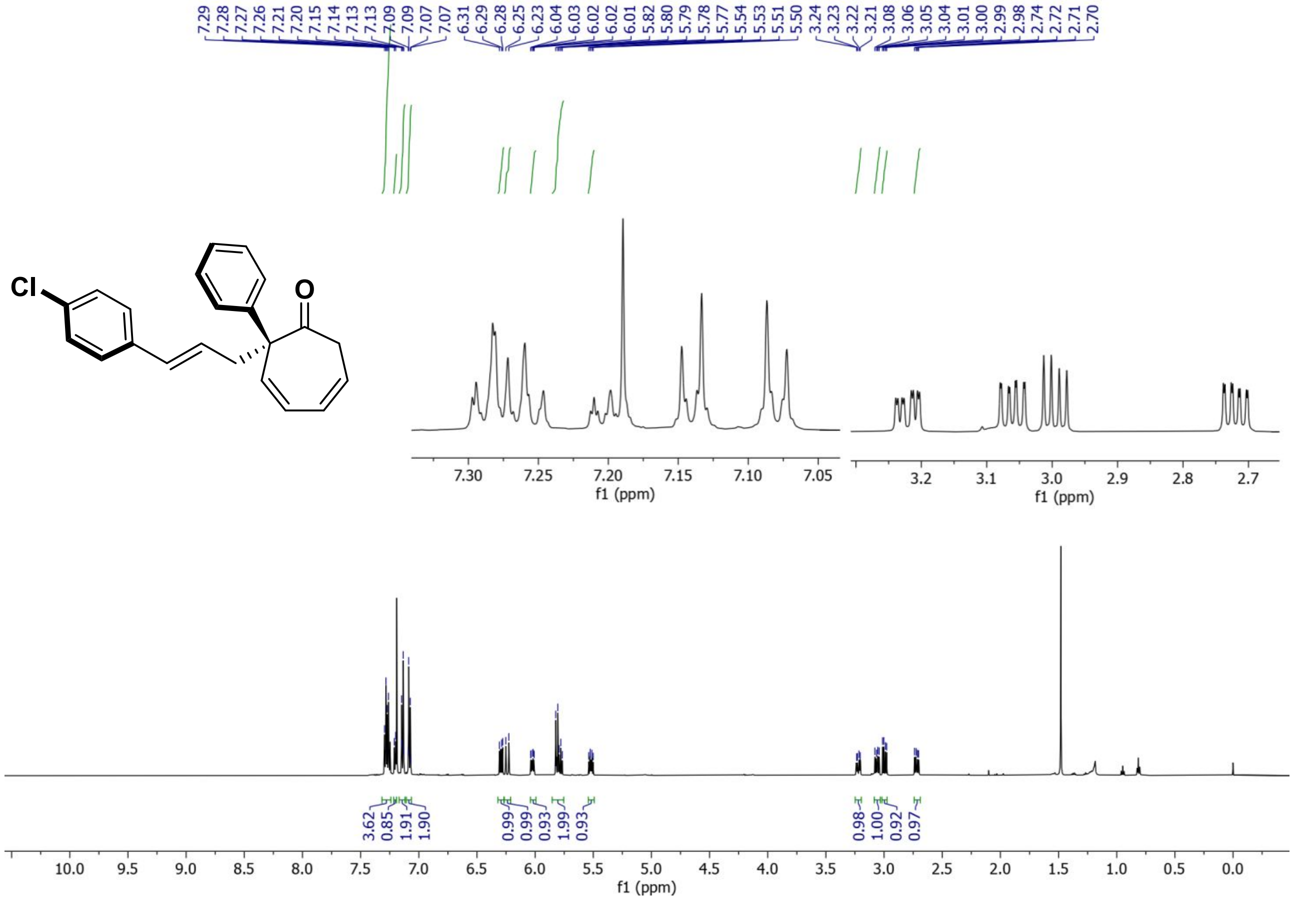

3ac ^{13}C NMR (151 MHz, CDCl$_3$)

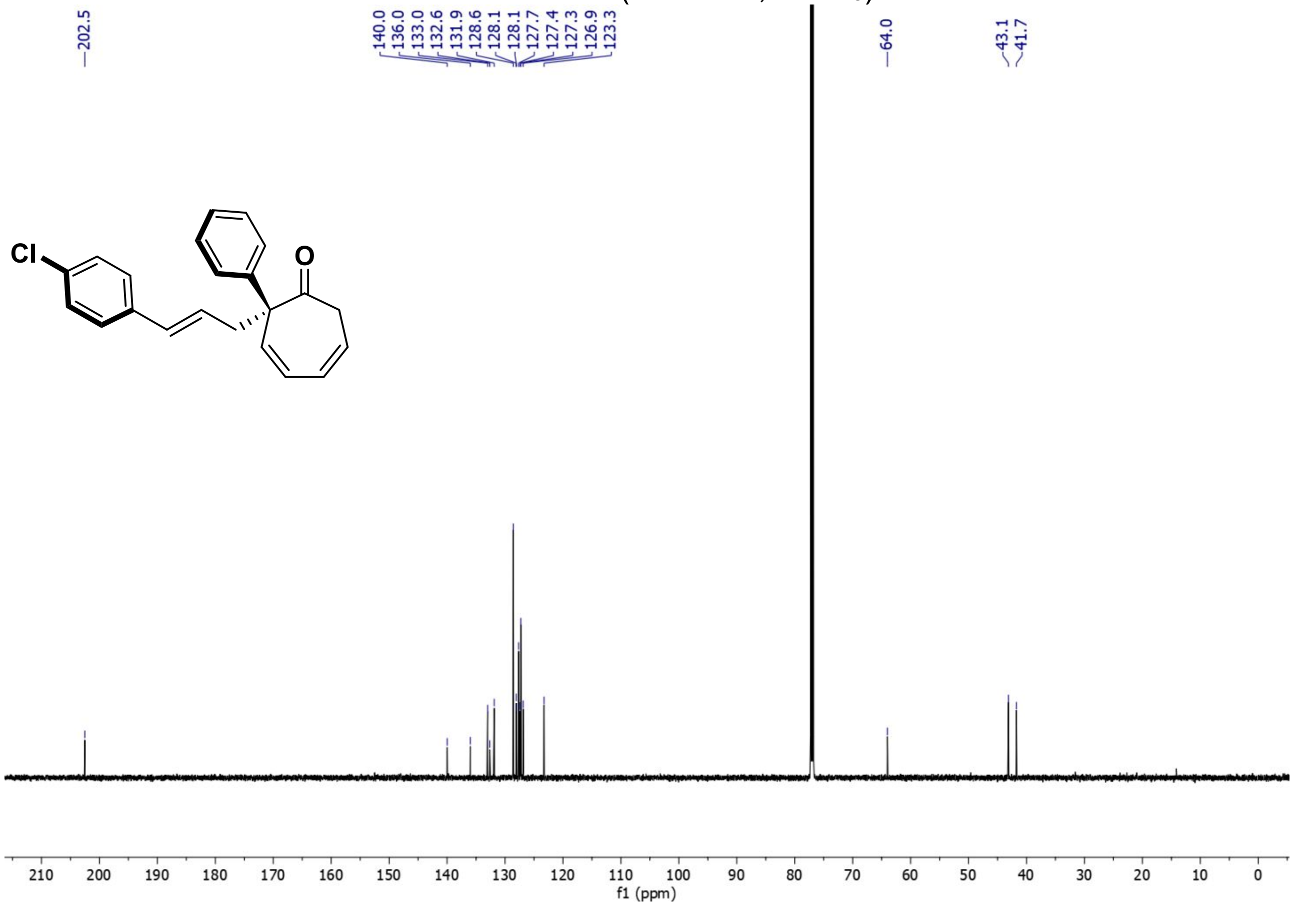

3ad ^{1}H NMR (600 MHz, $CDCl_3$)

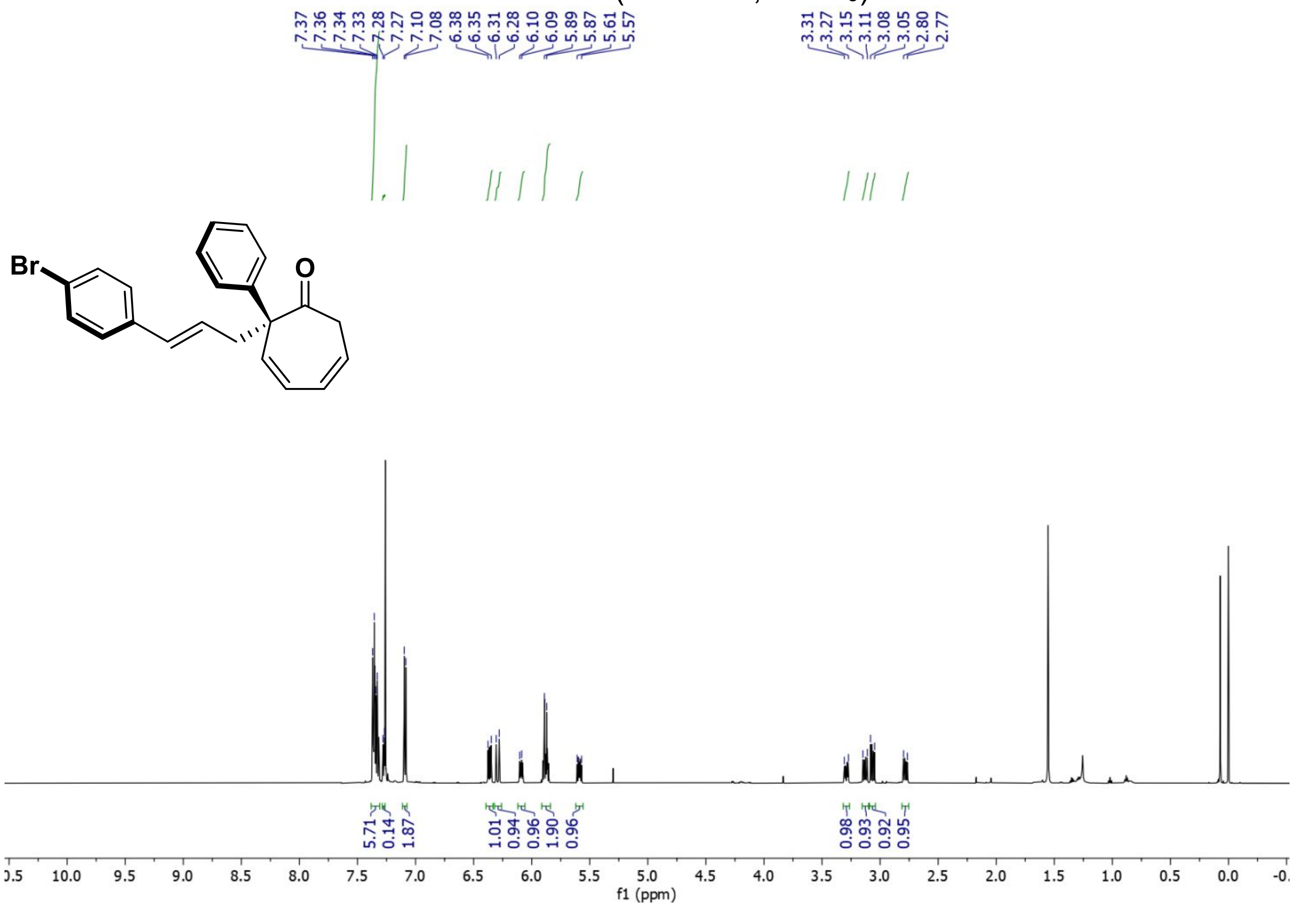

3ad ^{13}C NMR (151 MHz, $CDCl_3$)

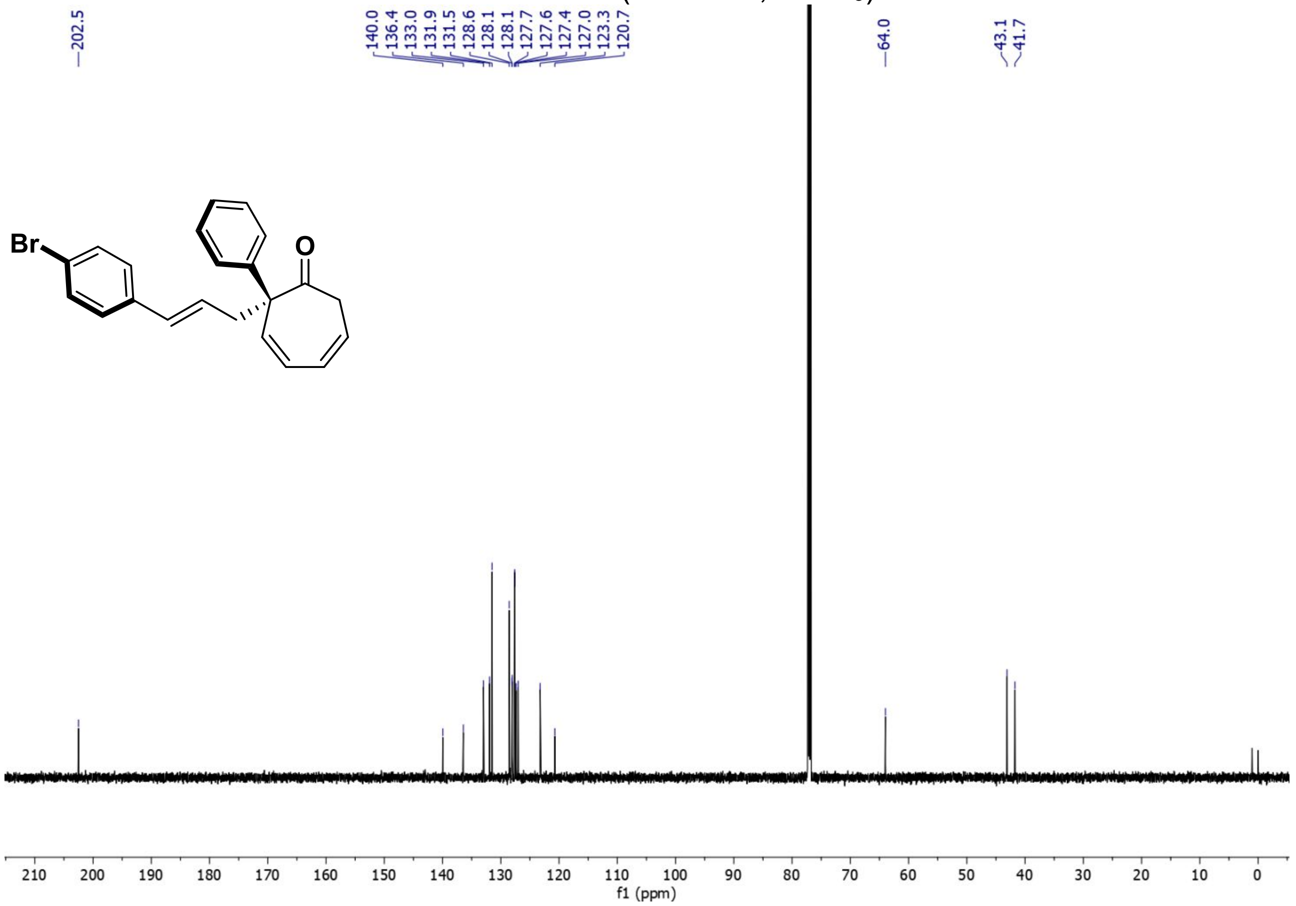

3ae ¹H NMR (600 MHz, CDCl$_3$)

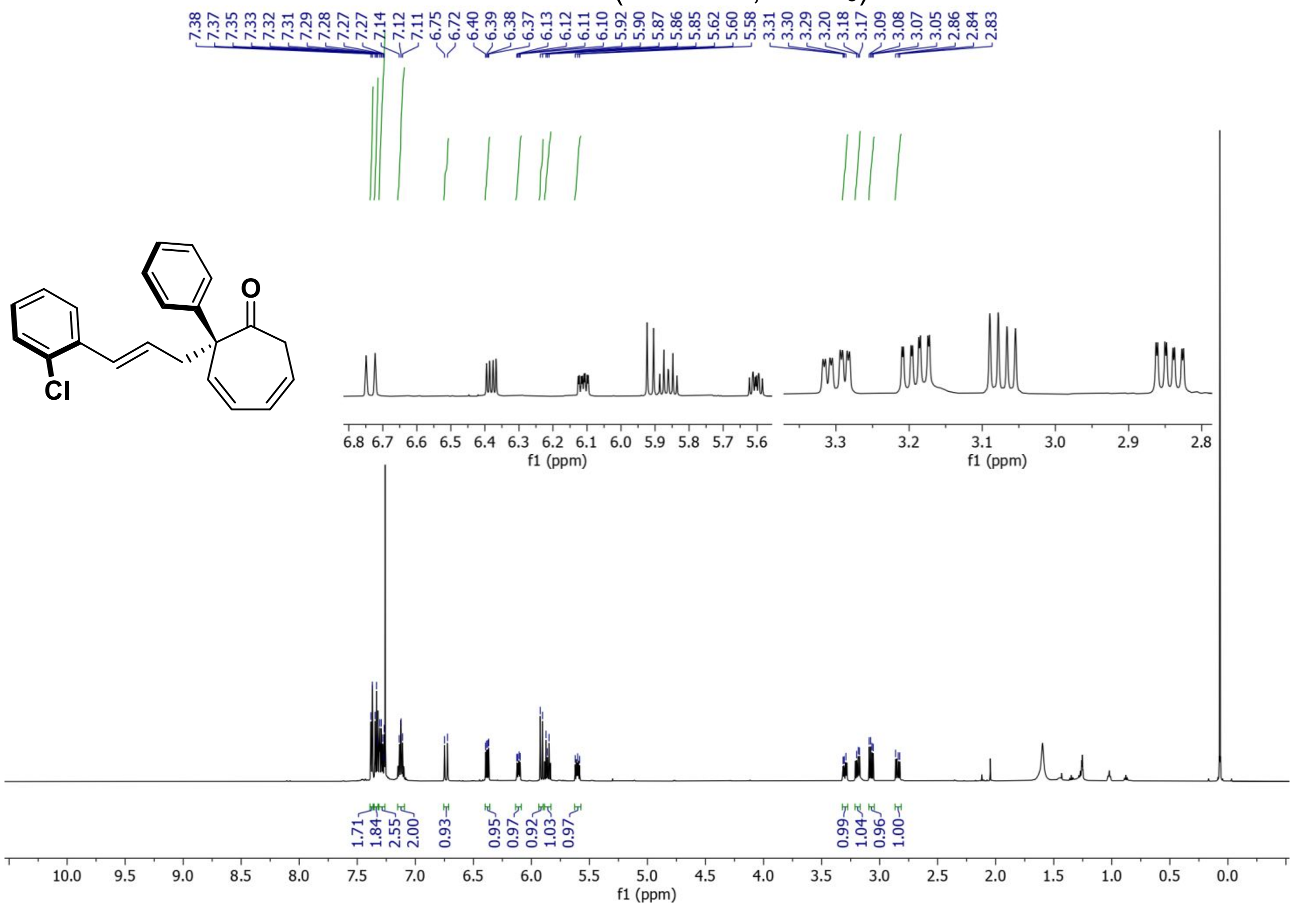

3ae ¹³C NMR (151 MHz, CDCl$_3$)

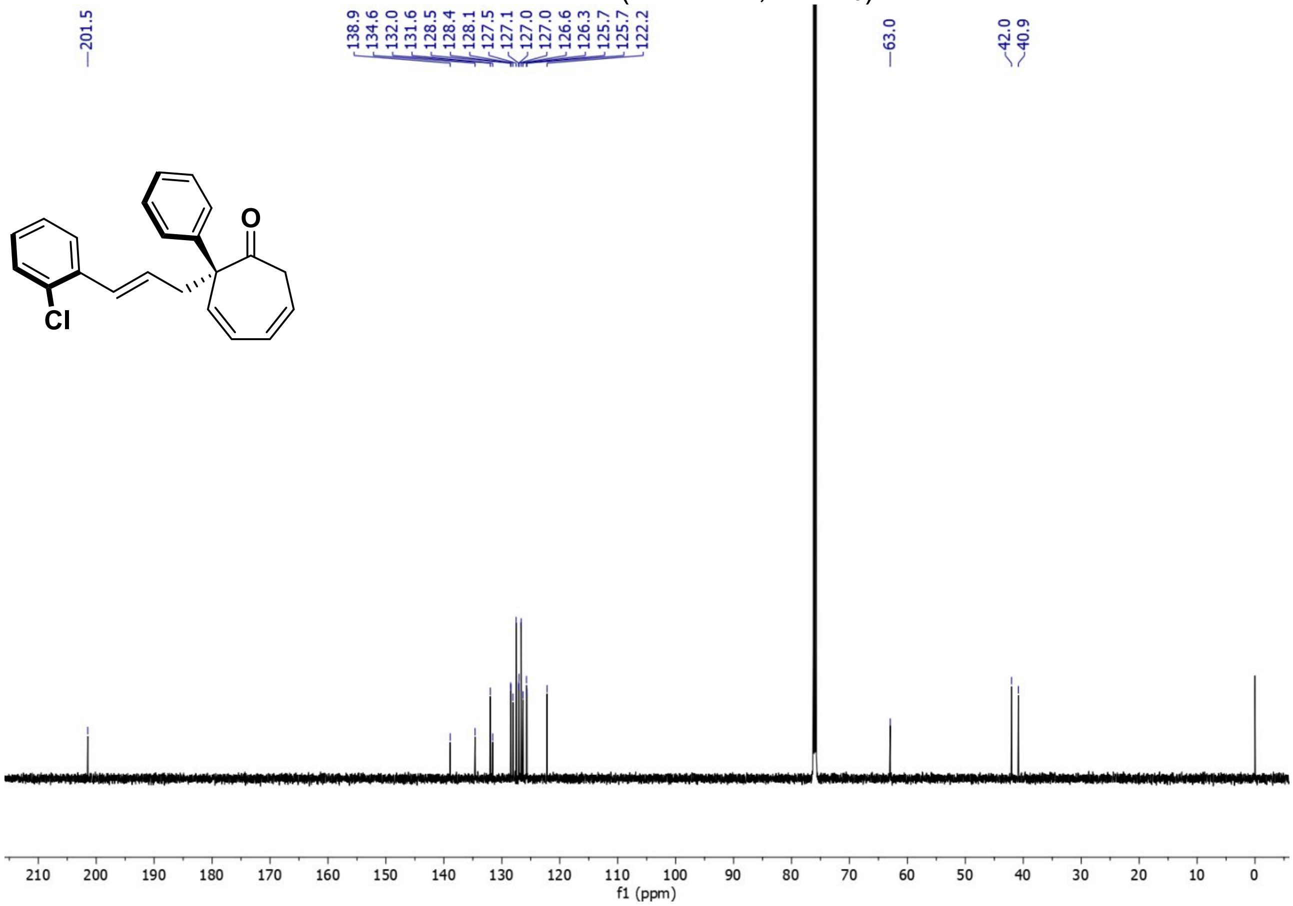

3af ^{1}H NMR (600 MHz, $CDCl_3$)

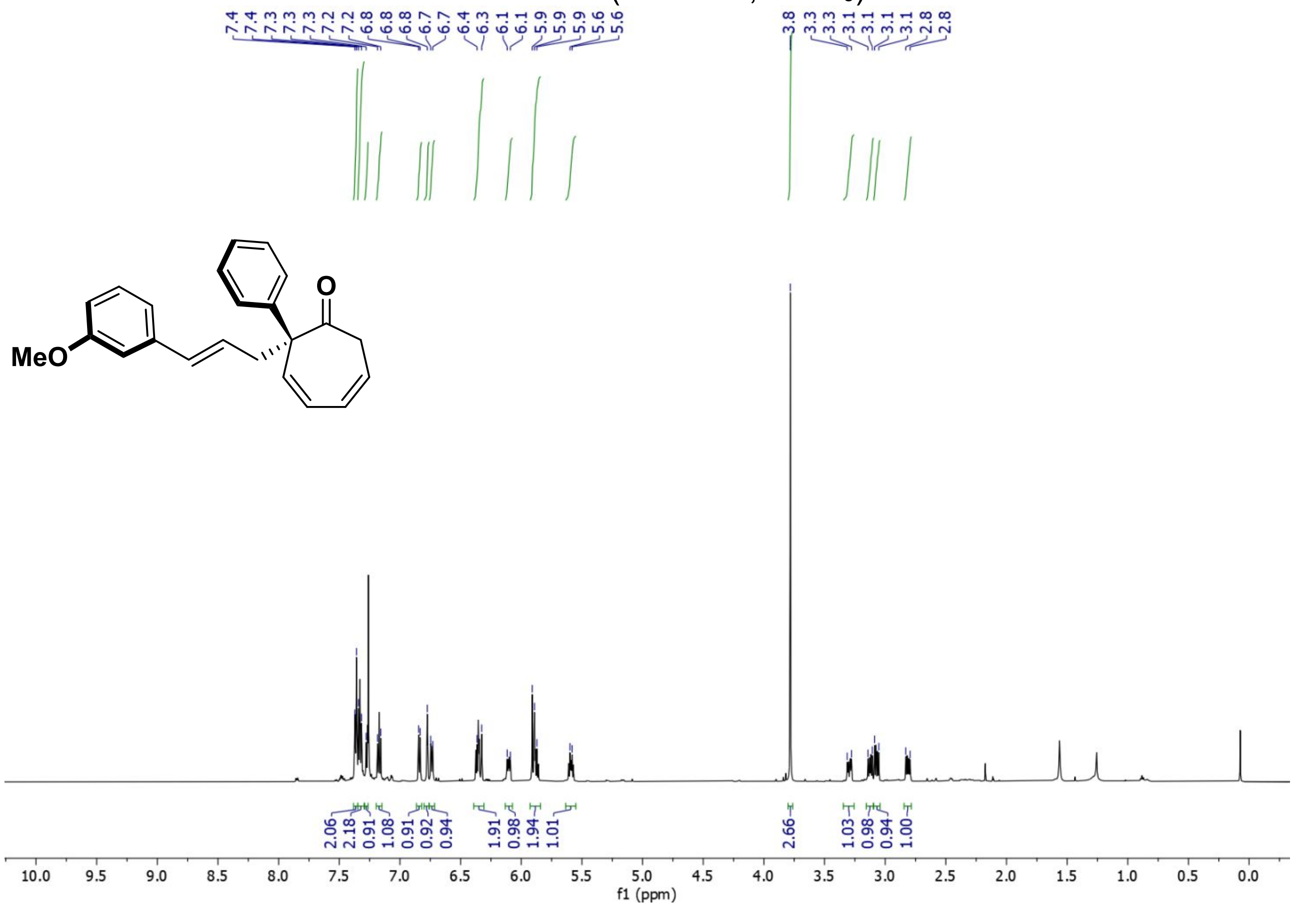

3af ^{13}C NMR (151 MHz, $CDCl_3$)

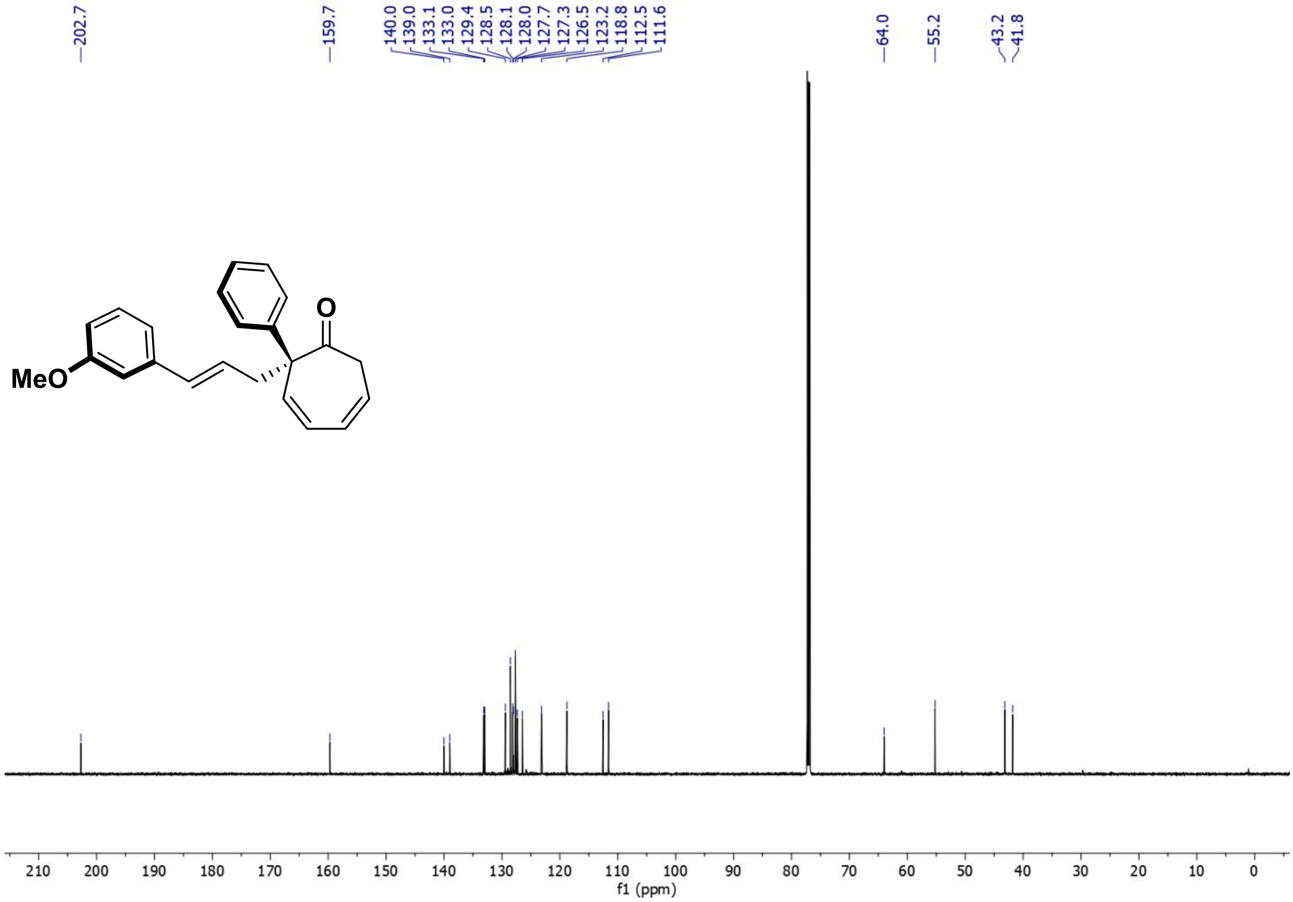

3ag ^{1}H NMR (600 MHz, $CDCl_3$)

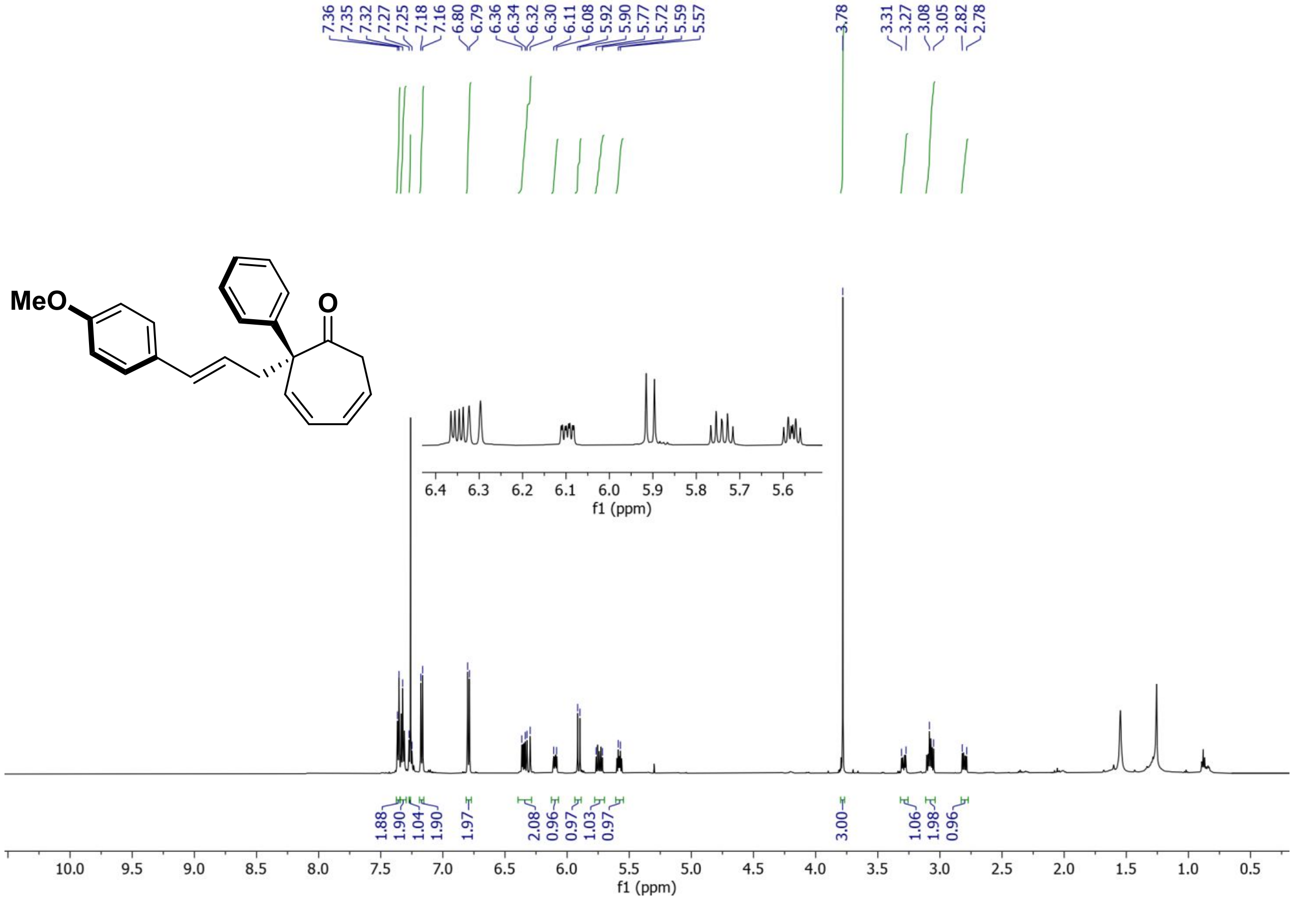

3ag ^{13}C NMR (151 MHz, $CDCl_3$)

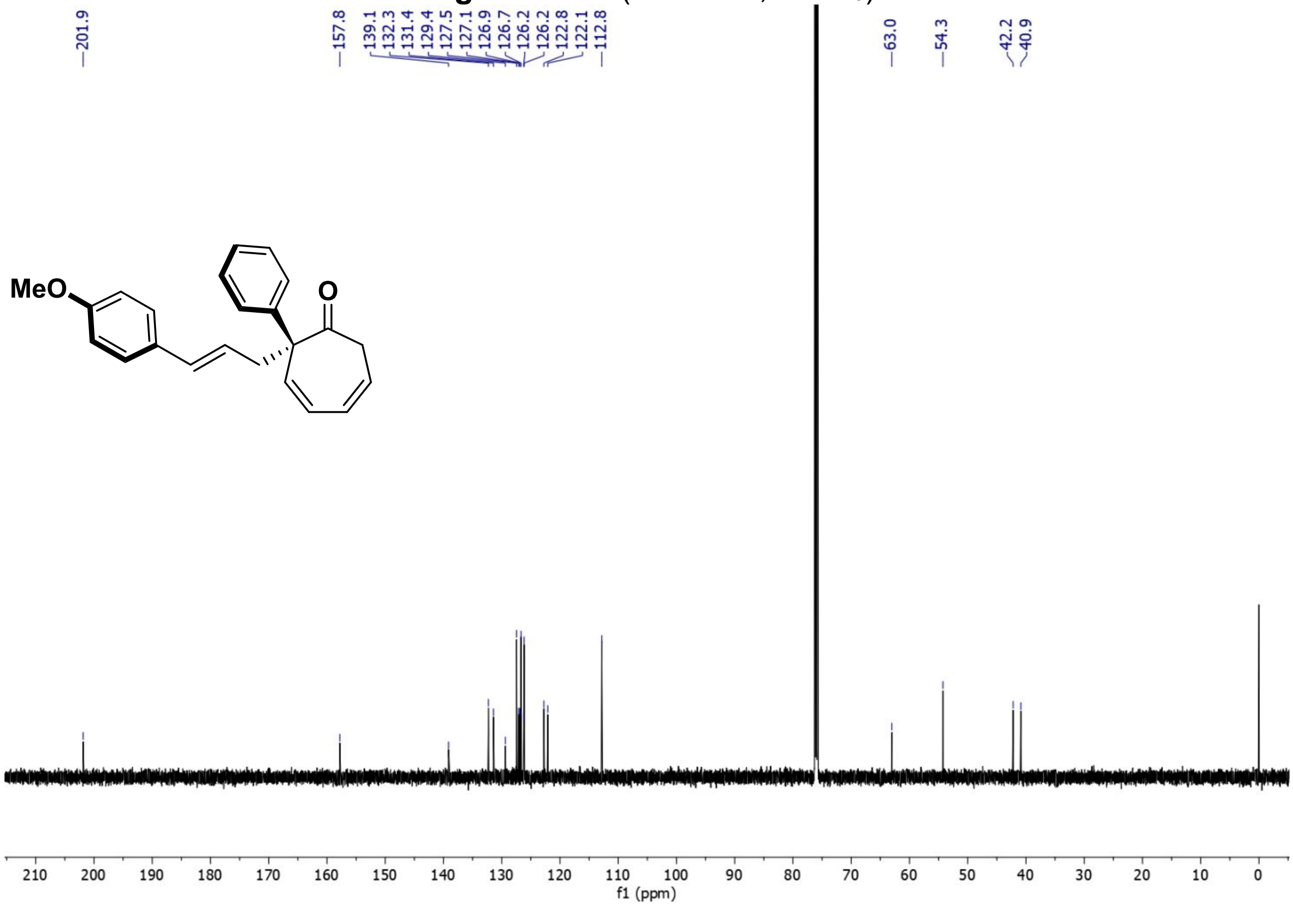

3ah ^{1}H NMR (600 MHz, $CDCl_3$)

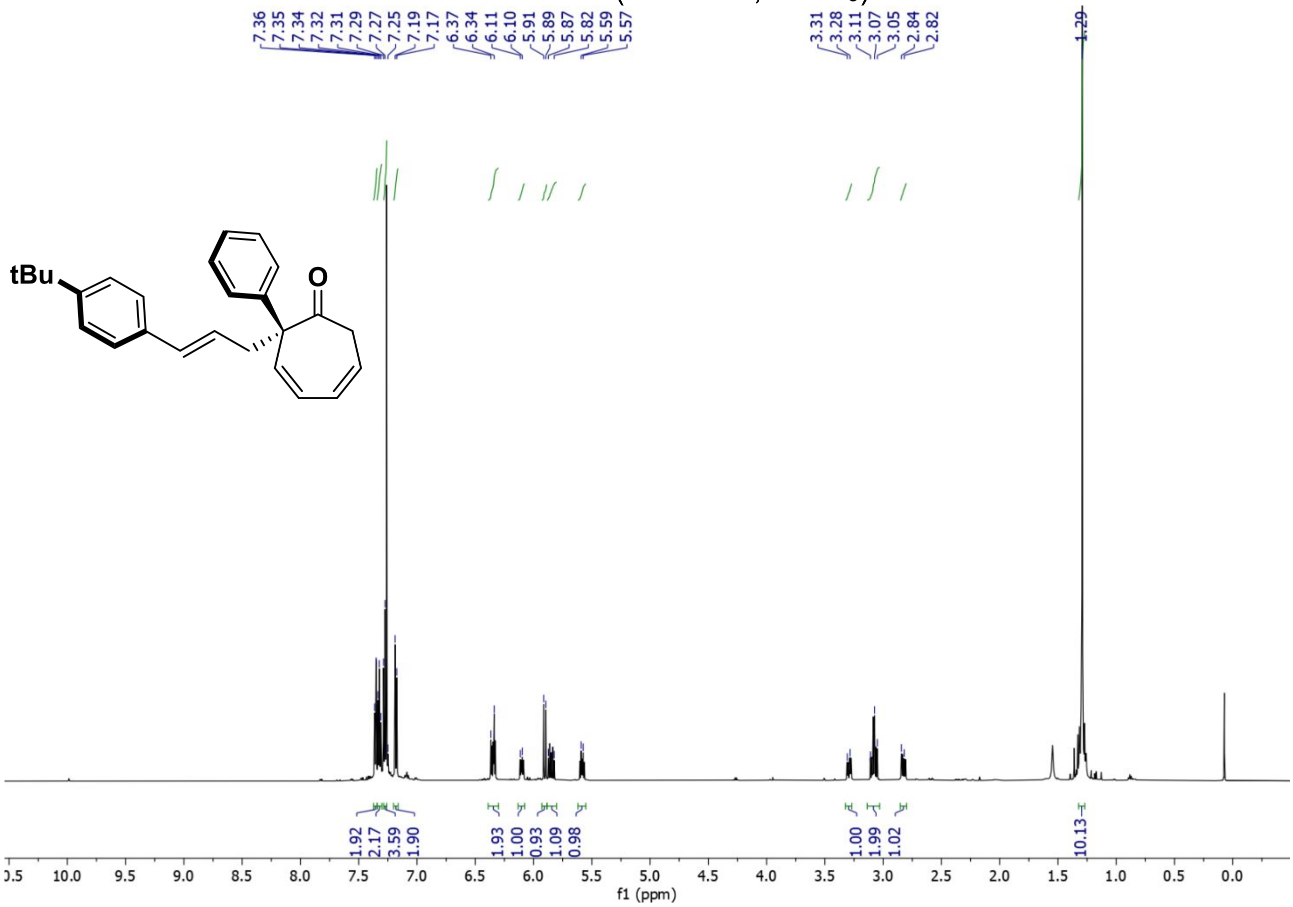

3ah ^{13}C NMR (151 MHz, $CDCl_3$)

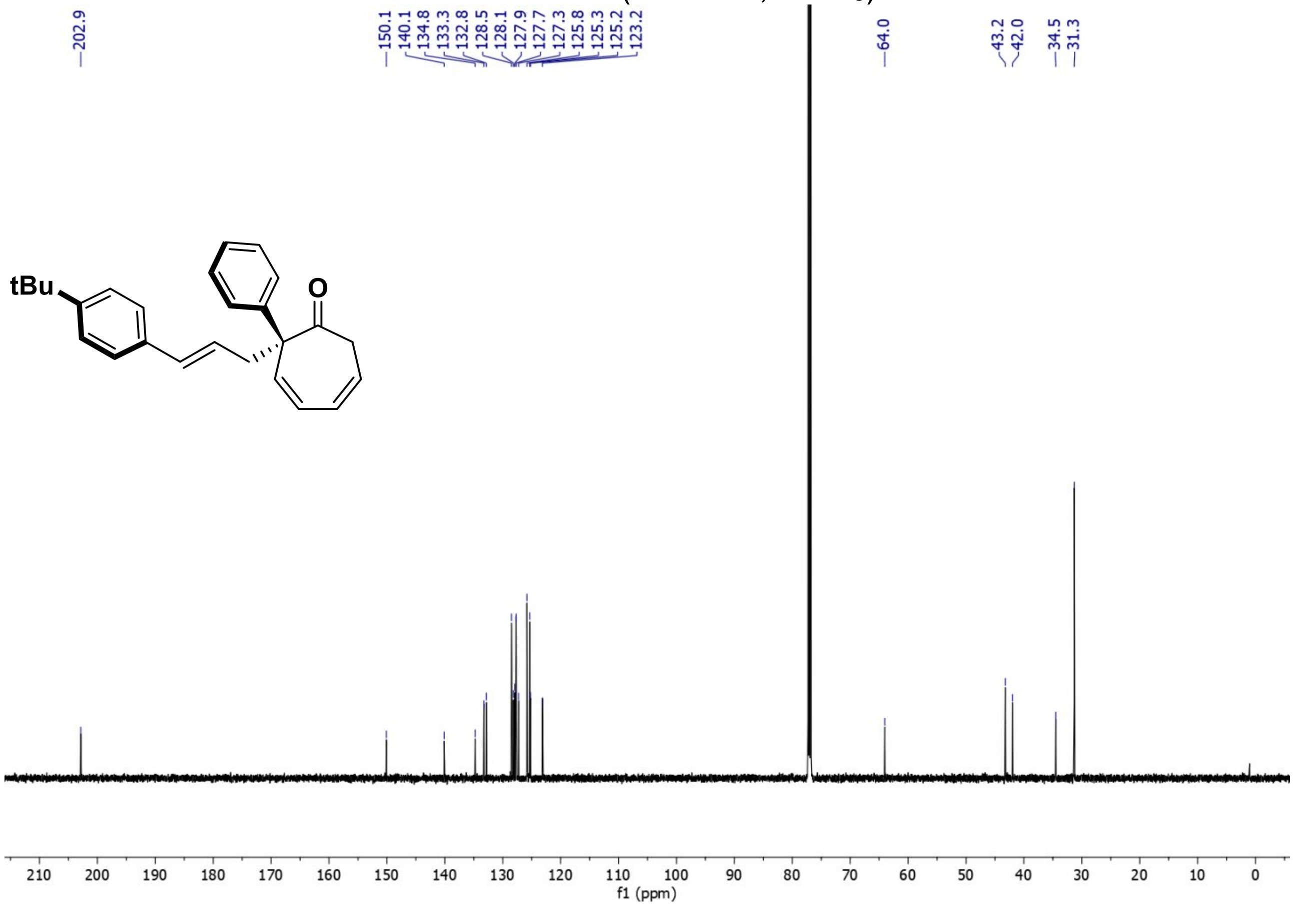

3ai ^{1}**H NMR** (600 MHz, $CDCl_3$)

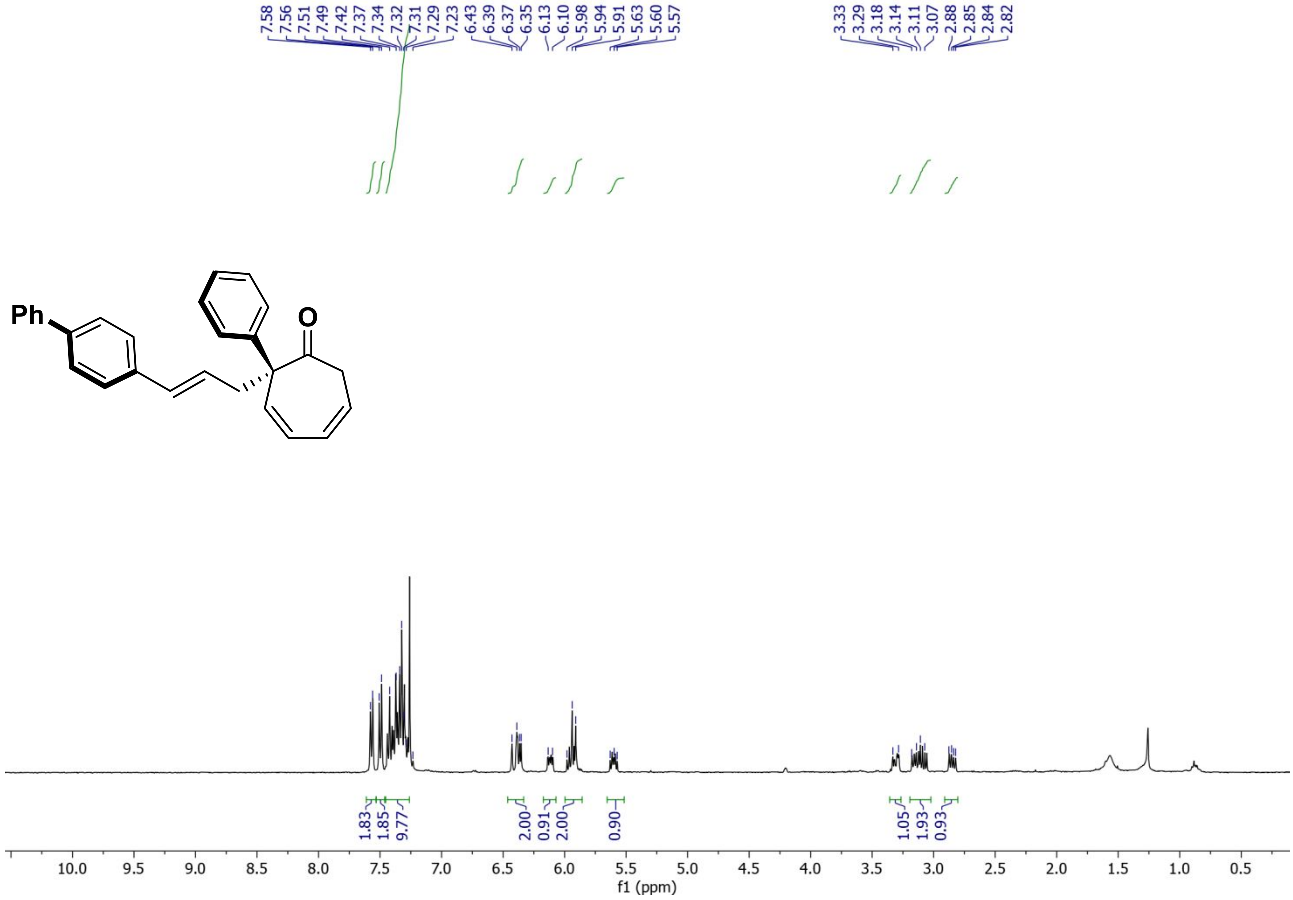

3ai ^{13}**C NMR** (151 MHz, $CDCl_3$)

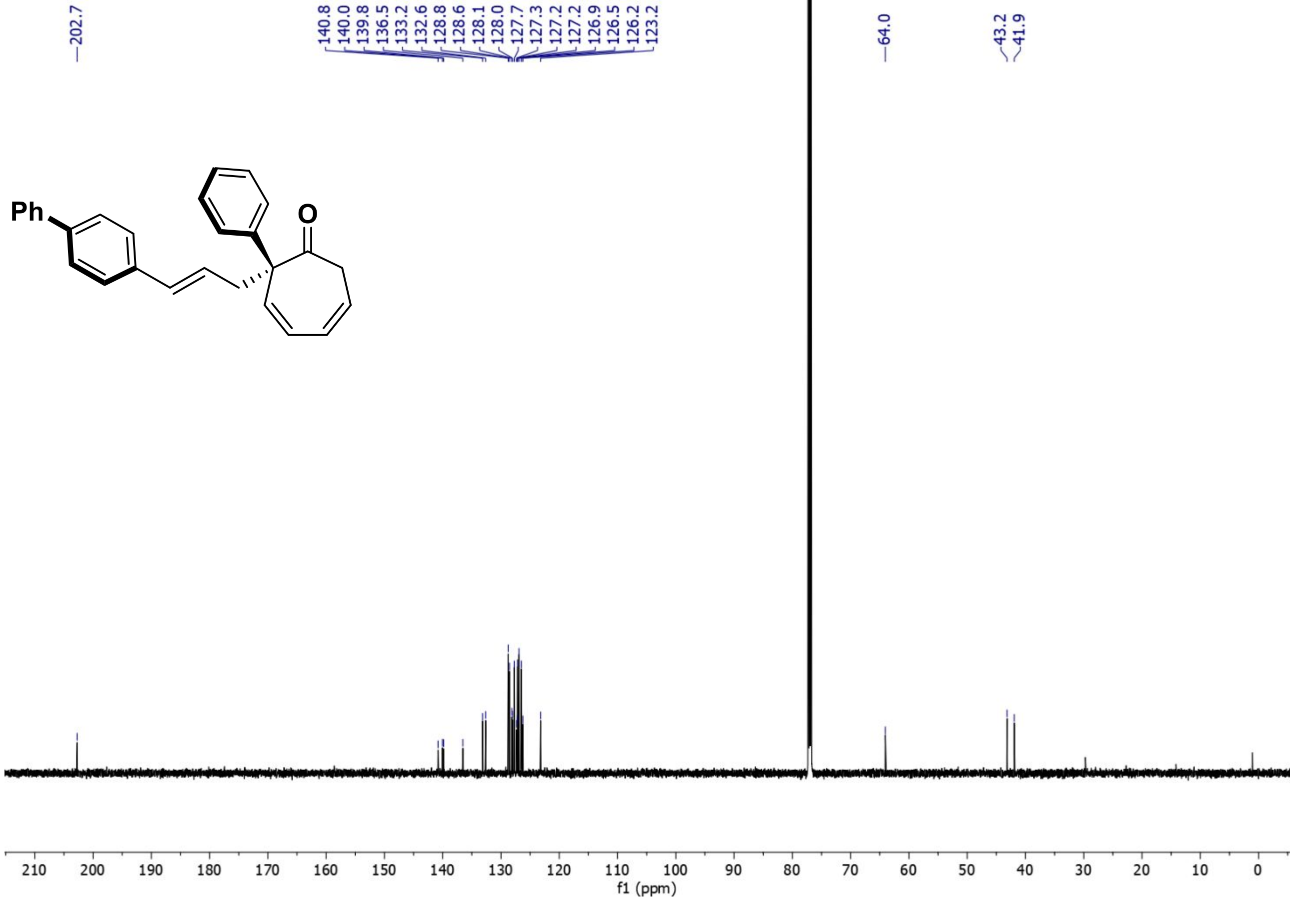

3aj **^{1}H NMR** (600 MHz, $CDCl_3$)

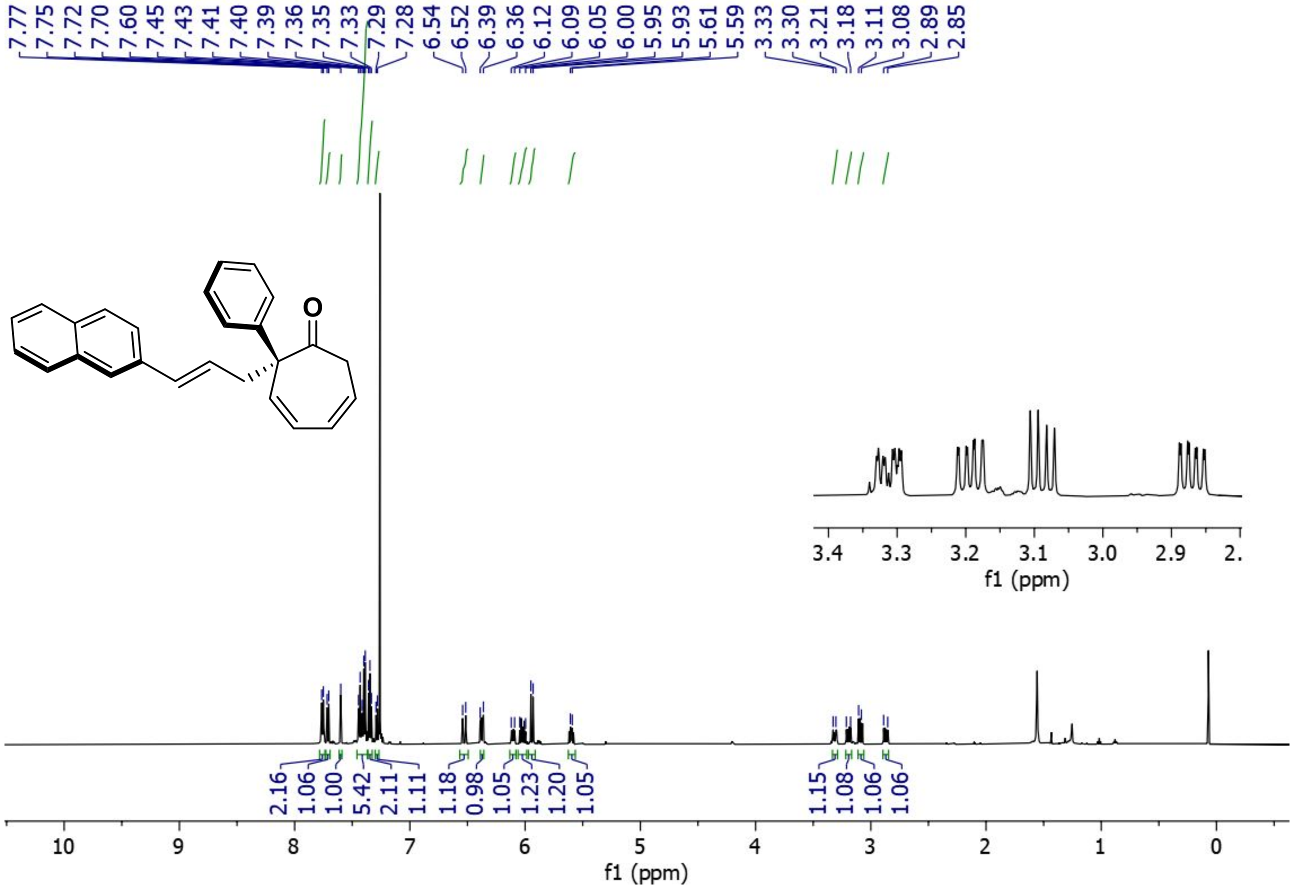

3aj **^{13}C NMR** (151 MHz, $CDCl_3$)

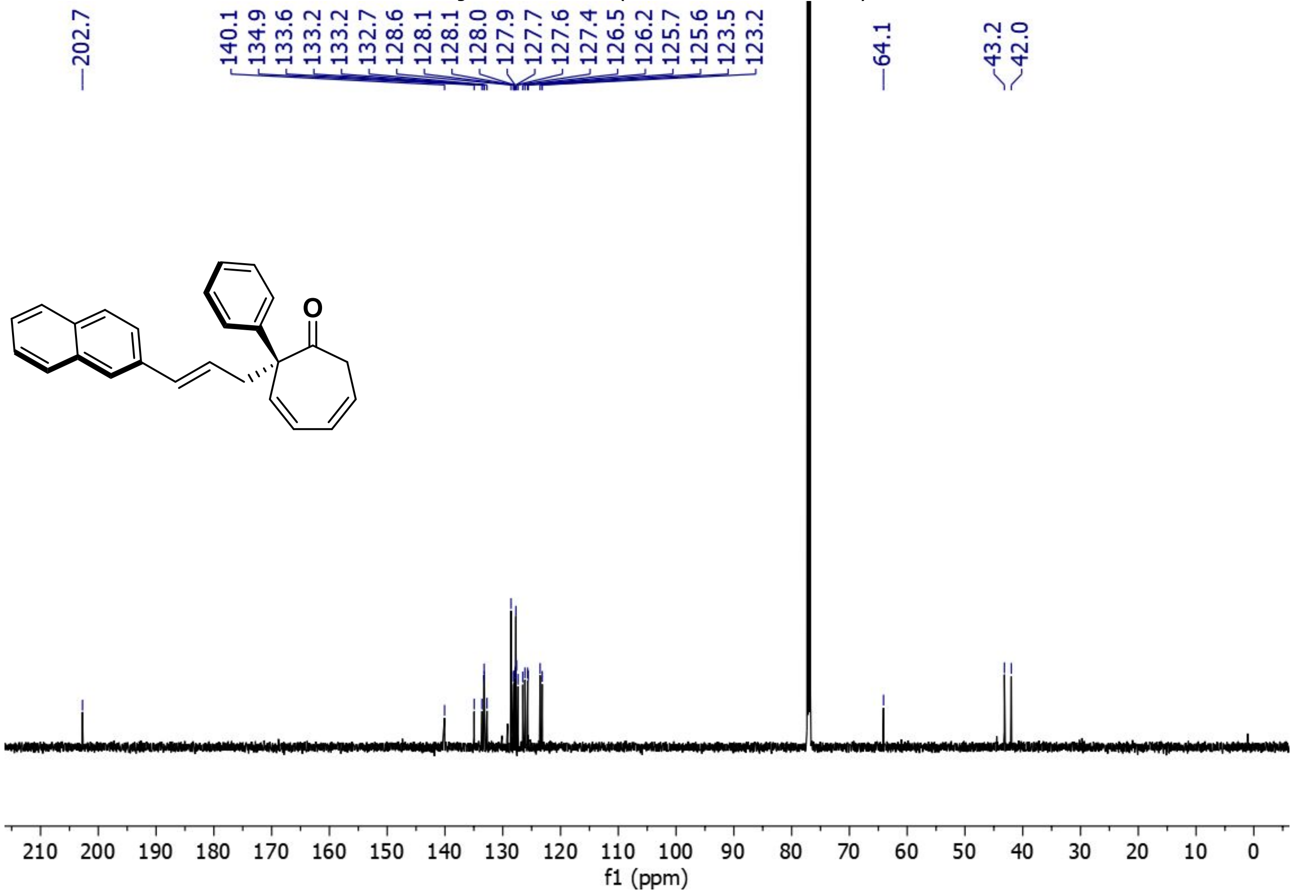

3ak ¹H NMR (600 MHz, CDCl$_3$)

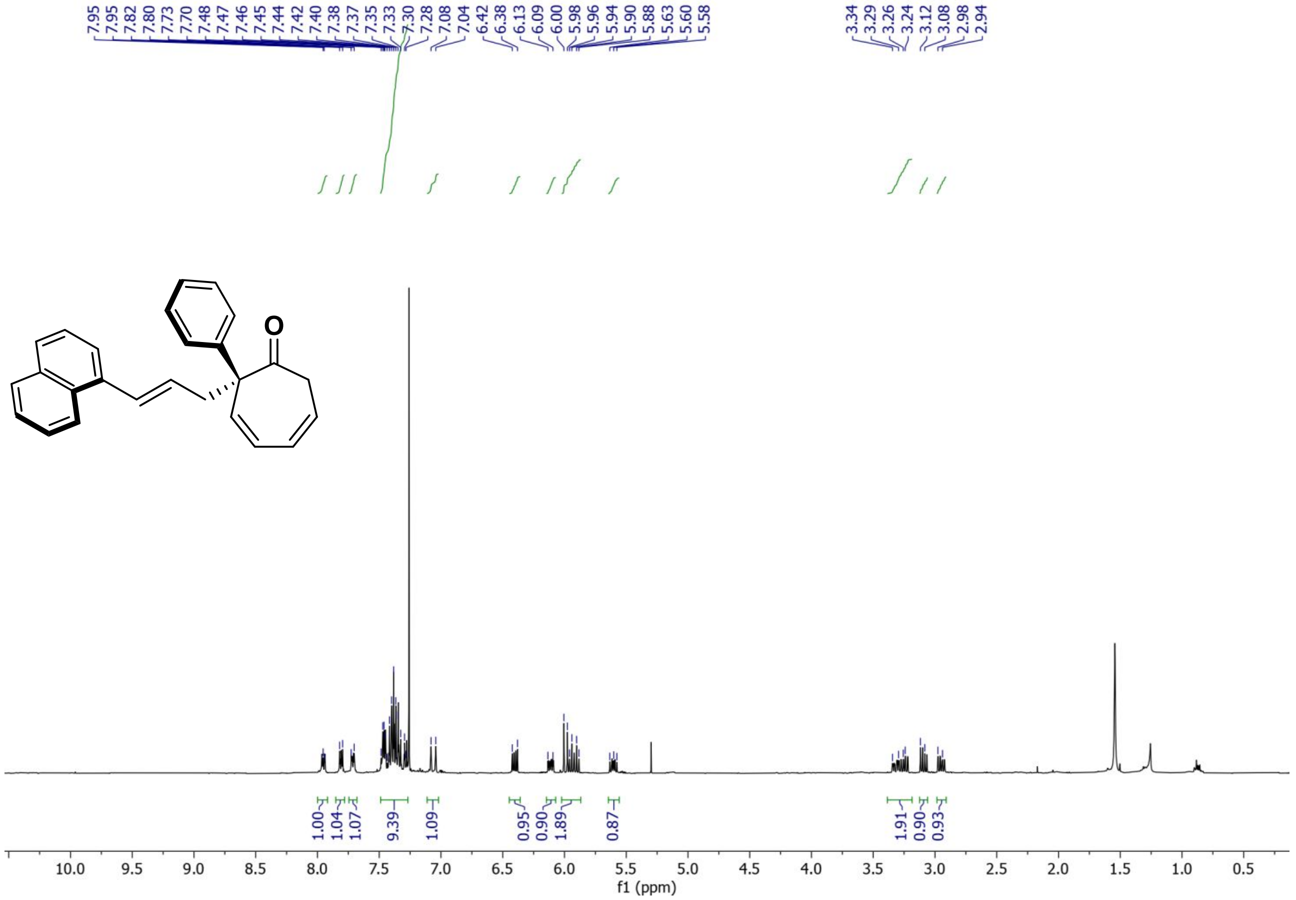

3ak ¹³C NMR (151 MHz, CDCl$_3$)

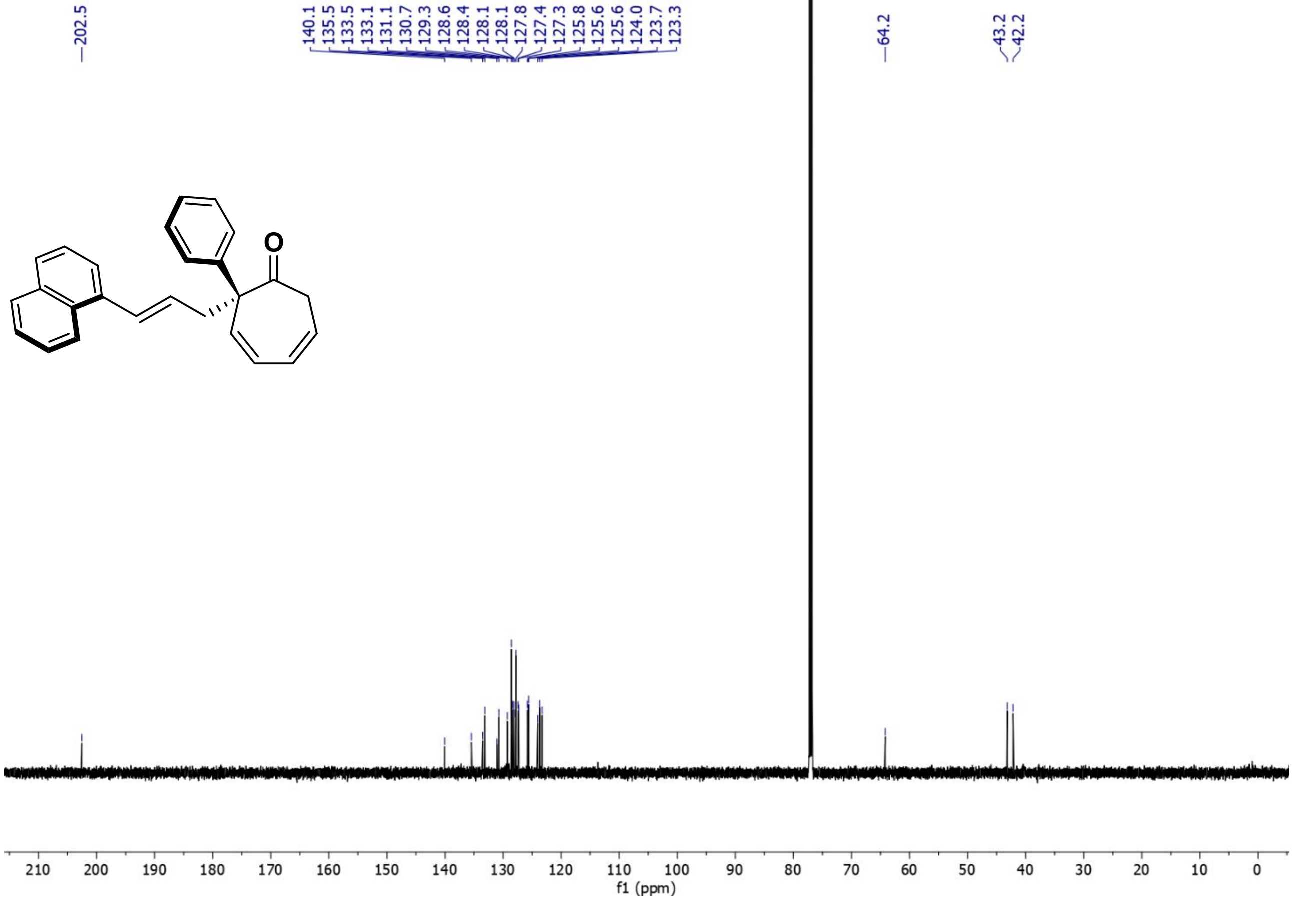

3al ^{1}H NMR (600 MHz, $CDCl_3$)

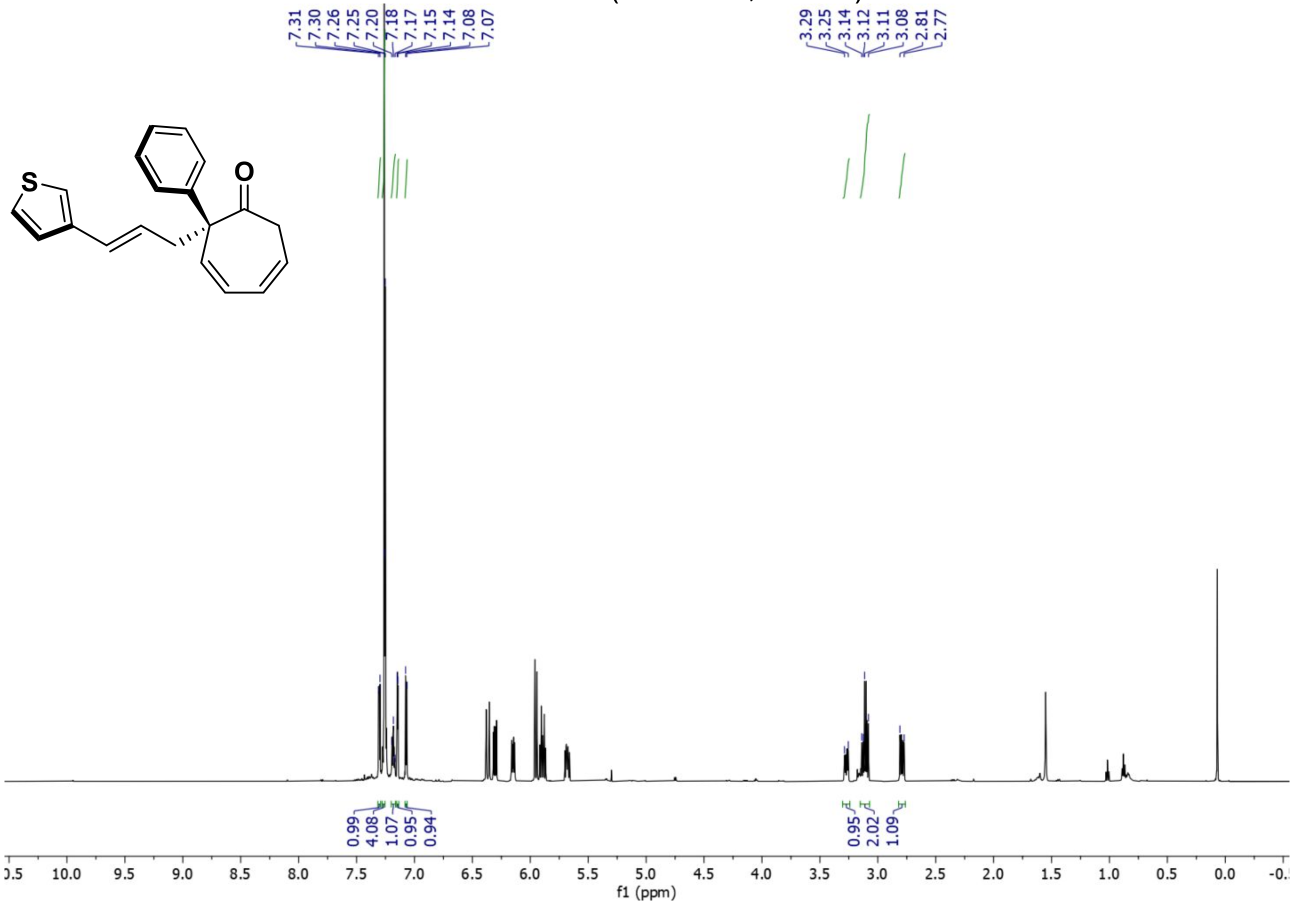

3al ^{13}C NMR (151 MHz, $CDCl_3$)

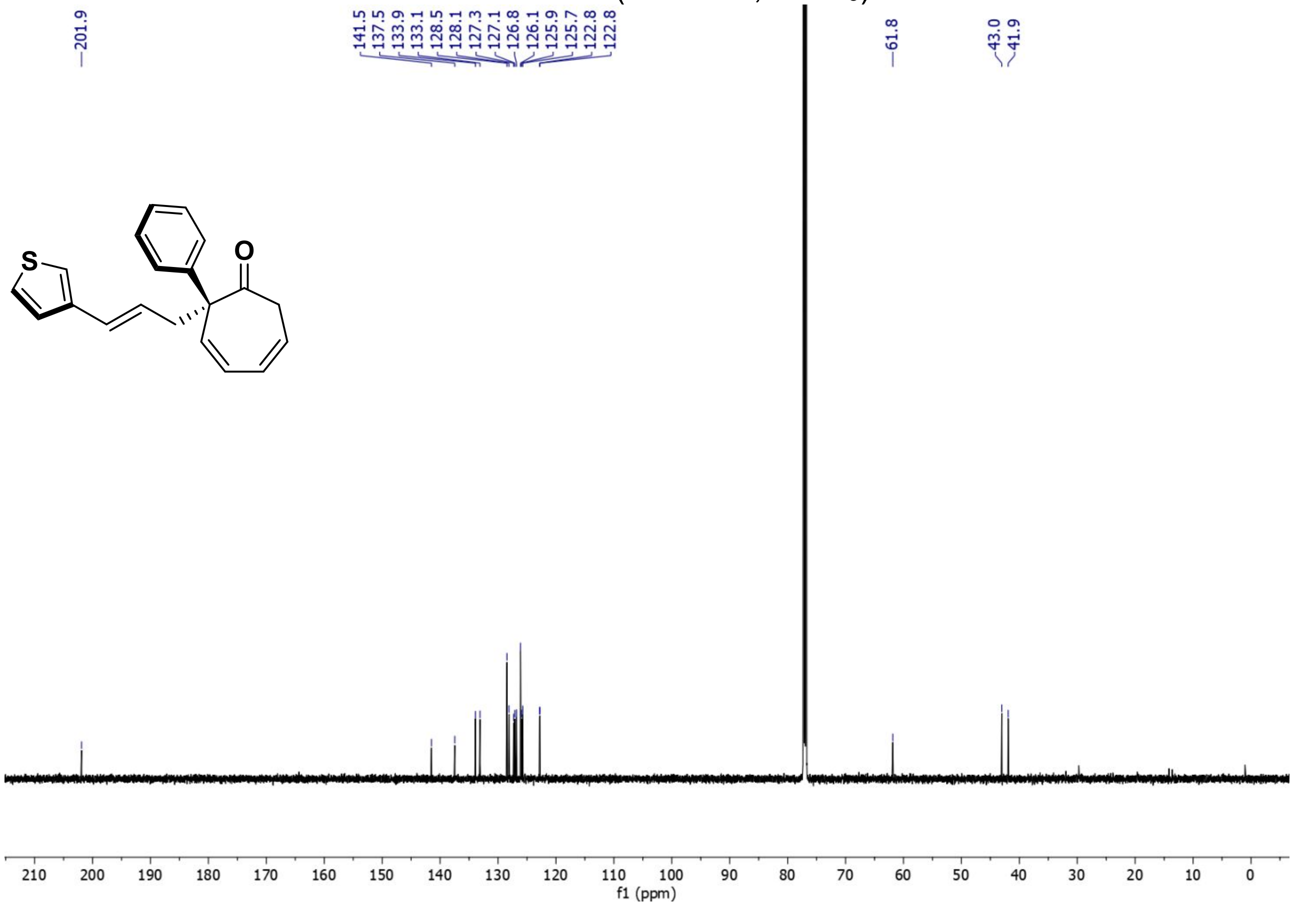

3am ^{1}H NMR (600 MHz, $CDCl_3$)

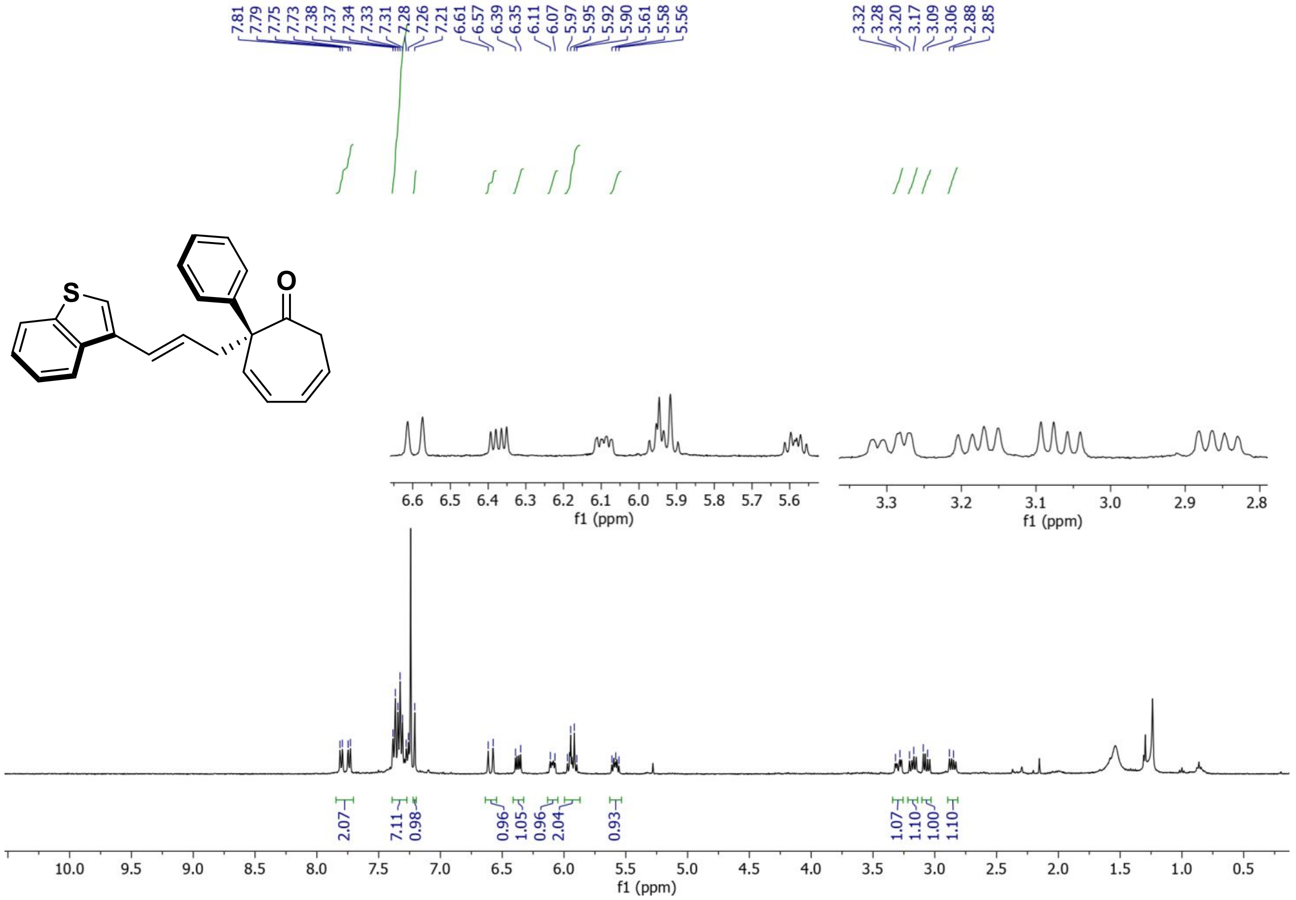

3am ^{13}C NMR (151 MHz, $CDCl_3$)

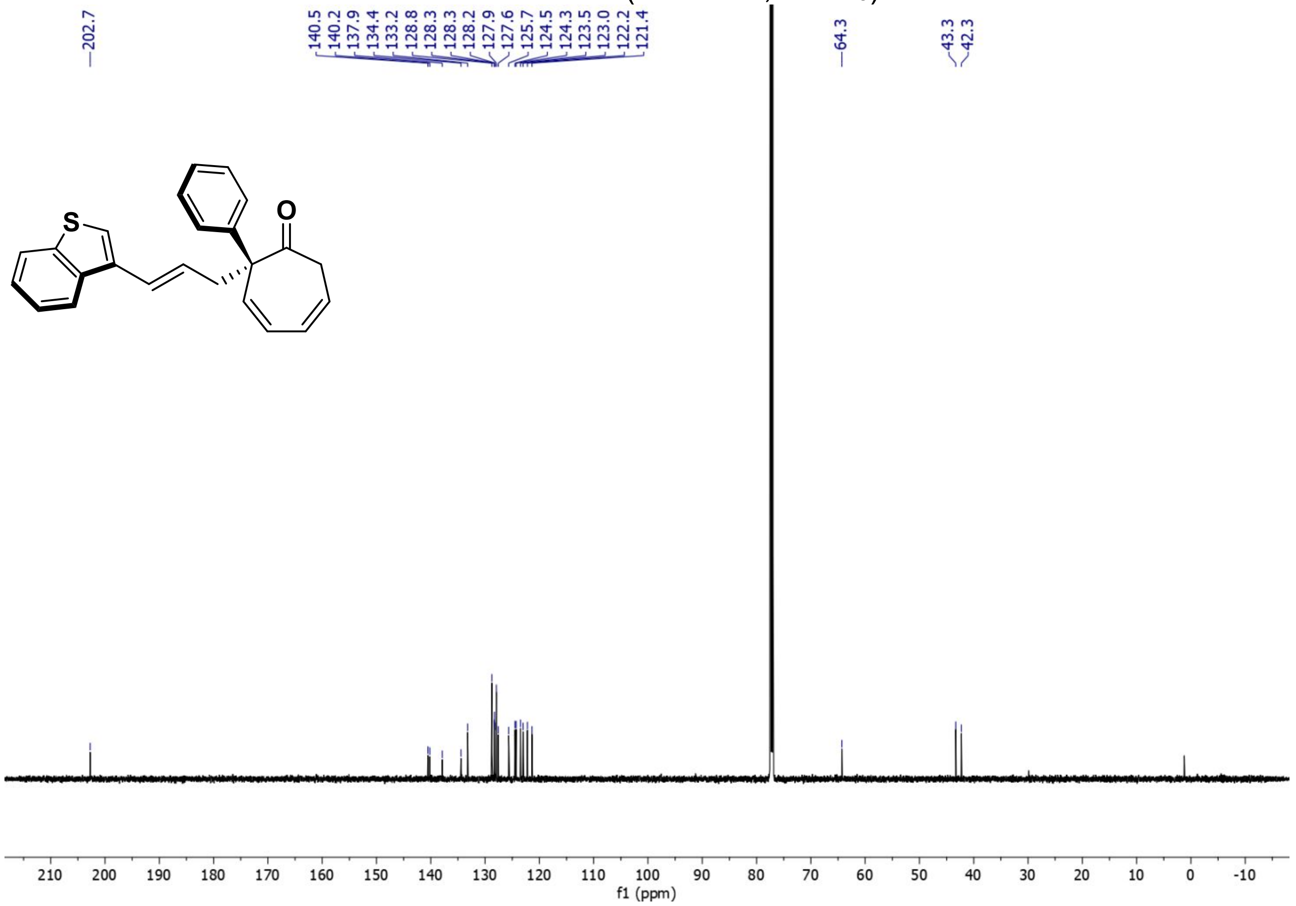

3an ^{1}H NMR (600 MHz, $CDCl_3$)

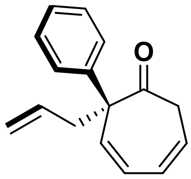

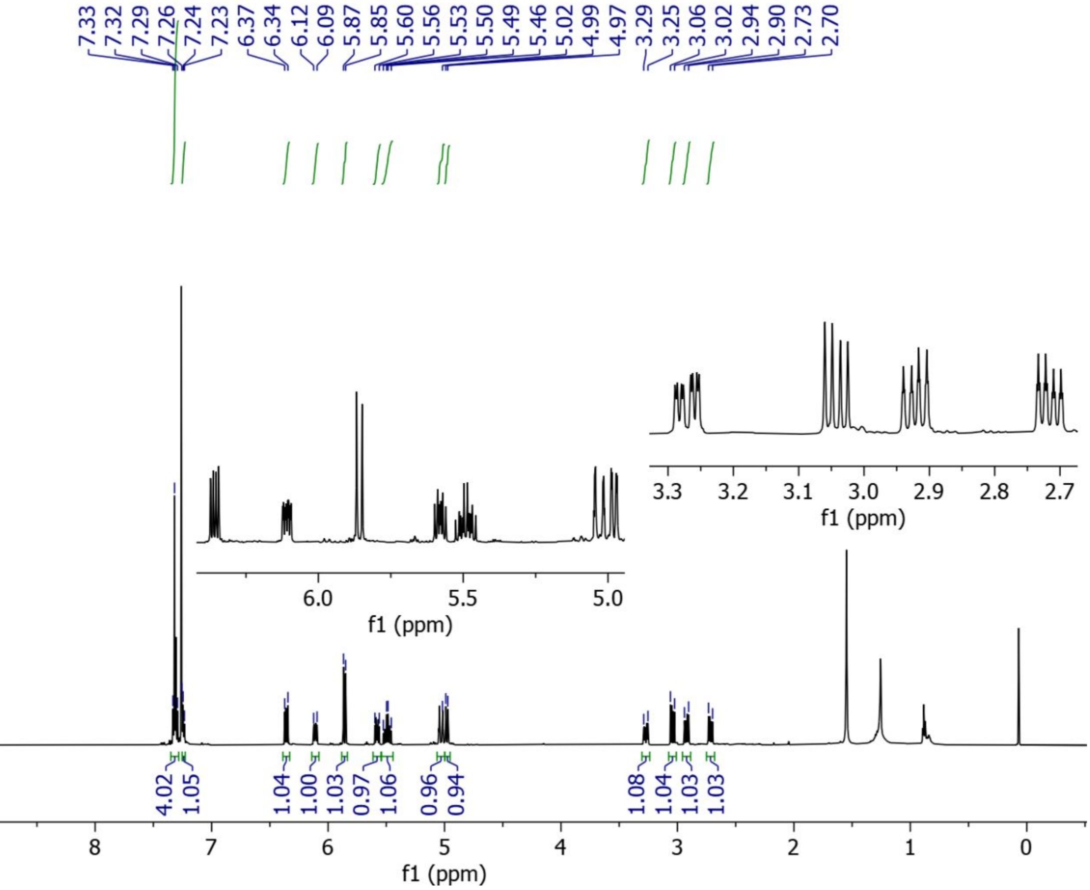

3an ^{13}C NMR (151 MHz, $CDCl_3$)

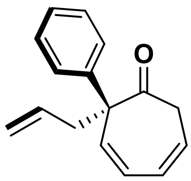

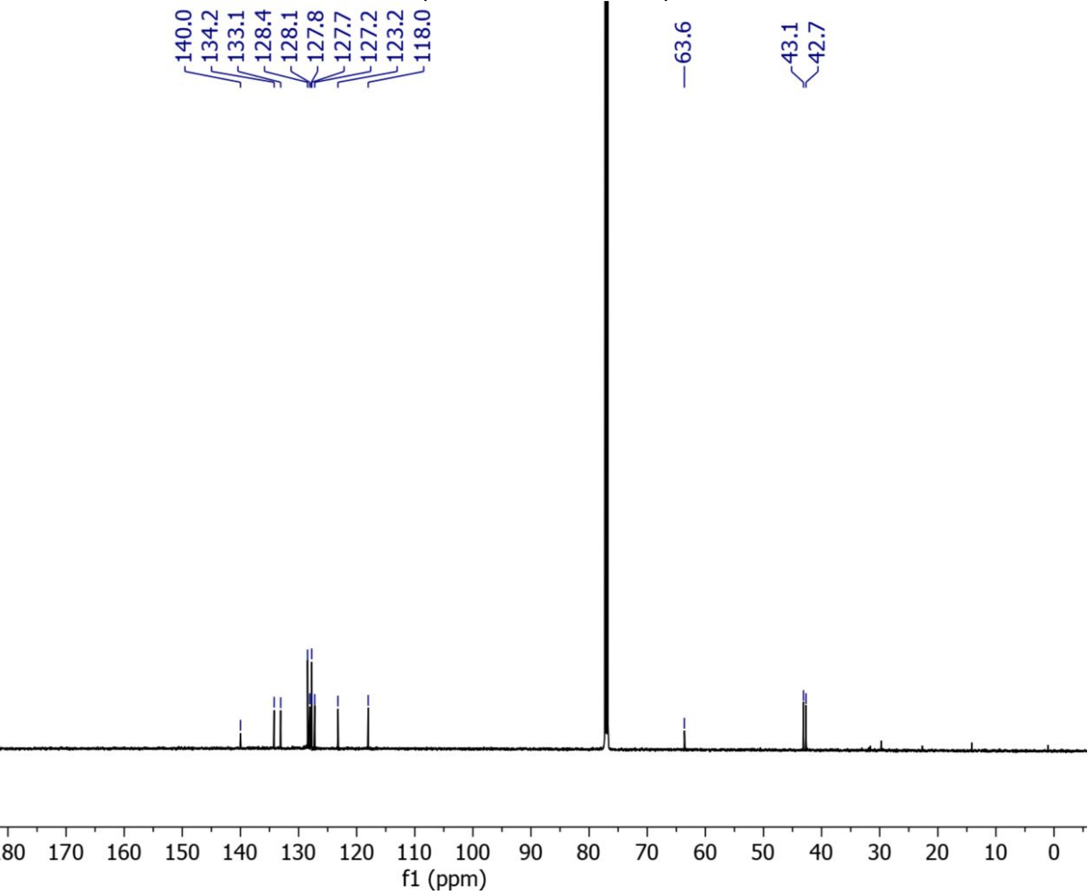

3ba ^{1}H NMR (600 MHz, $CDCl_3$)

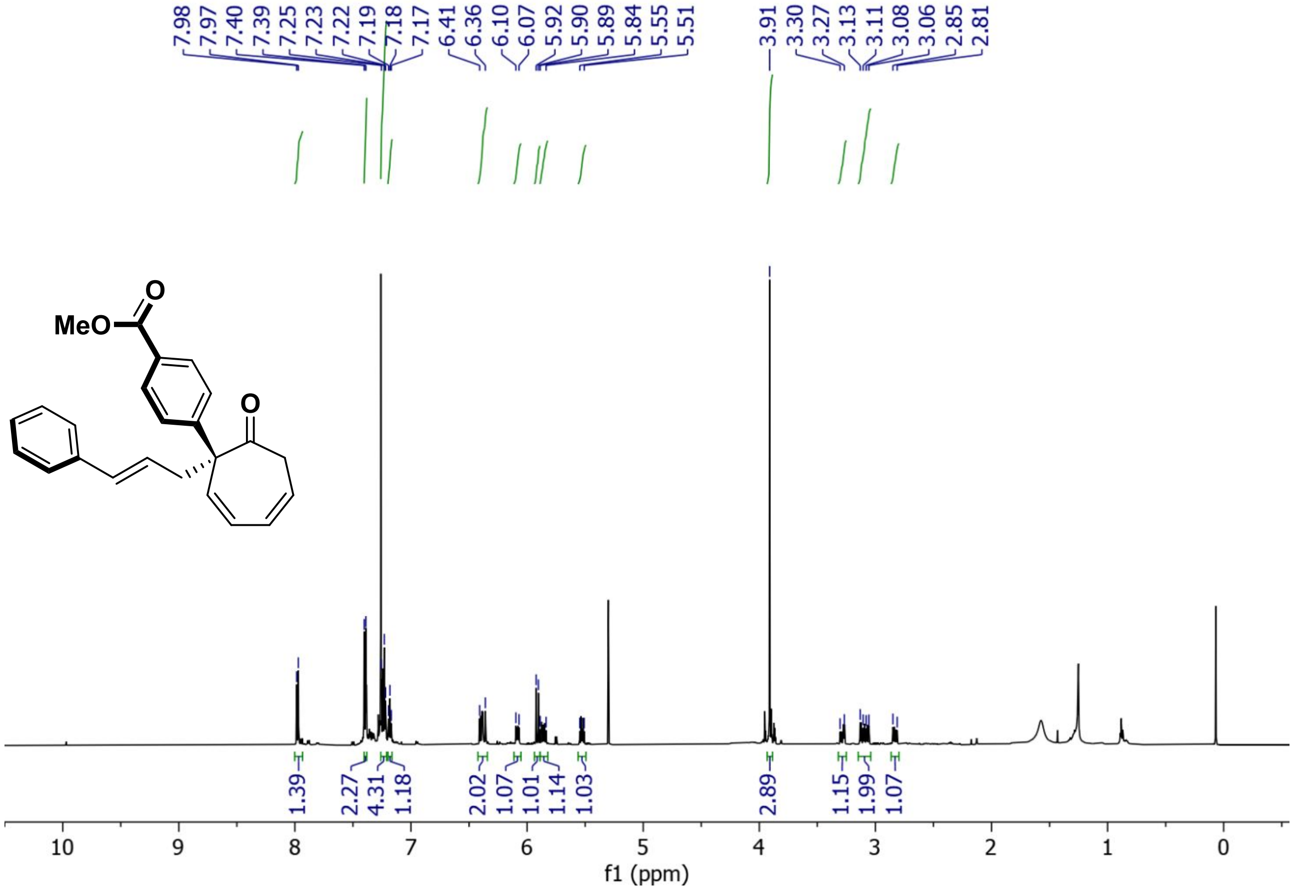

3ba ^{13}C NMR (151 MHz, $CDCl_3$)

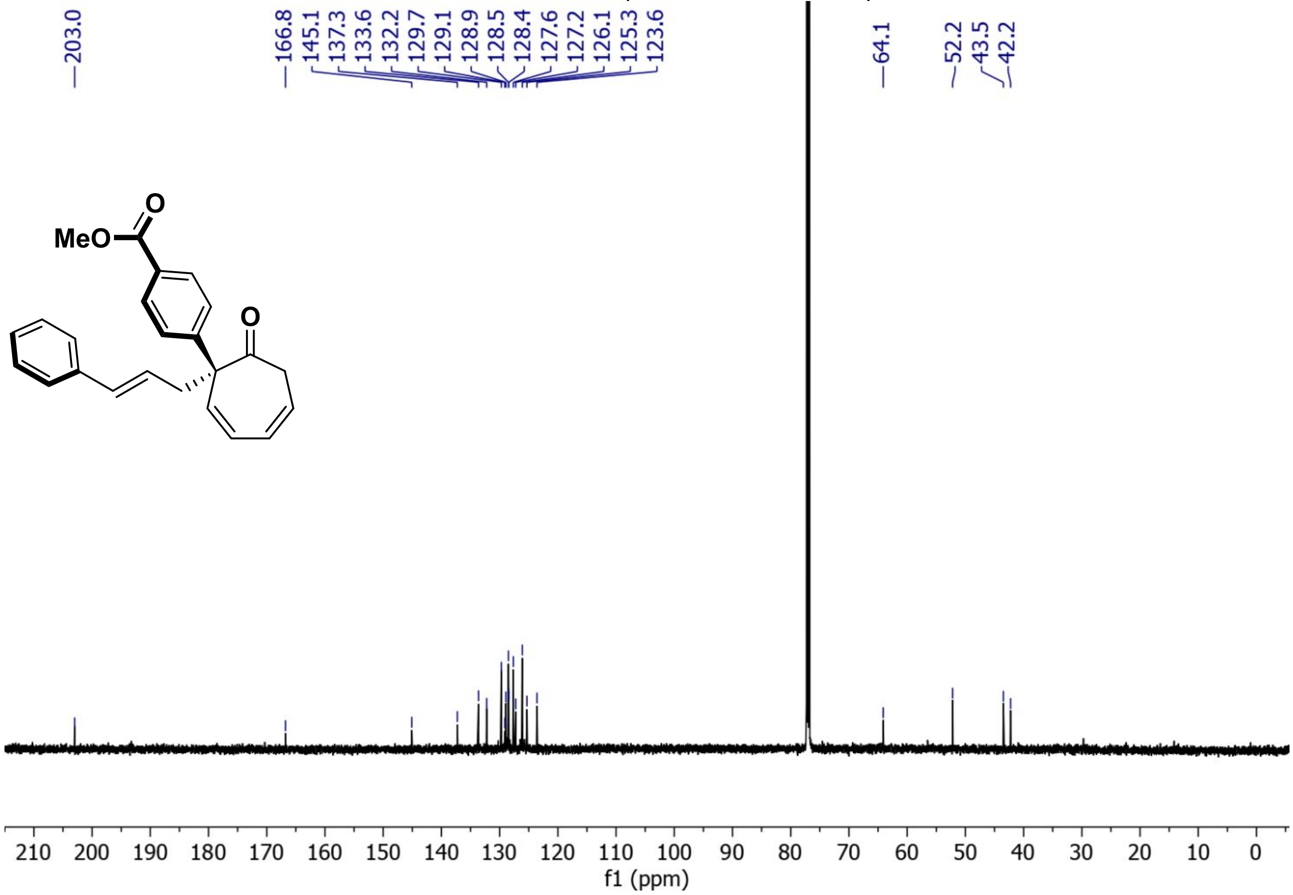

3ca ^{1}H NMR (600 MHz, $CDCl_3$)

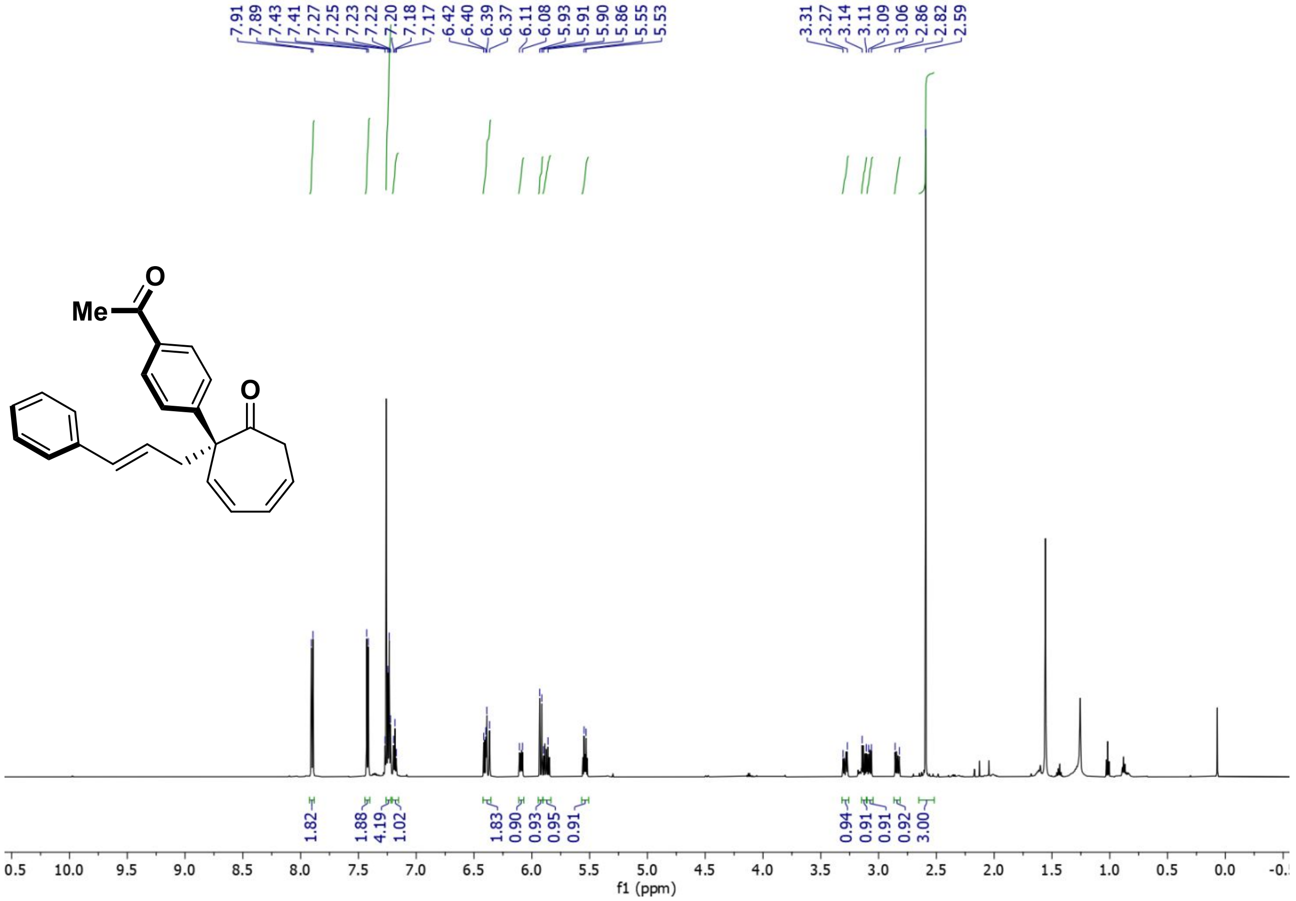

3ca ^{13}C NMR (151 MHz, $CDCl_3$)

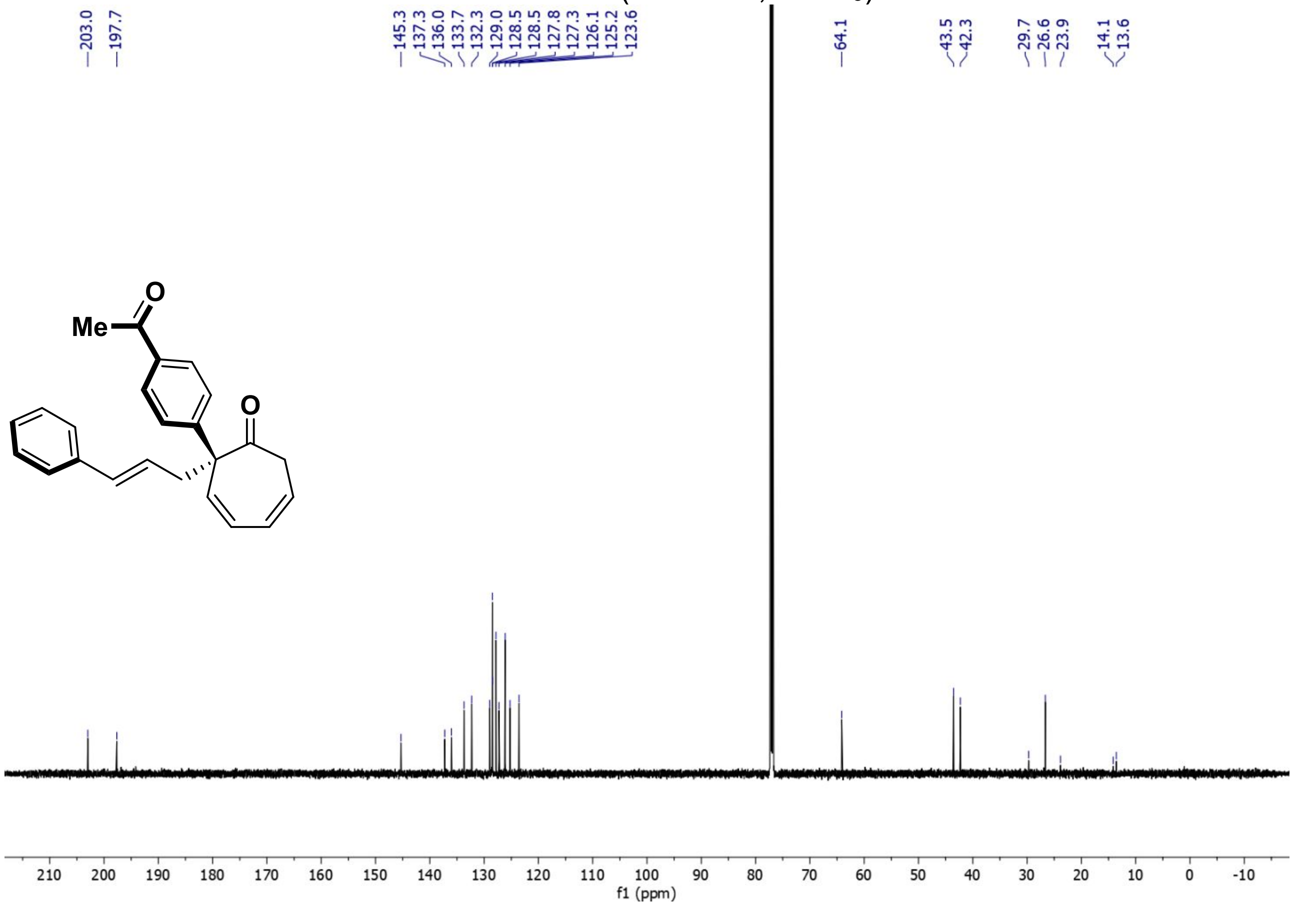

3da ^{1}H NMR (600 MHz, $CDCl_3$)

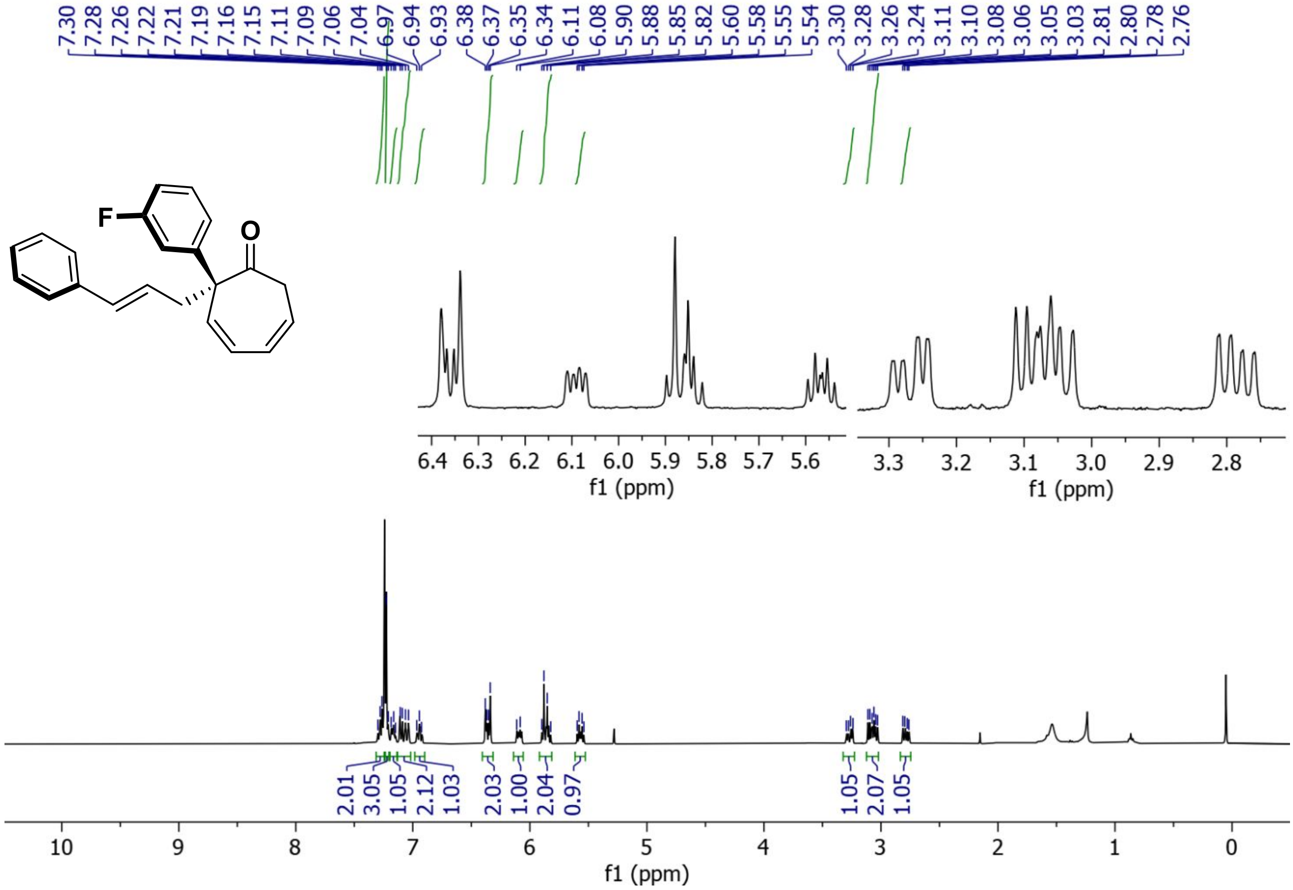

3da ^{13}C NMR (151 MHz, $CDCl_3$)

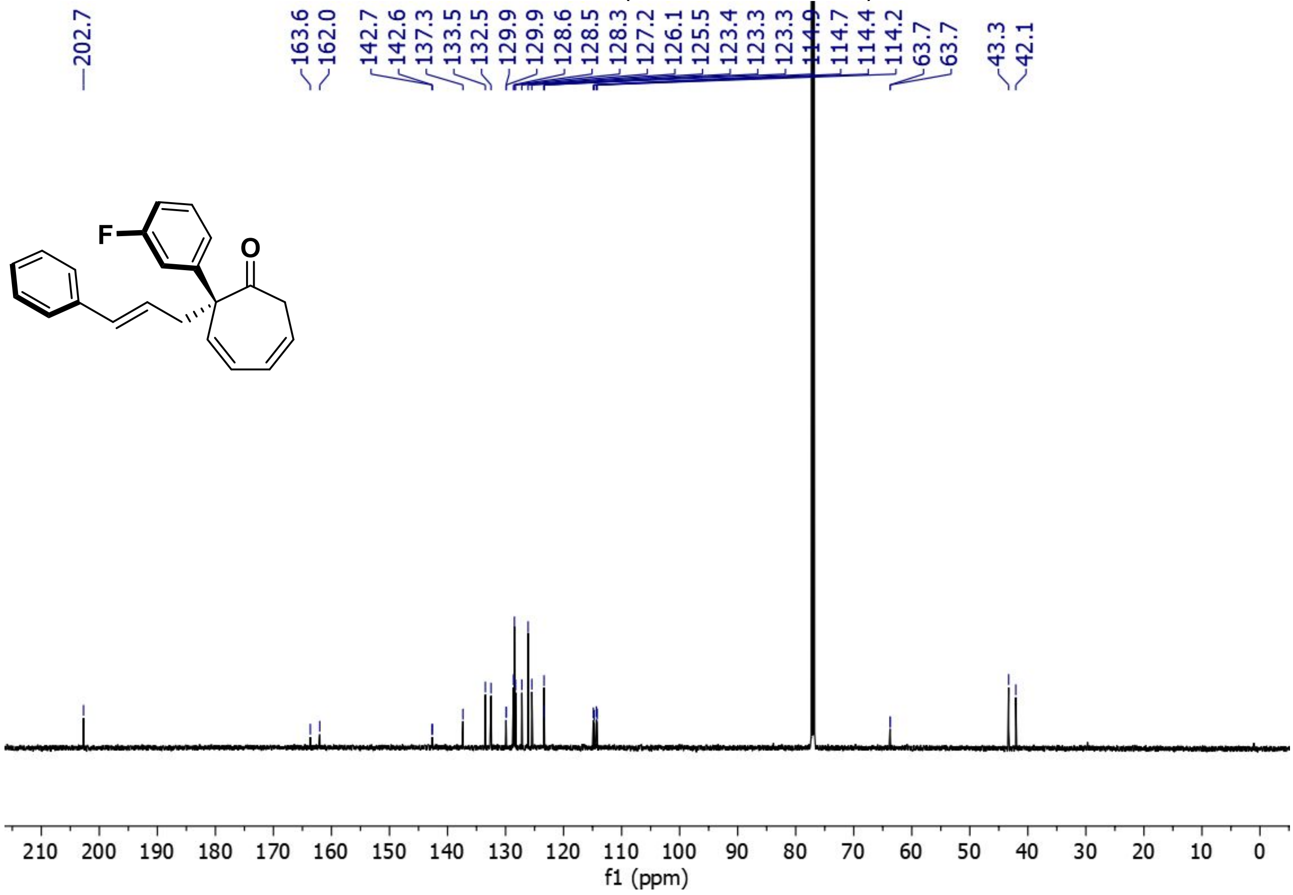

3da ^{19}F NMR (576 MHz, $CDCl_3$)

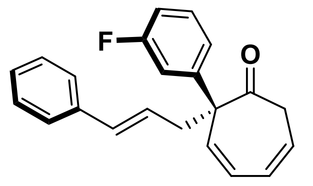

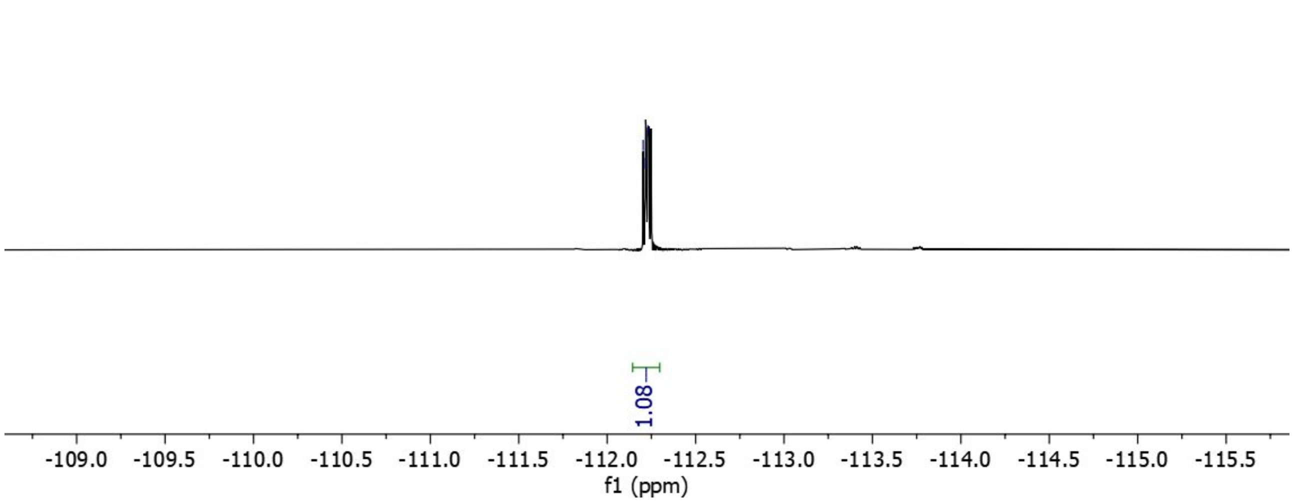

3ea ^{1}H NMR (600 MHz, $CDCl_3$)

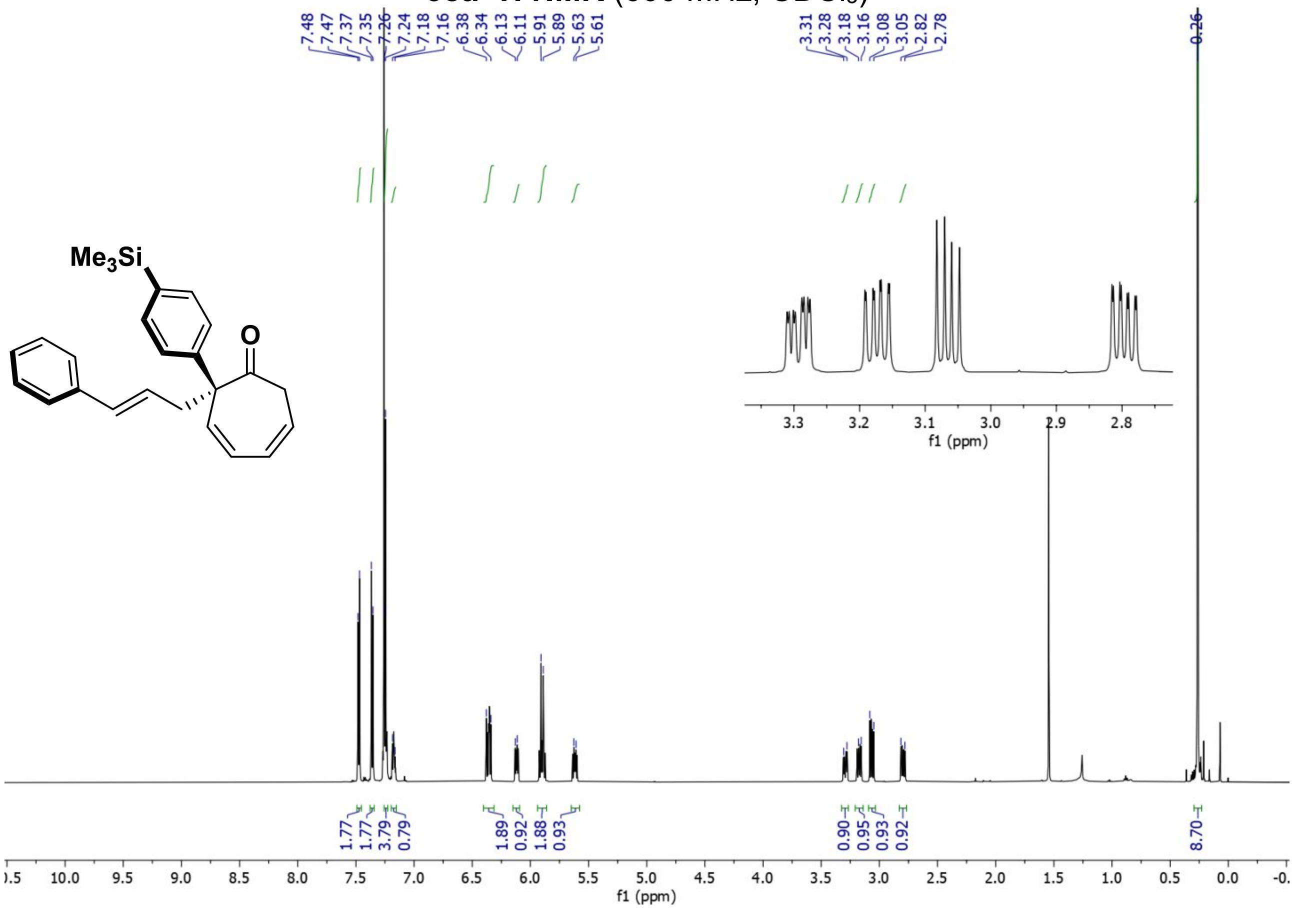

3ea ^{13}C NMR (151 MHz, $CDCl_3$)

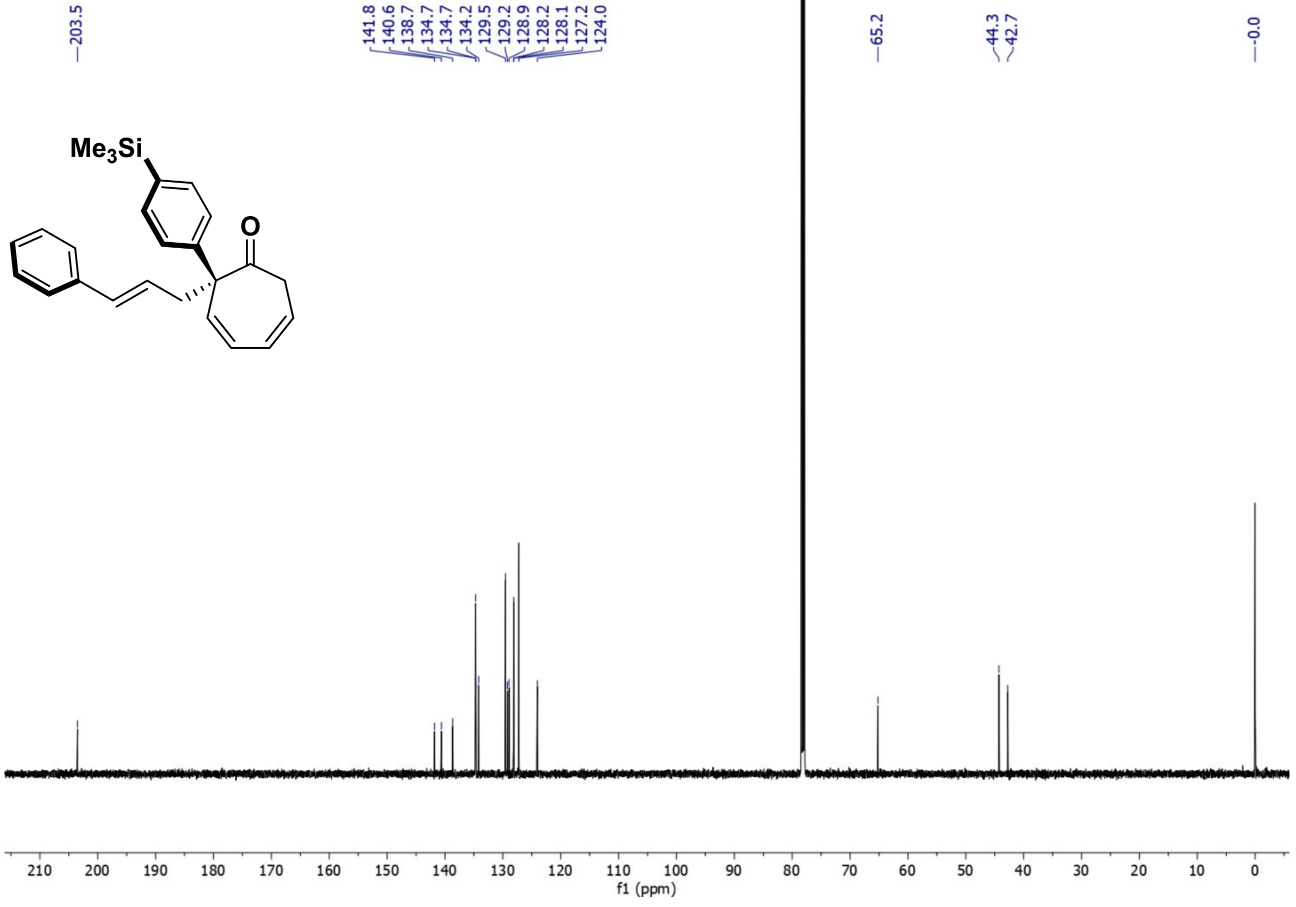

3fa ^{1}H NMR (600 MHz, $CDCl_3$)

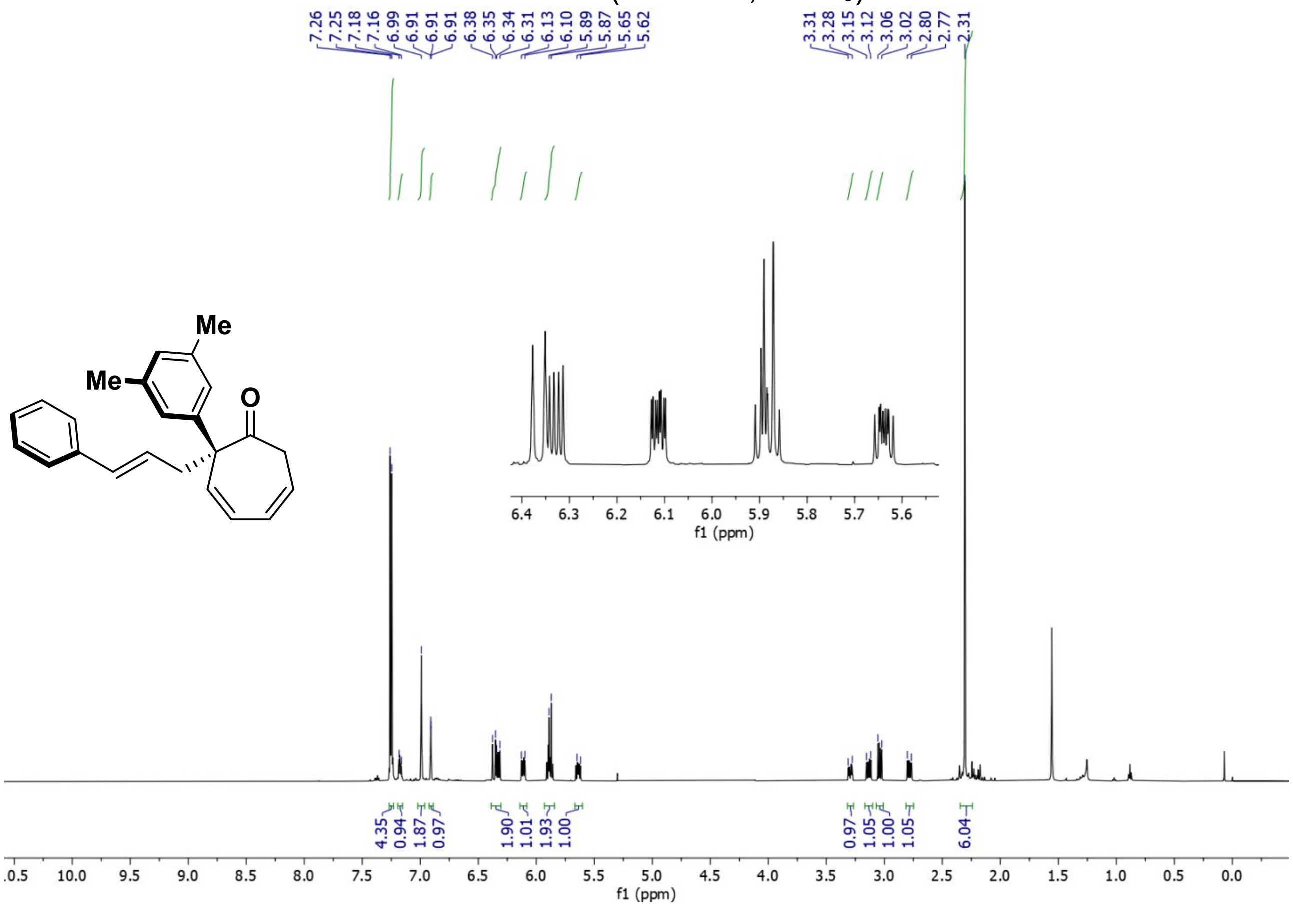

3fa ^{13}C NMR (151 MHz, $CDCl_3$)

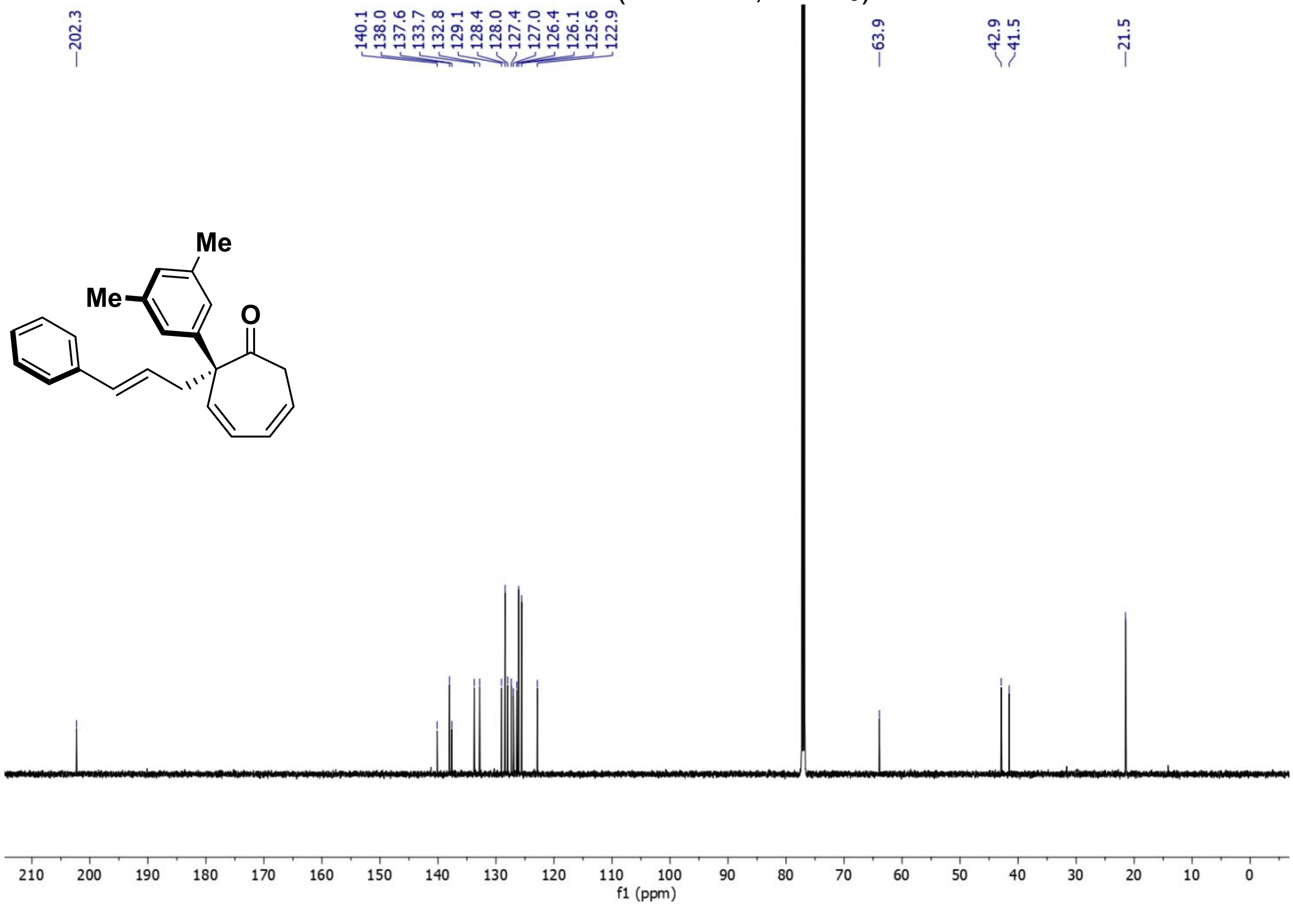

3ga ^{1}H NMR (600 MHz, $CDCl_3$)

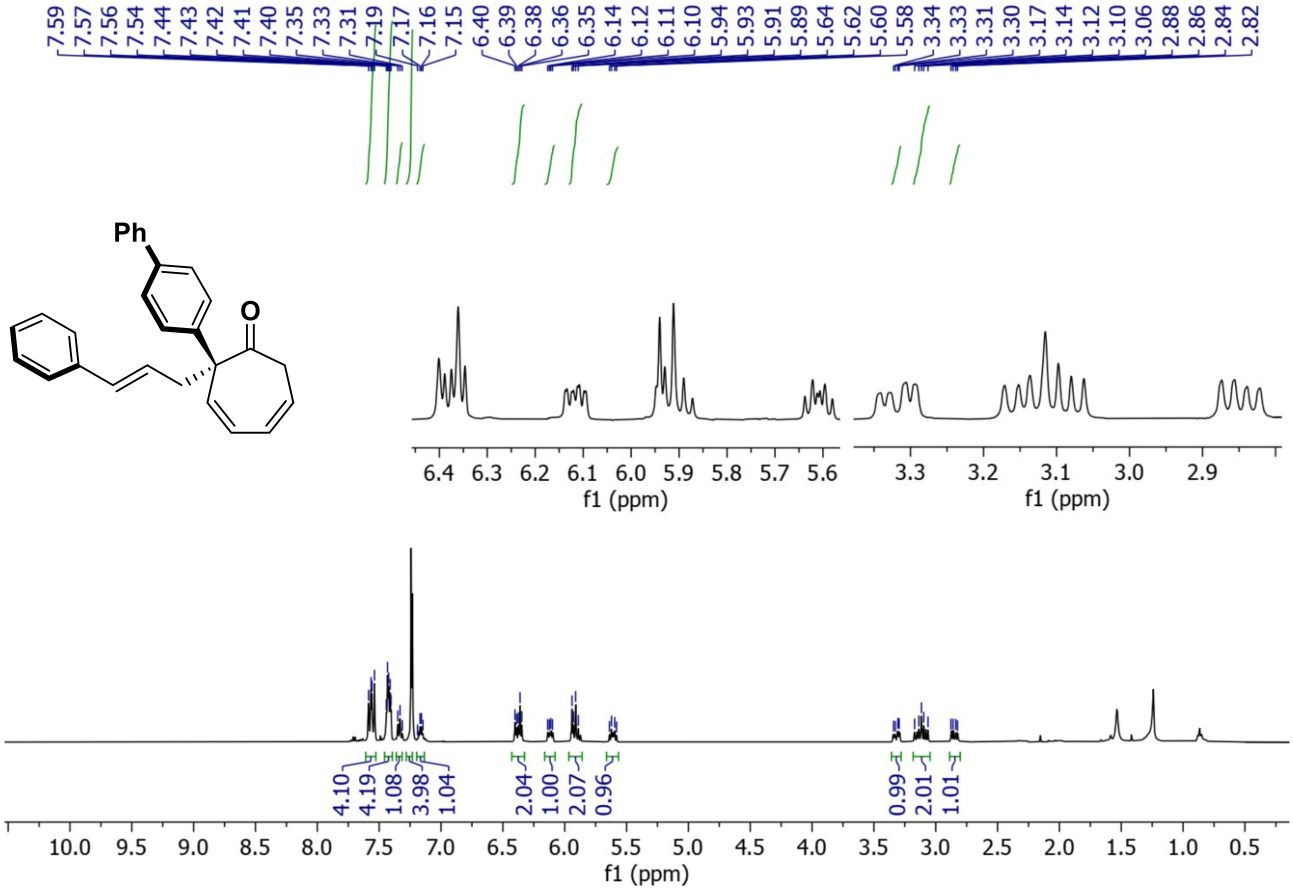

3ga ^{13}C NMR (151 MHz, $CDCl_3$)

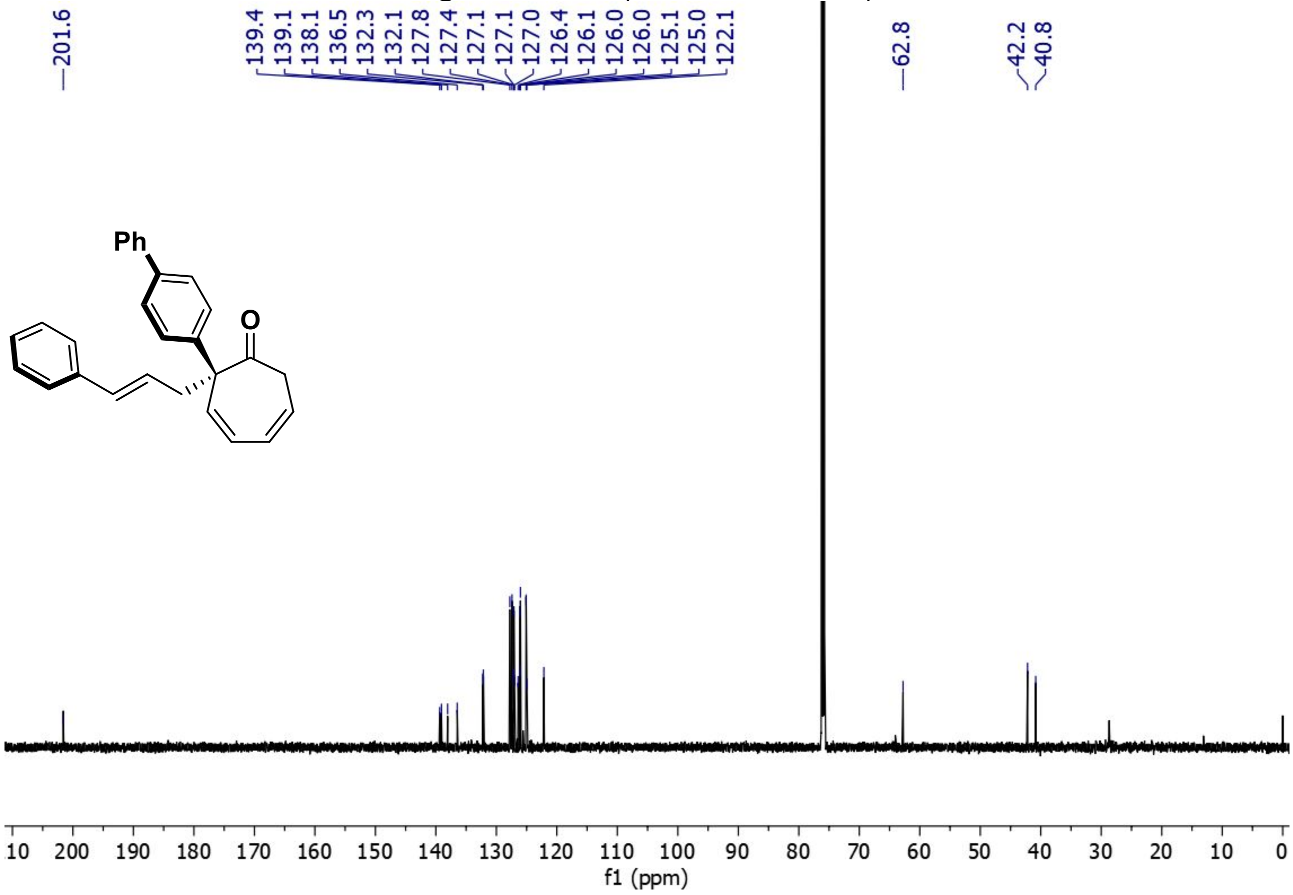

3ha ^{1}**H NMR** (600 MHz, $CDCl_3$)

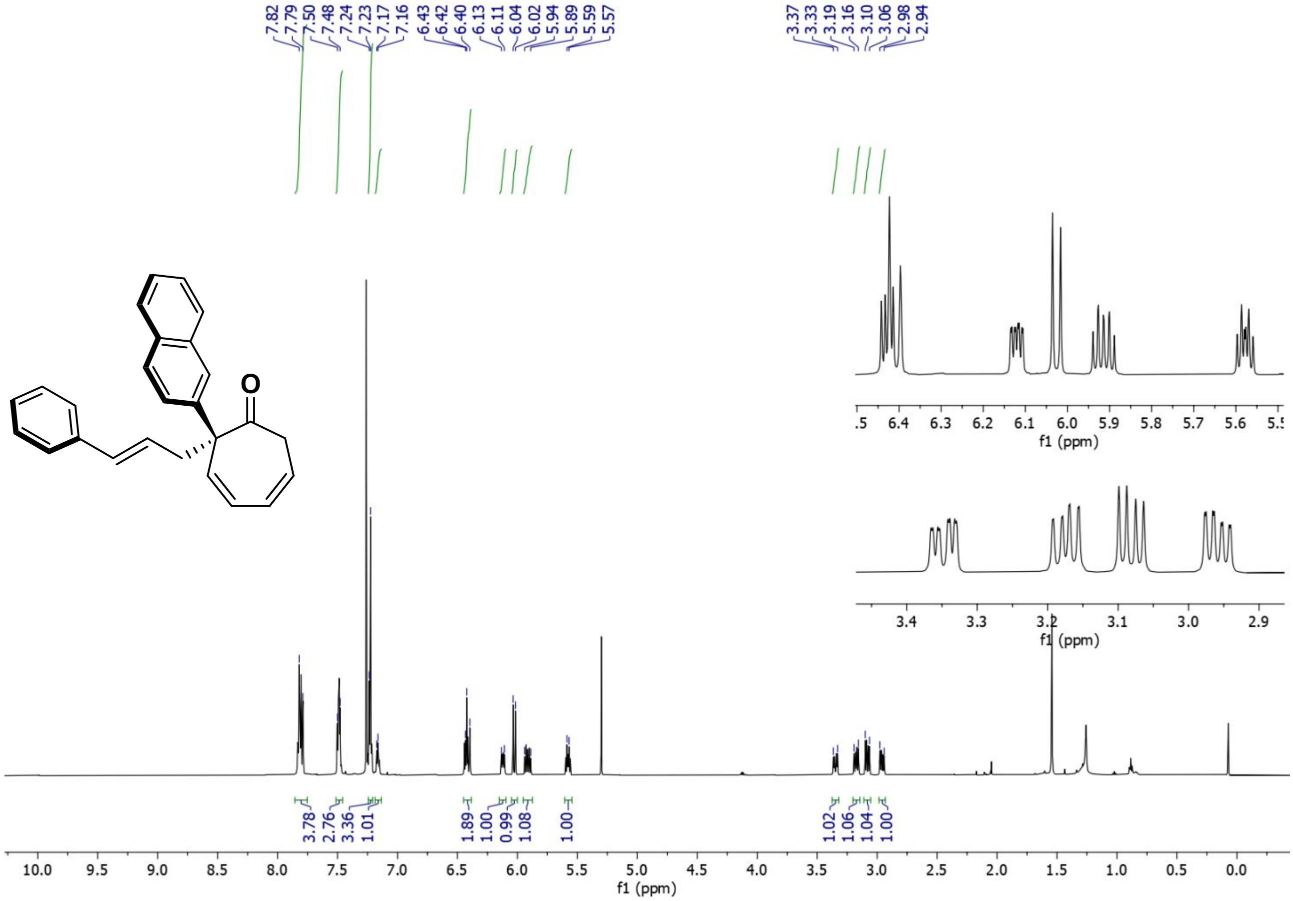

3ha ^{13}**C NMR** (151 MHz, $CDCl_3$)

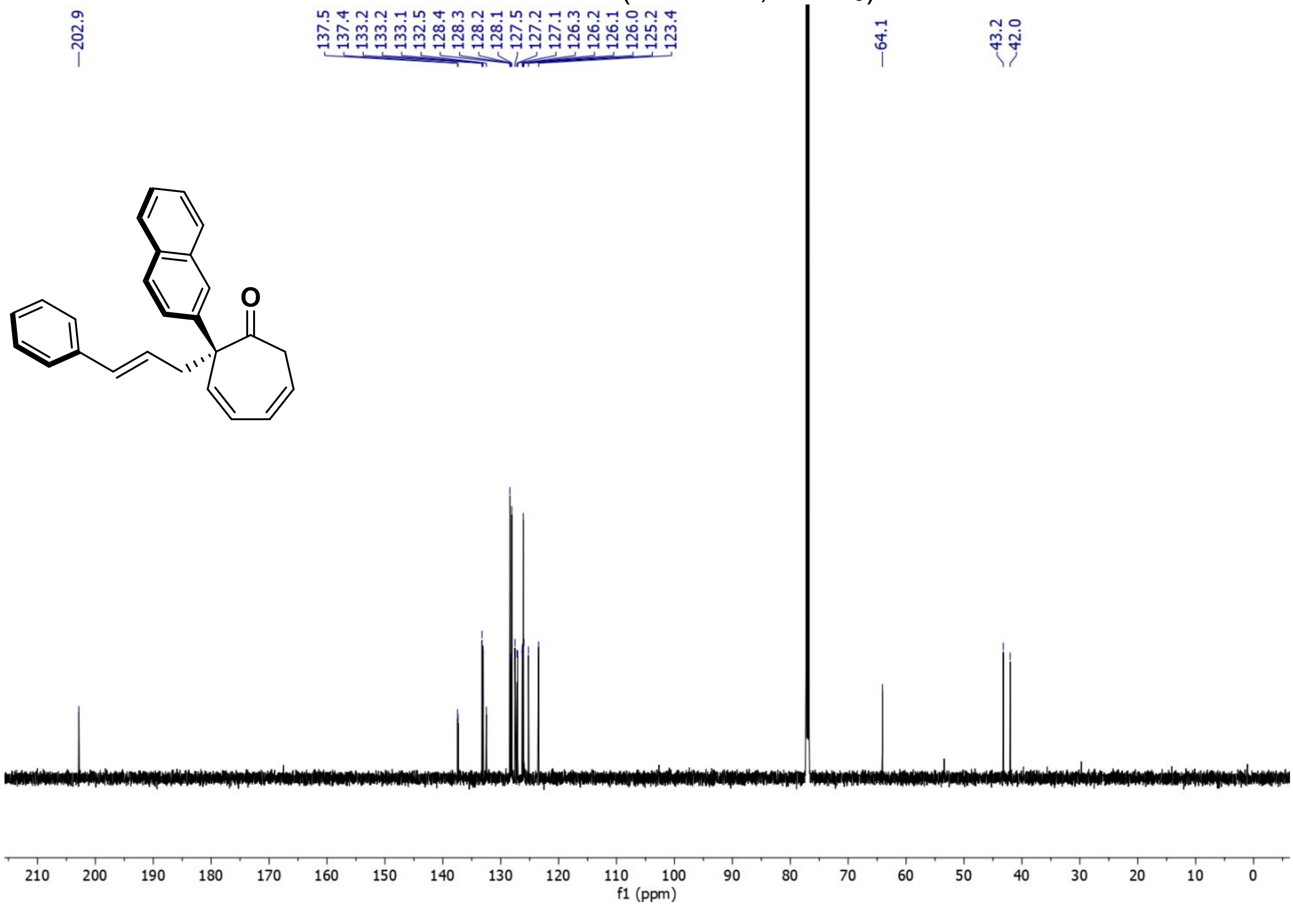

3ef ^{1}H NMR (600 MHz, $CDCl_3$)

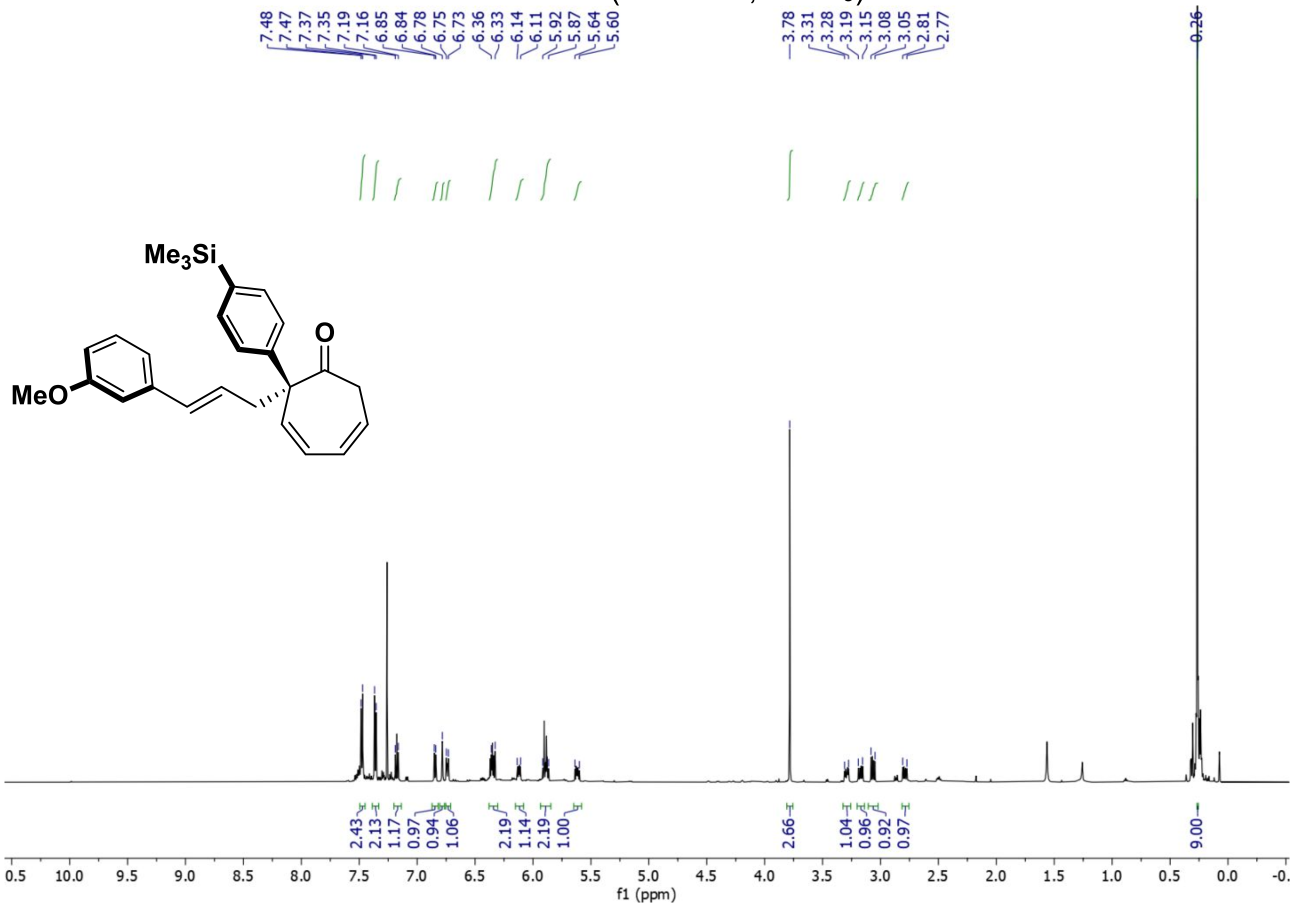

3ef ^{13}C NMR (151 MHz, $CDCl_3$)

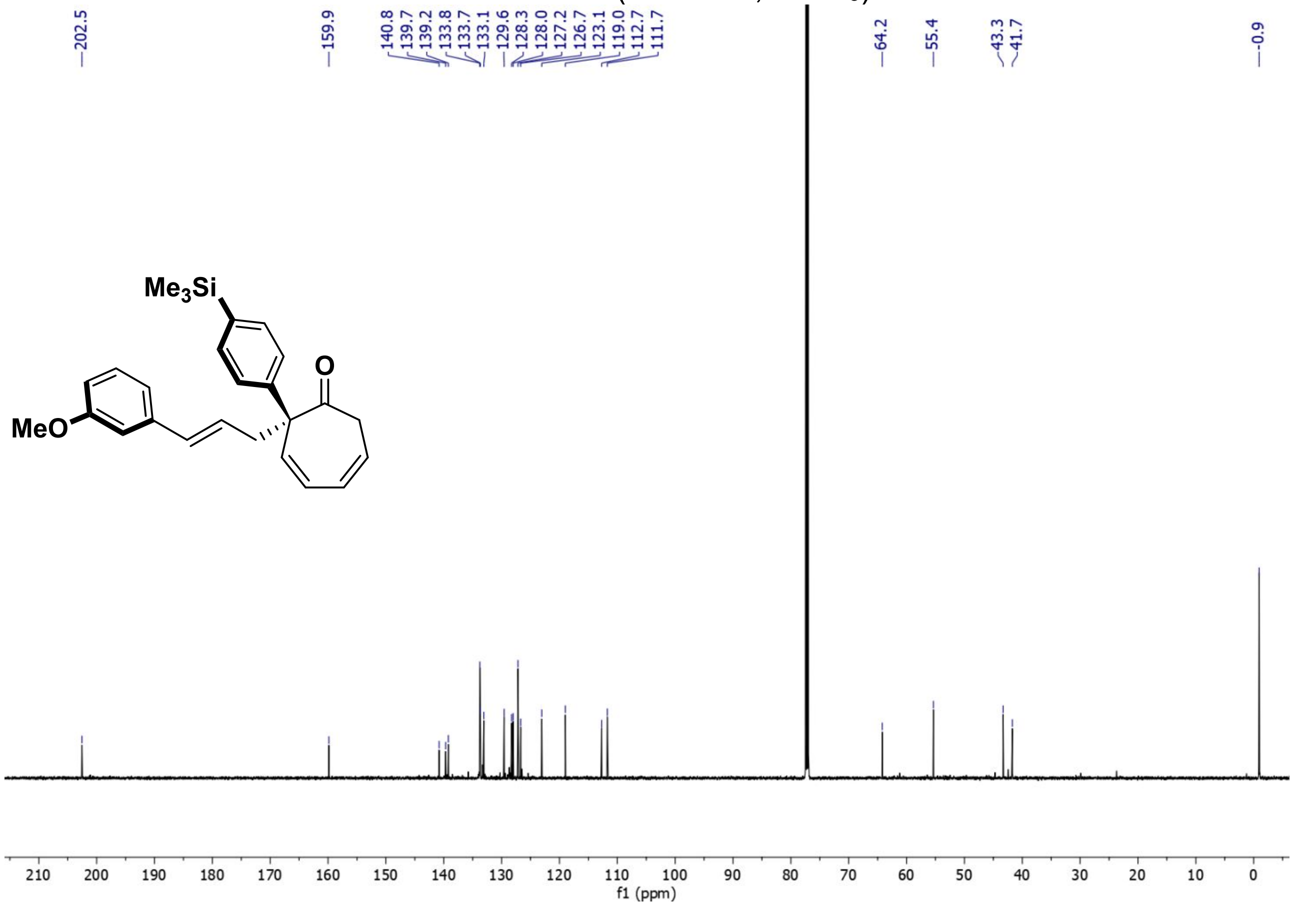

3if ^{1}**H NMR** (600 MHz, $CDCl_3$)

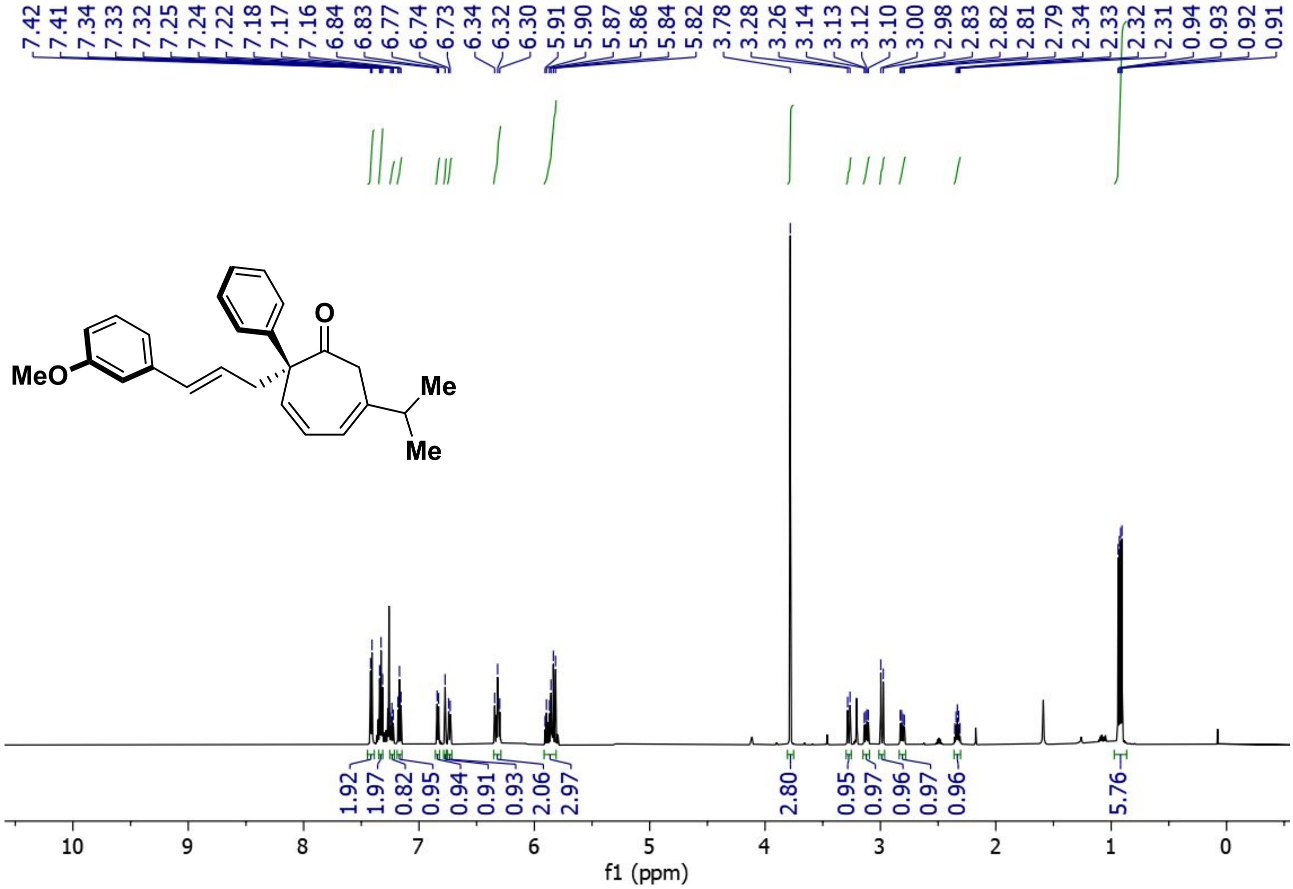

3if ^{13}**C NMR** (151 MHz, $CDCl_3$)

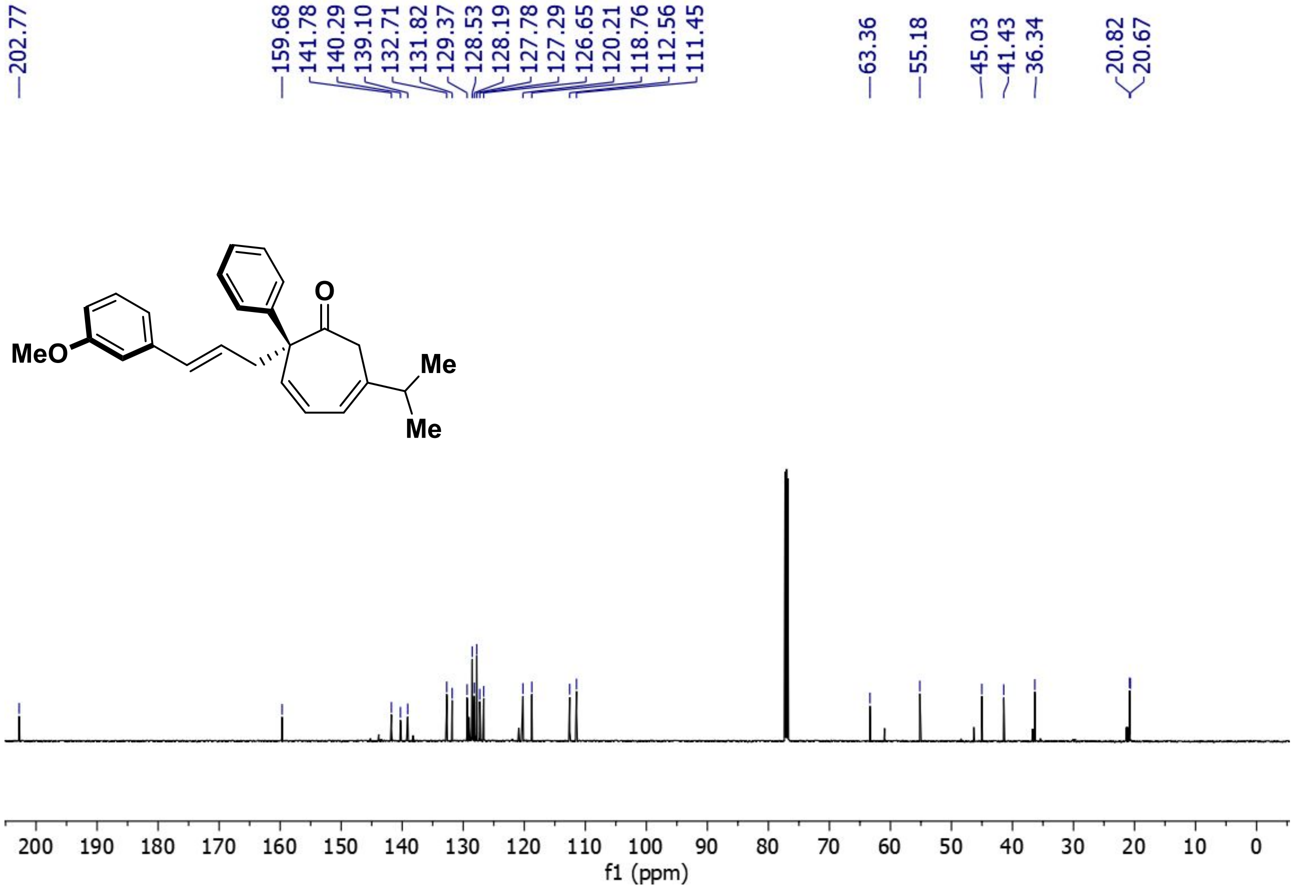

3jf ¹H NMR (600 MHz, CDCl₃)

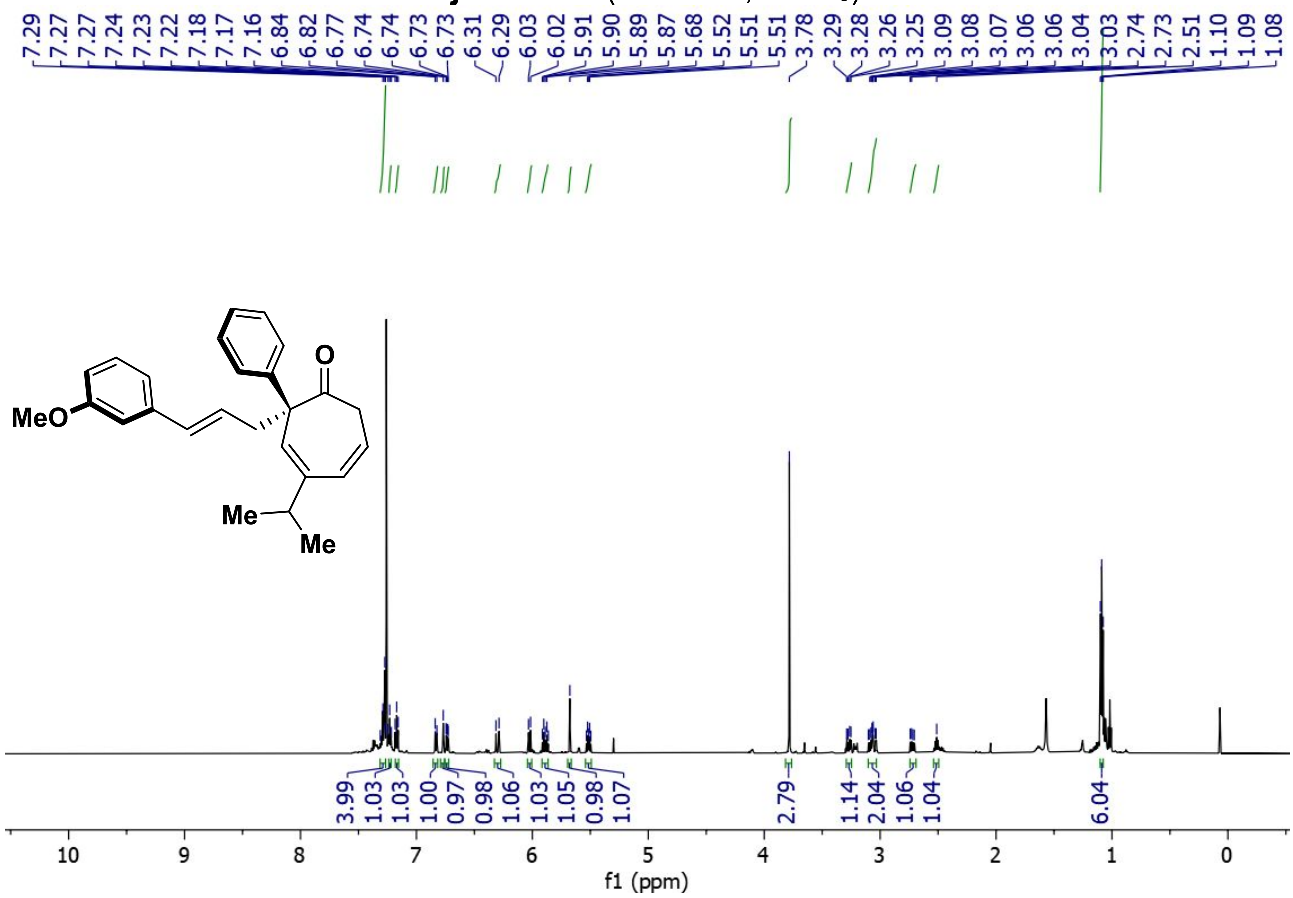

3jf ¹³C NMR (151 MHz, CDCl₃)

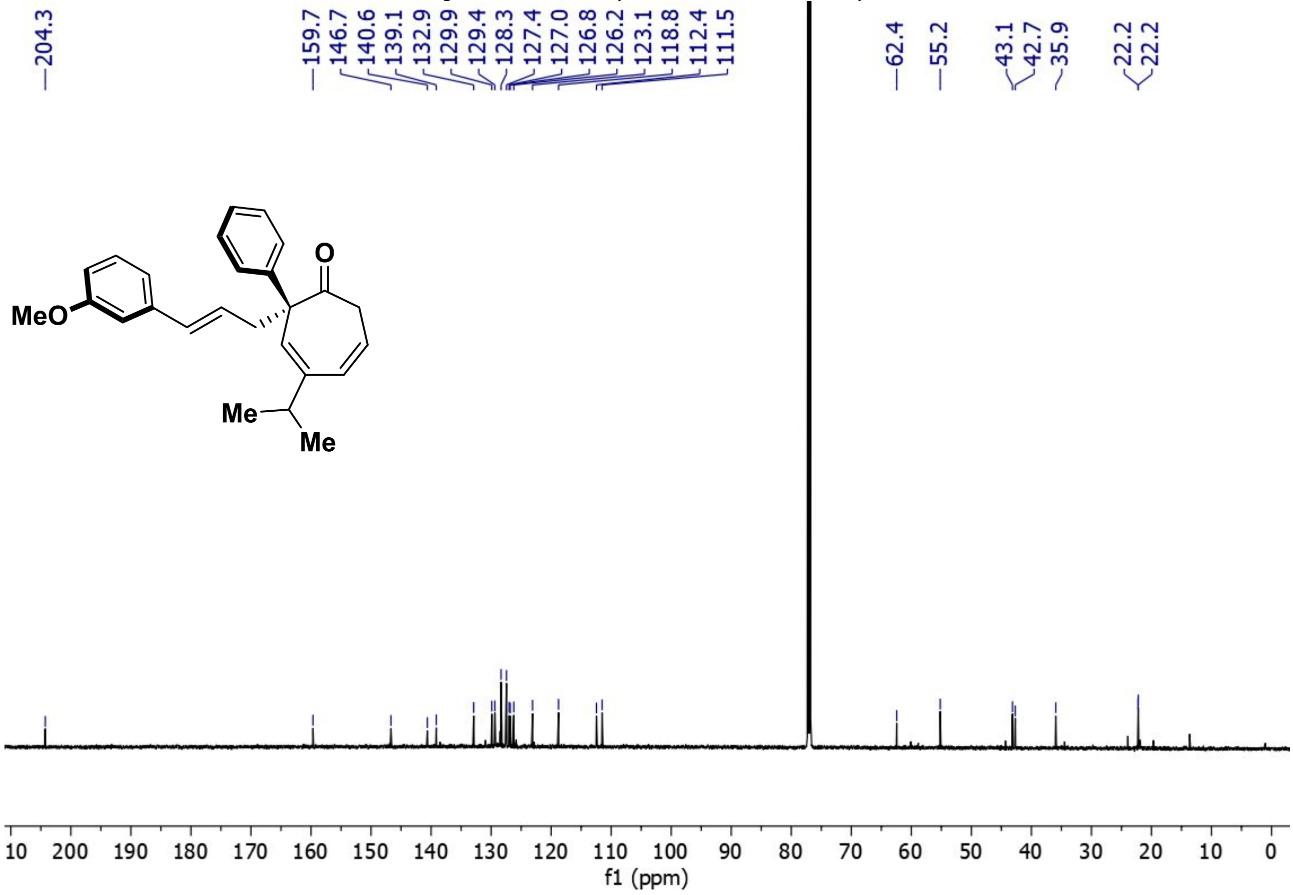

3ka ^{1}H NMR (600 MHz, $CDCl_3$)

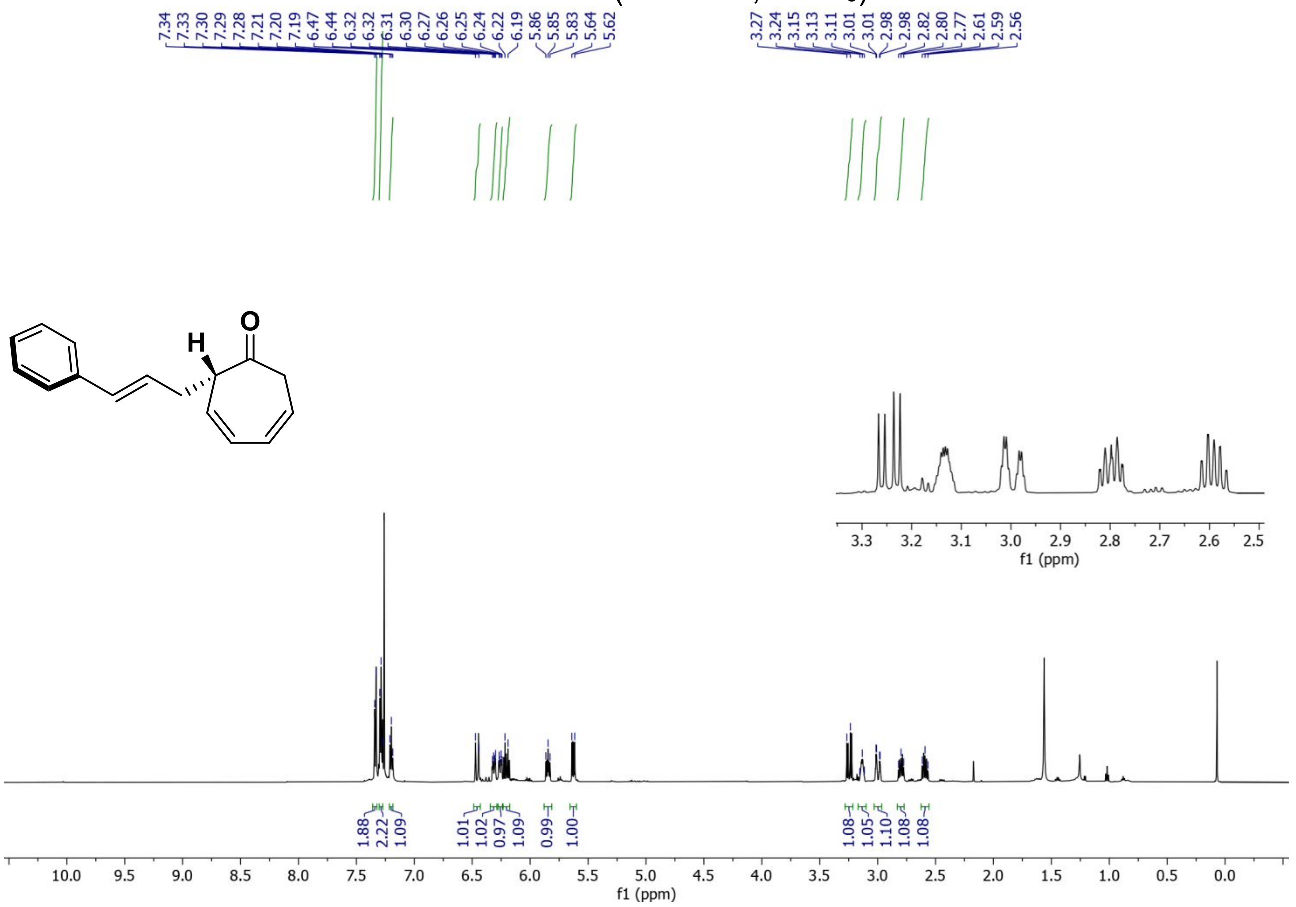

3ka ^{13}C NMR (151 MHz, $CDCl_3$)

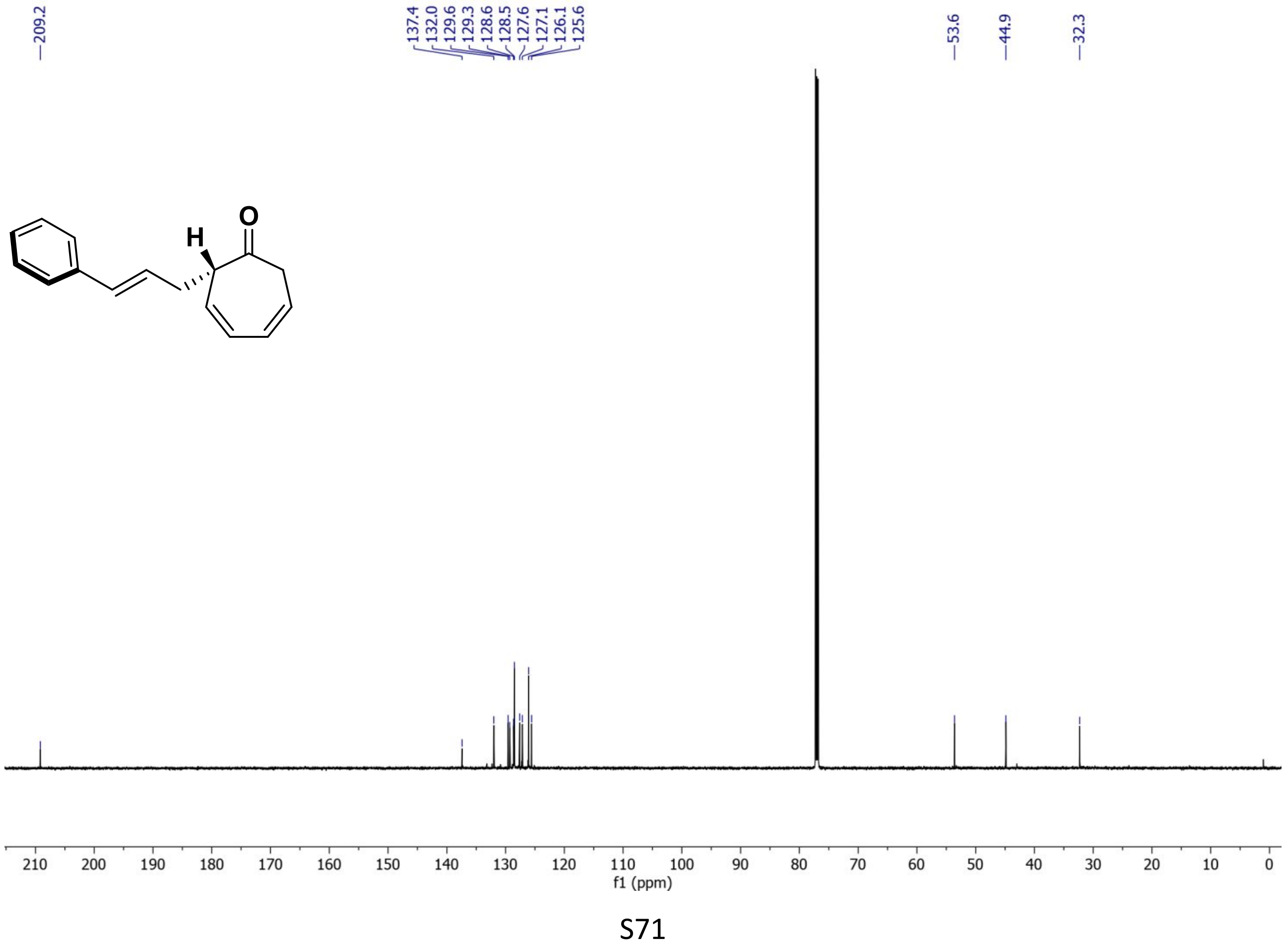

4 ¹H NMR (600 MHz, CDCl$_3$)

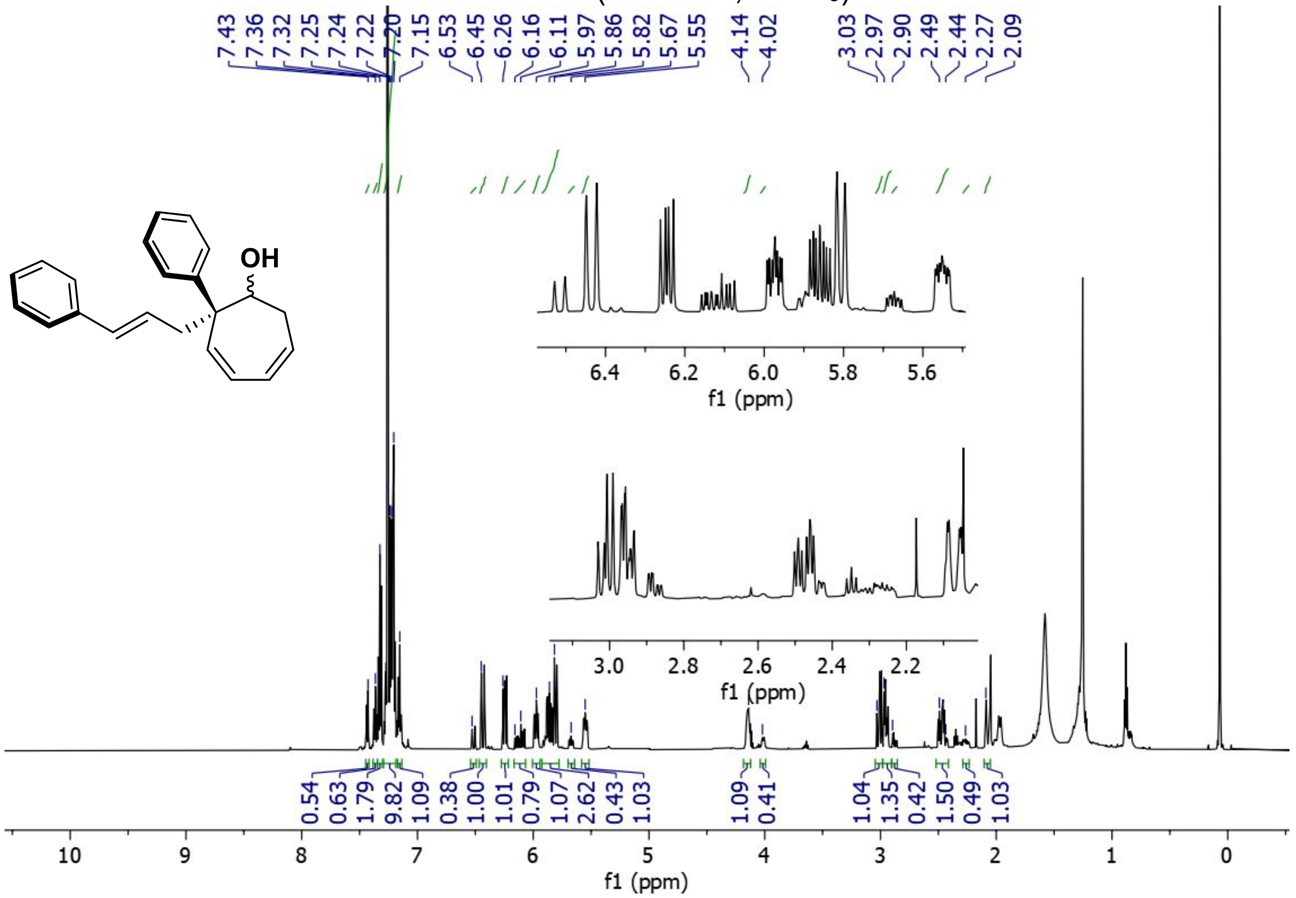

4 ¹³C NMR (151 MHz, CDCl$_3$)

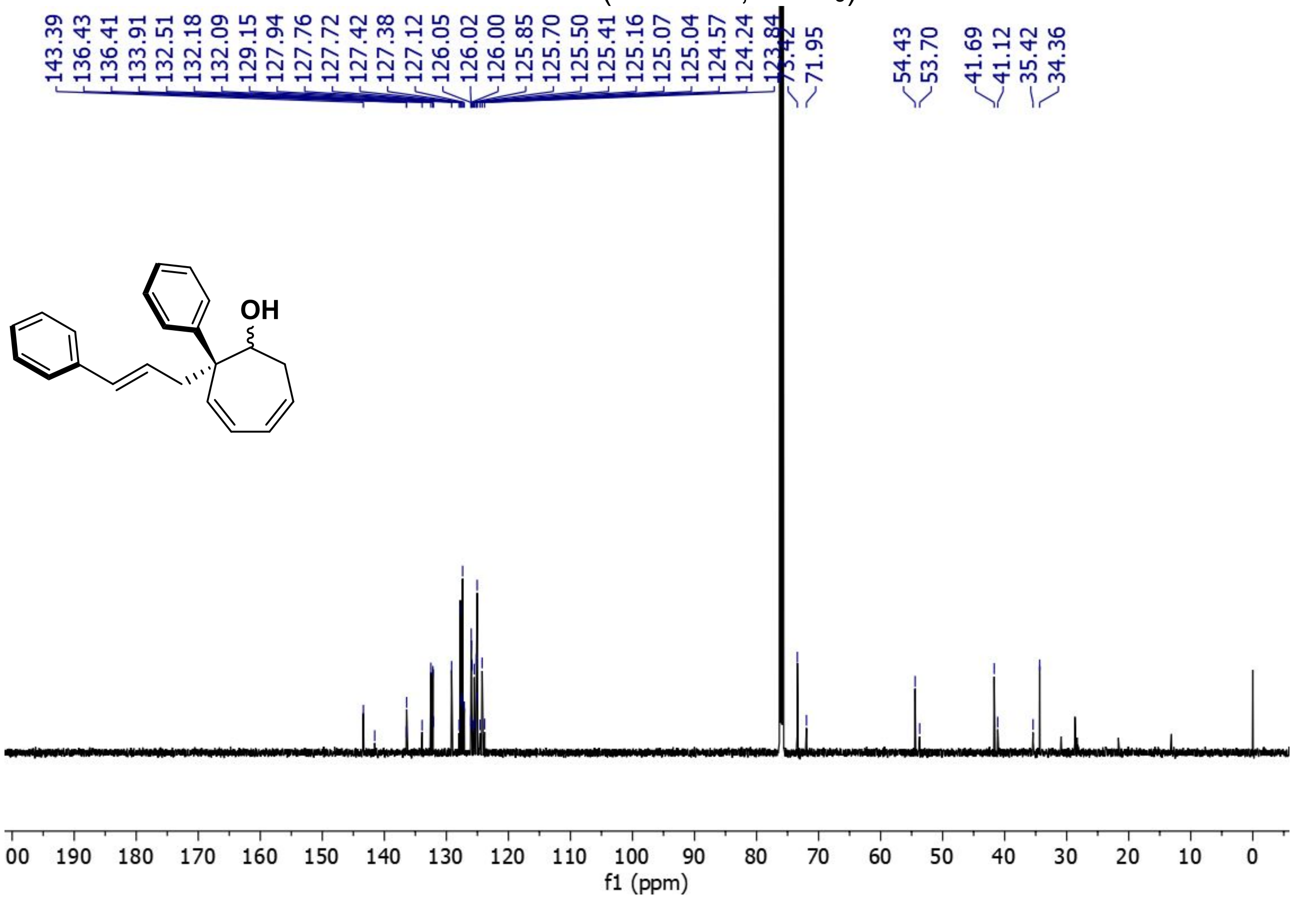

5 ^{1}H NMR (600 MHz, $CDCl_3$)

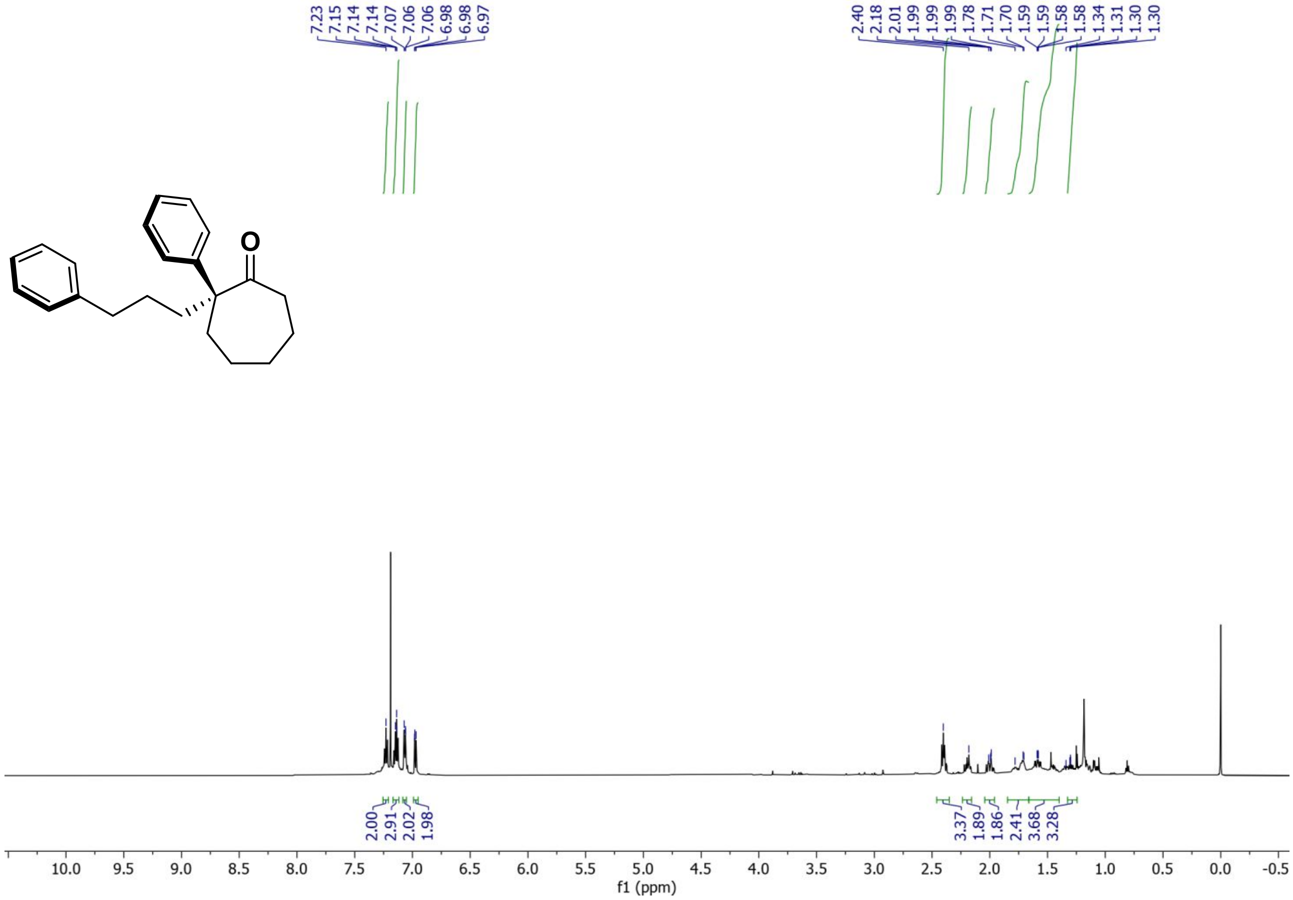

5 ^{13}C NMR (151 MHz, $CDCl_3$)

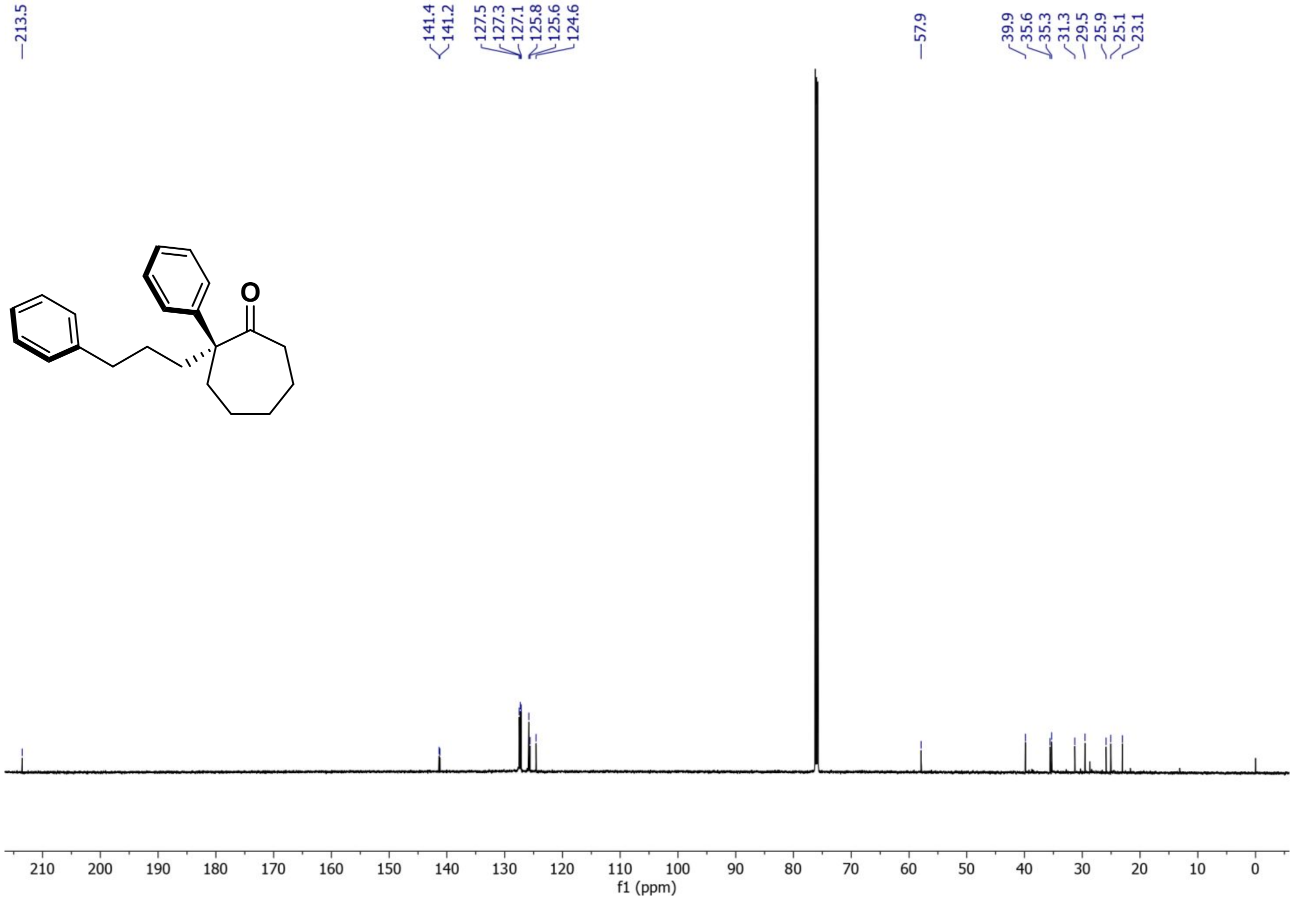

11. Chiral Stationary Phase HPLC Traces

Compound **3aa**

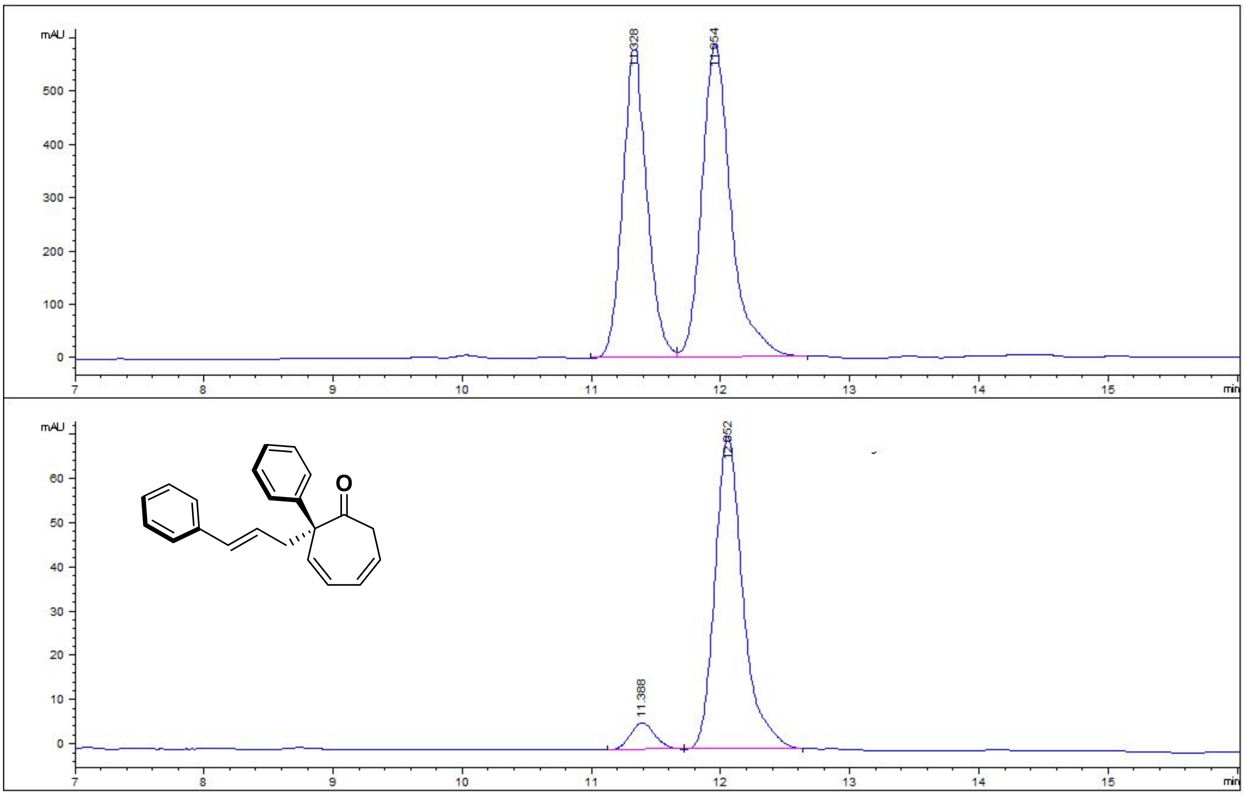

Peak	RetTime [min]	Width [min]	Area%
1	11.328	0.2122	49.5679
2	11.954	0.2378	50.4321

Peak	RetTime [min]	Width [min]	Area%
1	11.388	0.2057	5.9382
2	12.052	0.2326	94.0618

Compound **3ab**

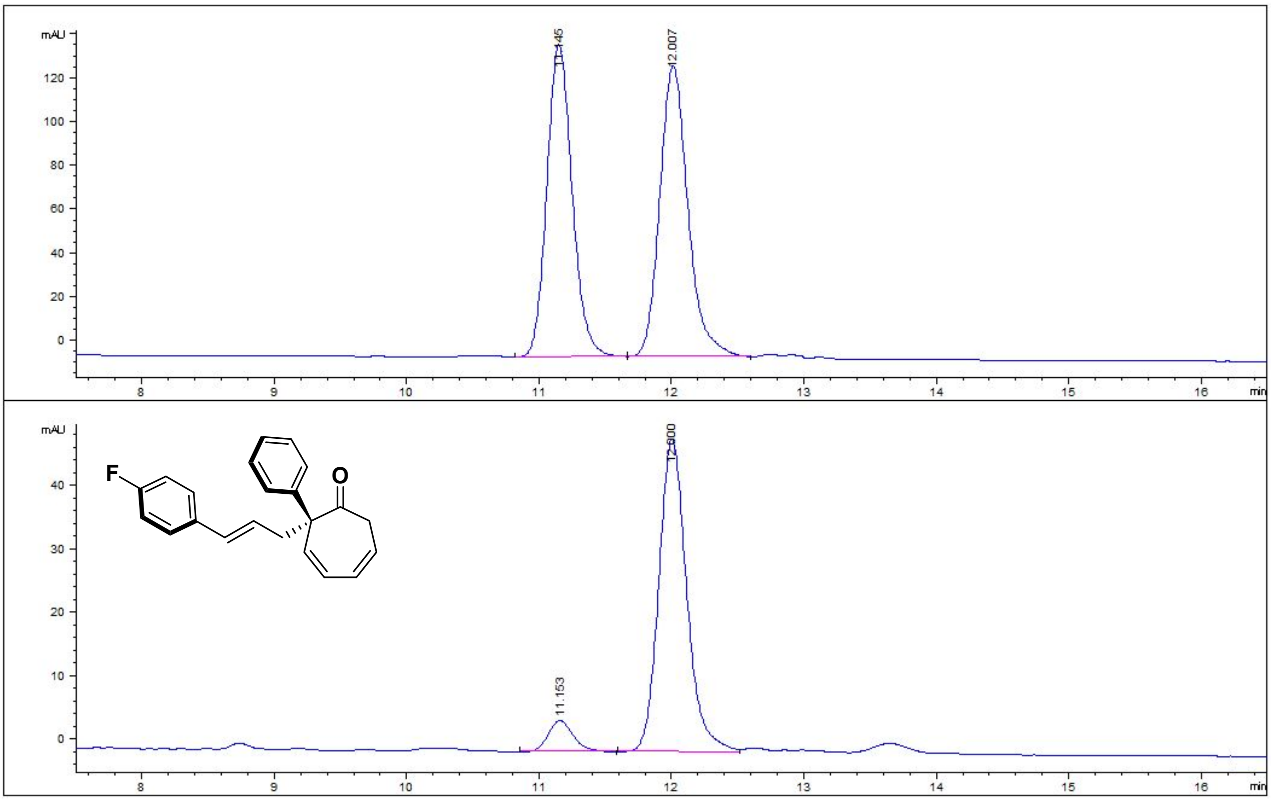

Peak	RetTime [min]	Width [min]	Area%
1	11.145	0.2047	49.1156
2	12.007	0.2261	50.8844

Peak	RetTime [min]	Width [min]	Area%
1	11.153	0.2053	8.2837
2	12.000	0.2258	91.7163

Compound **3ac**

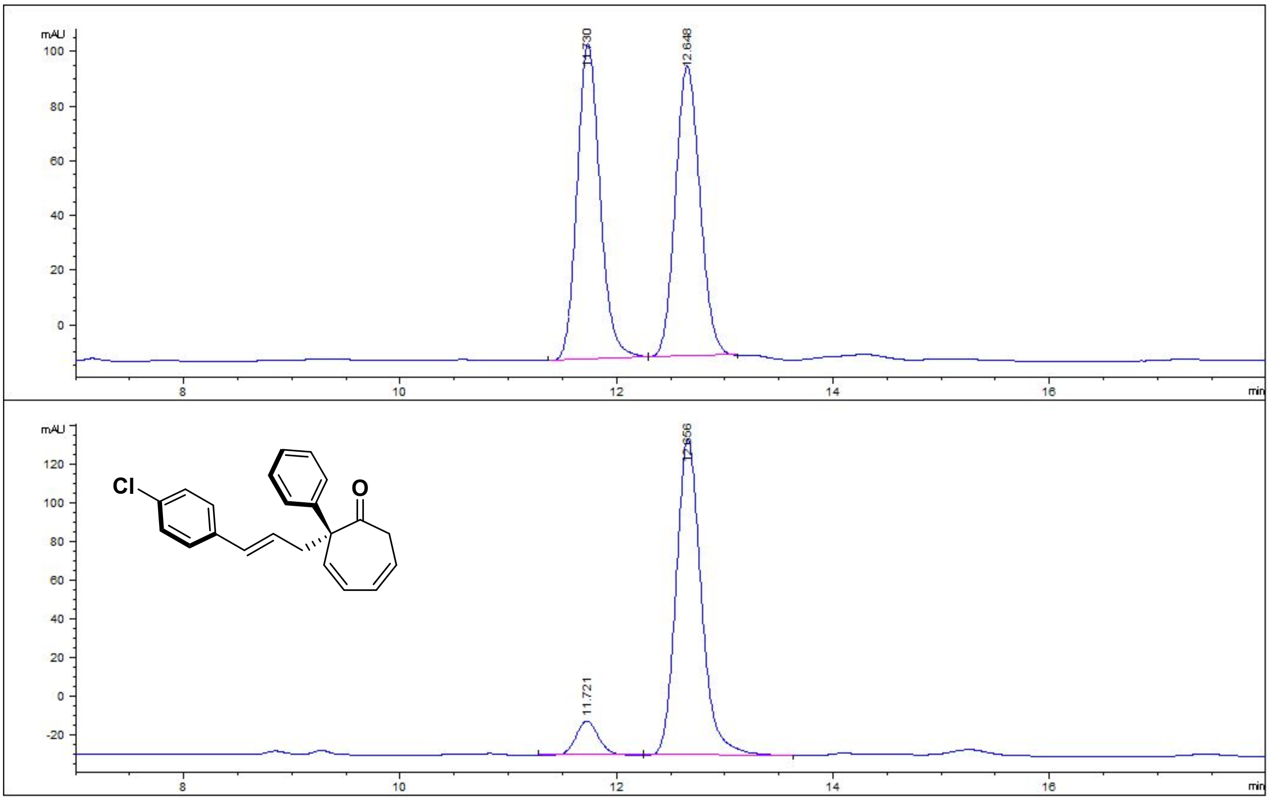

Peak	RetTime [min]	Width [min]	Area%
1	11.730	0.2285	50.4863
2	12.648	0.2457	49.5137

Peak	RetTime [min]	Width [min]	Area%
1	11.721	0.2250	8.9202
2	12.656	0.2495	91.0798

Compound **3ad**

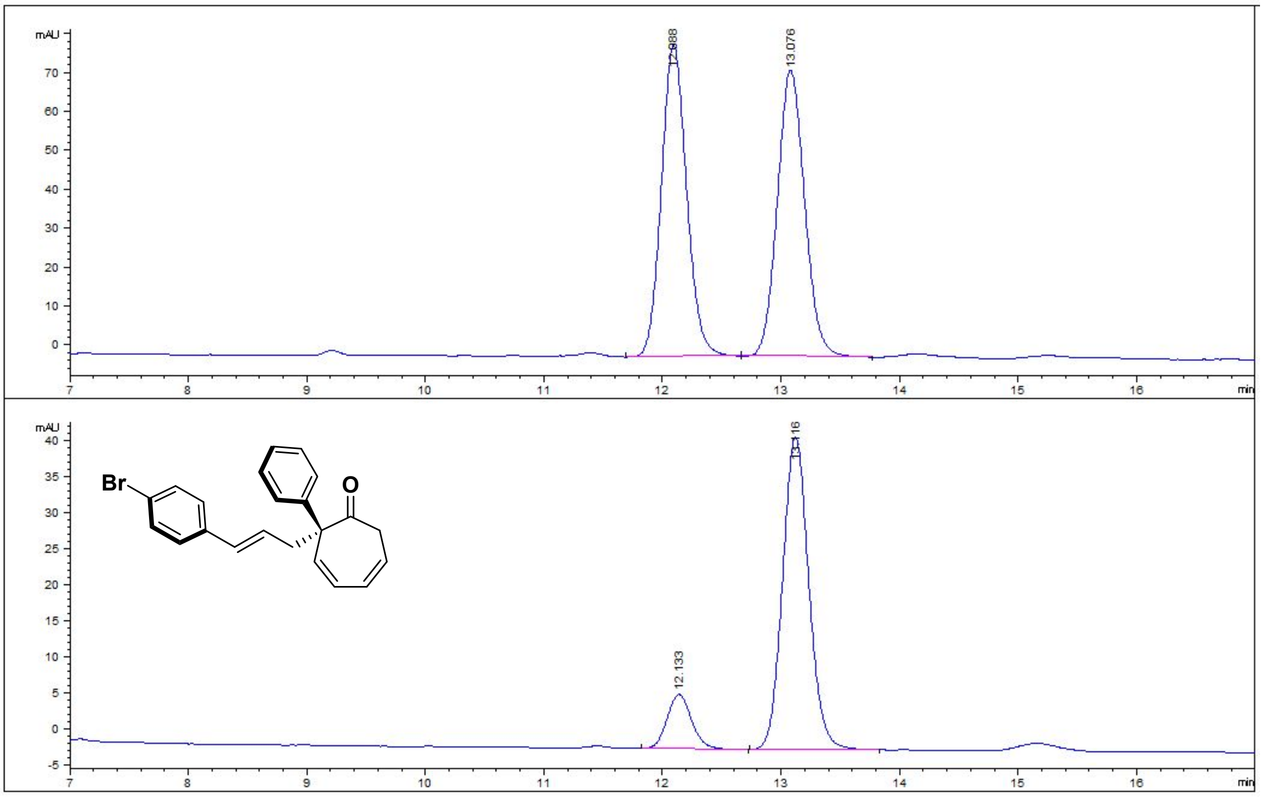

Peak	RetTime [min]	Width [min]	Area%
1	12.088	0.2287	50.2216
2	13.076	0.2487	49.7784

Peak	RetTime [min]	Width [min]	Area%
1	12.133	0.2243	14.0621
2	13.116	0.2392	85.9379

Compound **3ae**

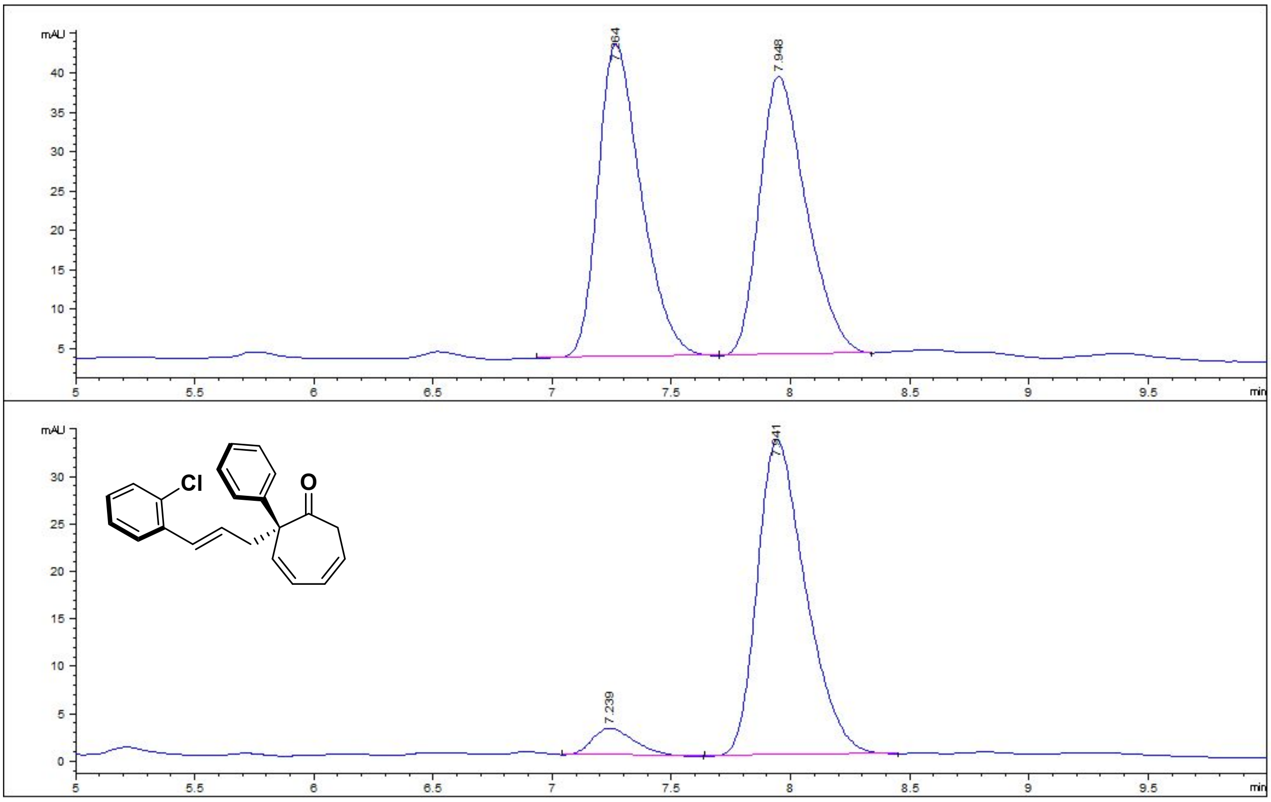

Peak	RetTime [min]	Width [min]	Area%
1	7.264	0.1907	50.8501
2	7.948	0.2073	49.1499

Peak	RetTime [min]	Width [min]	Area%
1	7.239	0.1868	6.9889
2	7.941	0.2148	93.0111

Compound **3af**

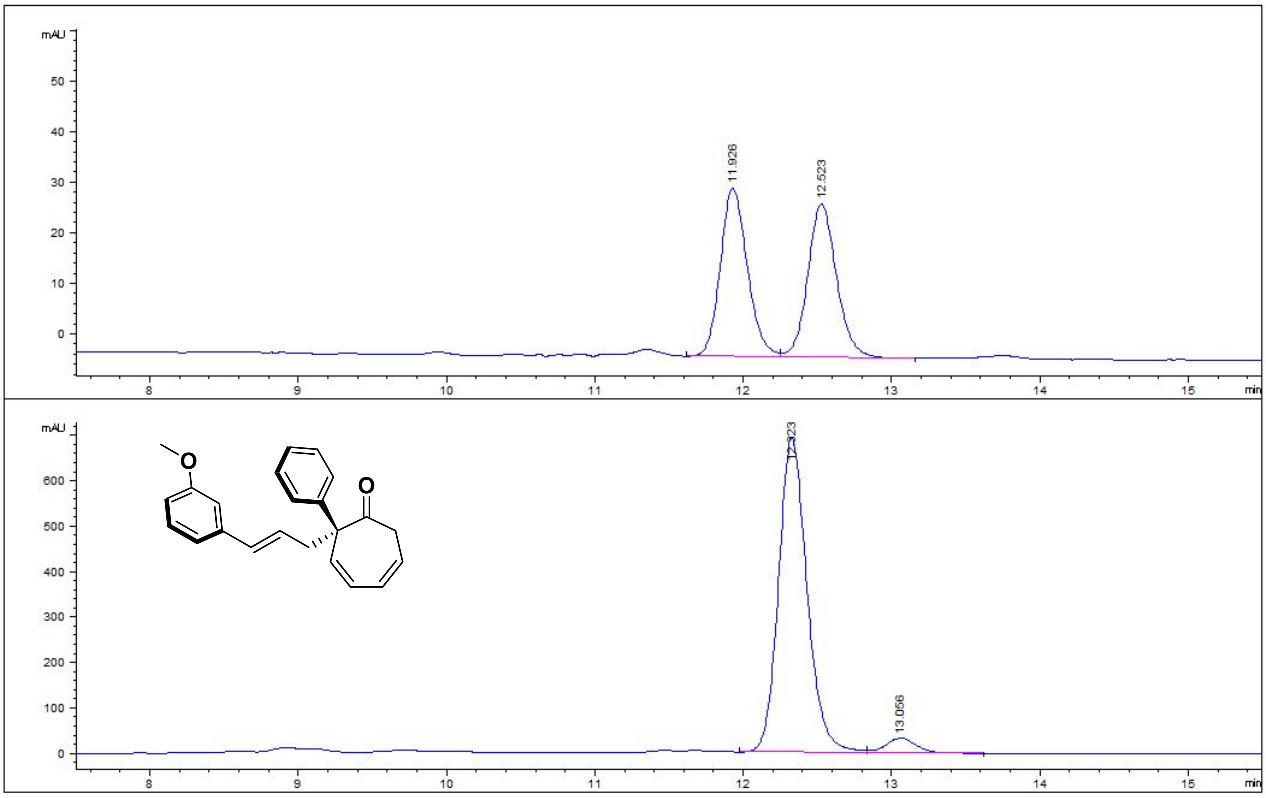

Peak	RetTime [min]	Width [min]	Area%
1	11.926	0.1939	50.8196
2	12.523	0.2052	49.1804

Peak	RetTime [min]	Width [min]	Area%
1	12.323	0.2037	95.2237
2	13.056	0.2177	4.7763

Compound **3ag**

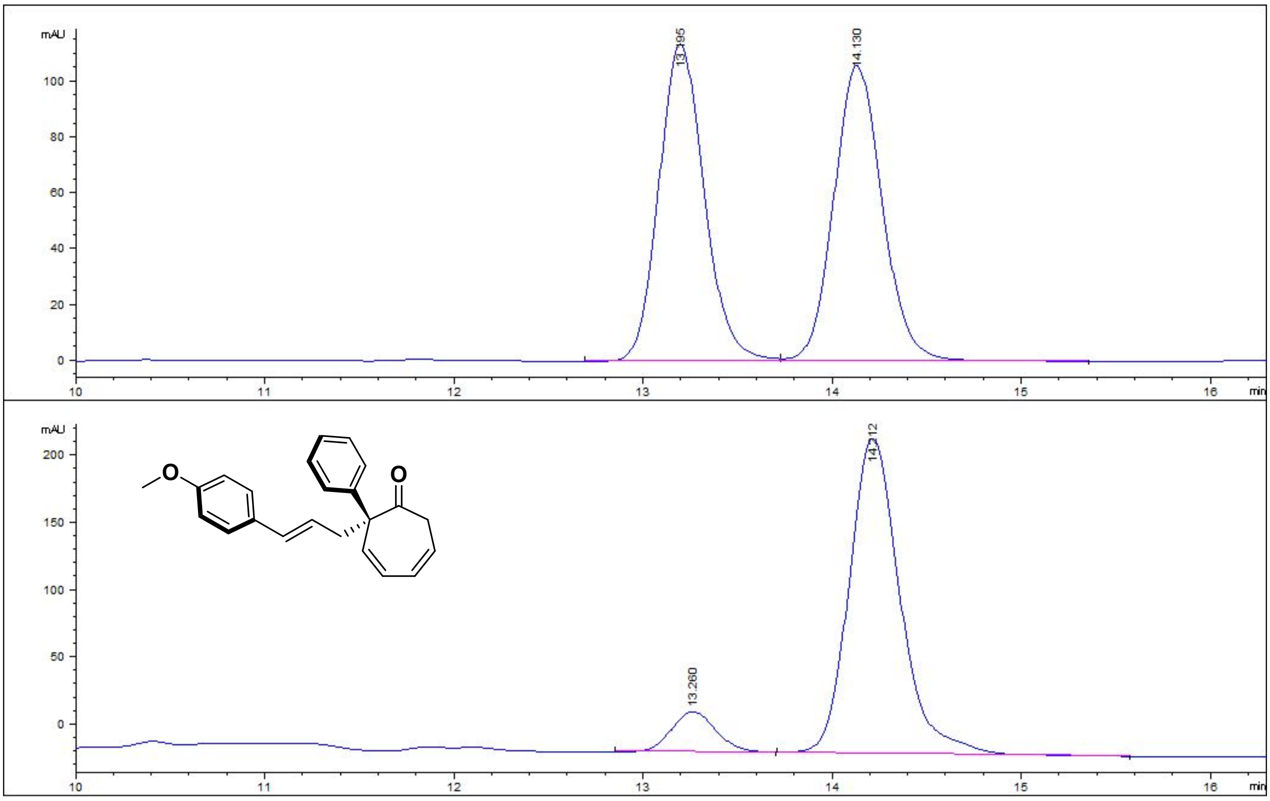

Peak	RetTime [min]	Width [min]	Area%
1	13.195	0.2585	50.0927
2	14.129	0.2737	49.9073

Peak	RetTime [min]	Width [min]	Area%
1	13.260	0.2555	10.1385
2	14.212	0.2878	89.8615

Compound **3ah**

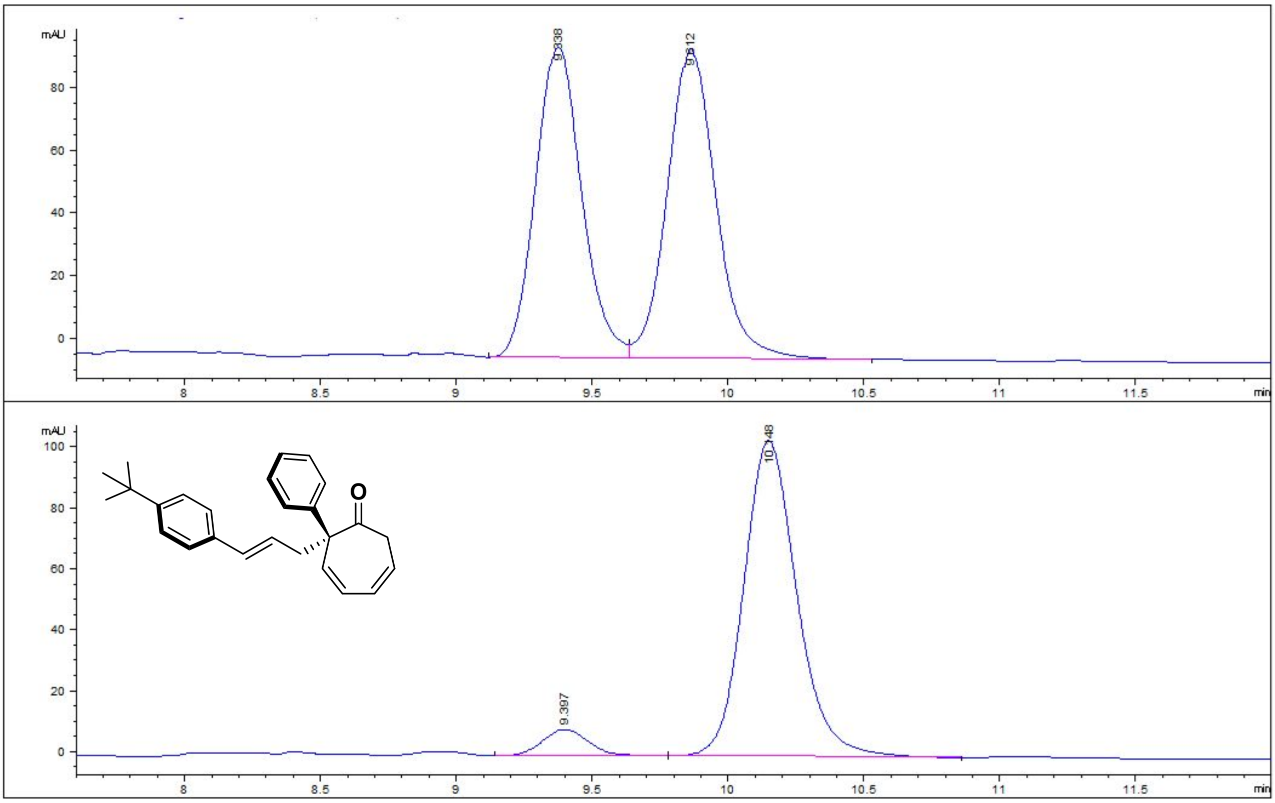

Peak	RetTime [min]	Width [min]	Area%
1	8.338	0.1707	44.5454
2	9.812	0.2060	55.4546

Peak	RetTime [min]	Width [min]	Area%
1	9.397	0.1866	7.0822
2	10.148	0.2089	92.9178

Compound **3ai**

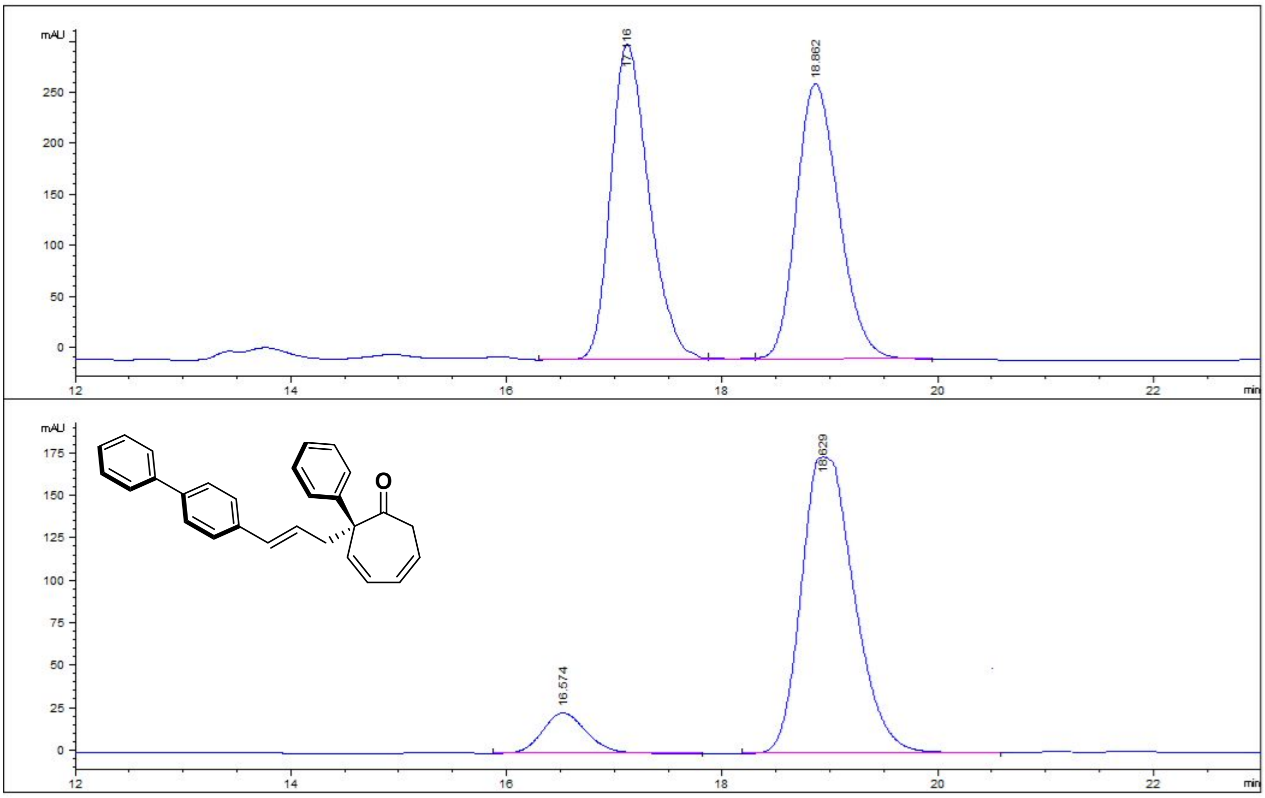

Peak	RetTime [min]	Width [min]	Area%
1	17.116	0.3875	51.7993
2	18.862	0.4332	48.2007

Peak	RetTime [min]	Width [min]	Area%
1	16.474	0.4416	10.1555
2	18.629	0.5043	89.8445

Compound **3aj**

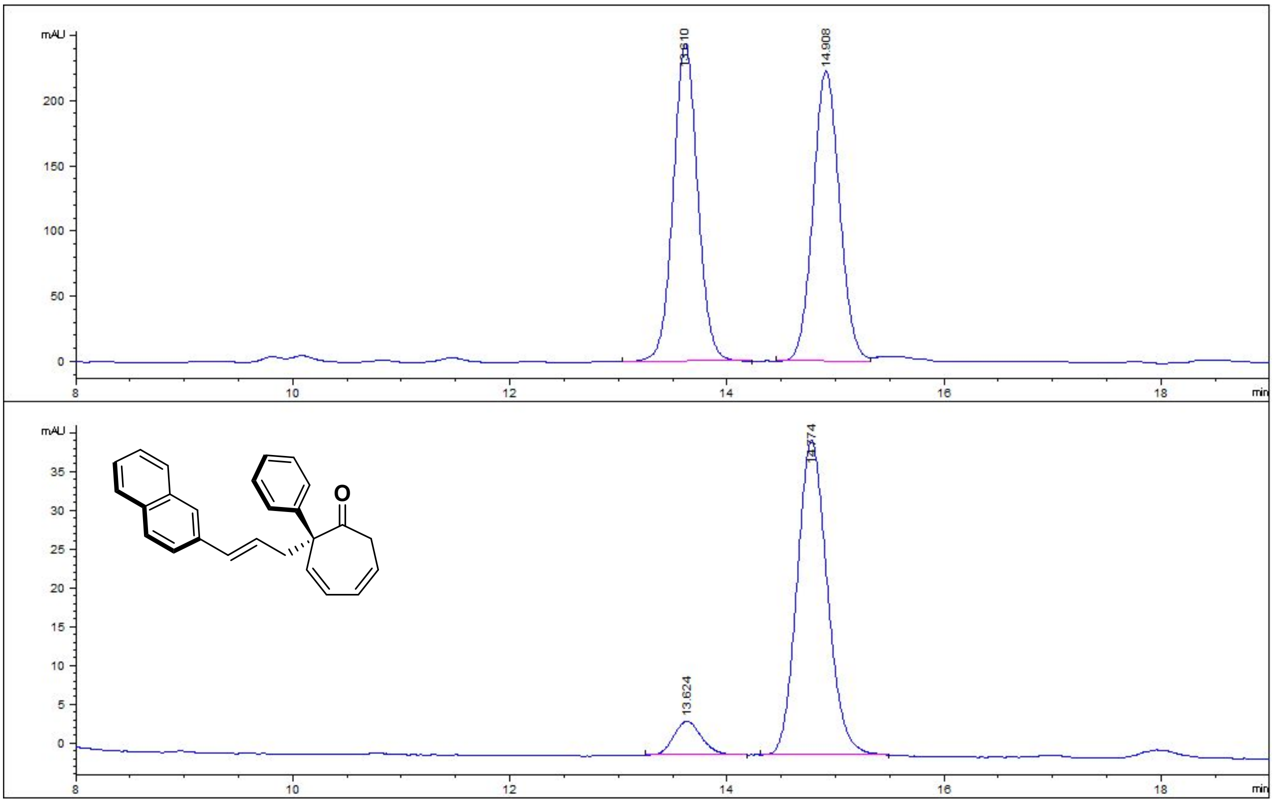

Peak	RetTime [min]	Width [min]	Area%
1	13.610	0.2439	50.3762
2	14.908	0.2621	49.6238

Peak	RetTime [min]	Width [min]	Area%
1	13.624	0.2287	9.0733
2	14.774	0.2487	90.9267

Compound **3ak**

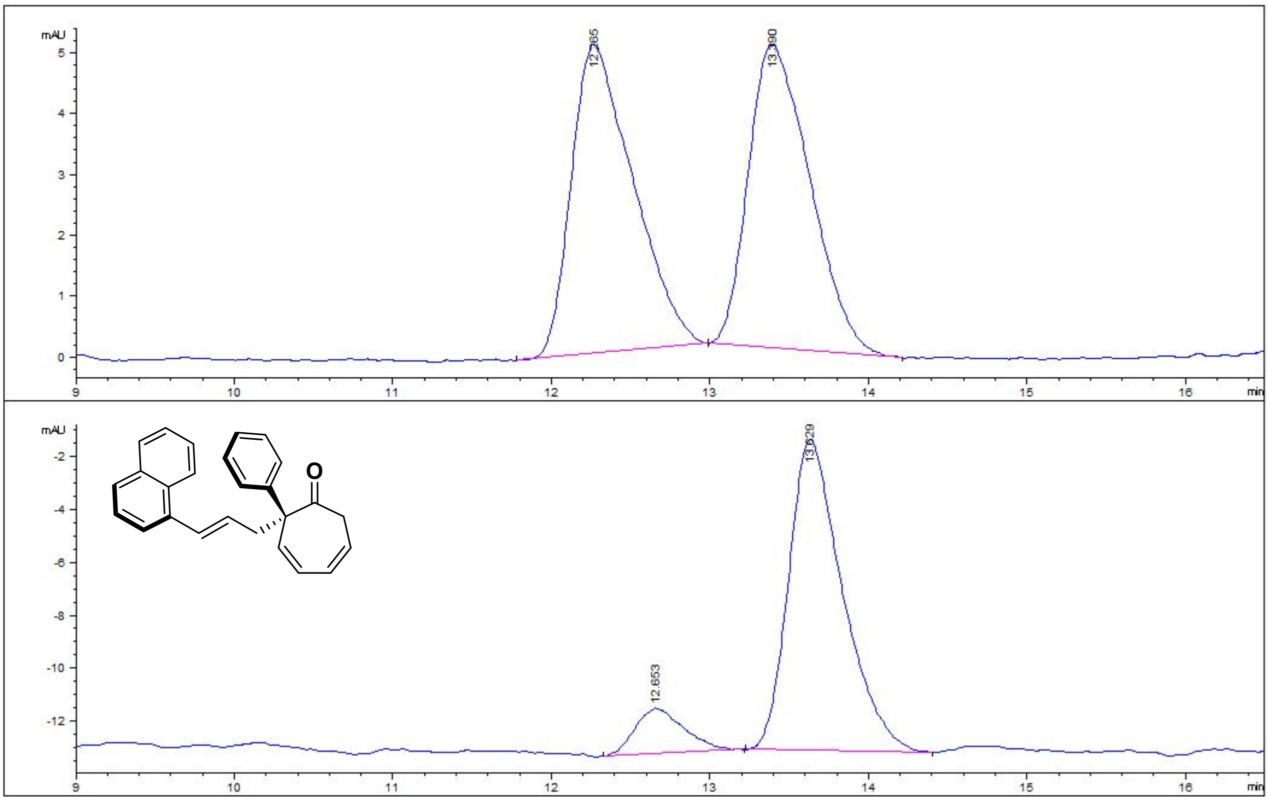

Peak	RetTime [min]	Width [min]	Area%
1	12.265	0.3798	50.3641
2	13.390	0.3910	49.6359

Peak	RetTime [min]	Width [min]	Area%
1	12.653	0.3403	11.9753
2	13.629	0.3615	88.0247

Compound **3al**

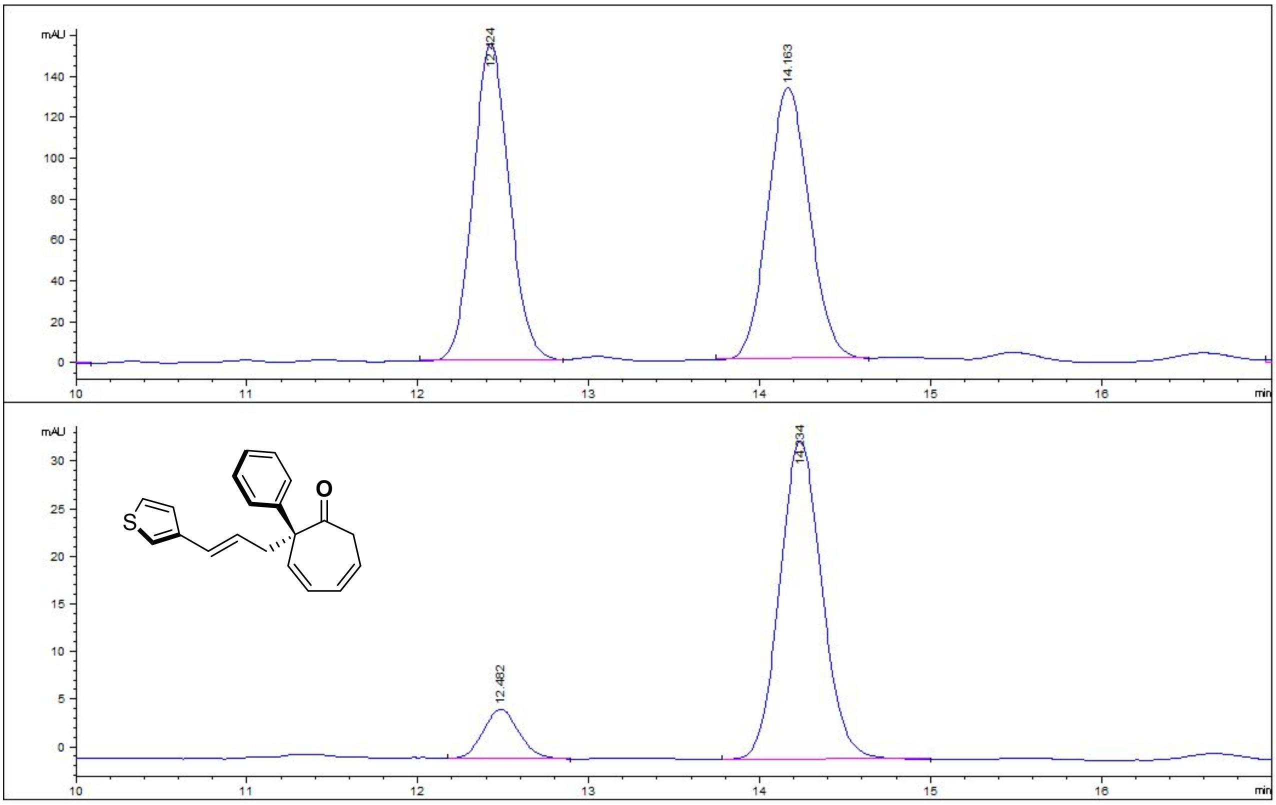

Peak	RetTime [min]	Width [min]	Area%
1	12.424	0.2280	50.4786
2	14.163	0.2652	49.5214

Peak	RetTime [min]	Width [min]	Area%
1	12.482	0.2250	11.7052
2	14.234	0.2661	88.2948

Compound **3am**

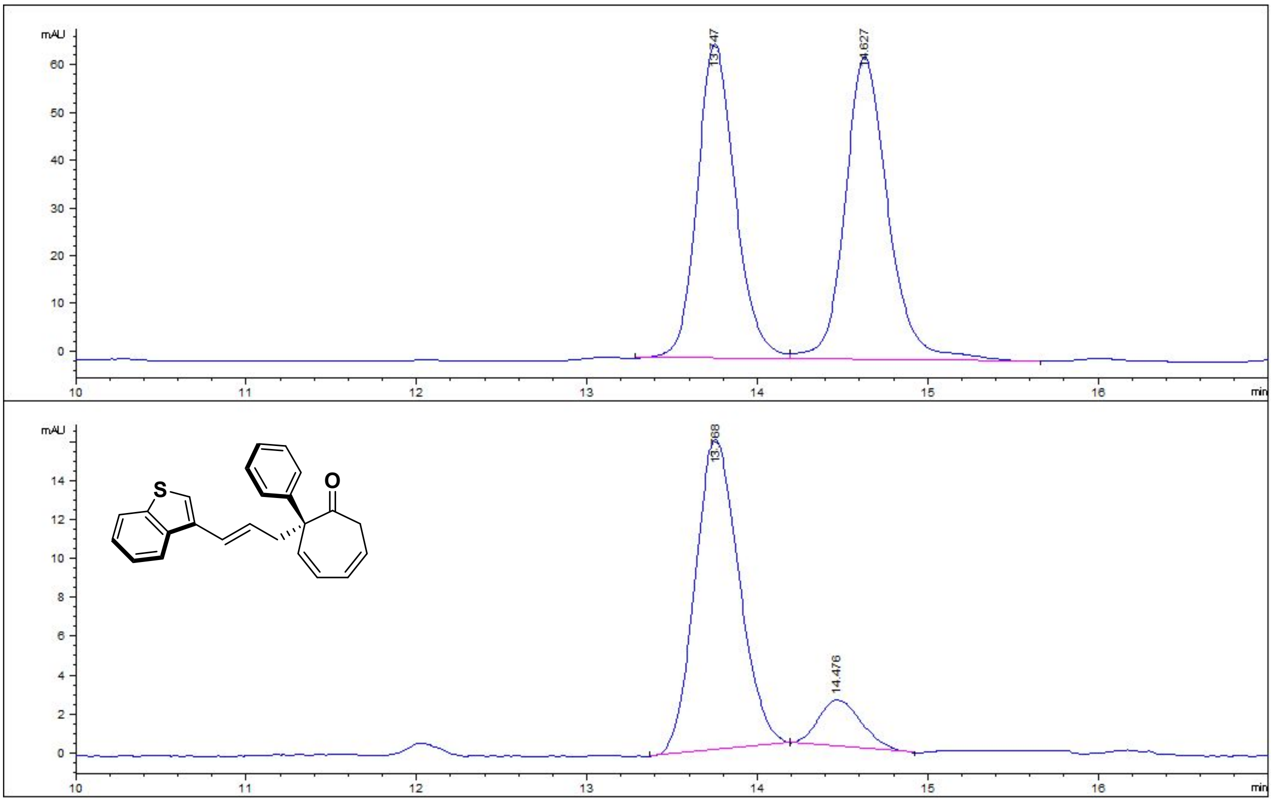

Peak	RetTime [min]	Width [min]	Area%
1	13.747	0.2394	47.8407
2	14.627	0.2674	52.1593

Peak	RetTime [min]	Width [min]	Area%
1	13.768	0.2829	87.0424
2	14.476	0.2343	12.9576

Compound **3an**

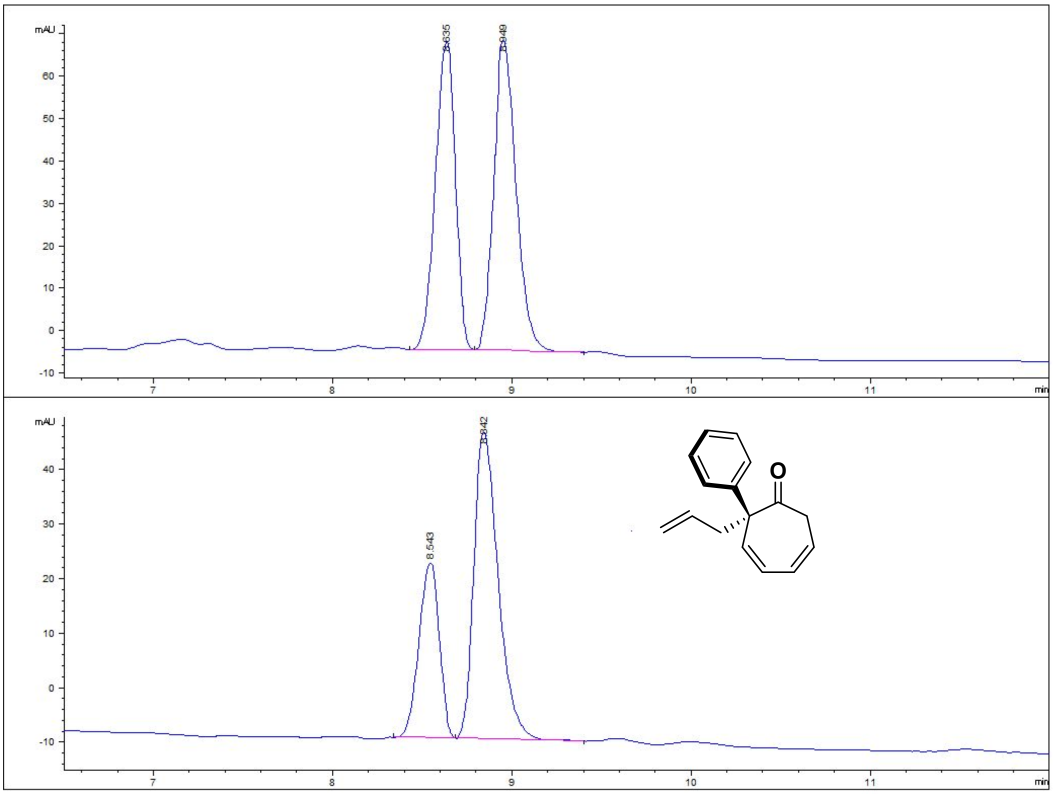

Peak	RetTime [min]	Width [min]	Area%
1	8.635	0.1553	48.6606
2	8.949	0.1642	51.3394

Peak	RetTime [min]	Width [min]	Area%
1	8.543	0.1567	34.0522
2	8.842	0.1731	65.9478

Compound **3ba**

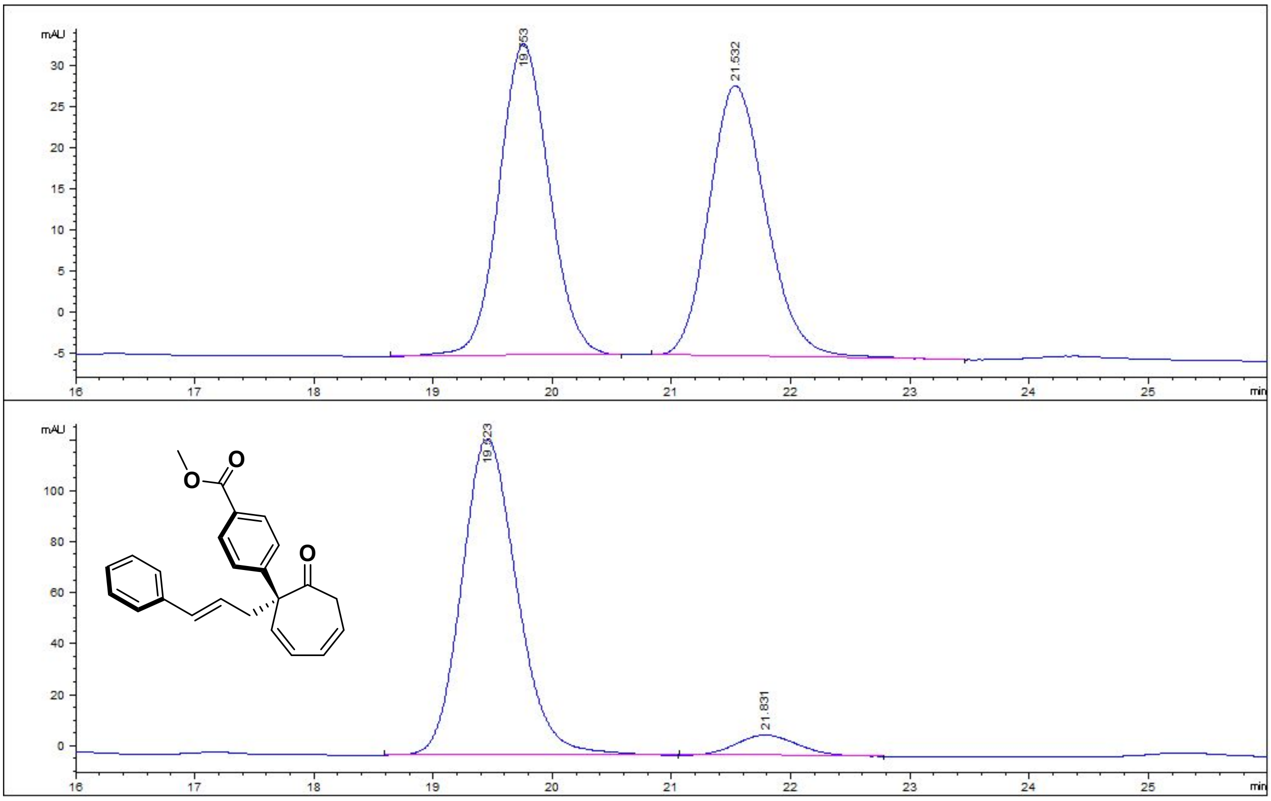

Peak	RetTime [min]	Width [min]	Area%
1	19.753	0.4547	50.1940
2	21.532	0.5115	49.8060

Peak	RetTime [min]	Width [min]	Area%
1	19.523	0.5028	94.1209
2	21.831	0.4427	5.8791

Compound **3ca**

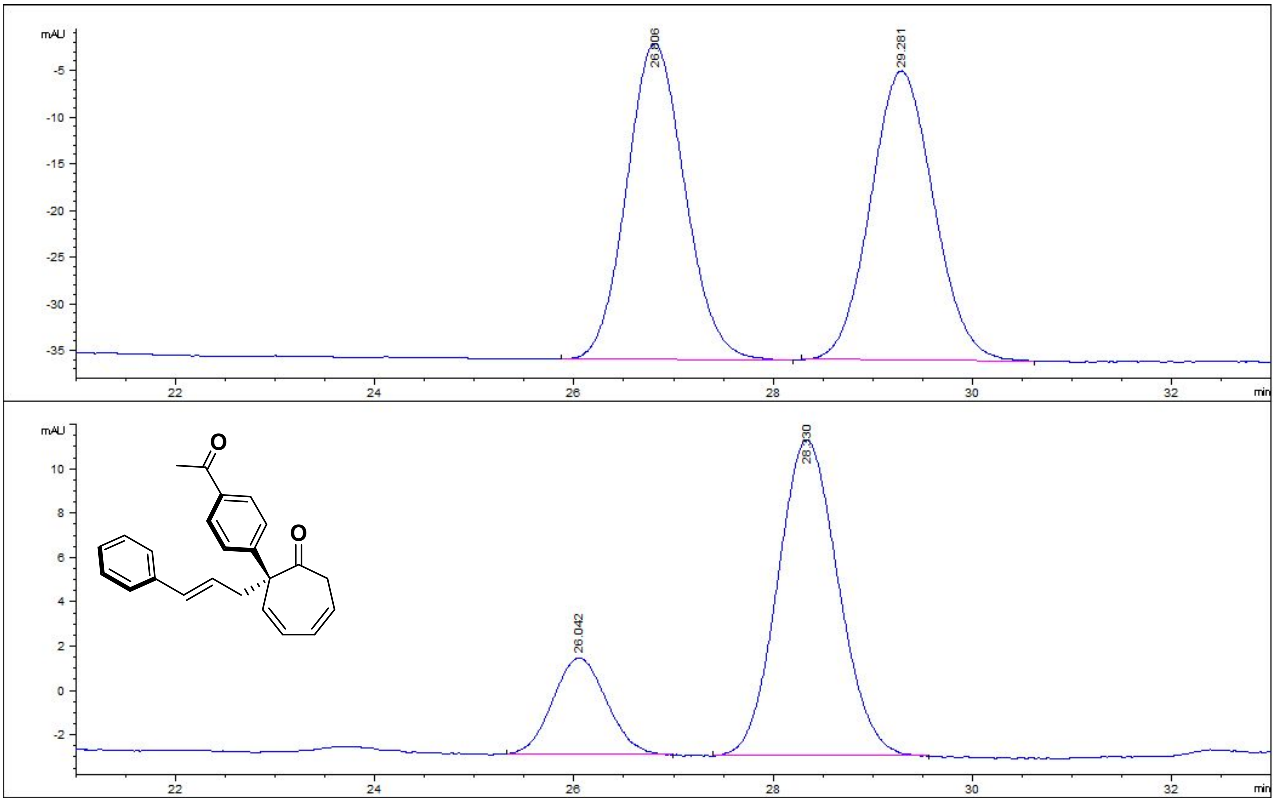

Peak	RetTime [min]	Width [min]	Area%
1	26.806	0.6160	50.1855
2	29.281	0.6794	49.8145

Peak	RetTime [min]	Width [min]	Area%
1	26.042	0.4521	21.2725
2	28.330	0.6128	78.7275

Compound **3da**

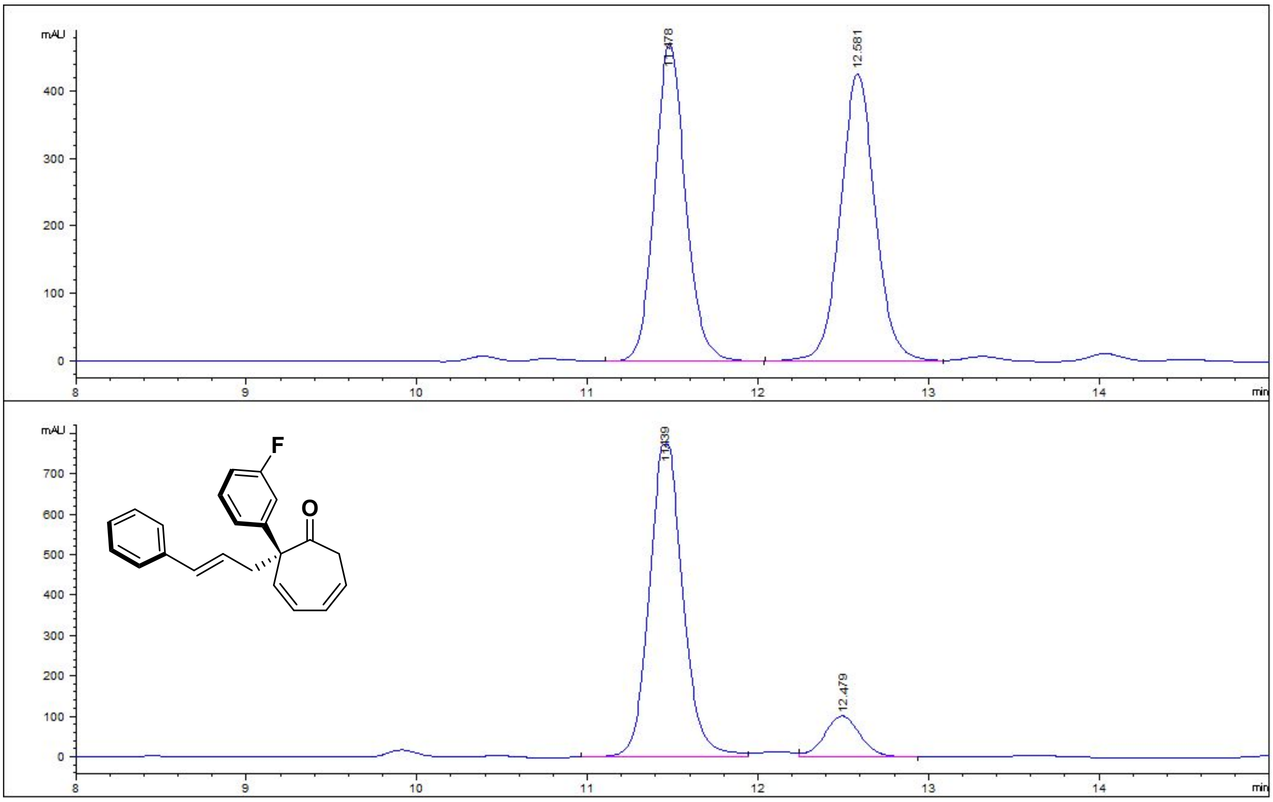

Peak	RetTime [min]	Width [min]	Area%
1	11.478	0.1921	49.3390
2	12.581	0.2173	50.6610

Peak	RetTime [min]	Width [min]	Area%
1	11.439	0.1921	87.8485
2	12.479	0.2173	12.1515

Compound **3ea**

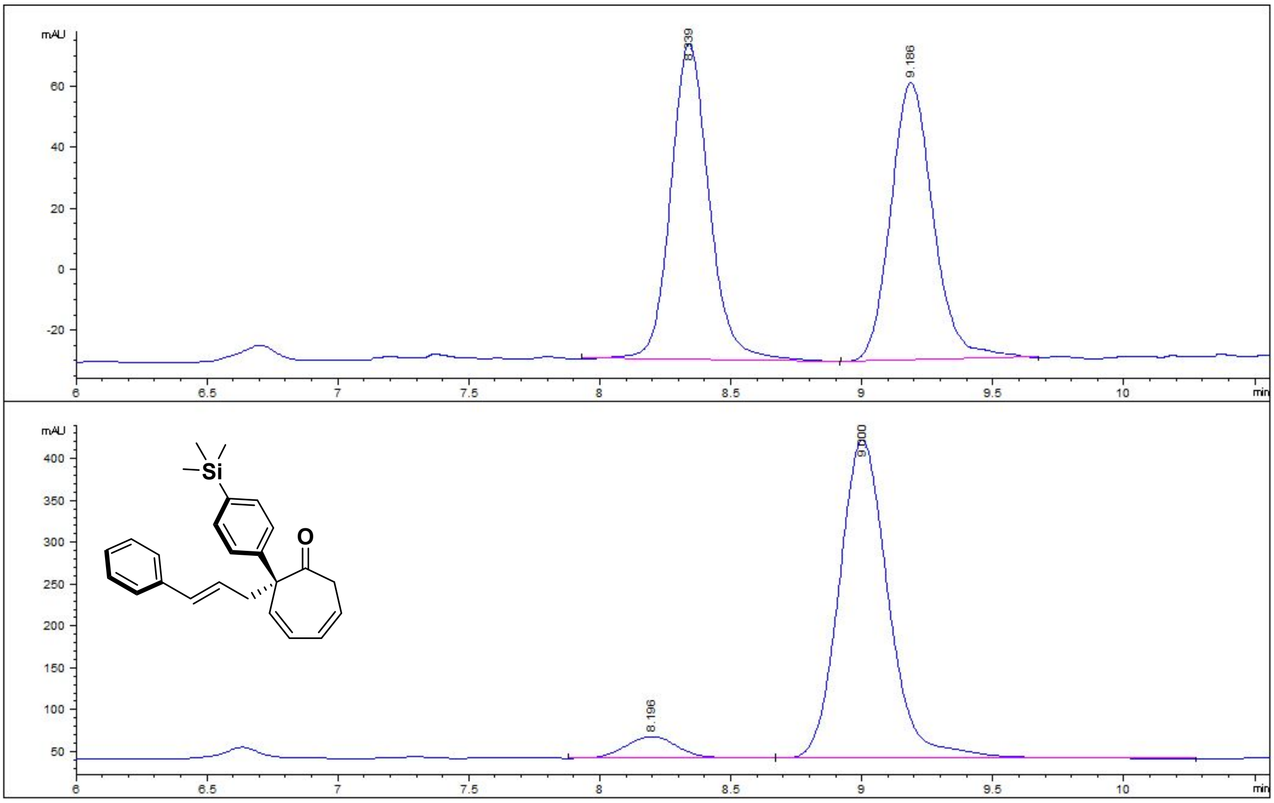

Peak	RetTime [min]	Width [min]	Area%
1	8.339	0.1554	50.6938
2	9.186	0.1723	49.3062

Peak	RetTime [min]	Width [min]	Area%
1	8.196	0.2205	6.8130
2	9.000	0.2034	93.1870

Compound **3fa**

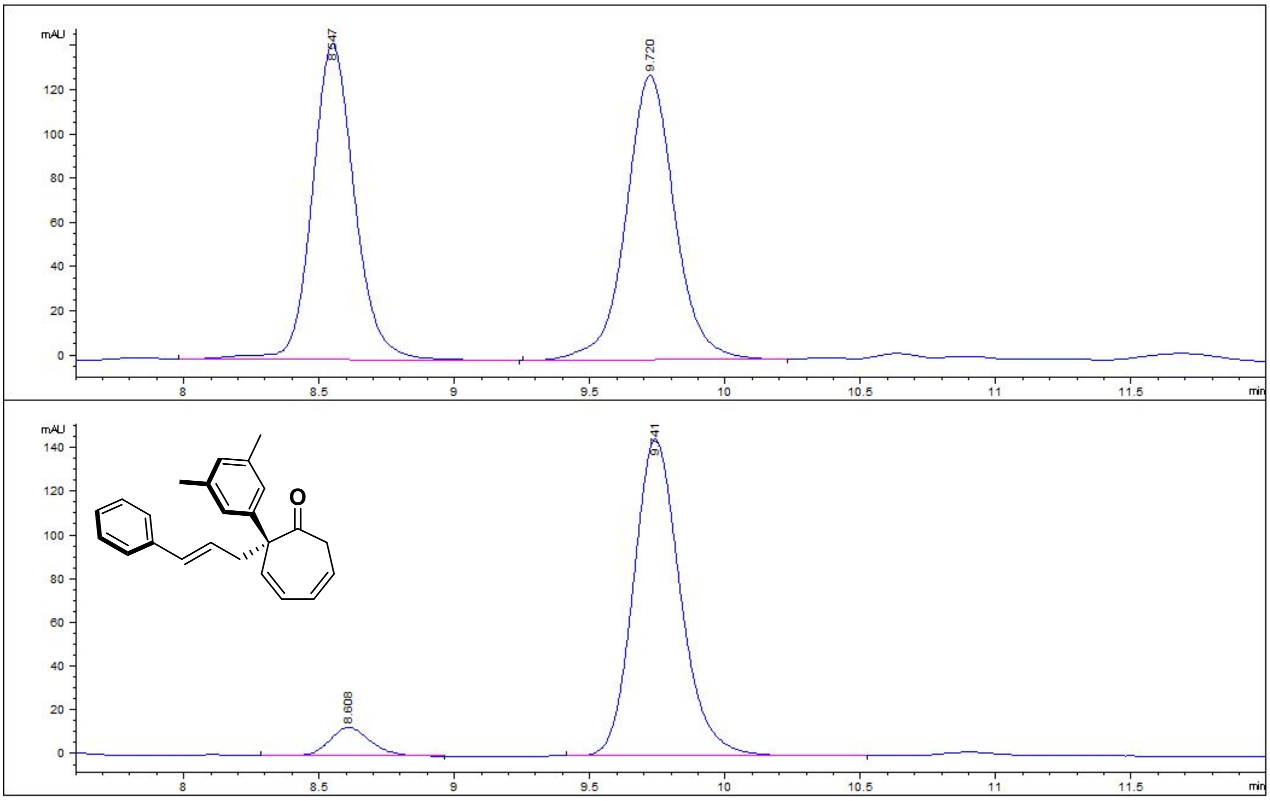

Peak	RetTime [min]	Width [min]	Area%
1	8.547	0.1647	49.0517
2	9.720	0.1923	50.9483

Peak	RetTime [min]	Width [min]	Area%
1	8.608	0.1598	7.2074
2	9.741	0.1861	92.7926

Compound **3ga**

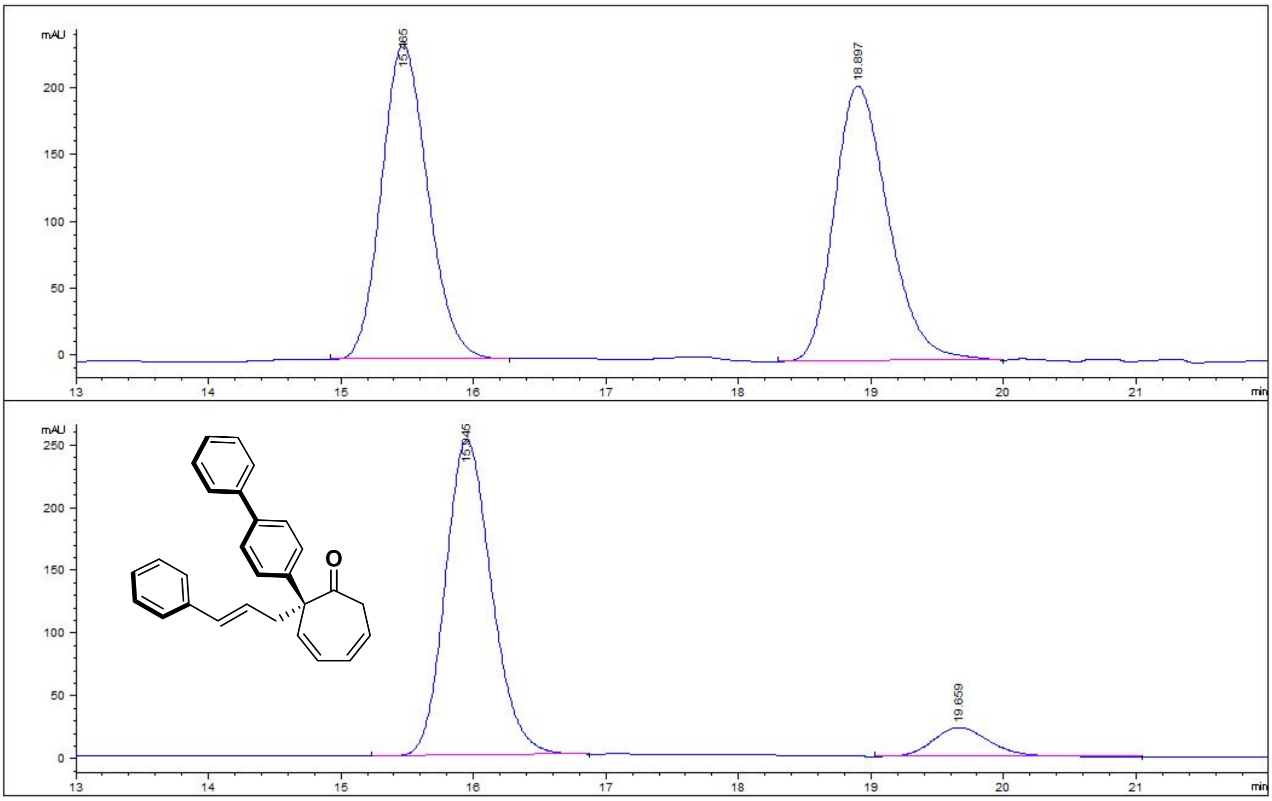

Peak	RetTime [min]	Width [min]	Area%
1	15.465	0.3769	49.4489
2	18.897	0.4391	50.5511

Peak	RetTime [min]	Width [min]	Area%
1	15.945	0.3762	89.8365
2	19.659	0.4474	10.1635

Compound **3ha**

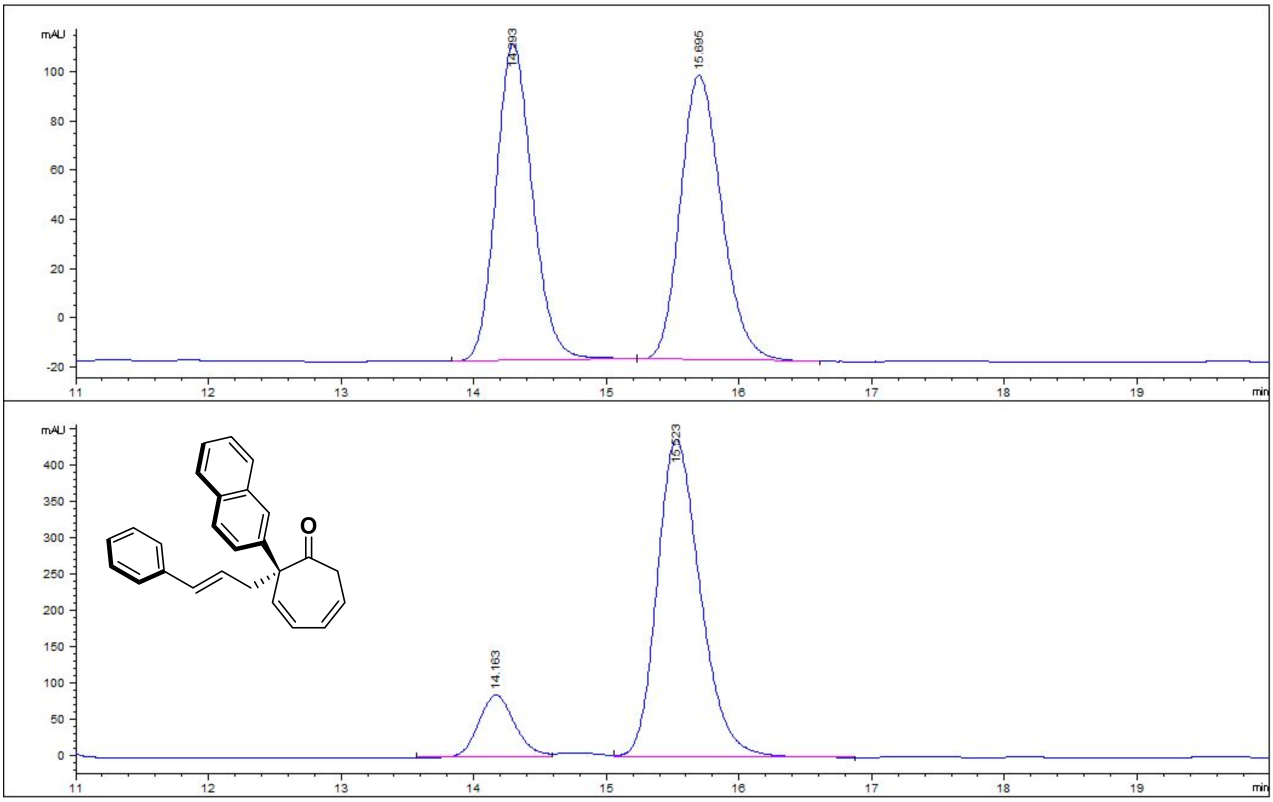

Peak	RetTime [min]	Width [min]	Area%
1	14.293	0.2933	49.6669
2	15.695	0.3375	50.3331

Peak	RetTime [min]	Width [min]	Area%
1	14.163	0.2958	14.2763
2	15.523	0.3530	85.7237

Compound **3ef**

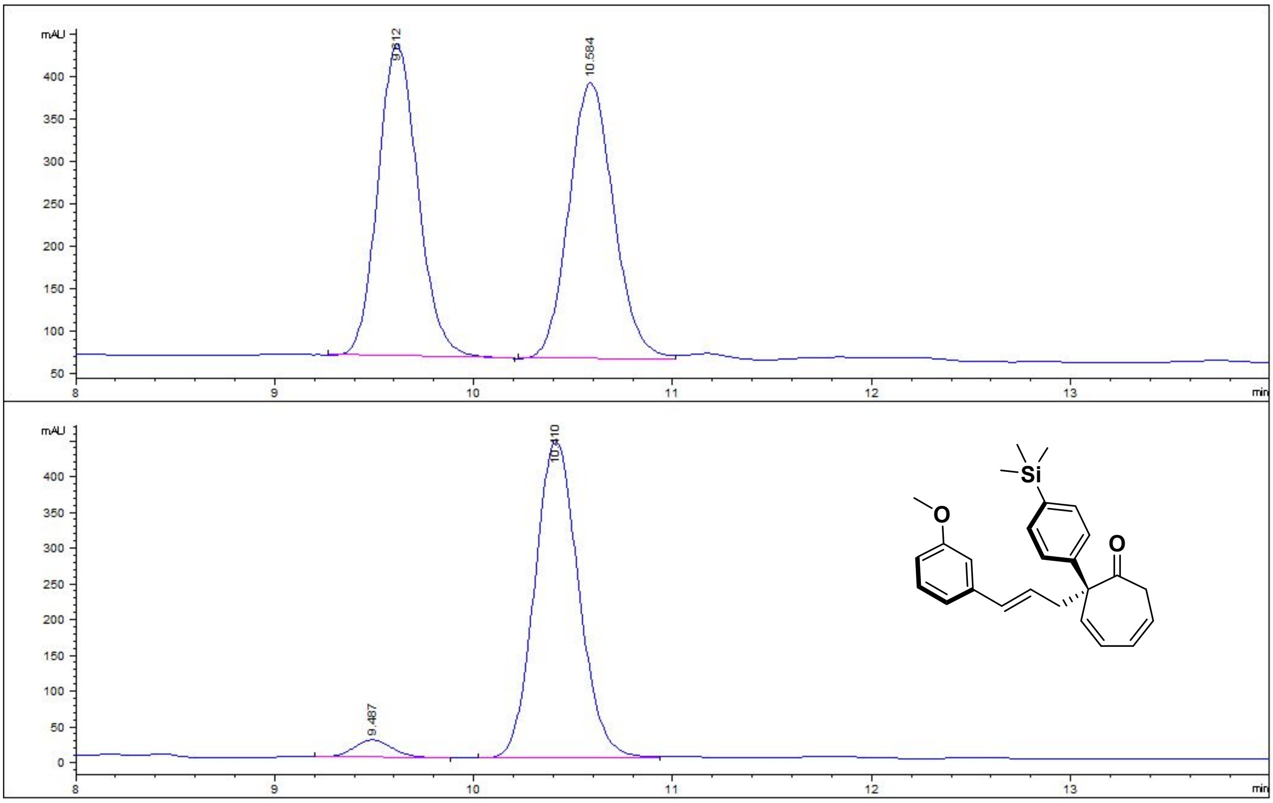

Peak	RetTime [min]	Width [min]	Area%
1	9.612	0.2196	50.2782
2	10.584	0.2430	49.7218

Peak	RetTime [min]	Width [min]	Area%
1	9.487	0.2052	4.4618
2	10.410	0.2367	95.5382

Compound **3if**

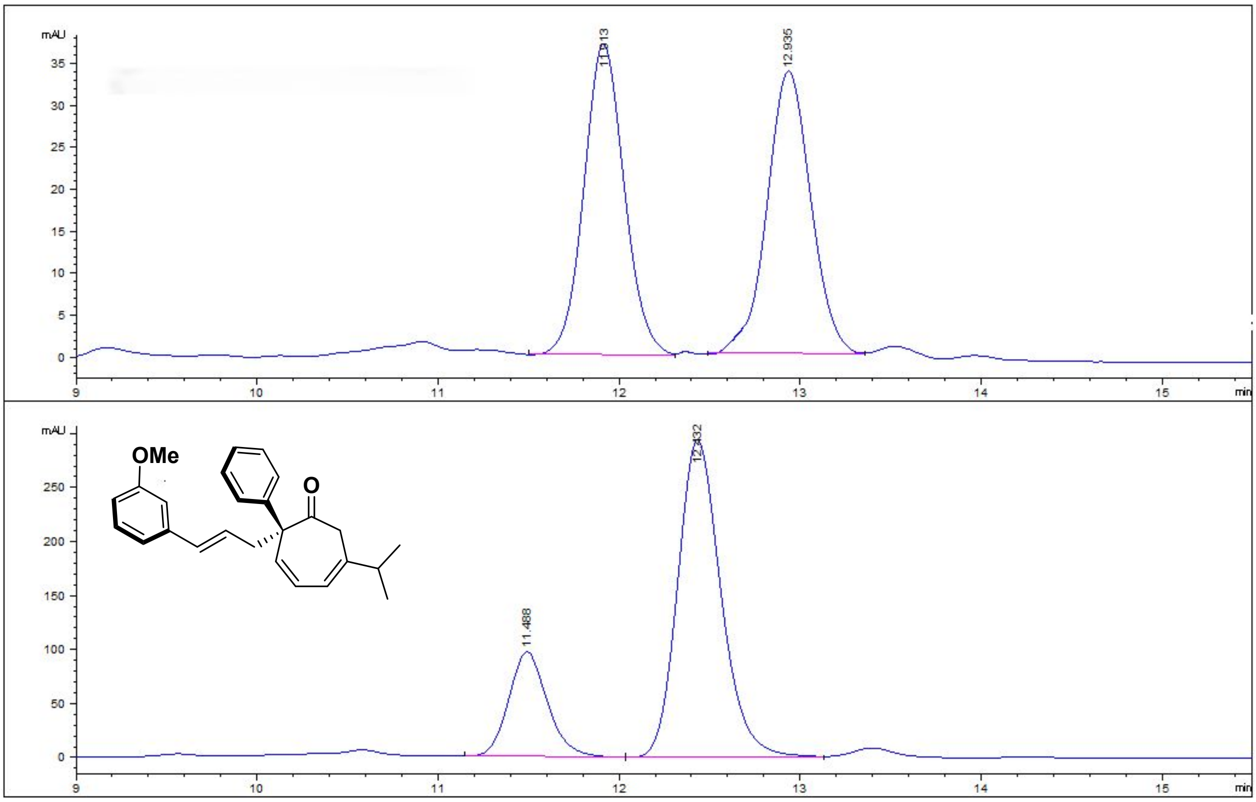

Peak	RetTime [min]	Width [min]	Area%
1	11.913	0.2393	50.0596
2	12.935	0.2587	49.9404

Peak	RetTime [min]	Width [min]	Area%
1	11.488	0.2340	23.2833
2	12.432	0.2537	76.7167

Compound **3jf**

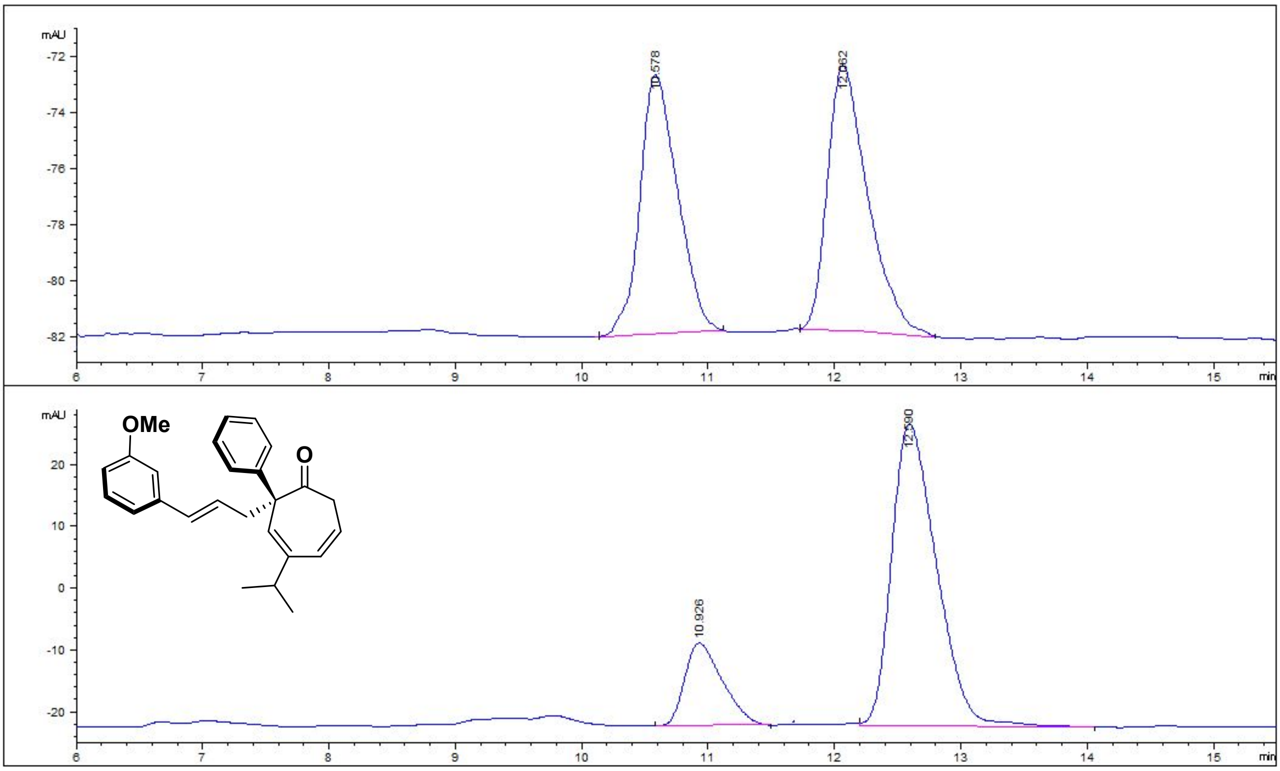

Peak	RetTime [min]	Width [min]	Area%
1	10.578	0.3020	48.8694
2	12.062	0.3196	51.1306

Peak	RetTime [min]	Width [min]	Area%
1	10.926	0.3056	18.6334
2	12.590	0.3764	81.3666

Compound **3ka**

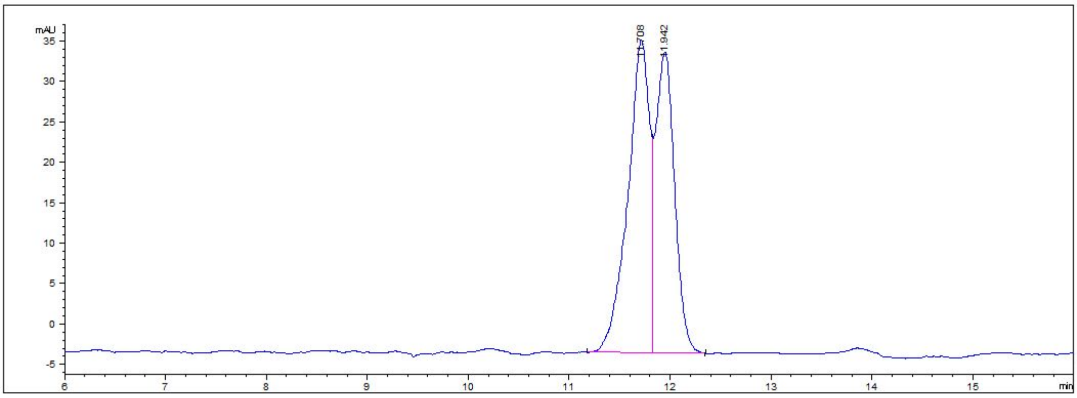

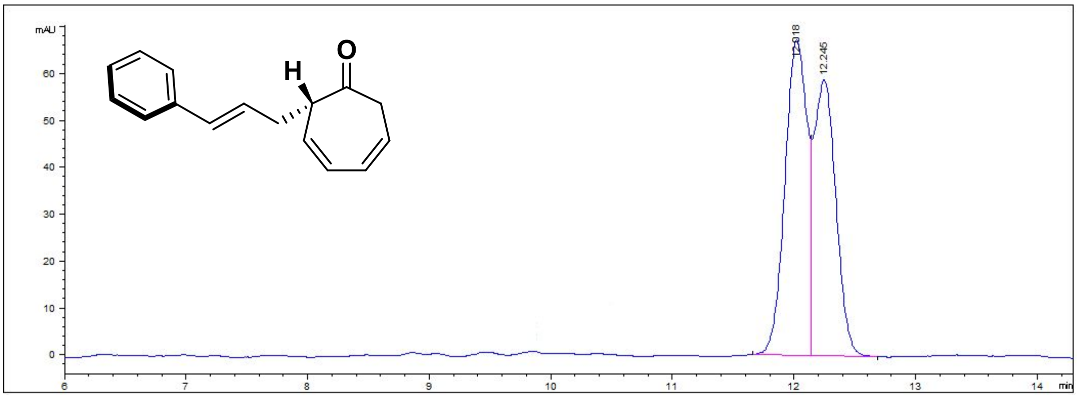

Peak	RetTime [min]	Width [min]	Area%
1	12.018	0.1964	54.1873
2	12.245	0.1868	45.8127

Peak	RetTime [min]	Width [min]	Area%
1	11.708	0.2285	56.2431
2	11.942	0.1906	43.7569

Compound **4**

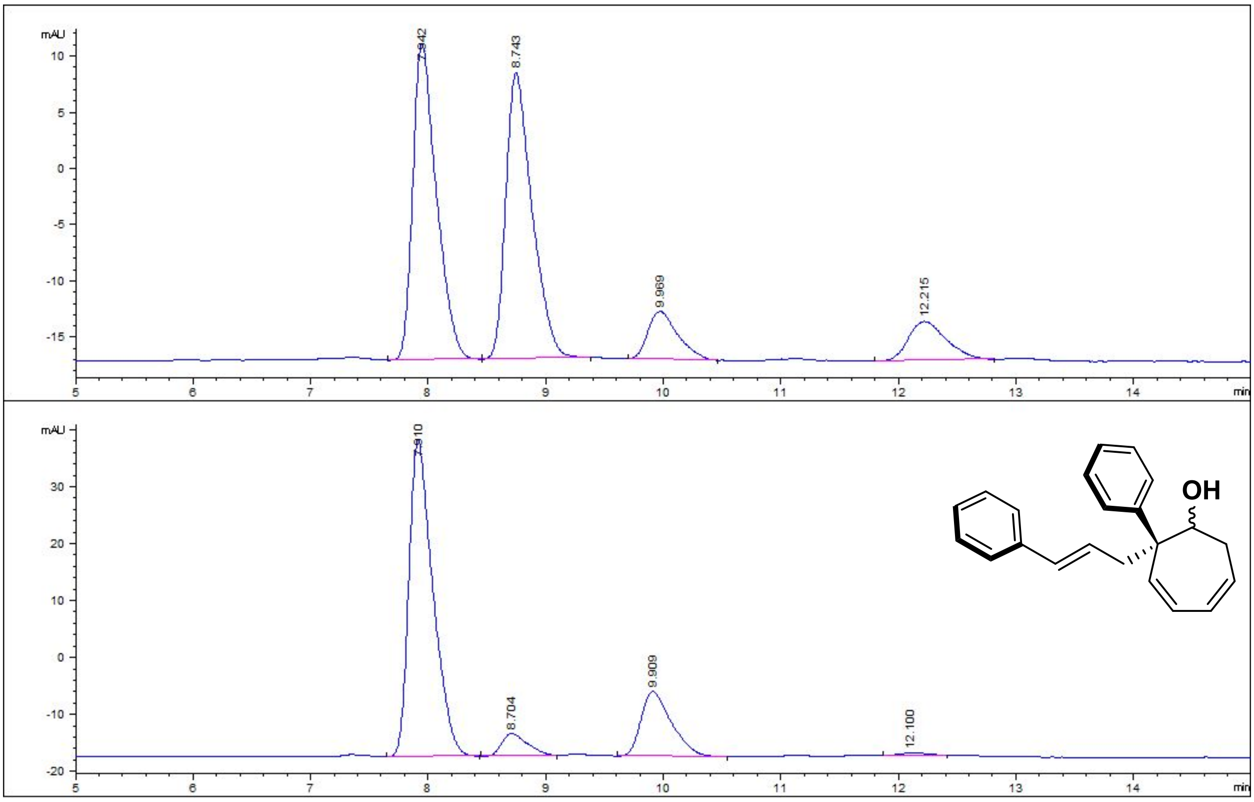

Peak	RetTime [min]	Width [min]	Area%
1	7.942	0.1871	49.9332
2	8.743	0.2643	50.0668

Peak	RetTime [min]	Width [min]	Area%
1	7.910	0.1913	92.8949
2	8.704	0.2545	7.1051

Peak	RetTime [min]	Width [min]	Area%
3	9.969	0.1772	49.1603
4	12.215	0.2468	50.8397

Peak	RetTime [min]	Width [min]	Area%
3	9.909	0.2032	96.7180
4	12.100	0.2846	3.2820

Compound **5**

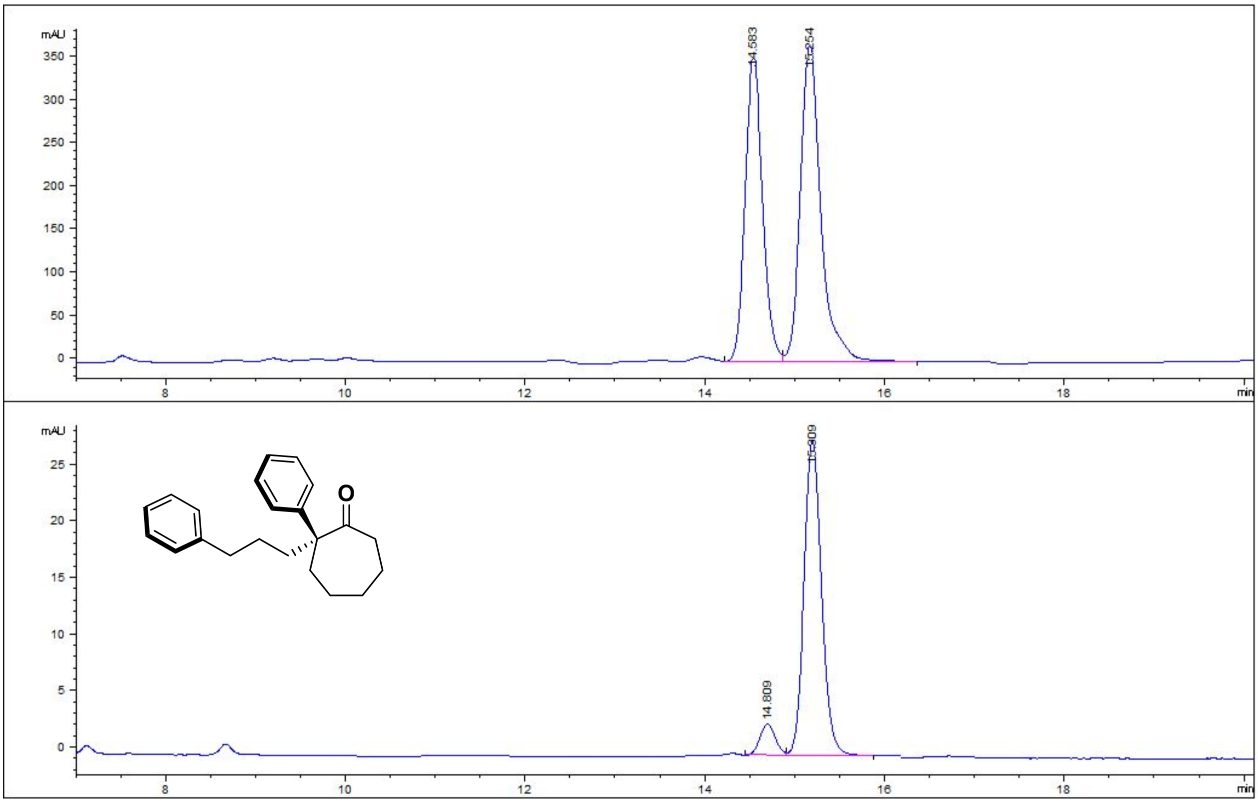

Peak	RetTime [min]	Width [min]	Area%
1	14.583	0.2130	48.3171
2	15.254	0.2406	51.6829

Peak	RetTime [min]	Width [min]	Area%
1	14.809	0.1899	8.0869
2	15.309	0.2106	91.9131

12. Cartesian coordinates

3an

Energy (FREE) = -435032.0783828448 kcal.mol^{-1}

Atom X Y Z

1 C 1.1675 1.2442 1.9609
2 C 0.1816 2.2845 1.7167
3 C -0.2108 2.7413 0.5062
4 C 1.4460 0.1918 1.1548
5 C 0.3658 2.3941 -0.8301
6 C 0.7966 -0.1208 -0.1768
7 C 1.1529 1.1097 -1.0483
8 H 1.0182 3.2186 -1.1816
9 H 1.7020 1.2953 2.9208
10 H -0.2664 2.7499 2.6067
11 H -0.9908 3.5153 0.4778
12 H 2.2420 -0.4991 1.4645
13 O 2.0274 1.0602 -1.8909
14 C -0.7209 -0.3298 -0.1357
15 C -1.4368 -0.3125 -1.3456
16 C -1.4129 -0.6398 1.0447
17 C -2.8099 -0.5787 -1.3750
18 H -0.9136 -0.0908 -2.2858
19 C -2.7852 -0.9131 1.0188
20 H -0.8713 -0.6654 1.9967
21 C -3.4908 -0.8798 -0.1896
22 H -3.3481 -0.5536 -2.3301
23 H -3.3070 -1.1529 1.9532
24 H -4.5668 -1.0902 -0.2081
25 C 1.4766 -1.3772 -0.7942
26 H 1.0988 -1.4885 -1.8293
27 H 2.5613 -1.1820 -0.8663
28 C 1.2221 -2.6336 -0.0190
29 H 0.1754 -2.9718 0.0348
30 C 2.1709 -3.3529 0.5986
31 H 3.2280 -3.0485 0.5675
32 H 1.9264 -4.2714 1.1496
33 H -0.4585 2.3670 -1.5732

D(S)

Energy (FREE) = -2.3632684284320357E6 kcal.mol^{-1}

Atom X Y Z

1 C 3.4660 -1.8223 5.4639
2 C 2.7623 -0.8617 4.7123
3 C 2.9322 0.5090 5.0521
4 C 3.8084 0.8569 6.0956
5 C 4.4920 -0.1120 6.8319
6 C 4.3137 -1.4608 6.5158
7 P 1.8051 -1.4099 3.2094
8 C 0.0954 -1.6293 3.9009
9 C -0.2198 -1.5050 5.2660

10 C -1.5317 -1.7121 5.7145
11 C -2.5362 -2.0659 4.8091
12 C -2.2308 -2.1929 3.4467
13 C -0.9299 -1.9599 2.9935
14 C 2.2684 1.7007 4.4015
15 O 2.8703 2.7729 4.2950
16 Pd 2.5627 -0.1005 1.5422
17 P 4.6066 0.6427 0.8724
18 C 5.8313 0.7919 2.2383
19 C 6.2055 2.0258 2.7979
20 C 7.0553 2.0714 3.9112
21 C 7.5467 0.8898 4.4752
22 C 7.1728 -0.3457 3.9273
23 C 6.3140 -0.3951 2.8271
24 C 0.4196 -1.1483 -1.4899
25 C 0.7335 -2.2611 -0.8087
26 C -0.6169 -1.0905 -2.5766
27 C -1.7119 0.0225 -2.3746
28 C -2.2144 -0.1777 -0.9139
29 O -1.9387 0.6462 -0.0430
30 C -1.1741 1.4468 -2.5488
31 C 0.1212 1.7433 -2.9944
32 C 0.5289 3.0706 -3.1919
33 C -0.3548 4.1254 -2.9525
34 C -1.6578 3.8425 -2.5207
35 C -2.0579 2.5194 -2.3237
36 C -2.7835 -0.1865 -3.4230
37 C -3.6726 -1.2260 -3.5563
38 C -3.9485 -2.2752 -2.6430
39 C -3.5882 -2.3370 -1.2812
40 C -2.8965 -1.4081 -0.5210
41 C 2.2939 -3.1841 3.0059
42 C 1.6393 -4.2473 3.6541
43 C 2.0559 -5.5679 3.4475
44 C 3.1318 -5.8439 2.5954
45 C 3.7893 -4.7920 1.9451
46 C 3.3689 -3.4731 2.1447
47 C 4.6004 2.3433 0.1422
48 C 3.4241 3.1218 0.2098
49 C 3.4401 4.4494 -0.2563
50 C 4.5988 4.9937 -0.8161
51 C 5.7601 4.2177 -0.9053
52 C 5.7574 2.9059 -0.4224
53 C 2.0896 2.5787 0.6585
54 O 1.5065 1.6837 0.0269
55 C 5.4990 -0.3310 -0.4138
56 C 4.7098 -0.9610 -1.3946
57 C 5.3072 -1.6973 -2.4225
58 C 6.7011 -1.8270 -2.4732
59 C 7.4929 -1.2130 -1.4953
60 C 6.8975 -0.4660 -0.4716

61 N 1.5285 3.2366 1.6981
62 C 0.1641 3.0035 2.1457
63 C 0.1424 2.7268 3.6764
64 C -1.2708 2.5360 4.1908
65 C -2.1035 1.5429 3.6474
66 C -3.4045 1.3686 4.1302
67 C -3.8905 2.1851 5.1588
68 C -3.0668 3.1767 5.7043
69 C -1.7638 3.3486 5.2229
70 C -0.7389 4.1782 1.8015
71 C -1.9539 3.9652 1.1338
72 C -2.8058 5.0393 0.8493
73 C -2.4510 6.3390 1.2284
74 C -1.2368 6.5603 1.8915
75 C -0.3868 5.4864 2.1764
76 N 0.9593 1.5762 4.0526
77 H 0.5929 3.6052 4.1729
78 H -0.1872 2.1077 1.6078
79 H 0.5627 5.6754 2.6939
80 H -0.9495 7.5760 2.1890
81 H -3.1176 7.1807 1.0051
82 H -3.7507 4.8583 0.3225
83 H -2.2265 2.9478 0.8287
84 H -1.7456 0.9034 2.8302
85 H -4.0426 0.5899 3.6960
86 H -4.9121 2.0498 5.5335
87 H -3.4396 3.8216 6.5091
88 H -1.1203 4.1278 5.6499
89 H 2.1217 3.8455 2.2605
90 H 0.4672 0.7032 4.2430
91 H 6.6781 2.3116 -0.4722
92 H 6.6734 4.6372 -1.3434
93 H 4.5924 6.0252 -1.1873
94 H 2.5238 5.0492 -0.1977
95 H 3.3605 -2.8833 5.2139
96 H 4.8416 -2.2399 7.0785
97 H 5.1637 0.1885 7.6444
98 H 3.9428 1.9202 6.3201
99 H 1.5080 -2.2350 -0.0254
100 H 0.9370 -0.2118 -1.2322
101 H 0.2331 -3.2221 -1.0049
102 H -0.1452 -0.9020 -3.5610
103 H -1.1268 -2.0668 -2.6537
104 H 0.5638 -1.2532 5.9914
105 H -1.7635 -1.6052 6.7811
106 H -3.5607 -2.2352 5.1610
107 H -3.0177 -2.4677 2.7338
108 H -0.6986 -2.0411 1.9218
109 H 0.7986 -4.0476 4.3286
110 H 1.5346 -6.3866 3.9582
111 H 3.4541 -6.8795 2.4336

112 H 4.6281 -4.9990 1.2697
113 H 3.8648 -2.6434 1.6211
114 H 5.8294 2.9620 2.3704
115 H 7.3348 3.0427 4.3370
116 H 8.2142 0.9283 5.3443
117 H 7.5455 -1.2789 4.3667
118 H 6.0133 -1.3690 2.4190
119 H 7.5284 0.0089 0.2890
120 H 8.5845 -1.3143 -1.5281
121 H 7.1707 -2.4128 -3.2725
122 H 4.6798 -2.1817 -3.1802
123 H 3.6156 -0.8764 -1.3413
124 H -2.8702 -1.5622 0.5649
125 H -3.9769 -3.2029 -0.7282
126 H -4.5870 -3.0877 -3.0113
127 H -4.2450 -1.2484 -4.4939
128 H -2.7703 0.5496 -4.2371
129 H -3.0812 2.3118 -1.9864
130 H -2.3653 4.6585 -2.3314
131 H -0.0347 5.1632 -3.1047
132 H 1.5509 3.2734 -3.5344
133 H 0.8443 0.9472 -3.1926

Aprime
Energy (FREE) = -361651.6246580094 kcal.mol^{-1}
Atom X Y Z
1 C -0.6169 -0.0216 0.7503
2 C 0.5785 -0.1930 1.5271
3 C 1.7385 0.4876 1.2956
4 C -0.9903 1.1434 0.0873
5 C 1.9122 1.2976 0.0481
6 C -0.3473 2.4201 -0.0046
7 C 1.0810 2.5910 0.1473
8 H 2.9635 1.5991 -0.1001
9 H -1.3783 -0.8133 0.8121
10 H 0.5339 -0.8918 2.3784
11 H 2.5651 0.4381 2.0195
12 H -2.0175 1.1099 -0.3038
13 O 1.6907 3.6760 0.2767
14 C -1.1731 3.5879 -0.3879
15 C -2.3102 3.4615 -1.2299
16 C -0.8749 4.9019 0.0613
17 C -3.0961 4.5598 -1.5877
18 H -2.5753 2.4801 -1.6400
19 C -1.6631 5.9997 -0.2980
20 H -0.0005 5.0423 0.6995
21 C -2.7837 5.8457 -1.1246
22 H -3.9596 4.4084 -2.2490
23 H -1.3978 6.9952 0.0830
24 H -3.3998 6.7088 -1.4053
25 H 1.5732 0.7032 -0.8281

C(S)
Energy (FREE) = -2.363270697539571E6 kcal.mol^{-1}
Atom X Y Z
1 C 3.0641 -1.5398 3.6180
2 C 2.0644 -0.7797 2.9792
3 C 1.9066 0.5768 3.3609
4 C 2.7454 1.1225 4.3475
5 C 3.7123 0.3481 4.9864
6 C 3.8734 -0.9905 4.6156
7 P 1.0128 -1.5693 1.6723
8 C -0.4543 -2.0763 2.6746
9 C -0.2658 -2.6721 3.9376
10 C -1.3643 -3.0977 4.6888
11 C -2.6638 -2.9296 4.1925
12 C -2.8592 -2.3252 2.9473
13 C -1.7605 -1.8957 2.1927
14 C 0.9409 1.5566 2.7559
15 O 1.3136 2.6862 2.4288
16 Pd 0.8401 -0.7838 -0.5374
17 P 2.6060 0.7029 -0.9797
18 C 3.5616 1.6916 0.2431
19 C 3.5231 3.0951 0.2737
20 C 4.3219 3.8038 1.1781
21 C 5.1674 3.1207 2.0580
22 C 5.2045 1.7200 2.0384
23 C 4.4024 1.0098 1.1431
24 C -0.0366 -2.0865 -2.0787
25 C 0.3594 -0.9105 -2.7461
26 C -0.9927 -2.0264 -1.0482
27 C -2.5572 -4.6488 -3.5199
28 C -3.2795 -3.7451 -2.6278
29 O -3.8087 -4.2600 -1.6155
30 C -1.8092 -4.4432 -4.6772
31 C -1.5149 -3.2542 -5.3594
32 C -1.9582 -1.9653 -4.9996
33 C -2.8074 -1.5867 -3.9549
34 C -3.4336 -2.2988 -2.9017
35 C 1.8395 -3.1746 1.3150
36 C 1.1660 -4.4019 1.4426
37 C 1.7814 -5.5911 1.0304
38 C 3.0723 -5.5706 0.4929
39 C 3.7478 -4.3499 0.3578
40 C 3.1330 -3.1604 0.7546
41 C 2.0611 2.0060 -2.1796
42 C 0.7453 2.5261 -2.1814
43 C 0.3973 3.5207 -3.1158
44 C 1.3286 4.0193 -4.0258
45 C 2.6272 3.5010 -4.0336
46 C 2.9806 2.5036 -3.1228
47 C -0.4305 2.0610 -1.3516

48 O -1.4201 1.5978 -1.9253
49 C 3.9895 -0.2010 -1.8117
50 C 3.7775 -1.5043 -2.2938
51 C 4.8119 -2.2145 -2.9141
52 C 6.0761 -1.6332 -3.0550
53 C 6.3051 -0.3428 -2.5609
54 C 5.2738 0.3659 -1.9375
55 N -0.4215 2.2741 -0.0137
56 C -1.6711 2.0816 0.7284
57 C -1.3846 2.1346 2.2439
58 C -2.6158 1.9603 3.1151
59 C -3.6660 1.0915 2.7790
60 C -4.7605 0.9342 3.6371
61 C -4.8182 1.6394 4.8443
62 C -3.7737 2.5058 5.1901
63 C -2.6826 2.6637 4.3300
64 C -2.7069 3.1238 0.3306
65 C -3.9319 2.7343 -0.2283
66 C -4.8881 3.6913 -0.5841
67 C -4.6251 5.0530 -0.3904
68 C -3.3977 5.4509 0.1547
69 C -2.4435 4.4913 0.5114
70 N -0.3565 1.1705 2.6400
71 H -0.9477 3.1248 2.4608
72 H -2.0525 1.0810 0.4586
73 H -1.4795 4.8154 0.9247
74 H -3.1805 6.5157 0.3024
75 H -5.3728 5.8048 -0.6705
76 H -5.8406 3.3687 -1.0222
77 H -4.1373 1.6703 -0.3971
78 H -3.6461 0.5303 1.8383
79 H -5.5753 0.2556 3.3567
80 H -5.6778 1.5170 5.5138
81 H -3.8112 3.0664 6.1317
82 H -1.8670 3.3463 4.6006
83 H 0.3609 2.7444 0.4497
84 H -0.6612 0.2842 3.0428
85 H 3.9972 2.0999 -3.1560
86 H 3.3688 3.8659 -4.7534
87 H 1.0354 4.8006 -4.7365
88 H -0.6315 3.8985 -3.1213
89 H 3.2120 -2.5872 3.3357
90 H 4.6324 -1.6152 5.1008
91 H 4.3460 0.7914 5.7631
92 H 2.6256 2.1806 4.6043
93 H 1.1706 -0.9455 -3.4846
94 H 0.5234 -3.0214 -2.2337
95 H -0.3399 -0.0674 -2.8143
96 H -1.2464 -2.9304 -0.4820
97 H -1.7152 -1.1992 -1.0243
98 H 0.7460 -2.8145 4.3356

99 H -1.2038 -3.5638 5.6680
100 H -3.5249 -3.2644 4.7826
101 H -3.8726 -2.1788 2.5569
102 H -1.9250 -1.4029 1.2288
103 H 0.1539 -4.4377 1.8599
104 H 1.2426 -6.5402 1.1344
105 H 3.5518 -6.5037 0.1746
106 H 4.7575 -4.3206 -0.0686
107 H 3.6645 -2.2101 0.6242
108 H 2.8717 3.6458 -0.4139
109 H 4.2839 4.8996 1.1888
110 H 5.7989 3.6781 2.7601
111 H 5.8617 1.1753 2.7261
112 H 4.4487 -0.0859 1.1320
113 H 5.4783 1.3651 -1.5368
114 H 7.2966 0.1156 -2.6545
115 H 6.8880 -2.1893 -3.5384
116 H 4.6268 -3.2320 -3.2781
117 H 2.8010 -1.9808 -2.1583
118 H -2.9872 -0.5029 -3.9197
119 H -1.6110 -1.1397 -5.6365
120 H -0.8770 -3.3342 -6.2492
121 H -1.3797 -5.3570 -5.1154
122 H -2.6348 -5.6856 -3.1645
123 C -4.3855 -1.5357 -2.0645
124 C -4.5383 -1.7385 -0.6675
125 C -5.2103 -0.5424 -2.6531
126 C -5.4629 -1.0008 0.0778
127 H -3.9208 -2.4887 -0.1708
128 C -6.1349 0.1920 -1.9061
129 H -5.1512 -0.3721 -3.7346
130 C -6.2748 -0.0332 -0.5305
131 H -5.5549 -1.1871 1.1561
132 H -6.7634 0.9381 -2.4089
133 H -7.0004 0.5393 0.0599

TS to-D(S)
Energy (FREE) = -2.363270058094331E6 kcal.mol^{-1}
Atom X Y Z
1 C -6.4423 -0.0442 -0.1779
2 C -6.2636 -1.4238 -0.3312
3 C -5.1476 -1.9278 -1.0065
4 C -4.1728 -1.0663 -1.5566
5 C -4.3698 0.3222 -1.3900
6 C -5.4878 0.8255 -0.7167
7 C -3.0333 -1.5925 -2.3793
8 C -2.8260 -0.9133 -3.6167
9 C -2.0563 -1.2110 -4.7426
10 C -1.3605 -2.3864 -5.0595
11 C -1.2973 -3.5536 -4.2753
12 C -1.8264 -3.8040 -3.0199

13 C -2.5669 -2.9797 -2.0738
14 O -2.7864 -3.4656 -0.9465
15 C -1.3300 -0.7531 -1.0608
16 C -0.0901 -1.2142 -1.5839
17 C 0.7438 -0.3827 -2.3844
18 Pd 1.4783 -0.2726 -0.3559
19 P 3.3263 1.0995 -0.7054
20 C 4.6858 0.2054 -1.5996
21 C 4.3654 -0.9243 -2.3726
22 C 5.3604 -1.6343 -3.0543
23 C 6.6965 -1.2283 -2.9674
24 C 7.0308 -0.1119 -2.1913
25 C 6.0361 0.5976 -1.5102
26 P 1.5946 -1.3284 1.7605
27 C 2.5225 -2.9014 1.4803
28 C 2.1791 -4.1169 2.0988
29 C 2.9170 -5.2774 1.8363
30 C 4.0080 -5.2387 0.9601
31 C 4.3597 -4.0316 0.3434
32 C 3.6186 -2.8735 0.5979
33 C 2.4835 -0.5813 3.2142
34 C 3.3976 -1.3720 3.9372
35 C 4.1202 -0.8572 5.0166
36 C 3.9584 0.4802 5.3892
37 C 3.0779 1.2866 4.6705
38 C 2.3260 0.7737 3.5986
39 C 1.4281 1.7852 2.9455
40 N 0.1538 1.4126 2.6621
41 C -0.8436 2.4253 2.3256
42 C -2.1977 2.0253 2.8770
43 C -2.7922 0.8104 2.4942
44 C -4.0526 0.4518 2.9835
45 C -4.7373 1.3081 3.8546
46 C -4.1516 2.5198 4.2397
47 C -2.8869 2.8739 3.7557
48 C 0.0500 -1.9557 2.5630
49 C -0.2683 -1.7309 3.9151
50 C -1.4761 -2.2021 4.4479
51 C -2.3706 -2.9155 3.6446
52 C -2.0526 -3.1625 2.3020
53 C -0.8550 -2.6830 1.7658
54 O 1.8180 2.9406 2.7466
55 C 4.2974 1.8454 0.6744
56 C 4.3755 3.2294 0.8952
57 C 5.1596 3.7370 1.9388
58 C 5.8782 2.8700 2.7674
59 C 5.7961 1.4860 2.5605
60 C 5.0027 0.9774 1.5309
61 C 3.0043 2.5898 -1.7699
62 C 1.7472 3.2362 -1.8274
63 C 1.5856 4.3605 -2.6623

64 C 2.6434 4.8668 -3.4154
65 C 3.8870 4.2293 -3.3650
66 C 4.0549 3.1046 -2.5559
67 C 0.4561 2.8196 -1.1604
68 N 0.3713 2.8551 0.1919
69 C -0.9455 2.6983 0.8016
70 C -1.8236 3.9183 0.5668
71 C -3.1630 3.7698 0.1834
72 C -3.9867 4.8885 0.0182
73 C -3.4747 6.1736 0.2355
74 C -2.1365 6.3309 0.6184
75 C -1.3163 5.2091 0.7840
76 O -0.5275 2.5779 -1.8658
77 H -0.5111 3.3607 2.8092
78 H -1.4208 1.8245 0.3263
79 H -0.2668 5.3385 1.0784
80 H -1.7277 7.3342 0.7889
81 H -4.1168 7.0528 0.1036
82 H -5.0319 4.7555 -0.2866
83 H -3.5606 2.7620 0.0163
84 H -2.2731 0.1314 1.8032
85 H -4.5019 -0.4998 2.6757
86 H -5.7280 1.0303 4.2335
87 H -4.6813 3.1949 4.9224
88 H -2.4309 3.8255 4.0554
89 H 1.1568 3.1450 0.7820
90 H -0.1832 0.4936 2.9504
91 H 5.0322 2.6120 -2.5419
92 H 4.7292 4.6011 -3.9602
93 H 2.4927 5.7488 -4.0486
94 H 0.5995 4.8358 -2.7139
95 H 3.5526 -2.4173 3.6505
96 H 4.8154 -1.5067 5.5614
97 H 4.5255 0.8985 6.2289
98 H 2.9567 2.3435 4.9304
99 H 1.4607 -0.8503 -3.0716
100 H 0.1172 -2.2947 -1.5359
101 H 0.3572 0.5960 -2.6997
102 H -1.8256 -1.2790 -0.2389
103 H -1.5819 0.3040 -1.2092
104 H 0.4285 -1.1906 4.5673
105 H -1.7119 -2.0103 5.5015
106 H -3.3159 -3.2807 4.0631
107 H -2.7390 -3.7169 1.6525
108 H -0.6212 -2.8916 0.7155
109 H 1.3324 -4.1628 2.7928
110 H 2.6355 -6.2186 2.3233
111 H 4.5820 -6.1501 0.7554
112 H 5.2098 -3.9897 -0.3481
113 H 3.8875 -1.9353 0.0980
114 H 3.8266 3.9234 0.2488

115 H 5.2113 4.8209 2.0980
116 H 6.4992 3.2697 3.5780
117 H 6.3487 0.7971 3.2099
118 H 4.9479 -0.1078 1.3792
119 H 6.3204 1.4612 -0.8989
120 H 8.0759 0.2102 -2.1105
121 H 7.4787 -1.7857 -3.4964
122 H 5.0873 -2.5147 -3.6480
123 H 3.3260 -1.2644 -2.4206
124 H -3.3494 0.0485 -3.6893
125 H -2.0443 -0.4271 -5.5122
126 H -0.8426 -2.4126 -6.0263
127 H -0.7394 -4.3907 -4.7188
128 H -1.6338 -4.7991 -2.5968
129 H -5.0404 -3.0084 -1.1217
130 H -3.6380 1.0307 -1.7961
131 H -7.0067 -2.1228 0.0722
132 H -5.6147 1.9100 -0.6128
133 H -7.3172 0.3500 0.3527

C(R)
Energy (FREE) = -2.363272060616793E6 kcal.mol^{-1}
Atom X Y Z
1 C 2.6255 -1.5226 3.5744
2 C 1.7121 -0.7954 2.7875
3 C 1.4917 0.5682 3.1030
4 C 2.1882 1.1506 4.1782
5 C 3.0653 0.4063 4.9639
6 C 3.2862 -0.9390 4.6571
7 P 0.8799 -1.6584 1.3725
8 C -0.6155 -2.3547 2.2070
9 C -1.0789 -1.8833 3.4497
10 C -2.2544 -2.3981 4.0123
11 C -2.9707 -3.4014 3.3541
12 C -2.4952 -3.9071 2.1368
13 C -1.3281 -3.3904 1.5698
14 C 0.5789 1.5248 2.3908
15 O 0.9342 2.6930 2.1940
16 Pd 0.6314 -0.5865 -0.7501
17 P 2.5312 0.7690 -1.0843
18 C 3.5268 1.4135 0.3207
19 C 3.6297 2.7848 0.6028
20 C 4.4422 3.2306 1.6523
21 C 5.1606 2.3139 2.4256
22 C 5.0508 0.9425 2.1592
23 C 4.2312 0.4939 1.1225
24 C -0.5107 -1.5956 -2.3296
25 C -0.0319 -0.3884 -2.8799
26 C -1.3627 -1.5419 -1.2110
27 C -3.8977 -2.6753 -2.3772
28 C -4.4609 -1.6626 -1.4658

29 O -4.6827 -1.9588 -0.2636
30 C -3.7043 -4.0399 -1.8381
31 C -2.5584 -4.8002 -2.1774
32 C -2.3656 -6.0996 -1.6977
33 C -3.3205 -6.6951 -0.8634
34 C -4.4702 -5.9662 -0.5243
35 C -4.6572 -4.6644 -0.9947
36 C -3.5272 -2.4694 -3.7337
37 C -3.5176 -1.3441 -4.5580
38 C -3.9382 -0.0264 -4.2630
39 C -4.4970 0.4093 -3.0552
40 C -4.7503 -0.2918 -1.8743
41 C 1.8932 -3.1746 1.1045
42 C 1.7534 -4.3284 1.8994
43 C 2.5499 -5.4520 1.6567
44 C 3.4935 -5.4399 0.6217
45 C 3.6384 -4.2975 -0.1729
46 C 2.8397 -3.1740 0.0659
47 C 2.2974 2.3022 -2.1174
48 C 1.0828 3.0171 -2.2538
49 C 1.0452 4.1629 -3.0746
50 C 2.1822 4.6329 -3.7272
51 C 3.3856 3.9342 -3.5916
52 C 3.4313 2.7836 -2.8044
53 C -0.2737 2.6637 -1.6943
54 O -1.2285 2.5399 -2.4659
55 C 3.8173 -0.1903 -2.0178
56 C 3.4187 -1.2349 -2.8712
57 C 4.3636 -1.9736 -3.5916
58 C 5.7264 -1.6847 -3.4646
59 C 6.1374 -0.6563 -2.6088
60 C 5.1933 0.0835 -1.8892
61 N -0.4250 2.5700 -0.3539
62 C -1.7700 2.3836 0.1851
63 C -1.6943 2.0927 1.7047
64 C -3.0328 1.6568 2.2656
65 C -3.6805 0.5070 1.7832
66 C -4.9115 0.1091 2.3158
67 C -5.5088 0.8560 3.3384
68 C -4.8697 2.0028 3.8252
69 C -3.6381 2.3990 3.2918
70 C -2.6613 3.5948 -0.0468
71 C -3.9890 3.4335 -0.4669
72 C -4.8252 4.5445 -0.6233
73 C -4.3399 5.8314 -0.3607
74 C -3.0144 6.0000 0.0592
75 C -2.1810 4.8871 0.2172
76 N -0.6675 1.1064 2.0496
77 H -1.3862 3.0319 2.1967
78 H -2.2167 1.5171 -0.3337
79 H -1.1414 5.0251 0.5416

80 H -2.6261 7.0048 0.2652
81 H -4.9933 6.7032 -0.4860
82 H -5.8602 4.4036 -0.9573
83 H -4.3669 2.4254 -0.6768
84 H -3.2452 -0.0978 0.9771
85 H -5.3947 -0.7829 1.9022
86 H -6.4756 0.5472 3.7539
87 H -5.3325 2.5950 4.6238
88 H -3.1408 3.3003 3.6716
89 H 0.3267 2.8267 0.2959
90 H -0.9935 0.1805 2.3259
91 H 4.3804 2.2446 -2.7288
92 H 4.2918 4.2762 -4.1047
93 H 2.1258 5.5350 -4.3472
94 H 0.0896 4.6858 -3.1909
95 H 2.8342 -2.5700 3.3360
96 H 3.9825 -1.5402 5.2532
97 H 3.5845 0.8798 5.8051
98 H 2.0205 2.2117 4.3890
99 H 0.7075 -0.4054 -3.6905
100 H -0.0777 -2.5593 -2.6369
101 H -0.6594 0.5106 -2.8242
102 H -1.6951 -2.4530 -0.7107
103 H -1.9657 -0.6401 -1.0328
104 H -0.5180 -1.1222 4.0052
105 H -2.6029 -2.0110 4.9770
106 H -3.8921 -3.8005 3.7937
107 H -3.0354 -4.7073 1.6170
108 H -0.9553 -3.8220 0.6341
109 H 1.0210 -4.3536 2.7140
110 H 2.4299 -6.3445 2.2820
111 H 4.1123 -6.3249 0.4324
112 H 4.3690 -4.2782 -0.9903
113 H 2.9456 -2.2884 -0.5702
114 H 3.0793 3.5159 0.0009
115 H 4.5149 4.3048 1.8598
116 H 5.8039 2.6658 3.2410
117 H 5.6038 0.2160 2.7654
118 H 4.1591 -0.5820 0.9208
119 H 5.5391 0.8777 -1.2191
120 H 7.2032 -0.4256 -2.4953
121 H 6.4686 -2.2656 -4.0246
122 H 4.0283 -2.7855 -4.2474
123 H 2.3598 -1.4964 -2.9552
124 H -5.2008 0.2802 -1.0518
125 H -4.7831 1.4719 -3.0279
126 H -3.8365 0.7205 -5.0610
127 H -3.1533 -1.5161 -5.5806
128 H -3.1779 -3.3816 -4.2378
129 H -5.5616 -4.1174 -0.7184
130 H -5.2367 -6.4213 0.1163

131 H -3.1737 -7.7140 -0.4850
132 H -1.4562 -6.6483 -1.9742
133 H -1.7879 -4.3494 -2.8148

B
Energy (FREE) = -2.0019807892113235E6 kcal.mol^{-1}
Atom X Y Z
1 C -5.1248 1.8348 -1.1595
2 C -4.1554 1.9902 -0.1589
3 C -4.3841 2.9079 0.8781
4 C -5.5639 3.6598 0.9122
5 C -6.5296 3.4973 -0.0890
6 C -6.3068 2.5825 -1.1255
7 C -2.9020 1.1294 -0.1560
8 N -1.6892 1.9014 0.1062
9 C -1.3148 2.8373 -0.7942
10 O -1.9434 3.0194 -1.8401
11 C -0.1382 3.7261 -0.4791
12 C -0.4663 5.0961 -0.5234
13 C 0.4925 6.0845 -0.3146
14 C 1.8203 5.7106 -0.0882
15 C 2.1648 4.3590 -0.0688
16 C 1.2060 3.3416 -0.2551
17 P 1.8309 1.5892 -0.1530
18 Pd 0.6198 -0.0252 -1.3745
19 C -0.5579 -0.8574 -3.0571
20 C 0.4208 0.0236 -3.5541
21 C 0.4195 1.3576 -3.0868
22 P 0.8136 -2.0761 -0.1612
23 C 0.9364 -2.0864 1.6895
24 C 0.1192 -1.2948 2.5340
25 C 0.2930 -1.3632 3.9279
26 C 1.2281 -2.2231 4.5006
27 C 2.0350 -3.0070 3.6727
28 C 1.8968 -2.9252 2.2853
29 C -0.9538 -0.3381 2.1035
30 O -1.0881 0.7505 2.6724
31 N -1.8102 -0.7322 1.1250
32 C -3.0546 0.0065 0.9015
33 C -4.1679 -0.9490 0.5249
34 C -4.0515 -1.7743 -0.6068
35 C -5.0869 -2.6467 -0.9581
36 C -6.2542 -2.6976 -0.1864
37 C -6.3784 -1.8764 0.9401
38 C -5.3387 -1.0097 1.2952
39 H -3.3010 0.4941 1.8611
40 H -2.7916 0.6696 -1.1546
41 H -3.6287 3.0420 1.6633
42 H -5.7298 4.3763 1.7256
43 H -7.4540 4.0865 -0.0633
44 H -7.0554 2.4545 -1.9165

45 H -4.9482 1.1224 -1.9752
46 H -3.1438 -1.7406 -1.2248
47 H -4.9815 -3.2876 -1.8415
48 H -7.0679 -3.3779 -0.4643
49 H -7.2897 -1.9108 1.5488
50 H -5.4386 -0.3655 2.1774
51 H -1.2779 1.8591 1.0459
52 H -1.7784 -1.6925 0.7837
53 H 3.2142 4.0960 0.0921
54 H 2.5965 6.4688 0.0656
55 H 0.2056 7.1419 -0.3395
56 H -1.5063 5.3740 -0.7268
57 H 2.5600 -3.5273 1.6570
58 H 2.7869 -3.6795 4.1013
59 H 1.3344 -2.2704 5.5903
60 H -0.3332 -0.7250 4.5596
61 C 3.5568 1.6866 -0.8230
62 C 2.1517 1.4054 1.6458
63 C 2.3689 -2.8824 -0.7291
64 C -0.4251 -3.4249 -0.4404
65 H 1.2437 2.0329 -3.3495
66 H 1.2867 -0.3793 -4.1003
67 H -0.5380 1.8436 -2.8569
68 H -0.5283 -1.9152 -3.3309
69 H -1.5335 -0.4632 -2.7330
70 C -1.2189 -3.9424 0.6000
71 C -2.1554 -4.9534 0.3472
72 C -2.2958 -5.4777 -0.9399
73 C -1.4805 -4.9991 -1.9739
74 C -0.5546 -3.9827 -1.7275
75 H -1.1025 -3.5810 1.6284
76 H -2.7686 -5.3357 1.1713
77 H -3.0270 -6.2703 -1.1365
78 H -1.5591 -5.4248 -2.9810
79 H 0.1045 -3.6561 -2.5387
80 C 2.5248 -4.2815 -0.7546
81 C 3.7316 -4.8467 -1.1787
82 C 4.7939 -4.0274 -1.5806
83 C 4.6465 -2.6363 -1.5572
84 C 3.4393 -2.0685 -1.1365
85 H 1.7062 -4.9372 -0.4382
86 H 3.8410 -5.9373 -1.1943
87 H 5.7365 -4.4757 -1.9158
88 H 5.4696 -1.9849 -1.8736
89 H 3.3272 -0.9791 -1.1319
90 C 1.6074 2.2870 2.5923
91 C 1.9407 2.1597 3.9460
92 C 2.8190 1.1557 4.3631
93 C 3.3530 0.2623 3.4249
94 C 3.0135 0.3780 2.0763
95 H 0.9258 3.0849 2.2791

96 H 1.5118 2.8567 4.6754
97 H 3.0870 1.0646 5.4224
98 H 4.0371 -0.5316 3.7452
99 H 3.4462 -0.3213 1.3504
100 C 4.6371 2.1107 -0.0237
101 C 5.9295 2.1812 -0.5531
102 C 6.1682 1.8242 -1.8851
103 C 5.1060 1.3894 -2.6845
104 C 3.8125 1.3185 -2.1563
105 H 4.4773 2.3859 1.0241
106 H 6.7553 2.5157 0.0854
107 H 7.1830 1.8762 -2.2962
108 H 5.2809 1.0931 -3.7252
109 H 3.0025 0.9424 -2.7874

D(R)
Energy (FREE) = -2.3632707991020647E6 kcal.mol^{-1}
Atom X Y Z
1 C 3.5365 -2.0527 4.8410
2 C 2.7153 -1.1540 4.1342
3 C 2.8044 0.2262 4.4513
4 C 3.7102 0.6570 5.4353
5 C 4.5065 -0.2512 6.1342
6 C 4.4170 -1.6135 5.8348
7 P 1.6587 -1.7760 2.7345
8 C 0.0546 -2.1308 3.5800
9 C -0.1282 -2.0631 4.9733
10 C -1.3852 -2.3261 5.5345
11 C -2.4650 -2.6711 4.7146
12 C -2.2898 -2.7427 3.3259
13 C -1.0409 -2.4660 2.7629
14 C 2.0283 1.3367 3.7945
15 O 2.5786 2.4007 3.4899
16 Pd 1.9934 -0.3548 1.0030
17 P 3.6801 0.7670 -0.0323
18 C 4.9109 1.3530 1.2146
19 C 5.2226 2.7063 1.4191
20 C 6.1356 3.0810 2.4143
21 C 6.7534 2.1100 3.2080
22 C 6.4472 0.7560 3.0096
23 C 5.5261 0.3820 2.0294
24 C 0.7049 -0.7690 -2.7583
25 C 1.1373 0.3019 -3.4409
26 C -0.3230 -0.7080 -1.6654
27 C -1.4420 -1.7922 -1.8132
28 C -2.5076 -1.5198 -0.7061
29 O -2.7725 -2.3811 0.1296
30 C -0.9202 -3.2173 -1.6151
31 C 0.2825 -3.5016 -0.9474
32 C 0.6933 -4.8247 -0.7358
33 C -0.0935 -5.8906 -1.1823

34 C -1.3007 -5.6221 -1.8391
35 C -1.7056 -4.3007 -2.0484
36 C -2.0481 -1.6371 -3.1899
37 C -2.7493 -0.5646 -3.6872
38 C -3.2525 0.5657 -2.9922
39 C -3.4245 0.7104 -1.5997
40 C -3.1525 -0.2117 -0.6025
41 C 2.3219 -3.4724 2.4367
42 C 1.7627 -4.6328 2.9985
43 C 2.3131 -5.8899 2.7168
44 C 3.4320 -6.0004 1.8835
45 C 3.9969 -4.8472 1.3217
46 C 3.4386 -3.5936 1.5877
47 C 3.4072 2.3021 -1.0376
48 C 2.1837 3.0090 -0.9926
49 C 2.0291 4.1723 -1.7733
50 C 3.0652 4.6507 -2.5738
51 C 4.2741 3.9473 -2.6308
52 C 4.4331 2.7861 -1.8734
53 C 0.9134 2.5961 -0.2832
54 O -0.1063 2.4159 -0.9533
55 C 4.7424 -0.3030 -1.1040
56 C 4.1319 -1.3921 -1.7512
57 C 4.8809 -2.2578 -2.5556
58 C 6.2574 -2.0540 -2.7107
59 C 6.8797 -0.9850 -2.0541
60 C 6.1295 -0.1161 -1.2535
61 N 0.8866 2.5611 1.0725
62 C -0.3997 2.4046 1.7491
63 C -0.1846 2.2742 3.2839
64 C -1.5096 2.1513 4.0100
65 C -2.3156 1.0131 3.8407
66 C -3.5501 0.9151 4.4916
67 C -3.9963 1.9569 5.3138
68 C -3.1974 3.0930 5.4886
69 C -1.9595 3.1867 4.8426
70 C -1.3258 3.5802 1.4692
71 C -2.6883 3.3713 1.2123
72 C -3.5526 4.4547 1.0229
73 C -3.0614 5.7648 1.0882
74 C -1.7015 5.9817 1.3419
75 C -0.8397 4.8952 1.5317
76 N 0.6886 1.1634 3.6522
77 H 0.3274 3.1948 3.6167
78 H -0.8786 1.4785 1.3783
79 H 0.2269 5.0709 1.7219
80 H -1.3076 7.0041 1.3917
81 H -3.7370 6.6154 0.9377
82 H -4.6150 4.2747 0.8188
83 H -3.0740 2.3457 1.1627
84 H -1.9860 0.1897 3.1922

85 H -4.1664 0.0192 4.3510
86 H -4.9662 1.8822 5.8199
87 H -3.5380 3.9120 6.1332
88 H -1.3367 4.0795 4.9778
89 H 1.7197 2.7500 1.6393
90 H 0.2534 0.3092 4.0014
91 H 5.3812 2.2410 -1.9347
92 H 5.0934 4.2995 -3.2683
93 H 2.9242 5.5639 -3.1637
94 H 1.0676 4.6980 -1.7481
95 H 3.4947 -3.1213 4.6039
96 H 5.0388 -2.3422 6.3682
97 H 5.2015 0.1065 6.9027
98 H 3.7832 1.7303 5.6414
99 H 1.9102 0.2092 -4.2166
100 H 1.1219 -1.7609 -2.9936
101 H 0.7466 1.3061 -3.2210
102 H 0.1663 -0.8235 -0.6641
103 H -0.7784 0.2941 -1.6538
104 H 0.7125 -1.8054 5.6289
105 H -1.5167 -2.2636 6.6214
106 H -3.4473 -2.8757 5.1570
107 H -3.1278 -2.9952 2.6668
108 H -0.9214 -2.5123 1.6729
109 H 0.8917 -4.5604 3.6601
110 H 1.8631 -6.7885 3.1556
111 H 3.8605 -6.9859 1.6654
112 H 4.8676 -4.9248 0.6596
113 H 3.8590 -2.6902 1.1245
114 H 4.7492 3.4809 0.8055
115 H 6.3646 4.1430 2.5650
116 H 7.4701 2.4052 3.9837
117 H 6.9212 -0.0134 3.6308
118 H 5.2780 -0.6788 1.8941
119 H 6.6353 0.7035 -0.7296
120 H 7.9599 -0.8279 -2.1599
121 H 6.8488 -2.7360 -3.3333
122 H 4.3876 -3.1022 -3.0519
123 H 3.0589 -1.5648 -1.6046
124 H -3.5342 -0.0060 0.4067
125 H -3.9138 1.6383 -1.2739
126 H -3.6372 1.3848 -3.6125
127 H -2.9365 -0.5765 -4.7699
128 H -1.7885 -2.4264 -3.9066
129 H -2.6635 -4.1084 -2.5480
130 H -1.9356 -6.4461 -2.1865
131 H 0.2297 -6.9254 -1.0165
132 H 1.6380 -5.0154 -0.2125
133 H 0.9231 -2.6935 -0.5700

Cprime(R)

Energy (FREE) = -2.3636493133787937E6 kcal.mol^{-1}
Atom X Y Z
1 C 2.5939 -1.4835 3.5099
2 C 1.6688 -0.7454 2.7484
3 C 1.4837 0.6245 3.0589
4 C 2.2255 1.2026 4.1052
5 C 3.1144 0.4480 4.8680
6 C 3.3007 -0.9031 4.5653
7 P 0.7533 -1.6132 1.3886
8 C -0.7307 -2.2225 2.3134
9 C -1.0702 -1.7380 3.5916
10 C -2.2151 -2.2041 4.2499
11 C -3.0242 -3.1747 3.6530
12 C -2.6755 -3.6887 2.3981
13 C -1.5411 -3.2177 1.7330
14 C 0.5576 1.5870 2.3723
15 O 0.9109 2.7546 2.1690
16 Pd 0.5132 -0.5752 -0.7470
17 P 2.4393 0.7302 -1.1181
18 C 3.4727 1.3697 0.2627
19 C 3.5935 2.7414 0.5339
20 C 4.4403 3.1866 1.5564
21 C 5.1749 2.2685 2.3120
22 C 5.0471 0.8966 2.0566
23 C 4.1933 0.4486 1.0476
24 C -0.6424 -1.5526 -2.3402
25 C -0.1262 -0.3398 -2.8482
26 C -1.4854 -1.4899 -1.2176
27 C -3.8786 -2.8742 -2.4225
28 C -4.5151 -1.9687 -1.4944
29 O -4.5357 -2.1091 -0.2492
30 C -3.4410 -4.2042 -1.9586
31 C -2.3649 -4.8812 -2.5907
32 C -1.9432 -6.1503 -2.1809
33 C -2.5746 -6.7998 -1.1127
34 C -3.6407 -6.1524 -0.4707
35 C -4.0629 -4.8850 -0.8795
36 C -3.6750 -2.5631 -3.8086
37 C -3.5171 -1.3444 -4.4542
38 C -3.5298 -0.0588 -3.8166
39 C -4.2224 0.2122 -2.6736
40 C -5.2236 -0.7544 -2.1172
41 C 1.6987 -3.1738 1.1310
42 C 1.5142 -4.3133 1.9364
43 C 2.2644 -5.4702 1.7014
44 C 3.2060 -5.5047 0.6655
45 C 3.3968 -4.3749 -0.1377
46 C 2.6443 -3.2186 0.0926
47 C 2.2250 2.2604 -2.1619
48 C 1.0334 3.0178 -2.2740
49 C 1.0192 4.1630 -3.0966

50 C 2.1567 4.5909 -3.7765
51 C 3.3362 3.8485 -3.6677
52 C 3.3585 2.6993 -2.8774
53 C -0.3260 2.7131 -1.6932
54 O -1.2932 2.6215 -2.4539
55 C 3.6857 -0.2756 -2.0560
56 C 3.2518 -1.3411 -2.8653
57 C 4.1686 -2.1159 -3.5843
58 C 5.5378 -1.8427 -3.5001
59 C 5.9841 -0.7941 -2.6874
60 C 5.0684 -0.0188 -1.9689
61 N -0.4633 2.6239 -0.3512
62 C -1.8047 2.4718 0.2044
63 C -1.7287 2.1523 1.7215
64 C -3.0676 1.6823 2.2542
65 C -3.6721 0.5170 1.7514
66 C -4.9088 0.0876 2.2439
67 C -5.5558 0.8195 3.2473
68 C -4.9596 1.9799 3.7554
69 C -3.7212 2.4071 3.2620
70 C -2.6653 3.7119 0.0052
71 C -4.0366 3.5929 -0.2646
72 C -4.8402 4.7323 -0.3838
73 C -4.2801 6.0064 -0.2313
74 C -2.9128 6.1330 0.0434
75 C -2.1117 4.9920 0.1640
76 N -0.6948 1.1693 2.0532
77 H -1.4388 3.0862 2.2344
78 H -2.2806 1.6254 -0.3214
79 H -1.0402 5.0976 0.3767
80 H -2.4657 7.1271 0.1648
81 H -4.9081 6.9001 -0.3284
82 H -5.9093 4.6230 -0.6015
83 H -4.4797 2.5963 -0.3807
84 H -3.1928 -0.0759 0.9617
85 H -5.3557 -0.8169 1.8163
86 H -6.5279 0.4876 3.6316
87 H -5.4614 2.5592 4.5397
88 H -3.2583 3.3197 3.6577
89 H 0.3013 2.8678 0.2883
90 H -1.0200 0.2501 2.3528
91 H 4.2894 2.1276 -2.8232
92 H 4.2419 4.1550 -4.2034
93 H 2.1185 5.4934 -4.3971
94 H 0.0804 4.7191 -3.1931
95 H 2.7733 -2.5372 3.2748
96 H 4.0055 -1.5121 5.1433
97 H 3.6692 0.9182 5.6879
98 H 2.0828 2.2679 4.3130
99 H 0.6169 -0.3476 -3.6562
100 H -0.2407 -2.5225 -2.6685

101 H -0.7486 0.5621 -2.7829
102 H -1.8460 -2.3967 -0.7294
103 H -2.0747 -0.5783 -1.0401
104 H -0.4350 -1.0034 4.0999
105 H -2.4645 -1.8068 5.2407
106 H -3.9204 -3.5395 4.1680
107 H -3.2910 -4.4600 1.9216
108 H -1.2733 -3.6574 0.7657
109 H 0.7848 -4.3020 2.7538
110 H 2.1093 -6.3519 2.3342
111 H 3.7880 -6.4155 0.4824
112 H 4.1276 -4.3911 -0.9550
113 H 2.7879 -2.3421 -0.5487
114 H 3.0317 3.4737 -0.0559
115 H 4.5270 4.2613 1.7559
116 H 5.8455 2.6196 3.1054
117 H 5.6131 0.1693 2.6496
118 H 4.1076 -0.6276 0.8536
119 H 5.4425 0.7890 -1.3311
120 H 7.0554 -0.5759 -2.6065
121 H 6.2578 -2.4518 -4.0591
122 H 3.8059 -2.9436 -4.2048
123 H 2.1885 -1.5930 -2.9152
124 H -5.8394 -0.2878 -1.3291
125 H -4.0644 1.1707 -2.1586
126 H -2.9223 0.7419 -4.2663
127 H -3.2472 -1.3819 -5.5199
128 H -3.5225 -3.4414 -4.4562
129 H -4.8970 -4.4037 -0.3631
130 H -4.1588 -6.6475 0.3614
131 H -2.2431 -7.7928 -0.7855
132 H -1.1021 -6.6311 -2.6974
133 H -1.8334 -4.3871 -3.4126
134 H -5.8870 -1.1078 -2.9353

A
Energy (FREE) =-361276.20427540975 kcal.mol^{-1}
Atom X Y Z
1 C -0.7588 0.0317 0.9395
2 C 0.5385 -0.5067 1.1519
3 C 1.7418 0.1762 0.9679
4 C -1.1166 1.3013 0.4932
5 C 1.9784 1.4938 0.5538
6 C -0.3776 2.4652 0.1205
7 C 1.0956 2.5872 0.1586
8 H 3.0312 1.8006 0.4816
9 H -1.6010 -0.6375 1.1678
10 H 0.5987 -1.5473 1.4980
11 H 2.6526 -0.4051 1.1824
12 H -2.2055 1.4471 0.4525
13 O 1.6517 3.6602 -0.1860

14 C -1.1608 3.6113 -0.3738
15 C -2.3472 3.4107 -1.1319
16 C -0.8160 4.9642 -0.1004
17 C -3.1359 4.4748 -1.5763
18 H -2.6392 2.3895 -1.4054
19 C -1.6071 6.0259 -0.5435
20 H 0.0827 5.1698 0.4837
21 C -2.7758 5.7982 -1.2873
22 H -4.0365 4.2664 -2.1691
23 H -1.3107 7.0538 -0.2949
24 H -3.3916 6.6364 -1.6358

TS to-Dprime(R)
Energy (FREE) = -2.3636453031318113E6 kcal.mol^{-1}
Atom X Y Z
1 C 2.6674 -1.2655 3.7446
2 C 1.7696 -0.5568 2.9240
3 C 1.5796 0.8243 3.1784
4 C 2.2878 1.4396 4.2275
5 C 3.1508 0.7141 5.0457
6 C 3.3430 -0.6478 4.7995
7 P 0.9235 -1.4597 1.5373
8 C -0.5851 -2.0897 2.4027
9 C -1.0021 -1.6206 3.6618
10 C -2.1746 -2.1139 4.2501
11 C -2.9312 -3.0930 3.6002
12 C -2.5090 -3.5840 2.3575
13 C -1.3497 -3.0839 1.7615
14 C 0.6861 1.7692 2.4263
15 O 1.0509 2.9302 2.2059
16 Pd 0.6966 -0.4829 -0.6265
17 P 2.6034 0.8002 -1.0537
18 C 3.6104 1.5243 0.3085
19 C 3.7392 2.9093 0.4989
20 C 4.5505 3.4101 1.5246
21 C 5.2449 2.5348 2.3649
22 C 5.1111 1.1508 2.1893
23 C 4.2906 0.6490 1.1779
24 C -0.6715 -1.5538 -1.9636
25 C -0.0404 -0.4849 -2.6610
26 C -1.8105 -1.3326 -1.1674
27 C -3.7025 -2.2672 -2.3459
28 C -4.5594 -1.8821 -1.2200
29 O -4.4772 -2.3629 -0.0785
30 C -3.1563 -3.6477 -2.4033
31 C -2.1721 -3.9930 -3.3633
32 C -1.6579 -5.2898 -3.4617
33 C -2.1015 -6.2986 -2.5990
34 C -3.0720 -5.9810 -1.6395
35 C -3.5865 -4.6853 -1.5390
36 C -3.7149 -1.5166 -3.5864

37 C -3.8888 -0.1660 -3.8029
38 C -4.2114 0.8156 -2.7992
39 C -4.9500 0.5299 -1.6924
40 C -5.6073 -0.8011 -1.5087
41 C 1.9035 -3.0156 1.3681
42 C 1.7102 -4.1325 2.2030
43 C 2.4808 -5.2868 2.0282
44 C 3.4529 -5.3424 1.0215
45 C 3.6517 -4.2368 0.1872
46 C 2.8783 -3.0835 0.3582
47 C 2.4198 2.2645 -2.1893
48 C 1.2271 3.0110 -2.3383
49 C 1.2028 4.1030 -3.2303
50 C 2.3343 4.4870 -3.9461
51 C 3.5161 3.7537 -3.8012
52 C 3.5474 2.6565 -2.9400
53 C -0.1230 2.7424 -1.7197
54 O -1.1016 2.6185 -2.4607
55 C 3.8829 -0.2399 -1.9082
56 C 3.4702 -1.3367 -2.6858
57 C 4.4052 -2.1412 -3.3464
58 C 5.7723 -1.8665 -3.2335
59 C 6.1972 -0.7849 -2.4531
60 C 5.2626 0.0209 -1.7943
61 N -0.2441 2.7426 -0.3707
62 C -1.5889 2.6992 0.1980
63 C -1.5673 2.3491 1.7137
64 C -2.9376 1.8905 2.1717
65 C -3.4635 0.6636 1.7316
66 C -4.7285 0.2374 2.1496
67 C -5.4882 1.0424 3.0077
68 C -4.9748 2.2686 3.4472
69 C -3.7045 2.6881 3.0341
70 C -2.3175 4.0228 0.0067
71 C -3.6209 4.0662 -0.5046
72 C -4.2949 5.2857 -0.6405
73 C -3.6671 6.4793 -0.2663
74 C -2.3630 6.4457 0.2446
75 C -1.6939 5.2250 0.3813
76 N -0.5596 1.3533 2.0788
77 H -1.2930 3.2705 2.2562
78 H -2.1376 1.9112 -0.3452
79 H -0.6714 5.2070 0.7803
80 H -1.8632 7.3764 0.5392
81 H -4.1918 7.4361 -0.3750
82 H -5.3134 5.3020 -1.0465
83 H -4.1102 3.1324 -0.8036
84 H -2.8859 0.0229 1.0521
85 H -5.1094 -0.7239 1.7842
86 H -6.4826 0.7144 3.3336
87 H -5.5649 2.9037 4.1187

88 H -3.3039 3.6488 3.3806
89 H 0.5343 2.9983 0.2446
90 H -0.8955 0.4386 2.3809
91 H 4.4784 2.0881 -2.8565
92 H 4.4163 4.0276 -4.3635
93 H 2.2899 5.3483 -4.6224
94 H 0.2630 4.6522 -3.3529
95 H 2.8519 -2.3274 3.5550
96 H 4.0272 -1.2361 5.4220
97 H 3.6807 1.2142 5.8646
98 H 2.1417 2.5123 4.3900
99 H 0.6979 -0.7073 -3.4419
100 H -0.2913 -2.5807 -2.0644
101 H -0.5985 0.4509 -2.8011
102 H -2.1493 -2.0500 -0.4164
103 H -2.2455 -0.3254 -1.1310
104 H -0.4071 -0.8779 4.2067
105 H -2.4882 -1.7318 5.2288
106 H -3.8470 -3.4796 4.0623
107 H -3.0906 -4.3556 1.8409
108 H -1.0234 -3.4886 0.7957
109 H 0.9556 -4.1046 2.9971
110 H 2.3182 -6.1498 2.6847
111 H 4.0516 -6.2508 0.8852
112 H 4.4048 -4.2703 -0.6091
113 H 3.0253 -2.2280 -0.3098
114 H 3.2103 3.6087 -0.1581
115 H 4.6422 4.4944 1.6599
116 H 5.8877 2.9288 3.1612
117 H 5.6451 0.4565 2.8480
118 H 4.1978 -0.4365 1.0496
119 H 5.6184 0.8575 -1.1837
120 H 7.2664 -0.5641 -2.3518
121 H 6.5070 -2.4991 -3.7455
122 H 4.0592 -2.9932 -3.9433
123 H 2.4063 -1.5838 -2.7526
124 H -6.3186 -0.7878 -0.6670
125 H -5.0930 1.2848 -0.9073
126 H -3.8440 1.8415 -2.9483
127 H -3.6992 0.1996 -4.8227
128 H -3.4491 -2.0933 -4.4831
129 H -4.3449 -4.4701 -0.7831
130 H -3.4431 -6.7574 -0.9580
131 H -1.6982 -7.3157 -2.6726
132 H -0.8952 -5.5089 -4.2198
133 H -1.7853 -3.2251 -4.0417
134 H -6.1405 -1.0819 -2.4417

Cprime(S)
Energy (FREE) = -2.363646949485873E6 kcal.mol^{-1}
Atom X Y Z

1 C 2.2898 -1.2184 3.7996
2 C 1.5305 -0.4982 2.8582
3 C 1.4459 0.9087 2.9995
4 C 2.1217 1.5400 4.0598
5 C 2.8436 0.8064 4.9990
6 C 2.9283 -0.5817 4.8658
7 P 0.7121 -1.4356 1.4820
8 C -0.9053 -1.8798 2.2634
9 C -1.3985 -1.2250 3.4067
10 C -2.6512 -1.5645 3.9356
11 C -3.4169 -2.5722 3.3436
12 C -2.9159 -3.2595 2.2295
13 C -1.6709 -2.9182 1.6962
14 C 0.7043 1.8607 2.1057
15 O 1.2121 2.9426 1.7893
16 Pd 0.7217 -0.6056 -0.7541
17 P 2.7942 0.4731 -1.0820
18 C 3.7574 1.1341 0.3388
19 C 4.0096 2.5054 0.4995
20 C 4.7990 2.9575 1.5638
21 C 5.3441 2.0466 2.4731
22 C 5.0836 0.6777 2.3276
23 C 4.2867 0.2247 1.2750
24 C -0.4504 -1.6374 -2.2997
25 C 0.2180 -0.5538 -2.9110
26 C -1.3136 -1.3600 -1.2236
27 C -3.2680 -0.0836 -3.8782
28 C -3.4341 -1.5628 -3.4806
29 O -2.8828 -2.4340 -4.1874
30 C -4.5701 0.5170 -4.3084
31 C -5.5358 0.8244 -3.3978
32 C -5.4792 0.4059 -2.0215
33 C -4.8892 -0.7638 -1.5706
34 C -4.1933 -1.8085 -2.2762
35 C 1.5634 -3.0703 1.4604
36 C 1.2589 -4.0892 2.3837
37 C 1.9334 -5.3129 2.3300
38 C 2.9157 -5.5370 1.3569
39 C 3.2213 -4.5313 0.4338
40 C 2.5456 -3.3073 0.4843
41 C 2.8359 1.9124 -2.2659
42 C 1.7407 2.7526 -2.5799
43 C 1.9218 3.8124 -3.4916
44 C 3.1623 4.0787 -4.0656
45 C 4.2489 3.2549 -3.7561
46 C 4.0778 2.1864 -2.8762
47 C 0.3064 2.6035 -2.1375
48 O -0.5761 2.4974 -2.9927
49 C 4.0072 -0.7266 -1.8115
50 C 3.5378 -1.7948 -2.5969
51 C 4.4277 -2.7134 -3.1639

52 C 5.8037 -2.5833 -2.9480
53 C 6.2826 -1.5323 -2.1575
54 C 5.3940 -0.6127 -1.5911
55 N 0.0215 2.6513 -0.8175
56 C -1.3764 2.6070 -0.3915
57 C -1.4388 2.5613 1.1557
58 C -2.8551 2.3663 1.6552
59 C -3.5366 1.1574 1.4365
60 C -4.8445 0.9870 1.9038
61 C -5.4898 2.0269 2.5834
62 C -4.8191 3.2368 2.7987
63 C -3.5076 3.4024 2.3409
64 C -2.1895 3.7853 -0.9013
65 C -3.4478 3.5738 -1.4822
66 C -4.2275 4.6583 -1.8974
67 C -3.7540 5.9668 -1.7373
68 C -2.4955 6.1837 -1.1621
69 C -1.7175 5.0974 -0.7446
70 N -0.5583 1.5381 1.7250
71 H -1.0570 3.5306 1.5223
72 H -1.8235 1.6812 -0.7970
73 H -0.7287 5.2724 -0.3004
74 H -2.1161 7.2052 -1.0379
75 H -4.3630 6.8177 -2.0655
76 H -5.2074 4.4782 -2.3560
77 H -3.8187 2.5478 -1.6155
78 H -3.0506 0.3347 0.8948
79 H -5.3620 0.0360 1.7307
80 H -6.5164 1.8941 2.9447
81 H -5.3180 4.0565 3.3292
82 H -2.9827 4.3504 2.5115
83 H 0.7343 2.8987 -0.1209
84 H -1.0041 0.7020 2.1016
85 H 4.9400 1.5473 -2.6646
86 H 5.2325 3.4345 -4.2048
87 H 3.2772 4.9187 -4.7601
88 H 1.0547 4.4324 -3.7444
89 H 2.3963 -2.3025 3.6979
90 H 3.5029 -1.1771 5.5847
91 H 3.3490 1.3200 5.8248
92 H 2.0628 2.6306 4.1354
93 H 0.9906 -0.7399 -3.6681
94 H -0.1418 -2.6742 -2.4960
95 H -0.2958 0.4123 -2.9886
96 H -1.7765 -2.1744 -0.6631
97 H -1.8128 -0.3829 -1.1598
98 H -0.8030 -0.4572 3.9151
99 H -3.0208 -1.0366 4.8224
100 H -4.3977 -2.8347 3.7565
101 H -3.4946 -4.0675 1.7657
102 H -1.2814 -3.4933 0.8485

103 H 0.4927 -3.9313 3.1508
104 H 1.6863 -6.0980 3.0541
105 H 3.4380 -6.5000 1.3157
106 H 3.9822 -4.6977 -0.3378
107 H 2.7777 -2.5309 -0.2524
108 H 3.5957 3.2308 -0.2088
109 H 4.9899 4.0313 1.6758
110 H 5.9699 2.4020 3.3004
111 H 5.5003 -0.0431 3.0402
112 H 4.0975 -0.8507 1.1690
113 H 5.7916 0.1957 -0.9685
114 H 7.3582 -1.4248 -1.9748
115 H 6.5021 -3.3051 -3.3875
116 H 4.0375 -3.5396 -3.7694
117 H 2.4632 -1.9322 -2.7479
118 H -5.0565 -0.9721 -0.5012
119 H -6.0532 0.9866 -1.2841
120 H -6.4169 1.3999 -3.7234
121 H -4.7258 0.7362 -5.3747
122 C -4.2495 -3.1594 -1.6886
123 C -5.3263 -3.5379 -0.8410
124 C -3.2516 -4.1488 -1.9056
125 C -5.3894 -4.7948 -0.2324
126 H -6.1511 -2.8344 -0.6794
127 C -3.3154 -5.4033 -1.2939
128 H -2.4206 -3.9135 -2.5740
129 C -4.3806 -5.7430 -0.4463
130 H -6.2470 -5.0392 0.4084
131 H -2.5156 -6.1317 -1.4836
132 H -4.4294 -6.7317 0.0261
133 H -2.8822 0.4826 -3.0020
134 H -2.5167 -0.0446 -4.6850

TS to-Dprime(S)
Energy (FREE) = -2.363643646869367E6 kcal.mol^{-1}
Atom X Y Z
1 C 3.1643 -1.1431 4.0682
2 C 2.1710 -0.5277 3.2819
3 C 1.9319 0.8560 3.4738
4 C 2.6881 1.5695 4.4202
5 C 3.6499 0.9365 5.2052
6 C 3.8888 -0.4285 5.0256
7 P 1.2728 -1.5576 2.0200
8 C -0.2011 -2.1170 2.9876
9 C -0.4690 -1.6867 4.3002
10 C -1.6158 -2.1311 4.9720
11 C -2.4948 -3.0239 4.3526
12 C -2.2195 -3.4810 3.0568
13 C -1.0851 -3.0275 2.3793
14 C 0.9328 1.6962 2.7326
15 O 1.2308 2.8262 2.3317

16 Pd 1.0630 -0.8838 -0.2611
17 P 2.8112 0.5586 -0.7927
18 C 3.7490 1.5445 0.4483
19 C 3.7024 2.9463 0.4926
20 C 4.4888 3.6502 1.4128
21 C 5.3289 2.9629 2.2937
22 C 5.3699 1.5622 2.2642
23 C 4.5791 0.8576 1.3556
24 C -0.1434 -2.1329 -1.6394
25 C 0.5655 -1.1205 -2.3496
26 C -1.3167 -1.8311 -0.9364
27 C -1.9716 -3.7987 -3.7467
28 C -2.9405 -3.6273 -2.5681
29 O -3.2434 -4.6213 -1.8881
30 C -2.6779 -3.6268 -5.0559
31 C -3.0971 -2.4036 -5.4732
32 C -3.0424 -1.2226 -4.6436
33 C -3.1790 -1.1921 -3.2744
34 C -3.4078 -2.2662 -2.3269
35 C 2.2807 -3.1043 1.9633
36 C 2.0012 -4.2316 2.7563
37 C 2.8034 -5.3754 2.6648
38 C 3.8949 -5.4065 1.7894
39 C 4.1842 -4.2854 1.0018
40 C 3.3794 -3.1455 1.0855
41 C 2.3454 1.8599 -2.0369
42 C 1.0467 2.4156 -2.1365
43 C 0.7948 3.4142 -3.0984
44 C 1.7994 3.8859 -3.9412
45 C 3.0805 3.3334 -3.8539
46 C 3.3399 2.3323 -2.9168
47 C -0.2071 1.9915 -1.4041
48 O -1.1551 1.5476 -2.0578
49 C 4.2129 -0.3768 -1.5699
50 C 4.0016 -1.6770 -2.0622
51 C 5.0493 -2.4074 -2.6349
52 C 6.3293 -1.8506 -2.7194
53 C 6.5585 -0.5644 -2.2154
54 C 5.5132 0.1637 -1.6392
55 N -0.3042 2.2155 -0.0749
56 C -1.5873 1.9791 0.5851
57 C -1.4066 2.0635 2.1201
58 C -2.6882 1.7367 2.8580
59 C -3.2389 0.4450 2.8093
60 C -4.4136 0.1502 3.5098
61 C -5.0600 1.1458 4.2521
62 C -4.5228 2.4376 4.2958
63 C -3.3402 2.7283 3.6068
64 C -2.6582 2.9567 0.1297
65 C -3.9399 2.4977 -0.2048
66 C -4.9442 3.4000 -0.5714

67 C -4.6749 4.7739 -0.6088
68 C -3.3951 5.2385 -0.2803
69 C -2.3923 4.3341 0.0880
70 N -0.3223 1.2018 2.5887
71 H -1.1046 3.0981 2.3603
72 H -1.9147 0.9560 0.3294
73 H -1.3887 4.7029 0.3367
74 H -3.1752 6.3126 -0.3115
75 H -5.4602 5.4821 -0.8990
76 H -5.9410 3.0259 -0.8357
77 H -4.1517 1.4208 -0.1847
78 H -2.7531 -0.3473 2.2225
79 H -4.8261 -0.8639 3.4671
80 H -5.9832 0.9142 4.7964
81 H -5.0233 3.2236 4.8738
82 H -2.9172 3.7397 3.6461
83 H 0.4490 2.6634 0.4580
84 H -0.5795 0.3256 3.0440
85 H 4.3461 1.9053 -2.8788
86 H 3.8823 3.6740 -4.5192
87 H 1.5776 4.6712 -4.6729
88 H -0.2211 3.8172 -3.1791
89 H 3.3822 -2.2070 3.9314
90 H 4.6472 -0.9449 5.6256
91 H 4.2192 1.5105 5.9453
92 H 2.5021 2.6431 4.5297
93 H 1.3833 -1.4104 -3.0211
94 H 0.2252 -3.1693 -1.6366
95 H 0.0160 -0.2162 -2.6419
96 H -1.7786 -2.5530 -0.2610
97 H -1.7155 -0.8107 -0.9475
98 H 0.2189 -1.0050 4.8146
99 H -1.8149 -1.7767 5.9902
100 H -3.3919 -3.3697 4.8794
101 H -2.8939 -4.1921 2.5661
102 H -0.8677 -3.4069 1.3751
103 H 1.1569 -4.2221 3.4541
104 H 2.5714 -6.2482 3.2865
105 H 4.5191 -6.3051 1.7198
106 H 5.0356 -4.2973 0.3107
107 H 3.6057 -2.2769 0.4573
108 H 3.0553 3.5002 -0.1963
109 H 4.4444 4.7456 1.4341
110 H 5.9501 3.5163 3.0081
111 H 6.0198 1.0140 2.9558
112 H 4.6281 -0.2378 1.3370
113 H 5.7209 1.1582 -1.2298
114 H 7.5612 -0.1237 -2.2632
115 H 7.1517 -2.4224 -3.1650
116 H 4.8608 -3.4213 -3.0068
117 H 3.0143 -2.1400 -1.9678

118 H -3.1934 -0.1835 -2.8332
119 H -2.9862 -0.2499 -5.1544
120 H -3.4980 -2.2921 -6.4921
121 H -2.8333 -4.5096 -5.6920
122 C -4.3531 -1.9942 -1.2244
123 C -4.4241 -2.7911 -0.0501
124 C -5.2664 -0.9112 -1.3053
125 C -5.3506 -2.5229 0.9605
126 H -3.7538 -3.6468 0.0505
127 C -6.1880 -0.6409 -0.2888
128 H -5.2773 -0.2843 -2.2037
129 C -6.2435 -1.4483 0.8537
130 H -5.3734 -3.1663 1.8498
131 H -6.8810 0.2028 -0.4019
132 H -6.9730 -1.2473 1.6474
133 H -1.1799 -3.0231 -3.6643
134 H -1.5156 -4.7988 -3.6564

Ts-to-D-inner(S)
Energy (FREE) = -2.3632608507405026E6 kcal.mol^{-1}
Atom X Y Z
1 C -1.6747 -2.4615 -2.9753
2 C -1.6701 -2.7442 -1.5940
3 C -2.6708 -3.6033 -1.0934
4 C -3.6593 -4.1269 -1.9356
5 C -3.6623 -3.8185 -3.2983
6 C -2.6588 -2.9910 -3.8149
7 P -0.3025 -2.0778 -0.5112
8 C 1.1249 -3.0048 -1.2426
9 C 2.4262 -2.4918 -1.0949
10 C 3.5237 -3.1782 -1.6266
11 C 3.3359 -4.3785 -2.3205
12 C 2.0439 -4.8931 -2.4794
13 C 0.9454 -4.2128 -1.9443
14 Pd 0.5019 0.1212 -0.4201
15 P 1.3161 0.5530 1.8945
16 C 3.0984 1.0764 1.8932
17 C 3.3855 2.3775 1.4367
18 C 4.6987 2.8505 1.4107
19 C 5.7530 2.0301 1.8350
20 C 5.4784 0.7344 2.2813
21 C 4.1594 0.2581 2.3097
22 O 2.3285 1.2126 -1.0272
23 C 2.5566 2.2550 -1.7325
24 C 1.6384 3.3673 -1.5548
25 C 1.5419 4.6022 -2.1880
26 C 2.3475 5.1743 -3.1838
27 C 3.5154 4.5953 -3.7273
28 C 4.0928 3.3602 -3.4498
29 C 3.7009 2.2665 -2.6164
30 C 4.6066 1.0933 -2.6411

31 C 5.1436 0.6253 -3.8629
32 C 6.0749 -0.4164 -3.9012
33 C 6.5146 -1.0157 -2.7136
34 C 5.9917 -0.5678 -1.4927
35 C 5.0434 0.4580 -1.4556
36 C -0.2551 0.4986 -2.3663
37 C 0.6583 0.1547 -3.4550
38 C 1.3116 1.0397 -4.2454
39 C 1.2862 -0.7505 3.1966
40 C 0.7180 -0.5725 4.4708
41 C 0.7958 -1.5889 5.4297
42 C 1.4432 -2.7926 5.1334
43 C 1.9786 -2.9927 3.8561
44 C 1.8827 -1.9884 2.8890
45 C 0.6275 2.0255 2.7958
46 C -0.5598 2.6877 2.4337
47 C -1.0311 3.7646 3.2089
48 C -0.3242 4.2038 4.3280
49 C 0.8669 3.5618 4.6869
50 C 1.3313 2.4872 3.9284
51 C -1.3646 2.3893 1.1946
52 N -2.4914 1.6854 1.4186
53 C -3.4256 1.3706 0.3511
54 C -4.4584 2.4655 0.1513
55 C -4.7521 2.9410 -1.1349
56 C -5.7303 3.9243 -1.3233
57 C -6.4253 4.4423 -0.2241
58 C -6.1339 3.9757 1.0644
59 C -5.1548 2.9942 1.2504
60 O -1.0616 2.8562 0.0954
61 C -4.0835 0.0103 0.6963
62 N -3.0669 -1.0345 0.8246
63 C -2.4377 -1.3163 1.9937
64 C -1.5975 -2.5652 2.0368
65 C -0.6800 -3.0160 1.0520
66 C -0.0257 -4.2435 1.2709
67 C -0.2767 -5.0203 2.4033
68 C -1.1688 -4.5644 3.3762
69 C -1.8045 -3.3376 3.1948
70 C -5.1275 -0.3912 -0.3237
71 C -4.7737 -0.6022 -1.6670
72 C -5.7467 -0.9484 -2.6100
73 C -7.0854 -1.0840 -2.2224
74 C -7.4454 -0.8752 -0.8861
75 C -6.4704 -0.5319 0.0574
76 O -2.6033 -0.6379 3.0142
77 H -4.5660 0.1132 1.6856
78 H -2.8425 1.2531 -0.5802
79 H -4.9222 2.6401 2.2630
80 H -6.6709 4.3812 1.9304
81 H -7.1905 5.2141 -0.3700

82 H -5.9465 4.2911 -2.3338
83 H -4.2053 2.5376 -1.9964
84 H -3.7288 -0.4945 -1.9884
85 H -5.4553 -1.1112 -3.6541
86 H -7.8484 -1.3518 -2.9630
87 H -8.4915 -0.9794 -0.5745
88 H -6.7549 -0.3650 1.1036
89 H -2.6179 1.2150 2.3209
90 H -2.9927 -1.6907 0.0474
91 H 2.2604 1.9878 4.2254
92 H 1.4360 3.8956 5.5623
93 H -0.7002 5.0482 4.9173
94 H -1.9601 4.2665 2.9123
95 H 0.7049 -4.6058 0.5419
96 H 0.2444 -5.9767 2.5282
97 H -1.3641 -5.1557 4.2781
98 H -2.4973 -2.9561 3.9522
99 H -2.6801 -3.8962 -0.0377
100 H -4.4243 -4.7905 -1.5158
101 H -4.4356 -4.2293 -3.9577
102 H -2.6321 -2.7571 -4.8858
103 H -0.8812 -1.8589 -3.4219
104 H -0.0578 -4.6341 -2.0711
105 H 1.8859 -5.8315 -3.0241
106 H 4.1966 -4.9095 -2.7441
107 H 4.5283 -2.7572 -1.5107
108 H 2.5794 -1.5346 -0.5776
109 H 0.2072 0.3611 4.7248
110 H 0.3480 -1.4320 6.4184
111 H 1.5131 -3.5830 5.8903
112 H 2.4611 -3.9437 3.6025
113 H 2.2815 -2.1707 1.8829
114 H 3.9716 -0.7538 2.6825
115 H 6.2927 0.0825 2.6198
116 H 6.7845 2.4013 1.8150
117 H 4.8992 3.8672 1.0516
118 H 2.5738 3.0329 1.1008
119 H 0.8704 3.1474 -0.8039
120 H 0.6999 5.2228 -1.8458
121 H 2.0686 6.1729 -3.5423
122 H 4.0629 5.2113 -4.4543
123 H 5.0375 3.1869 -3.9822
124 H -1.2236 -0.0284 -2.3932
125 H -0.4135 1.5880 -2.2635
126 H 0.8503 -0.9165 -3.6338
127 H 1.9932 0.7011 -5.0371
128 H 1.1668 2.1225 -4.1328
129 H 4.6546 0.8005 -0.4937
130 H 6.3273 -1.0223 -0.5515
131 H 7.2534 -1.8258 -2.7398
132 H 6.4608 -0.7621 -4.8684

133 H 4.8081 1.0793 -4.8034

Energy (FREE) = -1.9286110629079903E6 kcal.mol^{-1}
Atom X Y Z
1 C -1.2906 3.9711 1.6601
2 C -0.5144 3.1615 0.8144
3 C 0.8861 3.3657 0.7373
4 C 1.4601 4.3781 1.5241
5 C 0.6757 5.1846 2.3576
6 C -0.7041 4.9819 2.4282
7 C -1.2189 2.1768 -0.0790
8 O -0.9458 2.0802 -1.2751
9 P 1.9435 2.2073 -0.2480
10 Pd 1.5818 -0.0085 0.1393
11 P 1.8542 -2.2420 0.5023
12 C 0.8862 -3.3140 -0.6567
13 C -0.4881 -3.0529 -0.8863
14 C -1.1836 -3.7895 -1.8594
15 C -0.5420 -4.7825 -2.6065
16 C 0.8125 -5.0406 -2.3857
17 C 1.5176 -4.3069 -1.4238
18 C -1.2603 -2.0880 -0.0284
19 N -2.1623 -1.3125 -0.6925
20 C -3.3120 -0.6967 -0.0402
21 C -3.3077 0.8445 -0.2267
22 N -2.2181 1.4677 0.5159
23 O -1.1230 -2.0618 1.1944
24 C -4.5921 -1.3421 -0.5455
25 C -4.9725 -1.2238 -1.8938
26 C -6.1406 -1.8357 -2.3598
27 C -6.9459 -2.5739 -1.4829
28 C -6.5747 -2.6968 -0.1394
29 C -5.4040 -2.0848 0.3246
30 C -4.6261 1.4828 0.1781
31 C -5.1121 1.3542 1.4912
32 C -6.3155 1.9597 1.8667
33 C -7.0513 2.7009 0.9331
34 C -6.5751 2.8334 -0.3761
35 C -5.3689 2.2284 -0.7493
36 H -3.1961 -0.9147 1.0340
37 H -3.1092 1.0635 -1.2888
38 H -4.5498 0.7712 2.2329
39 H -6.6826 1.8494 2.8941
40 H -7.9956 3.1744 1.2271
41 H -7.1445 3.4121 -1.1132
42 H -4.9989 2.3330 -1.7769
43 H -4.3551 -0.6443 -2.5933
44 H -6.4252 -1.7331 -3.4138
45 H -7.8622 -3.0528 -1.8481
46 H -7.1988 -3.2734 0.5539
47 H -5.1165 -2.1820 1.3790

48 H -2.3468 1.5739 1.5206
49 H -2.1818 -1.3669 -1.7093
50 H 2.5427 4.5416 1.4920
51 H 1.1515 5.9733 2.9521
52 H -1.3278 5.6096 3.0750
53 H -2.3771 3.8267 1.6978
54 H 2.5830 -4.5121 -1.2744
55 H 1.3303 -5.8162 -2.9621
56 H -1.1037 -5.3531 -3.3549
57 H -2.2528 -3.6028 -2.0167
58 C 1.9199 2.8787 -1.9587
59 C 3.6450 2.6599 0.3152
60 C 1.6092 -2.9866 2.1646
61 C 3.5967 -2.7312 0.1249
62 C 4.1365 2.0350 1.4774
63 C 5.4109 2.3388 1.9644
64 C 6.2204 3.2595 1.2852
65 C 5.7444 3.8774 0.1236
66 C 4.4618 3.5843 -0.3580
67 H 3.5083 1.2956 1.9938
68 H 5.7789 1.8456 2.8721
69 H 7.2252 3.4899 1.6591
70 H 6.3741 4.5959 -0.4149
71 H 4.0998 4.0809 -1.2656
72 C 2.4050 2.0549 -2.9886
73 C 2.4510 2.5223 -4.3066
74 C 2.0027 3.8143 -4.6082
75 C 1.5120 4.6368 -3.5863
76 C 1.4727 4.1735 -2.2663
77 H 2.7420 1.0361 -2.7531
78 H 2.8311 1.8707 -5.1025
79 H 2.0310 4.1795 -5.6418
80 H 1.1556 5.6478 -3.8179
81 H 1.0844 4.8235 -1.4729
82 C 2.0271 -2.2393 3.2789
83 C 1.9045 -2.7647 4.5698
84 C 1.3535 -4.0383 4.7589
85 C 0.9287 -4.7838 3.6520
86 C 1.0575 -4.2626 2.3596
87 H 2.4461 -1.2346 3.1309
88 H 2.2336 -2.1729 5.4323
89 H 1.2505 -4.4486 5.7706
90 H 0.4921 -5.7797 3.7949
91 H 0.7191 -4.8520 1.4989
92 C 4.2587 -2.0506 -0.9145
93 C 5.5725 -2.3817 -1.2589
94 C 6.2494 -3.3878 -0.5566
95 C 5.6028 -4.0629 0.4847
96 C 4.2820 -3.7409 0.8223
97 H 3.7322 -1.2453 -1.4462
98 H 6.0745 -1.8438 -2.0720

99 H 7.2841 -3.6406 -0.8177
100 H 6.1281 -4.8486 1.0410
101 H 3.7858 -4.2819 1.6365

Dprime(S)
Energy (FREE) = -2.3636644914526218E6 kcal.mol^{-1}
Atom X Y Z
1 C -7.4836 3.5083 -4.0206
2 C -7.4104 4.7715 -3.4058
3 C -8.4680 5.6792 -3.6031
4 C -9.5685 5.3277 -4.3929
5 C -9.6316 4.0665 -4.9970
6 C -8.5859 3.1559 -4.8069
7 P -5.9158 5.1792 -2.3819
8 C -6.0650 7.0306 -2.4015
9 C -6.6266 7.7708 -1.3436
10 C -6.7967 9.1564 -1.4575
11 C -6.4276 9.8162 -2.6339
12 C -5.8706 9.0873 -3.6925
13 C -5.6819 7.7065 -3.5741
14 C -6.4757 4.8097 -0.6438
15 C -5.6136 4.8655 0.4865
16 C -6.1063 4.4734 1.7461
17 C -7.4207 4.0438 1.9214
18 C -8.2678 3.9772 0.8130
19 C -7.7933 4.3516 -0.4467
20 C -4.1511 5.2488 0.5232
21 O -3.3832 4.6654 1.2873
22 N -3.7438 6.2934 -0.2542
23 C -2.4431 6.9356 -0.0670
24 C -1.4149 6.5805 -1.1950
25 C -0.6676 5.2878 -0.9177
26 C -1.2601 4.0296 -1.0954
27 C -0.5615 2.8621 -0.7722
28 C 0.7440 2.9357 -0.2710
29 C 1.3486 4.1863 -0.1000
30 C 0.6443 5.3531 -0.4204
31 C -2.5951 8.4391 0.0673
32 C -2.0343 9.0912 1.1778
33 C -2.1126 10.4824 1.3062
34 C -2.7554 11.2426 0.3222
35 C -3.3201 10.6016 -0.7868
36 C -3.2408 9.2103 -0.9153
37 Pd -4.1468 3.8469 -3.1400
38 P -2.7745 4.6536 -4.8911
39 C -0.9346 4.4595 -4.8519
40 C -0.0424 5.3870 -5.4204
41 C 1.3385 5.1598 -5.3806
42 C 1.8469 3.9994 -4.7864
43 C 0.9655 3.0632 -4.2330
44 C -0.4130 3.2952 -4.2614

45 C -3.5819 1.7475 -3.1241
46 C -4.6228 1.8984 -2.1916
47 C -4.3942 1.8742 -0.7039
48 C -2.9518 6.4102 -5.4673
49 C -2.4176 7.5129 -4.7513
50 C -2.5876 8.8146 -5.2541
51 C -3.3250 9.0574 -6.4133
52 C -3.8810 7.9783 -7.1061
53 C -3.6792 6.6742 -6.6441
54 C -1.6020 7.4503 -3.4830
55 N -2.0569 6.6317 -2.4960
56 C -3.2186 3.7063 -6.4135
57 C -2.3068 3.4735 -7.4576
58 C -2.7009 2.7530 -8.5911
59 C -4.0091 2.2649 -8.6982
60 C -4.9245 2.4960 -7.6641
61 C -4.5283 3.2065 -6.5260
62 O -0.6181 8.1767 -3.3467
63 H -0.6702 7.3911 -1.2071
64 H -2.0369 6.5328 0.8741
65 H -3.6898 8.7310 -1.7945
66 H -3.8290 11.1865 -1.5622
67 H -2.8186 12.3328 0.4206
68 H -1.6711 10.9737 2.1816
69 H -1.5285 8.4993 1.9510
70 H -2.2789 3.9606 -1.5124
71 H -1.0365 1.8833 -0.9162
72 H 1.2906 2.0184 -0.0209
73 H 2.3741 4.2563 0.2826
74 H 1.1207 6.3324 -0.2832
75 H -4.4557 6.8398 -0.7407
76 H -2.8944 6.0534 -2.6512
77 H -8.4705 4.2755 -1.3028
78 H -9.3016 3.6284 0.9198
79 H -7.7759 3.7534 2.9168
80 H -5.4200 4.5090 2.5981
81 H -4.0952 5.8421 -7.2212
82 H -4.4592 8.1436 -8.0228
83 H -3.4619 10.0835 -6.7736
84 H -2.1326 9.6441 -4.7021
85 H -5.1268 2.5252 -0.1990
86 H -5.6423 1.6280 -2.5001
87 H -3.3908 2.2669 -0.4605
88 H -3.7850 1.3474 -4.1284
89 H -2.5565 1.5976 -2.7566
90 H -0.4167 6.2965 -5.9020
91 H 2.0194 5.8972 -5.8222
92 H 2.9289 3.8247 -4.7550
93 H 1.3517 2.1506 -3.7638
94 H -1.0947 2.5658 -3.8140
95 H -1.2808 3.8536 -7.3882

96 H -1.9791 2.5727 -9.3967
97 H -4.3140 1.7002 -9.5873
98 H -5.9505 2.1162 -7.7385
99 H -5.2393 3.3793 -5.7068
100 H -6.9467 7.2665 -0.4241
101 H -7.2301 9.7184 -0.6218
102 H -6.5689 10.8996 -2.7256
103 H -5.5756 9.5951 -4.6179
104 H -5.2483 7.1434 -4.4094
105 H -8.4404 6.6699 -3.1363
106 H -10.3827 6.0481 -4.5363
107 H -10.4938 3.7952 -5.6177
108 H -8.6222 2.1660 -5.2770
109 H -6.6615 2.7973 -3.8834
110 C -4.5057 0.4843 0.0015
111 C -5.9600 -0.0284 -0.0596
112 C -6.2410 -1.4666 0.3626
113 C -5.3484 -2.1845 1.3262
114 C -4.5677 -1.6310 2.2818
115 C -4.3289 -0.2087 2.4645
116 C -4.3057 0.7296 1.4881
117 O -6.8778 0.6919 -0.4074
118 H -6.3017 -2.0540 -0.5760
119 H -7.2809 -1.4699 0.7477
120 H -5.4174 -3.2809 1.2892
121 H -4.0833 -2.3056 3.0028
122 H -4.0962 0.1223 3.4873
123 H -4.1442 1.7795 1.7720
124 C -3.5121 -0.5021 -0.6072
125 C -2.2071 -0.6235 -0.1016
126 C -1.2645 -1.4424 -0.7323
127 C -1.6114 -2.1616 -1.8831
128 C -2.9052 -2.0427 -2.4021
129 C -3.8410 -1.2123 -1.7740
130 H -1.9258 -0.0697 0.8016
131 H -0.2515 -1.5211 -0.3188
132 H -0.8751 -2.8090 -2.3744
133 H -3.1891 -2.5921 -3.3079
134 H -4.8412 -1.1089 -2.2121

E
Energy (FREE) =- 434649.8642255796 kcal.mol^{-1}
Atom X Y Z
1 C -0.5611 0.5748 1.4724
2 C 0.6263 0.9045 2.1752
3 C 1.7104 1.6838 1.7247
4 C -0.9798 0.9806 0.2300
5 C 1.9151 2.2904 0.4969
6 C -0.3975 1.9157 -0.8003
7 C 1.1040 2.3253 -0.7169
8 H 2.8724 2.8068 0.3487

9 H -1.2522 -0.0900 2.0083
10 H 0.7162 0.4940 3.1882
11 H 2.5289 1.8082 2.4468
12 H -1.9596 0.5938 -0.0861
13 O 1.6191 2.7757 -1.7393
14 C -1.1573 3.2580 -0.6739
15 C -1.7540 3.8990 -1.7718
16 C -1.2162 3.8876 0.5840
17 C -2.3981 5.1327 -1.6120
18 H -1.7271 3.4457 -2.7666
19 C -1.8538 5.1212 0.7417
20 H -0.7580 3.4038 1.4548
21 C -2.4506 5.7495 -0.3582
22 H -2.8622 5.6135 -2.4816
23 H -1.8858 5.5923 1.7314
24 H -2.9547 6.7157 -0.2376
25 C -0.6178 1.2598 -2.1950
26 H -1.7102 1.1486 -2.3426
27 H -0.2310 1.9361 -2.9723
28 C 0.0476 -0.0809 -2.3129
29 H -0.3304 -0.8745 -1.6485
30 C 1.0528 -0.3604 -3.1542
31 H 1.4628 0.4075 -3.8267
32 H 1.4998 -1.3629 -3.1994

TS to-D(R)
Energy (FREE) = -2.3632697842238666E6 kcal.mol^{-1}
Atom X Y Z
1 C 2.7549 -1.4548 3.7295
2 C 1.8571 -0.7163 2.9340
3 C 1.6509 0.6483 3.2537
4 C 2.3401 1.2208 4.3384
5 C 3.2042 0.4660 5.1289
6 C 3.4146 -0.8805 4.8186
7 P 1.0560 -1.5443 1.4742
8 C -0.4877 -2.2168 2.2407
9 C -0.9259 -1.8689 3.5314
10 C -2.1377 -2.3681 4.0295
11 C -2.9137 -3.2357 3.2560
12 C -2.4712 -3.6110 1.9797
13 C -1.2729 -3.1014 1.4747
14 C 0.7655 1.6191 2.5239
15 O 1.1480 2.7737 2.3070
16 Pd 0.9262 -0.5535 -0.6749
17 P 2.7420 0.8554 -1.0465
18 C 3.6700 1.6536 0.3325
19 C 3.7406 3.0449 0.5040
20 C 4.4931 3.5919 1.5509
21 C 5.1877 2.7571 2.4315
22 C 5.1140 1.3664 2.2727
23 C 4.3515 0.8189 1.2397

24 C -0.5883 -1.6132 -1.8580
25 C 0.1738 -0.7557 -2.6998
26 C -1.8200 -1.1982 -1.2756
27 C -3.6067 -1.9546 -2.4169
28 C -4.4583 -1.1662 -1.4767
29 O -4.6901 -1.6116 -0.3347
30 C -3.4670 -3.4279 -2.1502
31 C -2.3952 -4.1545 -2.7167
32 C -2.2585 -5.5332 -2.5294
33 C -3.2035 -6.2413 -1.7787
34 C -4.2879 -5.5463 -1.2315
35 C -4.4171 -4.1646 -1.4079
36 C -3.3363 -1.5752 -3.7749
37 C -3.4843 -0.3763 -4.4686
38 C -4.1071 0.8140 -4.0653
39 C -4.8083 1.0152 -2.8612
40 C -4.9892 0.1573 -1.7892
41 C 2.0583 -3.0826 1.2672
42 C 1.7916 -4.2732 1.9678
43 C 2.5850 -5.4077 1.7622
44 C 3.6556 -5.3679 0.8608
45 C 3.9304 -4.1864 0.1618
46 C 3.1338 -3.0541 0.3606
47 C 2.4271 2.3058 -2.1660
48 C 1.1651 2.9345 -2.2774
49 C 1.0122 4.0269 -3.1551
50 C 2.0834 4.5200 -3.8978
51 C 3.3331 3.9010 -3.7925
52 C 3.4926 2.8066 -2.9413
53 C -0.1367 2.5280 -1.6255
54 O -1.0998 2.2428 -2.3419
55 C 4.1305 -0.0439 -1.8897
56 C 3.8331 -1.1699 -2.6775
57 C 4.8488 -1.8813 -3.3260
58 C 6.1826 -1.4808 -3.1904
59 C 6.4931 -0.3669 -2.4012
60 C 5.4775 0.3450 -1.7540
61 N -0.2528 2.6200 -0.2782
62 C -1.5800 2.4801 0.3119
63 C -1.4943 2.2271 1.8381
64 C -2.8437 1.8319 2.4055
65 C -3.5158 0.6912 1.9336
66 C -4.7579 0.3272 2.4639
67 C -5.3433 1.1002 3.4742
68 C -4.6803 2.2375 3.9506
69 C -3.4367 2.5994 3.4194
70 C -2.4484 3.7030 0.0525
71 C -3.7900 3.5593 -0.3275
72 C -4.6007 4.6841 -0.5180
73 C -4.0756 5.9682 -0.3284
74 C -2.7363 6.1199 0.0525

75 C -1.9283 4.9933 0.2427
76 N -0.4938 1.2246 2.2027
77 H -1.1641 3.1699 2.3086
78 H -2.0536 1.6080 -0.1665
79 H -0.8778 5.1183 0.5349
80 H -2.3170 7.1222 0.2018
81 H -4.7086 6.8508 -0.4800
82 H -5.6471 4.5560 -0.8206
83 H -4.1994 2.5533 -0.4780
84 H -3.0832 0.0737 1.1357
85 H -5.2645 -0.5583 2.0641
86 H -6.3192 0.8186 3.8879
87 H -5.1336 2.8495 4.7395
88 H -2.9210 3.4935 3.7911
89 H 0.5177 2.9412 0.3153
90 H -0.8321 0.3053 2.4872
91 H 4.4751 2.3272 -2.8839
92 H 4.1864 4.2633 -4.3775
93 H 1.9391 5.3775 -4.5653
94 H 0.0225 4.4880 -3.2482
95 H 2.9493 -2.5055 3.4920
96 H 4.0992 -1.4905 5.4196
97 H 3.7212 0.9314 5.9761
98 H 2.1831 2.2837 4.5495
99 H 0.9049 -1.1951 -3.3907
100 H -0.3143 -2.6756 -1.7829
101 H -0.2702 0.1966 -3.0211
102 H -2.2305 -1.7168 -0.4036
103 H -2.0948 -0.1413 -1.3914
104 H -0.3189 -1.2128 4.1672
105 H -2.4680 -2.0781 5.0340
106 H -3.8609 -3.6256 3.6468
107 H -3.0669 -4.2977 1.3663
108 H -0.9341 -3.4080 0.4774
109 H 0.9616 -4.3189 2.6818
110 H 2.3636 -6.3294 2.3133
111 H 4.2732 -6.2596 0.7006
112 H 4.7630 -4.1444 -0.5507
113 H 3.3411 -2.1374 -0.2040
114 H 3.2103 3.7134 -0.1834
115 H 4.5388 4.6809 1.6720
116 H 5.7837 3.1876 3.2451
117 H 5.6486 0.7028 2.9623
118 H 4.3026 -0.2712 1.1257
119 H 5.7424 1.2093 -1.1351
120 H 7.5357 -0.0479 -2.2841
121 H 6.9809 -2.0401 -3.6926
122 H 4.5936 -2.7585 -3.9323
123 H 2.7947 -1.5049 -2.7644
124 H -5.5992 0.5346 -0.9575
125 H -5.2915 1.9972 -2.7566

126 H -4.0948 1.6499 -4.7752
127 H -3.0828 -0.3821 -5.4912
128 H -2.8774 -2.3680 -4.3771
129 H -5.2720 -3.6517 -0.9653
130 H -5.0518 -6.0851 -0.6570
131 H -3.1006 -7.3229 -1.6296
132 H -1.4046 -6.0556 -2.9781
133 H -1.6433 -3.6337 -3.3175

Dprime(R)
Energy (FREE) = -2.3636628601901755E6 $kcal.mol^{-1}$
Atom X Y Z

1 C -0.7896 -2.1750 3.3381
2 C -0.0413 -2.8434 2.3533
3 C 0.5011 -4.1056 2.6611
4 C 0.2927 -4.6814 3.9190
5 C -0.4579 -4.0072 4.8901
6 C -1.0001 -2.7514 4.5958
7 P 0.2090 -2.0219 0.7058
8 C 1.7659 -2.9062 0.2118
9 C 1.7787 -4.0159 -0.6546
10 C 2.9709 -4.7064 -0.9066
11 C 4.1584 -4.3102 -0.2837
12 C 4.1544 -3.2078 0.5805
13 C 2.9690 -2.5052 0.8202
14 C -1.0442 -2.8616 -0.3871
15 C -1.2809 -2.4934 -1.7412
16 C -2.3447 -3.0956 -2.4405
17 C -3.1672 -4.0531 -1.8501
18 C -2.9457 -4.4102 -0.5181
19 C -1.9020 -3.8160 0.1959
20 C -0.5700 -1.4396 -2.5606
21 O -1.1998 -0.7800 -3.3852
22 N 0.7774 -1.2958 -2.3989
23 C 1.5811 -0.5091 -3.3338
24 C 2.0391 0.8637 -2.7363
25 C 0.9996 1.9576 -2.9081
26 C -0.1914 1.9830 -2.1670
27 C -1.1444 2.9804 -2.3914
28 C -0.9236 3.9687 -3.3584
29 C 0.2649 3.9577 -4.0966
30 C 1.2180 2.9573 -3.8703
31 C 2.7715 -1.3052 -3.8340
32 C 2.9688 -1.4529 -5.2169
33 C 4.0826 -2.1403 -5.7121
34 C 5.0170 -2.6914 -4.8277
35 C 4.8275 -2.5520 -3.4476
36 C 3.7140 -1.8640 -2.9530
37 Pd -0.1822 0.2777 0.9351
38 P 1.6245 1.6914 1.5216

39 C 1.9091 3.3110 0.6759
40 C 3.1831 3.8602 0.4447
41 C 3.3148 5.1115 -0.1696
42 C 2.1789 5.8369 -0.5458
43 C 0.9068 5.3048 -0.3036
44 C 0.7742 4.0489 0.2961
45 C -1.8666 1.6118 1.2805
46 C -2.3947 0.3253 1.0680
47 C -3.1637 -0.0611 -0.1693
48 C 3.3392 0.9827 1.6008
49 C 4.1279 0.7354 0.4472
50 C 5.4181 0.1961 0.5900
51 C 5.9223 -0.1642 1.8399
52 C 5.1373 0.0446 2.9775
53 C 3.8719 0.6262 2.8553
54 C 3.7463 1.0433 -0.9793
55 N 2.4912 0.6918 -1.3673
56 C 1.3634 2.2394 3.2676
57 C 1.9031 3.4354 3.7724
58 C 1.6867 3.8000 5.1060
59 C 0.9357 2.9735 5.9514
60 C 0.3974 1.7794 5.4573
61 C 0.6059 1.4191 4.1215
62 O 4.5764 1.5212 -1.7520
63 H 2.9328 1.1713 -3.3002
64 H 0.9212 -0.2830 -4.1858
65 H 3.5872 -1.7669 -1.8674
66 H 5.5511 -2.9827 -2.7452
67 H 5.8905 -3.2308 -5.2130
68 H 4.2184 -2.2476 -6.7950
69 H 2.2393 -1.0219 -5.9140
70 H -0.3727 1.2244 -1.3877
71 H -2.0662 2.9920 -1.7985
72 H -1.6740 4.7499 -3.5297
73 H 0.4548 4.7313 -4.8505
74 H 2.1492 2.9505 -4.4514
75 H 1.2764 -1.9795 -1.8284
76 H 1.8362 0.2822 -0.6868
77 H -1.7605 -4.0994 1.2431
78 H -3.5871 -5.1493 -0.0238
79 H -3.9846 -4.5051 -2.4235
80 H -2.5226 -2.7761 -3.4718
81 H 3.2888 0.8115 3.7632
82 H 5.5138 -0.2262 3.9709
83 H 6.9229 -0.6038 1.9241
84 H 6.0191 0.0513 -0.3142
85 H -2.9966 -1.1230 -0.4172
86 H -2.6331 -0.2755 1.9574
87 H -2.8278 0.5244 -1.0429
88 H -1.7543 2.0097 2.2996
89 H -1.9666 2.3655 0.4856

90 H 4.0864 3.3182 0.7440
91 H 4.3158 5.5214 -0.3495
92 H 2.2849 6.8163 -1.0272
93 H 0.0096 5.8633 -0.5954
94 H -0.2232 3.6314 0.4675
95 H 2.4958 4.0899 3.1225
96 H 2.1089 4.7376 5.4870
97 H 0.7674 3.2629 6.9956
98 H -0.1931 1.1269 6.1114
99 H 0.1771 0.4891 3.7261
100 H 0.8514 -4.3571 -1.1303
101 H 2.9646 -5.5653 -1.5882
102 H 5.0896 -4.8563 -0.4750
103 H 5.0807 -2.8869 1.0707
104 H 2.9746 -1.6459 1.5020
105 H 1.0897 -4.6510 1.9150
106 H 0.7228 -5.6653 4.1410
107 H -0.6158 -4.4595 5.8764
108 H -1.5854 -2.2110 5.3493
109 H -1.2067 -1.1876 3.1132
110 C -4.7247 0.1002 -0.0417
111 C -5.3543 -0.2996 -1.3937
112 C -6.8077 -0.7413 -1.3362
113 C -6.7802 -2.2223 -1.0818
114 C -6.2745 -2.7486 0.0581
115 C -5.7439 -1.9651 1.1642
116 C -5.1845 -0.7296 1.1311
117 O -4.7297 -0.2681 -2.4354
118 H -7.3021 -0.5038 -2.2913
119 H -7.3119 -0.2146 -0.5002
120 H -7.1504 -2.8877 -1.8726
121 H -6.3245 -3.8367 0.2027
122 H -5.8170 -2.4383 2.1550
123 H -4.9049 -0.3029 2.1033
124 C -5.1199 1.5819 0.1173
125 C -4.7194 2.5063 -0.8662
126 C -5.0467 3.8607 -0.7615
127 C -5.7931 4.3223 0.3300
128 C -6.2202 3.4108 1.3002
129 C -5.8939 2.0524 1.1915
130 H -4.1567 2.1565 -1.7382
131 H -4.7187 4.5585 -1.5415
132 H -6.0503 5.3846 0.4162
133 H -6.8187 3.7540 2.1528
134 H -6.2565 1.3598 1.9575

Ts-to-D-prime-inner(S)
Energy (FREE) = -2.363634085819527E6 kcal.mol^{-1}
Atom X Y Z

1 C 3.4069 -0.0298 -2.6703

2 C 4.1253 1.1851 -2.5534
3 C 5.5292 1.1092 -2.7387
4 C 6.1697 -0.0956 -3.0482
5 C 5.4330 -1.2787 -3.1760
6 C 4.0455 -1.2319 -2.9796
7 C 3.4744 2.4857 -2.2774
8 C 4.1710 3.6616 -2.7517
9 C 4.2416 4.9284 -2.2042
10 C 3.5769 5.3427 -0.9970
11 C 2.3782 4.8301 -0.6025
12 C 1.5854 3.9564 -1.5263
13 C 2.2517 2.5868 -1.5585
14 O 1.7464 1.6409 -0.8516
15 Pd 0.4260 -0.0342 -0.5958
16 C -0.6242 0.4105 -2.3772
17 C 0.1678 0.6892 -3.5765
18 C 0.4952 1.9240 -4.0190
19 P 1.1632 0.3364 1.7444
20 C 0.4779 -0.5357 3.2131
21 C -0.4196 0.0811 4.1008
22 C -0.8953 -0.6079 5.2231
23 C -0.4734 -1.9156 5.4777
24 C 0.4142 -2.5424 4.5926
25 C 0.8758 -1.8645 3.4634
26 C 0.8283 2.1165 2.1773
27 C -0.2167 2.9049 1.6383
28 C -0.3500 4.2456 2.0558
29 C 0.5001 4.8071 3.0064
30 C 1.5313 4.0315 3.5422
31 C 1.6903 2.7104 3.1212
32 C -1.2477 2.5462 0.5933
33 O -1.3830 3.2766 -0.3904
34 N -2.1068 1.5162 0.8271
35 C -3.3455 1.4879 0.0386
36 C -3.9911 0.0612 -0.0127
37 C -4.8223 -0.1505 -1.2768
38 C -4.7477 -1.3312 -2.0348
39 C -5.5338 -1.5105 -3.1801
40 C -6.4245 -0.5155 -3.5900
41 C -6.5264 0.6569 -2.8326
42 C -5.7387 0.8353 -1.6915
43 C -4.2872 2.5485 0.5977
44 C -4.5346 3.7331 -0.1139
45 C -5.3576 4.7281 0.4265
46 C -5.9353 4.5532 1.6896
47 C -5.6810 3.3804 2.4122
48 C -4.8587 2.3862 1.8716
49 N -3.0205 -1.0016 0.1785
50 C -2.6785 -1.3776 1.4362
51 O -3.0996 -0.7556 2.4177
52 C -1.8814 -2.6395 1.6316

53 C -0.7780 -3.1094 0.8746
54 C -0.1907 -4.3348 1.2479
55 C -0.6803 -5.0943 2.3121
56 C -1.7664 -4.6270 3.0544
57 C -2.3415 -3.4029 2.7216
58 C 2.9887 0.1758 2.0300
59 C 3.8553 0.3997 0.9440
60 C 5.2397 0.2784 1.1028
61 C 5.7797 -0.0683 2.3469
62 C 4.9252 -0.2890 3.4337
63 C 3.5394 -0.1687 3.2797
64 C 3.8550 -3.4388 -0.2419
65 C 2.6409 -2.7571 -0.1290
66 C 1.5227 -3.1499 -0.8849
67 C 1.6481 -4.2363 -1.7697
68 C 2.8704 -4.9087 -1.8916
69 C 3.9741 -4.5175 -1.1258
70 C -1.1299 -2.9953 -1.9476
71 C -2.1819 -3.8880 -1.6730
72 C -2.9234 -4.4533 -2.7188
73 C -2.6198 -4.1426 -4.0474
74 C -1.5614 -3.2693 -4.3312
75 C -0.8242 -2.7010 -3.2904
76 H -4.6689 -0.0186 0.8562
77 H -3.0566 1.7763 -0.9829
78 H -4.6503 1.4775 2.4513
79 H -6.1244 3.2388 3.4051
80 H -6.5809 5.3320 2.1129
81 H -5.5462 5.6464 -0.1425
82 H -4.0812 3.8697 -1.1024
83 H -4.0863 -2.1511 -1.7421
84 H -5.4444 -2.4444 -3.7483
85 H -7.0401 -0.6540 -4.4865
86 H -7.2293 1.4451 -3.1277
87 H -5.8526 1.7570 -1.1142
88 H -2.1239 1.0571 1.7407
89 H -2.6422 -1.4873 -0.6315
90 H 2.5161 2.1261 3.5391
91 H 2.2220 4.4518 4.2823
92 H 0.3629 5.8493 3.3167
93 H -1.1477 4.8506 1.6114
94 H 0.6716 -4.7138 0.6917
95 H -0.2013 -6.0486 2.5602
96 H -2.1578 -5.2051 3.8993
97 H -3.1776 -3.0084 3.3070
98 H -2.4338 -4.1525 -0.6403
99 H -3.7432 -5.1428 -2.4858
100 H -3.2020 -4.5854 -4.8640
101 H -1.3051 -3.0302 -5.3698
102 H 0.0208 -2.0448 -3.5235
103 H 0.7920 -4.5749 -2.3620

104 H 2.9536 -5.7511 -2.5884
105 H 4.9281 -5.0491 -1.2226
106 H 4.7143 -3.1121 0.3555
107 H 2.5691 -1.9023 0.5496
108 H -0.7467 1.1127 3.9278
109 H -1.5948 -0.1103 5.9052
110 H -0.8372 -2.4503 6.3633
111 H 0.7462 -3.5700 4.7795
112 H 1.5730 -2.3694 2.7845
113 H 2.8908 -0.3416 4.1449
114 H 5.3374 -0.5558 4.4143
115 H 6.8647 -0.1673 2.4711
116 H 5.8950 0.4526 0.2403
117 H 3.4310 0.6687 -0.0298
118 H -1.3784 -0.3783 -2.5299
119 H -1.0865 1.3303 -1.9690
120 H 1.0918 2.0667 -4.9300
121 H 0.1561 2.8234 -3.4881
122 H 0.5182 -0.1719 -4.1671
123 H 4.8396 3.4875 -3.6086
124 H 4.9564 5.6280 -2.6618
125 H 4.0759 6.0957 -0.3679
126 H 1.9692 5.0638 0.3915
127 H 0.5453 3.8420 -1.1862
128 H 1.6072 4.3960 -2.5442
129 H 6.1366 2.0131 -2.6158
130 H 7.2593 -0.1078 -3.1794
131 H 5.9322 -2.2258 -3.4146
132 H 3.4486 -2.1490 -3.0635
133 H 2.3251 -0.0246 -2.5128
134 P -0.0751 -2.2568 -0.6225

13. References

[1] a) F. Neese, *Wiley Interdiship. Rev. Comput. Mol. Sci.*, **2012**, *2*, 73-78. b) F. Neese, *Wiley Interdiship. Rev. Comput. Mol. Sci.*, **2025**, *15*, e70019.

[2] a) J. W. Furness, A. D. Kaplan, J. Ning, J. P. Perdew, J. Sun, *J. Phys. Chem. Lett.* **2020**, *11*, 8208-8215. b) F. Weigend, *Phys. Chem. Chem. Phys.* **2006**, 8, 1057. c) F. Weigend, R. Ahlrichs, *Phys. Chem. Chem. Phys.* **2005**, 7, 3297.

[3] V. Barone, M. Cossi, *J. Phys. Chem. A*, **1998**, *102,* 1995–2001. b) M. Garcia-Rates, F. Neese, *J. Comput. Chem.* **2020** *41* 922-939.

[4] A. Brunetti, S. Kiriakidi, M. Garbini, G. Monda, C. Zanardi, C. Silva Lòpez, G. Bertuzzi, M. Bandini, *ACS Catal.* **2025**, *15*, 3184-3190.

[5] T. Tang, E. Jones, T. Wild, A. Hazra, S. D. Minteer, M. S. Sigman, Investigating Oxidative Addition Mechanisms of Allylic Electrophiles with Low-Valent Ni/Co Catalysts Using Electroanalytical and Data Science Techniques, *J. Am. Chem. Soc.* **2022**, *144*, 20056-20066.

[6] a) C. L. Barløse, J. Faghtmann, M. Kaasik, R. Mastroddi, K. A. Jørgensen, *Org. Lett.* **2024**, *26*, 1539; b) G. Gallorini, S. Kiriakidi, S. Bellini, C. Silva Lòpez, G. Bertuzzi, M. Bandini, *Org. Lett.* **2024***, 26*, 9251-9256.

[7] P. Pracht, S. Grimme, C. Bannwarth, F. Bohle, S. Ehlert, G. Feldmann, J. Gorges, M. Müller, T. Neudecker, C. Plett, S. Spicher, P. Steinbach, P. A. Wesołowski, F. Zeller, *J. Chem. Phys.* **2024**, *160*, 114110.

[8] C. Bannwarth, S. Ehlert, S. Grimme, *J. Chem. Theory Comput.* **2019**, *15*, 1652–1671.

[9] S. Ehlert, M. Stahn, S. Spicher, S. Grimme, *J. Chem. Theory Comput.* **2021**, *17*, 4250–4261.

[10] G. Pescitelli, T. Bruhn, *Chirality* **2016**, *28*, 466–474.

[11] Y. Zhao, D. G. Truhlar, *Theor. Chem. Acc.* **2008**, *120*, 215-241.

[12] J.-D. Chai, M. Head-Gordon, *Phys. Chem. Chem. Phys.*, **2008**, *10*, 6615-6620.

[13] T. Yanai, D. Tewand, N. Handy, *Chem. Phys. Lett.* **2004**, *393*, 51-57.

[14] a) D. Pecorari, E. Giuliani, A. Mazzanti, S. Stagni, V. Fiorini, G. Vigarani, F. Zinna, G. Pescitelli, M. Mancinelli *J. Org. Chem.* **2023**, *88*, 871-881; b) M. Chiarucci, A. Mazzanti, P. Righi, G. Bencivenni, M. Mancinelli. *Eur. J. Org. Chem.* **2021**, 2594-2603; c) M. Mancinelli, S. Perticarari, L. Prati, A. Mazzanti. *J.Org. Chem.* **2017**, *82*, 6874-6885; d) M. Meazza, M. E. Light, A. Mazzanti, R. Rios. *Chem. Sci.* **2016**, *7*, 984; e) P. Gunasekaran, S. Perumal, J. Carlos Menéndez, M. Mancinelli, S. Ranieri, A. Mazzanti, *J. Org. Chem.* **2014**, *79,* 11039–11050; f) L. Caruana, M. Fochi, M. Comes Franchini, S. Ranieri, A. Mazzanti, L. Bernardi, *Chem. Commun.* **2014**, *50*, 445-447; g) M. Ambrogi, A. Ciogli, M. Mancinelli, S. Ranieri, A. Mazzanti, *J. Org. Chem.* **2013**, *78*, 3709-3719.

[15] P. J. Stephens, D. M. McCann, F. J. Devlin, J. R. Cheeseman, M. J. Frisch, *J. Am. Chem. Soc.* **2004**, *126*, 7514-7521.

[16] The preparation of this complex followed the procedure reported in: C. Amatore, A. Jutand, G. Meyer, L. Moitter, *Chem. Eur. J.* **1999**, *5*, 466-473. The purification step, instead of the reported crystallization, was carried out by trituration in benzene, collecting the resulting insoluble white solid as the desired product. While indefinitely stable in air as a solid, when in solution (DCM or ACN) this complex undergoes degradation, even under Ar atmosphere. NMR and voltametric analyses had to be performed right after preparing a fresh solution.

[17] E. R. Johnson, S. Keinan, P. Mori-Sánchez, J. Contreras-García, A. J. Cohen, W. Yang, *J. Am. Chem. Soc.* **2010**, *132*, 6498-6506.

[18] T. Lu, F. Chen, *J. Comput. Chem.* **2012**, *33*, 580-592.

[19] W. Humphrey, A. Dalke, K. Schulten, *J. Mol. Graph.* **1996**, *14*, 33-38.
